# Src Tyrosine Kinase Activation by 4-Hydroxynonenal Upregulates p38, ERK/AP-1 Signaling and COX-2 Expression in YPEN-1 Cells

**DOI:** 10.1371/journal.pone.0129244

**Published:** 2015-10-14

**Authors:** Eun Ji Jang, Hyoung Oh Jeong, Daeui Park, Dae Hyun Kim, Yeon Ja Choi, Ki Wung Chung, Min Hi Park, Byung Pal Yu, Hae Young Chung

**Affiliations:** 1 Molecular Inflammation Research Center for Aging Intervention (MRCA), Department of Pharmacy, Pusan National University, Busan, Republic of Korea; 2 Interdisciplinary Research Program of Bioinformatics and Longevity Science, Pusan National University, Busan 609–735, Republic of Korea; 3 Department of Physiology, The University of Texas Health Science Center at San Antonio, San Antonio, TX 78229–3900, United States of America; Louisiana State University Health Sciences center, UNITED STATES

## Abstract

4-Hydroxynonenal (4-HNE), a major end product of lipid peroxidation, is highly reactive and involved in various cellular processes, such as inflammatory signaling. However, to date, the mechanistic roles of 4-HNE in inflammatory signaling related to protein tyrosine kinases have not been elucidated. In the present study, we investigated the interaction between 4-HNE and Src (a non-receptor tyrosine kinase) for its involvement in the molecular modulation of the inflammatory signaling pathway utilizing the YPEN-1 cell system. Immunoprecipitation experiments showed that 4-HNE phosphorylates (activates) Src at Tyr416 via adduct formation. In addition, LC-MS/MS and a docking simulation model revealed an addiction site at the Cys248 residue of Src, resulting in the stimulation of downstream p38, ERK/AP-1 and cyclooxygenase-2 (COX-2) signaling in YPEN-1 cells. The role of 4-HNE-activated Src in downstream inflammatory signaling was further investigated using dasatinib (a Src inhibitor) and by siRNA knockdown of Src. p38 and ERK were directly regulated by Src, as revealed by immunoblotting of the phosphorylated forms of mitogen-activated protein kinases (MAPKs), which are key elements in the signaling transduction pathway initiated by Src. The study also shows that Src modulates the HNE-enhanced activation of AP-1 and the expression of COX-2 (a target gene of AP-1). Together, the results of this study show that 4-HNE stimulates Src tyrosine kinase in activation of the inflammation process.

## Introduction

Oxidative stress and the peroxidation of cellular membrane arachidonic and linoleic acids [1] are major sources of several highly reactive aldehydes [2]. Among these, 4-hydroxy-2-nonenal (4-HNE) is the most active and abundant [3], and has been implicated in various vascular pathologies, such as atherosclerosis and cardiovascular diseases [4]. Studies identify elevated levels of 4-HNE adducted proteins in atherosclerotic lesions [5,6]. In our previous study of 4-HNE and its age-related serum changes in young and old rats, levels of free 4-HNE and 4-HNE-modified proteins were found to increase with age [7].

4-HNE contains highly reactive electrophilic moieties that form adducts with proteins by chemically modifying cysteine, histidine, or lysine residues. Although it has been shown that 4-HNE inactivates receptor tyrosine kinases (RTKs), such as epidermal growth factor receptor (EGFR) [8] and platelet-derived growth factor receptor (PDGFR) [9], the interactions between 4-HNE and non-receptor tyrosine kinases (NRTKs), like Src, have not been fully explored.

Src is one of the major NRTKs and is widely expressed in many cell types in different cellular locations. By interacting with many proteins related to signal transduction and cellular function, Src plays crucial roles in the regulation of cell proliferation, differentiation, adhesion, growth, movement, immune response, and other essential cellular functions.

Structurally, Src and its family members contain an SH3 domain, a phospho-tyrosine-binding SH2 domain, a kinase domain, and a C-terminal regulatory domain. When Src is inactivated, it folds upon itself by internal binding between the SH2 domain and phosphorylated Tyr527. Autophosphorylation of Tyr416 in the activation loop of Src leads to kinase activation, which is promoted by de-phosphorylation of Tyr527 by protein tyrosine phosphatases (PTP), or disruption of SH2 binding by SH2 phosphorylation or direct oxidation. Src is inactivated either by CSK-dependent phosphorylation of Tyr527 or by de-phosphorylation of Tyr416 [10].

One of the essential roles of Src upon activation is phosphorylation of a variety of proteins [11,12,13], some of which are involved in the inflammatory response [14]. Recent studies [15,16,17] describe an expanded role for Src via its involvement in MAPK activation. Three major MAPK isoforms, that is, p38, ERK, and JNK, are implicated in the regulation of inflammatory response. In relation to the various roles of MAPKs, such as regulating inflammation, cell cycle, cell death, cell development, cell differentiation, and cell senescence, p38 has been linked to the permeability [18], survival [19], and migration [20] of endothelial cells and ERK has been involved in proliferation[21,22] and inflammation[23,24] of endothelial cells. MAPKs are also known to transduce signaling cascades by phosphorylating and activating a group of transcription factors. The AP-1 superfamily is composed of proteins belonging to the c-Fos, c-Jun, ATF, and JDP families; and some AP-1 members are activated downstream of p38 and ERK. Furthermore, the p38 and ERK pathway (with the involvement of AP-1) plays essential roles in proinflammatory cytokines production [25], cancer invasion and metastasis[26,27], and the induction of enzymes such as COX-2 [28,29]. Notably, COX-2 is not detectable in most normal tissues, but its expression is selectively induced by various stimuli, whereupon it induces proinflammatory prostaglandins in several cell types, including vascular smooth muscle [30] and endothelial cells [31].

Many cardiovascular diseases arise from endothelial cell activation caused by proinflammatory and procoagulant states in the endothelial linings of blood vessel lumens. Furthermore, endothelial cell activation involves the activation of MAPKs [32] and the expression of proinflammatory proteins, such as COX-2 via AP-1 signaling [33,34]. Although 4-HNE-induced inflammatory signaling has been studied in many cell types [35,36], it has not been fully explored in endothelial cells. Because MAPKs are involved in the production of COX-2 via AP-1 and Src/MAPKs signaling, we considered that MAPKs/AP-1 signaling in endothelial cells following 4-HNE treatment is probably activated through Src.

In the present study, we investigated whether Src-4-HNE adduct formation upregulates MAPK/AP-1 signaling and proinflammatory COX-2 gene expression in YPEN-1 cells on behalf of endothelial cells using an Src inhibitor and knock down of Src by siRNA transfection. More significantly, we show how Src is directly modified and activated by 4-HNE using LC-MS/MS analysis.

## Materials and Methods

### Reagents and antibodies

4-HNE (purity >98%; Cat. No. 32100), was obtained from Cayman Chemical Inc. (Michigan, USA). Working solutions of HNE 20 mM (< 0.1% ethanol) were prepared in 100% ethanol immediately before use. Proto-oncogene c-Src Protein (GST Tag) was from Sinobiological Inc. (Beijing, China). 4-HNE and p-Src (Tyr416) antibodies were purchased from Abcam (Cambridge, MA, USA) and the other antibodies from Santa Cruz Biotechnology (Santa Cruz, CA, USA) and Cell Signaling Technology (New England, Hertfordshire, UK). Polyvinylidene difluoride (PVDF) membranes were obtained from the Millipore Corporation (Bedford, MA, USA). Dasatinib (Cat.No. D-3307) was purchased from LC labs (Woburn MA, USA).

### Cell culture conditions

YPEN-1 cells (a rat prostate endothelial cell line) were obtained from the ATCC (American Type Culture Collection, Manassas, Virginia, USA). The cells were grown in DMEM (Dulbecco's Modified Eagle Medium Media, Nissui, Tokyo, Japan) containing 2 mM L-glutamine, 100 mg/mL streptomycin, 2.5 mg/L amphotericin B, and 5% heat-inactivated fetal bovine serum (FBS). Cells were maintained at 37°C in a humidified 5% CO2/95% air atmosphere. Medium was replaced with fresh medium after 1 day to remove non-adherent cells and cell debris. Cells were discarded after 3 months and new cells were obtained from frozen stock.

### Preparation of cytosolic and nuclear extracts

Nuclear and cytosolic extracts were prepared as described by Deng et al. [37]. Treated cells were washed and then scraped into 1.0 ml of ice-cold PBS and pelleted at 3000 rpm for 5 min at 4°C. Pellets were suspended in 10 mM Tris (pH 8.0) containing 1.5 mM MgCl2, 1 mM dithiothreitol (DTT), 0.1% Nonidet P-40 (NP-40), and inhibitors, and then incubated on ice for 15 min. Nuclei were separated from cytosol by centrifugation at 12,000 rpm for 15 min at 4°C. Supernatants (cytosolic fractions) were removed and pellets were suspended in 10 mM Tris (pH 8.0) containing 50 mM KCl, 100 mM NaCl, and inhibitors, incubated on ice for 30 min, and then centrifuged at 12,000 rpm for 30 min at 4°C to obtain nuclear fractions.

### Western blotting

Western blotting was carried out as described previously [38]. Lysed samples were boiled for 5 min in a gel-loading buffer [0.125 M Tris–HCl (pH 6.8), 4% SDS, 10% 2-mercaptoethanol, and 0.2% bromophenol blue] at a volume ratio of 1:1. Total protein equivalents were separated by sodium dodecyl sulfate–polyacrylamide gel electrophoresis using 10% acrylamide gels as described by Laemmli [39], and blotted onto PVDF membranes at 100V for 1 h. Membranes were immediately placed into blocking buffer [1% nonfat milk in 10 mM Tris (pH 7.5), 100 mM NaCl, and 0.1% Tween 20] at room temperature for 30 min. Membranes were then incubated with specific primary antibodies at 25°C for 3 h, followed by horseradish peroxidase-conjugated anti-mouse antibody (Santa Cruz, 1:10,000), an anti-rabbit antibody (Santa Cruz, 1:10,000), or an anti-goat antibody (Santa Cruz, 1:10,000) at 25°C for 1 h. Antibody labeling was detected using West-zol Plus and chemiluminescence FluorchemTMSP (Alpha Innotech Corporation, San Leandro, CA, USA). Pre-stained protein markers were used for molecular weight determinations. All the raw data for the Western blotting are provided as supplementary data (S3 Data).

### Immunoprecipitation

Cell lysates were immunoprecipitated in a buffer containing 40 mM Tris, pH 7.6, 120 mM NaCl, 5 mM EDTA, 0.1% NP-40, protease inhibitors, and phosphatase inhibitors. Samples (300 μg) were precleared by incubation with a 50% slurry of protein A at 4°C for 2 h, centrifuged at 12,000 rpm at 4°C for 10 min. Pellets were then incubated for 3 h with respective antibodies at 4°C, and incubated overnight with a 50% slurry of protein A agarose at 4°C. After washing with buffer, immunoprecipitated proteins were analyzed by Western blotting as described previously [38]

### Immunocytochemistry

Cells were seeded on 35-mm culture dishes with cover slides and allowed to attach for 24 h. Medium was replaced with serum-free medium to observe the nuclear translocation of c-Jun in the absence of serum. After incubation with or without dasatinib for 30 min and transfection with Src siRNA for 48 h, cells were treated with 10 μM 4-HNE for 1 h, fixed in 4% paraformaldehyde in PBS (pH 7.4), and washed with PBS. Cells were then blocked in ABS/0.1% Triton X-100/3% goat serum (ABS-TS) at room temperature for 30 min and incubated with primary c-Jun antibody (rabbit polyclonal; Santa Cruz Biotechnology; 1:500) in ABS-TS at 4°C overnight. Cells were then rinsed in ABS-TS, washed in ABS, incubated for 3 h in the presence of anti-rabbit IgG labeled with Alexa Fluor-488, washed in ABS, and finally 1 mg/ml of Hoechst 33342 was added to label nuclei. Images were acquired using a Motic AE30/31 Inverted microscope (Motic Incorporation, Seoul, Korea).

### Src siRNA transfection

Small interfering RNA (siRNA) for Src and negative controls were purchased from Integrated DNA Technologies, Inc. (Coralville, IA, USA). The Src siRNA-targeting sequence was 5’-UCCAGAAUUGGUUGUAAAUACUUTG -3′ and the negative control siRNA-targeting sequence was 5’-CGUUAAUCGCGUAUAAUACGCGUAT -3′. For siRNA transfection, cells were seeded in six-well plates and grown for 24 h to reach 60–70% confluence. The cells were transfected with 25 nM Src siRNA or negative control siRNA using Lipofectamine 2000 (Invitrogen, Carlsbad, CA, USA).

### Luciferase assay

1 X 10^5^ cells per each well were cultured in 48-well plate and incubated overnight. 1μg AP-1 luciferase reporter vector was transfected to cells by using 1ul lipofectamine, a transfection reagent, per well with OPTI-MEM and serum-free media (OPTI-MEM-SFM, 1:50 mixture). After 4 h, the media was changed to DEME supplemented with 5%. After 20h, cells were treated with dasatinib (200 nM) was pretreated for 30 min before 4-HNE stimulation. Cells were lysed by 100 μl ONE-Glo Luciferase Assay System (Promega, Corporate)’s lysis buffer per well. And lysate is transferred to 96-white well and 50 μl ONE-Glo Luciferase Assay System (Promega, Corporate)’s substrate solution was added. Level of Luciferase expression was measured by a luminometer (Berthold)

### In-gel protein digestion

Src (10 μg) was treated with 4-HNE at molar ratio of 1:1 for 30 min at 37°C, loaded onto an SDS-PAGE gel and stained with Coomassie blue. Protein bands were excised and digested in-gel with sequencing grade modified trypsin (Promega, Madison, WI, USA), as previously described [40]. In brief, each protein spot was excised from the gel, placed in a polypropylene (Eppendorf) tube and washed 4–5 times (until the gel was clear) with 150 μl of 1:1 acetonitrile/25 mM ammonium bicarbonate (pH 7.8). Gel slices were dried in a Speedvac concentrator, rehydrated in 30 μl of 25 mM ammonium bicarbonate (pH 7.8) containing 20 ng of trypsin for 20 h at 37°C, and transferred to new tubes. Tryptic peptides remaining in gel matrix were extracted for 40 min at 30°C with 20 μl of 50% (v/v) aqueous acetonitrile containing 0.1% (v/v) formic acid. Combined supernatants were evaporated using a Speedvac concentrator and dissolved in 8 μl of 5% (v/v) aqueous acetonitrile solution containing 0.1% (v/v) formic acid for mass spectrometric analysis.

### Identification of proteins by LC-MS/MS

The resulting tryptic peptides were separated and analyzed by reversed phase capillary HPLC directly coupled to a Finnigan LCQ ion trap mass spectrometer (LC-MS/MS) [41] (with slight modification). Both of the 0.1 x 20 mm trappings and a 0.075 x 130 mm resolving column used were packed with Vydac 218MS low trifluoroactic acid C18 beads (5 μm, 300Å pore size; Vydac, Hesperia, CA, USA) and placed in-line. Peptides were bound to the trapping column for 10 min in 5% (v/v) aqueous acetonitrile containing 0.1% (v/v) formic acid, and subsequently, bound peptides were eluted using a 50 min gradient of 5–80% (v/v) acetonitrile containing 0.1% (v/v) formic acid at a flow rate of 0.2 μl/min. For tandem mass spectrometry, we used a mass scan range mode of 450–2000 Da. After determining the charge states of ions on zoom scans, product ion spectra were acquired in MS/MS mode at relative collision energy of 55%. The individual MS/MS spectra were processed using TurboSEQUEST software (Thermo Quest, San Jose, CA, USA). The peak list files generated were used to query either the MSDB database or NCBI using MASCOT (http://www.matrixscience.com). Modifications of methionine and cysteine, peptide mass tolerance at 2 Da, MS/MS ion mass tolerance at 0.8 Da, allowance for missed cleavage at 2, and charge states (+1, +2, and +3) were taken into account. Initially, only significant hits as defined by MASCOT probability analysis were considered. All the raw data for the LC-MS/MS data are provided as supplementary data (S1 Data, S2 Data).

### Docking simulation of the interaction between Src and 4-HNE

Of the many tools available for protein-ligand docking studies, AutoDock 4.2 was used for molecular docking simulation between Src and 4-HNE because of its level of automation [42]. The crystal structure of the active conformation of human c-Src (active conformation PDB ID: 1Y57) was used as a basis. We selected the SH2 domain in active Src protein for binding with 4-HNE. Docking simulation scores for interactions between ligands and receptors are calculated using different energy terms, such as, electrostatic energy, van der Waals energy, and solvation energy. Based on docking simulation results, we attempted to detect possible hydrogen bonds between Src and 4-HNE using the Ligandscout 3.0 program. Docking results were visualized using Chimera (http://www.cgl.ucsf.edu/chimera/). The ChemOffice program (http://www.cambridgesoft.com) was used to prepare 4-HNE for docking simulation. The following steps were taken: conversion of 2D structures into 3D structures, calculation of charges, and the addition of hydrogen atoms.

## Results

### Phosphorylation of Src by 4-HNE

**Fig 1:**
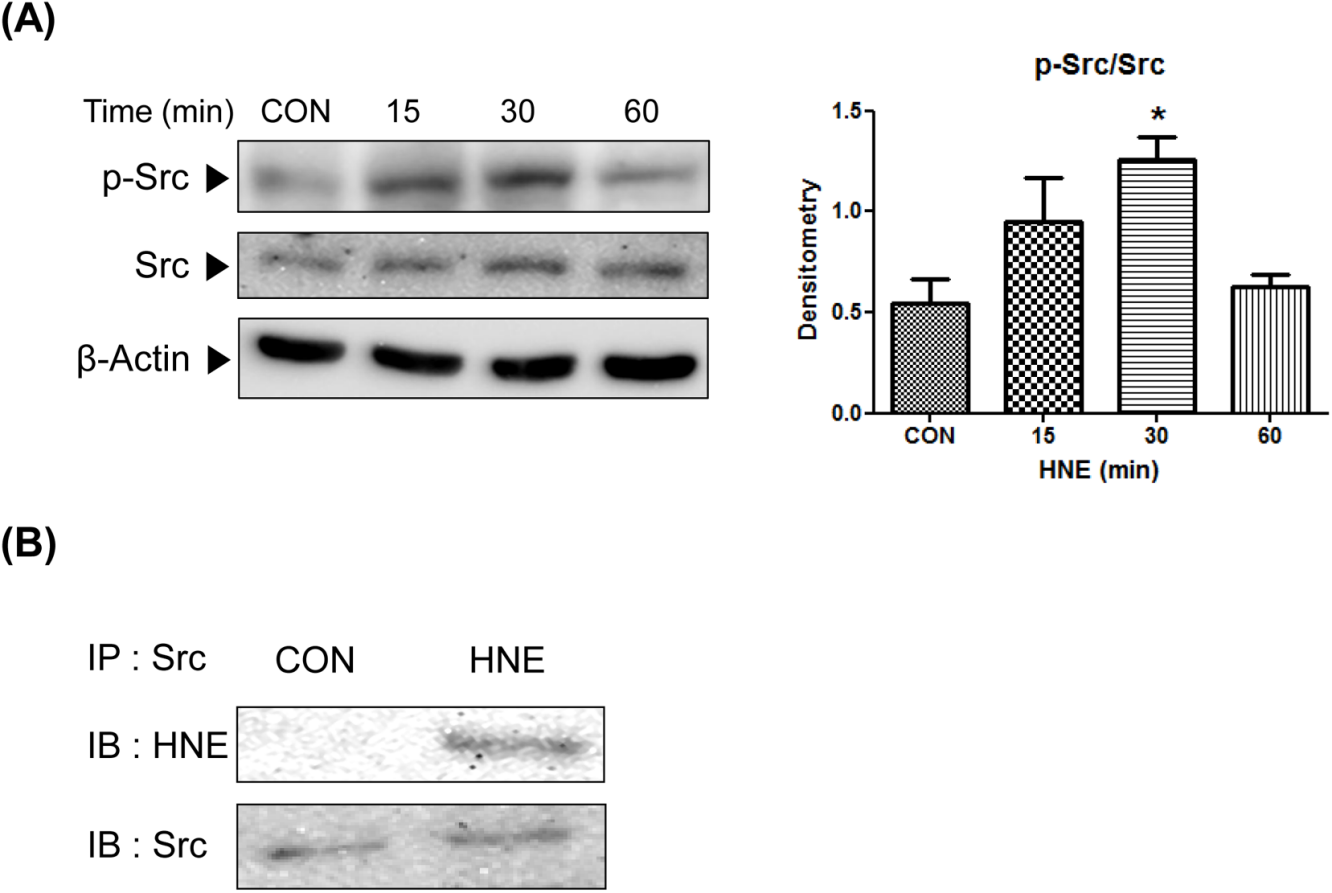
Phosphorylation and activation of Src by direct adduction with 4-HNE. (A) YPEN-1 cells were incubated in serum free medium with 10 μM 4-HNE for 15–60 min. Src phosphorylation was quantified densitometrically. Results are representative of at least three separate experiments. Statistical significance: *p < 0.05 *vs*. CON. (B) 4-HNE and Src were immunoblotted after immunoprecipitating Src in cells treated or not treated with 4-HNE 10 μM for 30 min. CON, control; HNE, 4-hydroxy-2-nonenal.

To determine whether Src is phosphorylated and activated by 4-HNE, p-Src (Tyr416) (the active form of Src) was quantified by Western blotting of YPEN-1 cells treated with 10 μM 4-HNE for 15–60 min. Densitometric analysis showed a significant, 2-fold increase in the level of Src phosphorylation versus controls in about 15–30 min (Fig 1A). To identify Src with 4-HNE adducts, total Src was immunoprecipitated and analyzed by Western blotting using 4-HNE and Src-specific antibodies. Data presented in Fig 1B show that 4-HNE highly adducted to Src in 4-HNE-treated cells. These results suggest that 4-HNE activates Src and forms adduction with Src in cytosol.

### Identification of the site of Src modification by 4-HNE and molecular docking

Further verification of the interaction between 4-HNE and Src was sought by molecular docking simulation. To obtain clues, specific residues in Src modified by 4-HNE, recombinant constitutively active GST-tagged Src was incubated with 4-HNE at a molar ratio of 1:1. This was followed by SDS-PAGE, band excision, tryptic digestion, and LC-MS/MS analysis. After trypsin digestion, 70% coverage of protein was obtained. LC-MS/MS showed that a 236–260 range of peptides in Src protein contained 4-HNE. The MASCOT results obtained that His236, Cys241, and Cys248 of Src directly interacted with 4-HNE with ion scores of 24 and 14 (Table 1), indicating that Cys248 of Src is more likely to be involved.

**Table 1:** Identification of the site of Src modification by 4-HNE using LC-MS/MS.

Start—End	Observed^a^	Mr(expt)^b^	Mr(calc)^c^	Delta	Sequence	Score^d^
236–260	923.5	2767.6	2767.6	0.0299	K.HADGL**C**HRLTTV**C**PTSKPQTQGLAK.D 2 HNE (H C)	24
236–260	923.3	2766.9	2768.5	-1.674	K.**H**ADGLCHRLTTV**x0C**PTSKPQTQGLAK.D Deamidation (NQ); 2 HNE (H C)	14

Sequence coverage: 70%; protein score: 28842; ion search cut off: 10. Src (10μg) was exposed to 4-HNE at a molar ratio of 1:1, digested with trypsin, and analyzed by LC MS/MS as described in Methods. Among ten ion search cut off, 4-HNE was detected in 236–260 range of amino acid. The peptide sequence containing 4-HNE adduct at amino acids, His236, Cys241, and Cys248 is denoted in bold font.

^a^ Observed values are those actually measured by LC MS/MS.

^b^ Mr(expt) are the experimental values of charge states.

^c^ Mr(calc) are relative molecular mass values calculated from the matched peptide sequence.

^d^ MASCOT scores are MS/MS ion score based on calculated probabilities.

**Fig 2:**
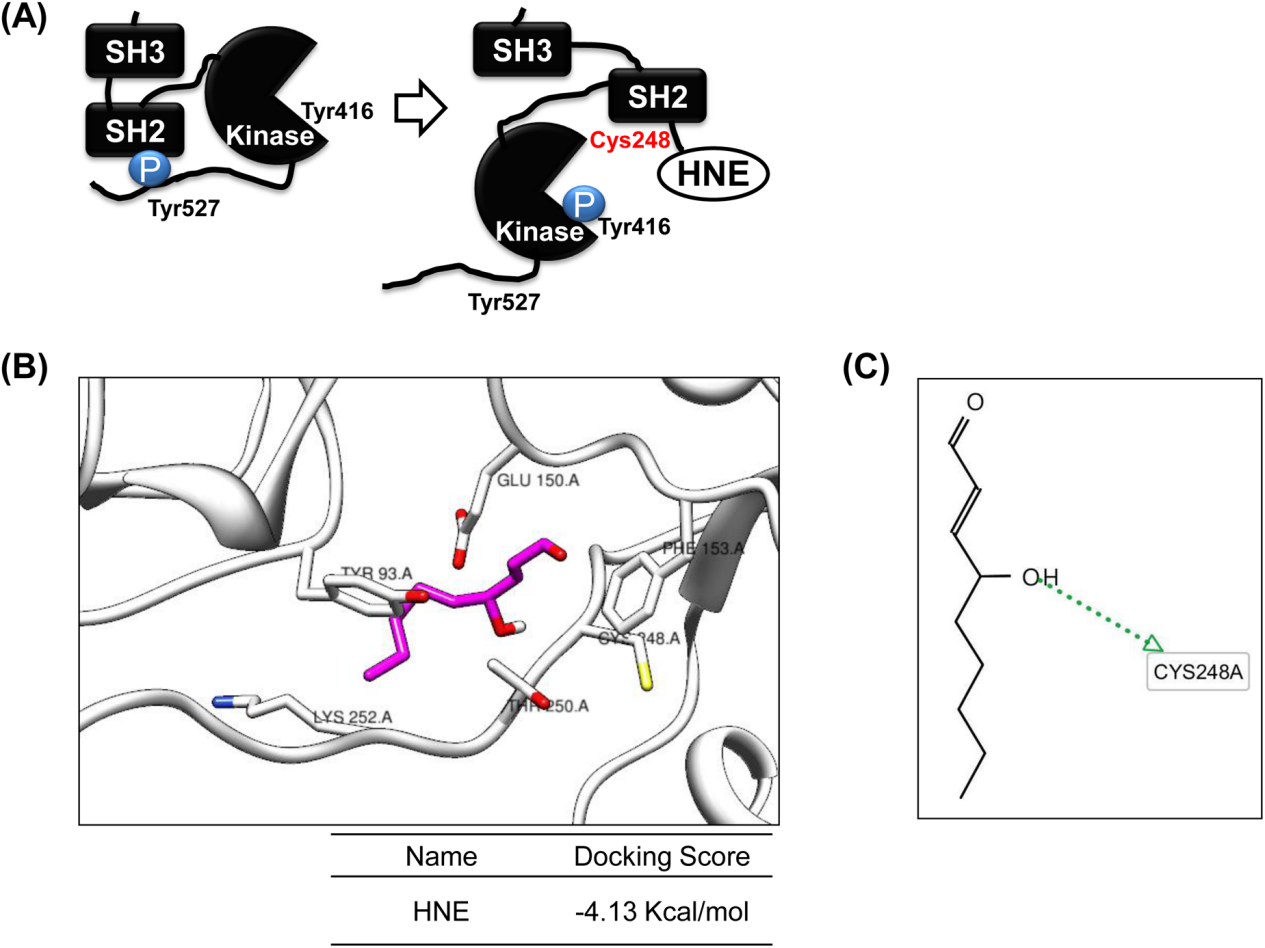
Simulation and conformational modeling of Src modified by 4-HNE. (A) Conformational modeling of Src modified by 4-HNE at Cys248 in its SH2 domain. (B) Docking simulation for the interaction between Src and 4-HNE using AutoDock 4.2 [42]. The SH2 domain in the crystal structure of human active c-Src constitutes a 4-HNE binding pocket. The binding energy of the 4-HNE/Src interaction was -4.13 kcal/mol. (C) hydrogen bonding between Src and 4-HNE as determined using the Ligand Scout program based on docking simulation results. It was predicted that certain residues of Src, notably Cys248, were mainly responsible for hydrogen bonding between 4-HNE and Src.

Src and its family members contain a SH3 domain, a phospho-tyrosine-binding SH2 domain, a kinase domain, and a C-terminal regulatory domain. When Src is inactivated, it folds upon itself by internal binding between the SH2 domain and phosphorylated Tyr527. Conformational changes caused by binding between 4-HNE and Cys248 of the Src SH2 domain induced the autophosphorylation of Tyr416 in the Src kinase domain and led to the activation of Src (Fig 2A). We performed docking simulation on Src and 4-HNE to confirm the LC-MS/MS results and to visualize the disposition of 4-HNE binding to the SH2 domain. Docking simulations provided significant AutoDock4.2 scores (Fig 2B). The binding energy of 4-HNE to Src was calculated at -4.13 kcal/mol. We also searched for hydrogen-bonding interactions between Src and 4-HNE using the LigandScout program based on docking simulation results. It was predicted that certain residues of Src, notably Cys248, were mainly responsible for hydrogen bonds with 4-HNE (Fig 2C). Furthermore, we expected the flexible conformation of Cys248 residue to improve docking accuracy, however this flexible side chain conformation didn’t show a consistent increase in scoring (-4.15 kcal/mol). Our data support that the site of Src modification by 4-HNE can be Cys248 in the SH2 domain of Src.

### Modulation of p38 and ERK by 4-HNE-activated Src

**Fig 3:**
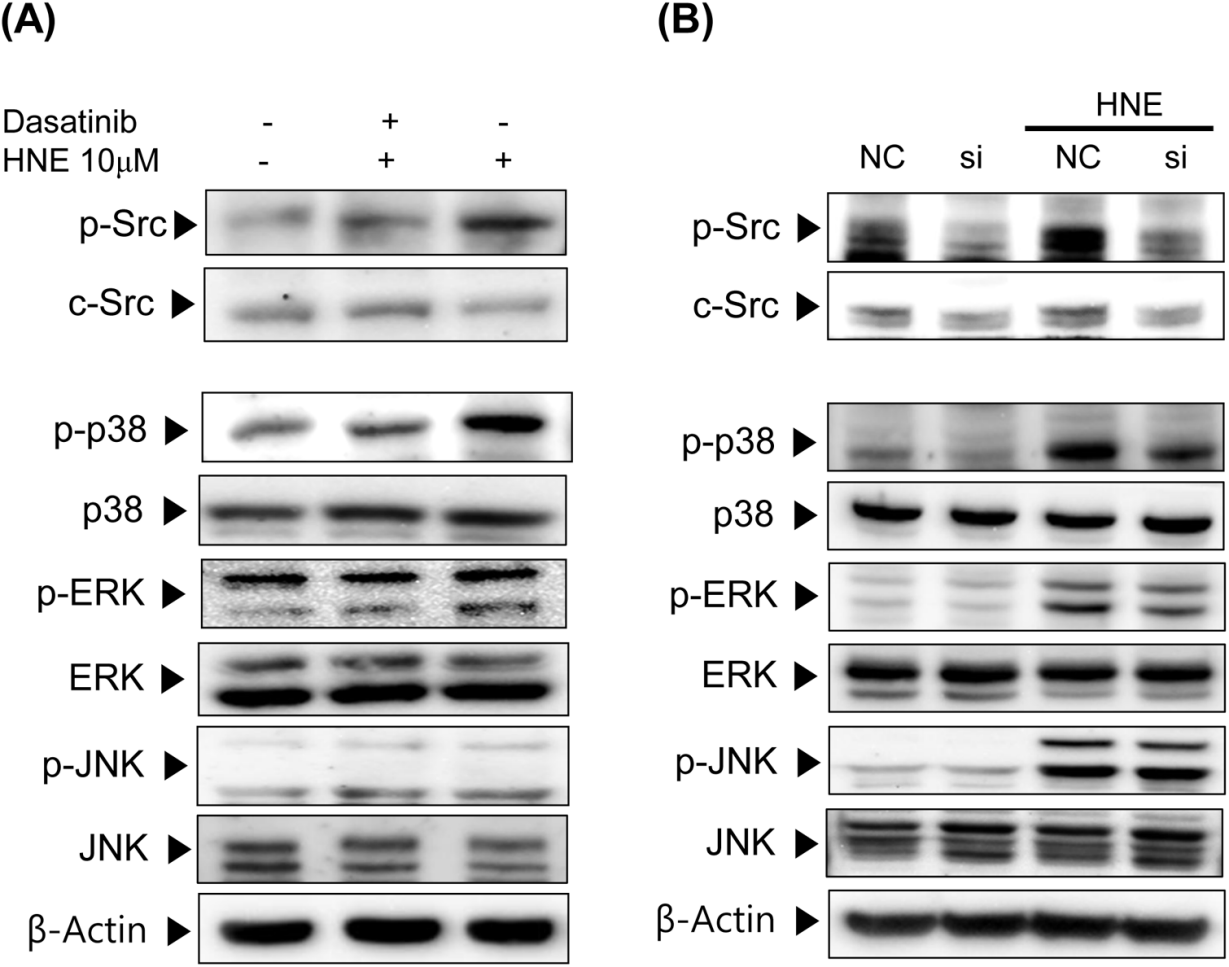
Involvement of Src in the 4-HNE-induced phosphorylation of p38, ERK and JNK. (A) YPEN-1 cells were pre-incubated with 300 nM of dasatinib (a Src inhibitor) for 30 min. After refreshing medium, 10 μM 4-HNE was added for 1 h. phospho-Src (Tyr416), phospho-ERK (Tyr204), phospho-p38 (Tyr182), phosphor-JNK (Thr183/Tyr185), the total form of each MAPK, and c-Src levels were analyzed by Western blotting. (B) YPEN-1 cells were transfected with Src siRNA(Si) or negative control siRNA(NC) for 48 h, and then exposed to 10 uM 4-HNE for 1 h. Phosphorylated MAPK and Src levels were evaluated by immunoblotting. AP-1, activator protein-1; COX-2, cyclooxygenase-2; ERK, extracellular signal-regulated kinase; HNE, 4-hydroxy-2-nonenal; JNK, c-Jun N-terminal kinase; MAPK, mitogen-activated protein kinase; NC, negative control; si, Src siRNA treated.

Because 4-HNE phosphorylated and activated all three MAPKs in YPEN-1 cells, we investigated whether Src is important for 4-HNE-induced MAPK activation. In Fig 3A, dasatinib (a specific Src inhibitor) was used to block the phosphorylation of Src, and was found to do so effectively at 300 nM. After pretreatment with dasatinib (300 nM; 30min), cells were incubated with 10 μM 4-HNE for an additional 1 h. The phosphorylation of p38 by 4-HNE was significantly blocked by dasatinib, and it showed slight effect on ERK, especially ERK2 (the lower band), but no effect was observed on JNK. In YPEN-1 cells transfected with Src siRNA, the 4-HNE-enhanced phosphorylation of p38 and ERK was rather abolished (Fig 3B), confirming the importance of the Src-related MAPKs signaling pathway in the action of 4-HNE. Consistent with the effect of dasatinib on MAPKs signaling, JNK were almost unaffected. These results suggest that Src participates in the 4-HNE-induced p38 and ERK phosphporylation (activation).

### Activation of AP-1 (c-Jun) by the 4-HNE- Src adduct

**Fig 4:**
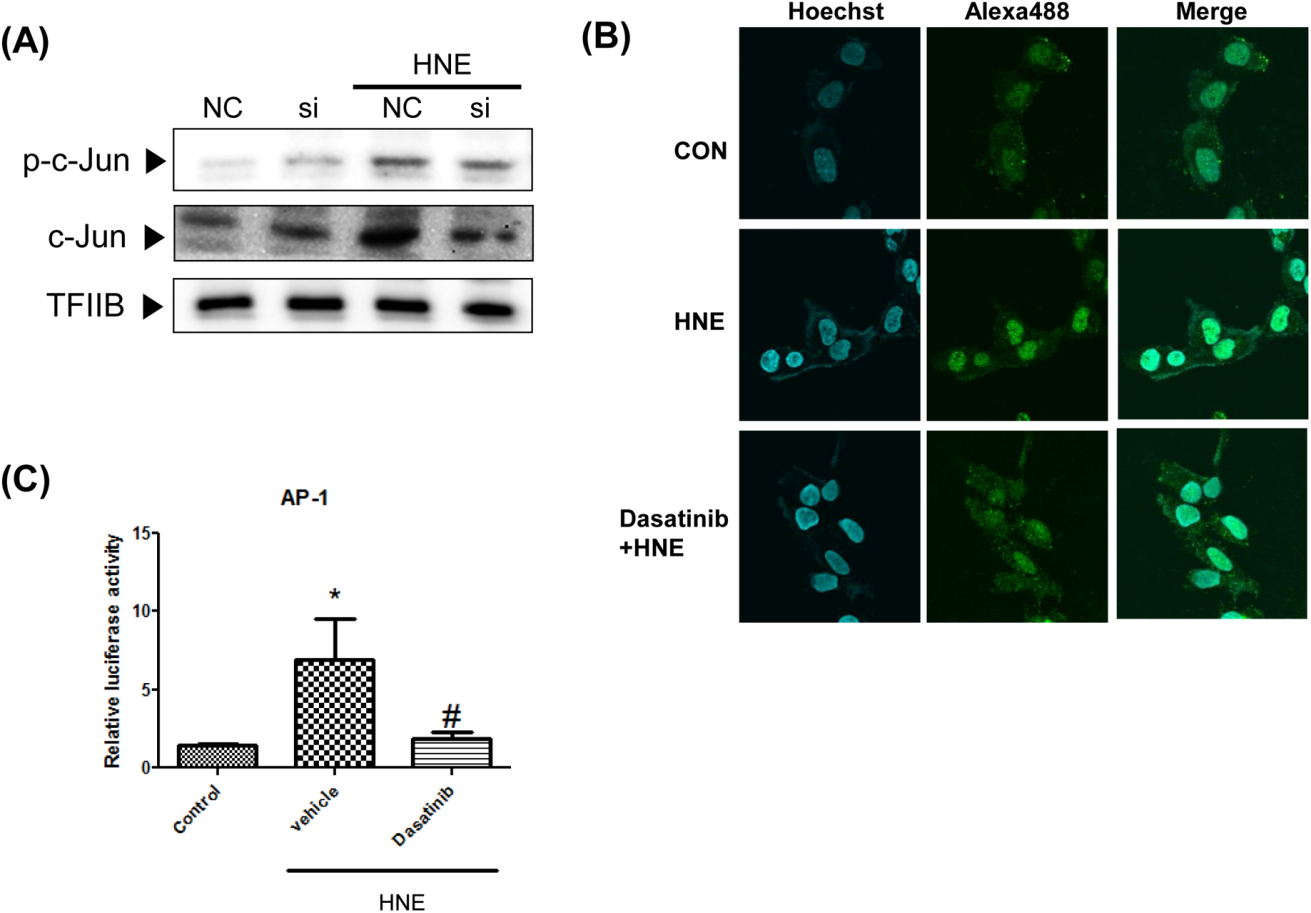
Mediation of Src in 4-HNE-induced enhancement of AP-1 activity in YPEN-1 cells (A) YPEN-1 cells were transfected with Src siRNA for 48 h, treated with HNE (10 μM), and immunoblotted for c-Jun and phosphor-c-Jun. (B) The nuclear translocation of c-Jun was visualized by immunocytochemical staining. Nuclei were stained with Hoechst dye (blue) and alexa-488-conjugated (green) goat anti-rabbit IgG to detect c-Jun antibody. Merged images are shown on the right. (D) AP-1 activities were measured by luciferase reporter assay in cells transfected with AP-1 luciferase reporter constructs. The Src inhibitor dasatinib (200 nM) was pretreated for 30 min before 4-HNE stimulation. Bars represent means±SEs (n = 4).*p<0.05 *vs*. control, #p<0.05 *vs*. vehicle. AP-1, activator protein-1; CON, control; HNE, 4-hydroxy-2-nonenal; NC, negative control; si, Src siRNA treated; TFIIB, transcription factor II B.

Based upon the finding that 4-HNE-activated p38 and ERK via Src, the transcription factor AP-1 was viewed as a downstream component of the p38 and ERK signaling pathway. To determine whether Src acts as a mediator of 4-HNE-enhanced AP-1 activation, we treated YPEN-1 cells with Src siRNA (Fig 4A). The phosphorylation of c-Jun (a subunit of AP-1) was down regulated by Src siRNA transfection. Furthermore, immunocytochemistry confirmed that the nuclear translocation of c-Jun was inhibited by dasatinib. To investigate the ability of Src to inhibit the 4-HNE-mediated transcriptional activation of AP-1, cells were transfected with a reporter plasmid, in which luciferase expression was controlled by a promoter containing AP-1-binding sites. In cells treated with 4-HNE, the luciferase activity of AP-1 was markedly enhanced, and this enhancement was blocked by pretreating cells with dasatinib. These results indicate that Src also functions as a bridge, possibly via p38 and ERK that increases the AP-1 signaling pathway regulated by 4-HNE.

### Up-regulation of COX-2 gene expression by 4-HNE-Src adduct

**Fig 5:**
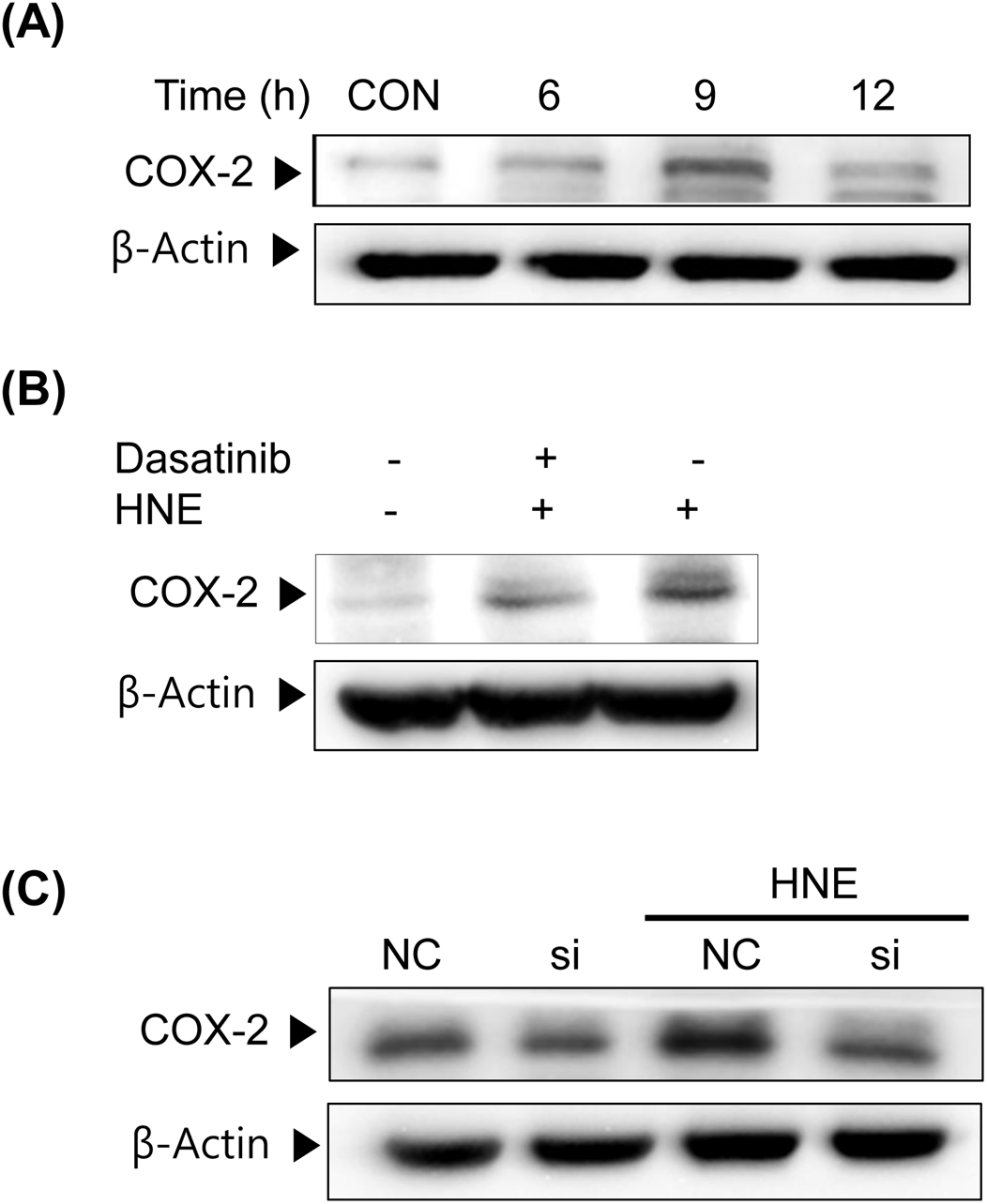
Effects of Src on HNE-induced COX-2 expression in YPEN cells. (A) YPEN-1 cells were stimulated with 5 μM HNE for the indicated times (0–12 h). COX-2 protein expressions in cell lysates were analyzed by immunoblotting. (B) After pretreatment with dasatinib (300 nM) for 30 min, cells were stimulated with 5 μM 4-HNE for 9 h. (C) YPEN-1 cells were transfected with Src siRNA for 48 h and then treated with 5 μM HNE for 9 h. COX-2 protein expression levels were determined by immunoblotting. CON, control; NC, negative control; si, Src siRNA-treated cells; HNE, 4-hydroxy-2-nonenal; COX-2, cyclooxygenase-2.

COX-2 (targeted by AP-1) protein levels were increased when YPEN-1 cells were treated with 5 μM 4-HNE for 6–12 h (Fig 5A). To define the role of Src with respect to the expression of COX-2, we used dasatinib (Fig 5B) and Src siRNA (Fig 5C). When Src was inhibited, COX-2 upregulation by 4-HNE was diminished. These findings demonstrate the participation of Src in the 4-HNE-induced expression of COX-2.

## Discussion

It is well documented that under conditions of chronic oxidative stress or inflammation, 4-HNE accumulates within cells and can initiate cellular damage. 4-HNE is a highly reactive major end-product of lipid peroxidation and has been causally associated with various degenerative disorders, including cardiovascular disease and atherosclerosis [4,42]. Among the lipid aldehydes, 4-HNE is most deleterious because of its high reactivity [4,43], but to date the mechanism by which 4-HNE acts on endothelial cell function has not been elucidated.

The present study demonstrates the ability of 4-HNE to induce the activation of Src via thiol-modified adduct formation and the subsequent increase in COX-2 expression via the MAPKs/AP-1 signaling pathway in YPEN-1 cells representing endothelial cells. Furthermore, the results show that the increased activities of p38, ERK and AP-1 and subsequent COX-2 expression by 4-HNE were significantly attenuated by inhibition of Src. Thus, these results support the hypothesis that 4-HNE enhances endothelial COX-2 production via the Src-mediated activation of p38, ERK/AP-1 pathways.

It has been previously reported that oxidized LDL and related lipid peroxidation products, including 4-HNE, can interact with EGFR and PDGFR and activate downstream signaling pathways [44]. Furthermore, because EGFR and PDGFR are known as upstream kinases of Src [45,46], further studies are needed to rule out the possibility of 4-HNE-induced upstream kinase activation, for example, making cells double knocked down of Src and EGFR. However, the exact domain or residue of RTK, which is modulated by 4-HNE, has not been identified. Another unexplored aspect of 4-HNE is the possible interaction with NRTKs, such as Src, that are widely involved in various signaling pathways. Our results demonstrate that the stimulation of YPEN-1 cells with 4-HNE increases Src activity; the direct binding between 4-HNE and Src within cells was verified by immunoprecipitation. In addition, LC-MS MS enabled us to identify that the specific 4-HNE binding site was Cys 248 in the SH2 domain of Src, binding which led to a conformational change. However, to certain this finding, it needs additional validation using appropriate deletion mutants of Src which lack Cys 248 residue. Moreover, although the activation of Src by 4-HNE in YPEN-1 cells was verified, further experiments are needed to identify the adduction of Src by 4-HNE leading to phosphorylation (activation) of Src.

Because 4-HNE is known to activate MAPKs [32], that is, ERK [47], JNK [48], and p38, we sought to determine how Src activation modulates these pro-inflammatory transcriptional factors. Our Src knock down experiments in YPEN-1 cells and dasatinib studies show that reductions in Src activity affected p38 and ERK activation, suggesting that Src mediates 4-HNE- induced p38 and ERK activation, not those of JNK. Actually, it has been previously suggested that p38 [49,50] and ERK [16,23] are downstream targets of Src.

Among the known p38 and ERK-regulated transcription factors, such as AP-1, NF-κB, SP-1 and p53, AP-1(c-Jun) was selected more notably as a downstream target of p38 and ERK in the present study because of its importance in inflammatory gene regulation [51]. In addition, the activations of the AP-1 components, c-Jun and c-fos by lipid peroxides [52] may be relevant in the context of disease development, such as tumor progression [53]. In the case of c-Jun, our results show that its activity was upregulated by 4-HNE, and downregulated by Src inhibition. Although c-Jun is usually phosphorylated by JNK, new evidence suggests that p38 [54,55,56] and ERK [57] also phosphorylate c-Jun and regulate its transcriptional activity. Therefore, it is plausible that p38 and ERK is an upstream signaling molecule for c-Jun.

Evidence shows that the expression of COX-2 is regulated by AP-1 [58], and that the p38, ERK/AP-1-dependent expression of COX-2 regulates the inflammatory response [28,29]. Specifically, Zarrouki et al.[30] reported that 4-HNE induces COX-2 expression via p38 activation in 3T3-L1 adipose cells. These findings are consistent with our notion that the Src-dependent activations of the p38, ERK and c-Jun pathways enhance COX-2 expression in endothelial cells. Accordingly, we suggest that the Src-dependent p38, ERK/c-Jun pathway is a major regulator of 4-HNE-induced COX-2 expression in YPEN-1 cells.

COX-2 is largely responsible for inflammation, a common disease mechanism in cardiovascular disease, atherosclerosis, and other diseases [59]. Furthermore, many studies have shown that selective COX-2 inhibition improves endothelial function and ameliorates cardiovascular disease [33]. On the other hand, what we show in the current study is evidence that the inhibition of Src could improve 4-HNE-induced endothelial dysfunction, which is caused in part by COX-2.

In summary, the present study provides evidence of the 4-HNE induced activation of Src and the subsequent activation of the p38 and ERK pathway and the upregulation of c-Jun in endothelial cells. These series of reactions could contribute to 4-HNE-induced COX-2 production, and thereby contribute to the development of atherosclerosis. Although the present study shows that HNE enhances c-Jun activity and COX-2 expression via Src signaling only in YPEN-1 cells, further studies are needed to identify that it applies to other endothelial cells for the assurance that 4-HNE and Src can be key molecules in endothelial inflammation. Furthermore, it is possible that strategies targeting the modulation of Src activity could have significant therapeutic potential in terms of attenuating the chronic inflammatory state induced by 4-HNE accumulation.

## Supporting Information

S1 Data Mascot search results (Protein view). (DOCX)

S2 Data Mascot search results (Probability Based Mowse Score). (DOCX)

S3 Data Raw data for all the western results. (PPTX)
