# Supplementary material for: Src Tyrosine Kinase Activation by 4-Hydroxynonenal Upregulates p38, ERK/AP-1 Signaling and COX-2 Expression in YPEN-1 Cells
Source: PLoS One. 2015 Oct 14;10(10):e0129244. doi: 10.1371/journal.pone.0129244 (PMC4605600; doi:10.1371/journal.pone.0129244)
Supplement: S1 Data — (DOCX) [file pone.0129244.s001.docx]

**
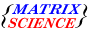
Mascot Search Results**

**Protein View**

양식의 맨 위

Match to: **gi|00000001** Score: **28842**

**Busan JEJ**

Found in search of JEJ.xml

Nominal mass (M_r_): **59797**; Calculated pI value: **7.10**

NCBI BLAST search of [gi|00000001](http://www.ncbi.nlm.nih.gov/blast/Blast.cgi?ALIGNMENTS=50&ALIGNMENT_VIEW=Pairwise&AUTO_FORMAT=Semiauto&CDD_SEARCH=on&CLIENT=web&COMPOSITION_BASED_STATISTICS=on&DATABASE=nr&DESCRIPTIONS=100&ENTREZ_QUERY=(none)&EXPECT=10&FILTER=L&FORMAT_BLOCK_ON_RESPAGE=None&FORMAT_OBJECT=Alignment&FORMAT_TYPE=HTML&GAPCOSTS=11+1&I_THRESH=0.001&LAYOUT=TwoWindows&MATRIX_NAME=BLOSUM62&NCBI_GI=on&PAGE=Proteins&PROGRAM=blastp&QUERY=MGSNKSKPKDASQRRRSLEPAENVHGAGGGAFPASQTPSKPASADGHRGPSAAFAPAAAEPKLFGGFNSSDTVTSPQRAGPLAGGVTTFVALYDYESRTETDLSFKKGERLQIVNNTEGDWWLAHSLSTGQTGYIPSNYVAPSDSIQAEEWYFGKITRRESERLLLNAENPRGTFLVRESETTKGAYCLSVSDFDNAKGLNVKHYKIRKLDSGGFYITSRTQFNSLQQLVAYYSKHADGLCHRLTTVCPTSKPQTQGLAKDAWEIPRESLRLEVKLGQGCFGEVWMGTWNGTTRVAIKTLKPGTMSPEAFLQEAQVMKKLRHEKLVQLYAVVSEEPIYIVTEYMSKGSLLDFLKGETGKYLRLPQLVDMAAQIASGMAYVERMNYVHRDLRAANILVGENLVCKVADFGLARLIEDNEYTARQGAKFPIKWTAPEAALYGRFTIKSDVWSFGILLTELTTKGRVPYPGMVNREVLDQVERGYRMPCPPECPESLHDLMCQCWRKEPEERPTFEYLQAFLEDYFTSTEPQYQPGENL&SERVICE=plain&SET_DEFAULTS.x=9&SET_DEFAULTS.y=5&SHOW_OVERVIEW=on&WORD_SIZE=3&END_OF_HTTPGET=Yes) against nr

Unformatted [sequence string](http://df3/mascot/cgi/getseq.pl?mina50+gi%7c00000001+seq) for pasting into other applications

Variable modifications: Carbamidomethyl (C),Deamidation (NQ),Oxidation (M),HNE (H C)

Cleavage by Trypsin: cuts C-term side of KR unless next residue is P

Sequence Coverage: **70%**

Matched peptides shown in **Bold Red**

**1** MGSNKSKPKD ASQRRR**SLEP AENVHGAGGG AFPASQTPSK PASADGHRGP**

**51 SAAFAPAAAE PKLFGGFNSS DTVTSPQRAG PLAGGVTTFV ALYDYESR**TE

**101** TDLSFKKGER LQIVNNTEGD WWLAHSLSTG QTGYIPSNYV APSDSIQAEE

**151** WYFGKITRR**E SERLLLNAEN PRGTFLVRES ETTKGAYCLS VSDFDNAKGL**

**201 NVK**HYKIR**KL DSGGFYITSR TQFNSLQQLV AYYSKHADGL CHRLTTVCPT**

**251 SKPQTQGLAK DAWEIPRESL R**LEVK**LGQGC FGEVWMGTWN GTTRVAIKTL**

**301 KPGTMSPEAF LQEAQVMKK**L RHEKLVQLYA VVSEEPIYIV TEYMSK**GSLL**

**351 DFLKGETGK**Y LR**LPQLVDMA AQIASGMAYV ER**MNYVHR**DL RAANILVGEN**

**401 LVCKVADFGL ARLIEDNEYT ARQGAKFPIK WTAPEAALYG RFTIKSDVWS**

**451 FGILLTELTT KGRVPYPGMV NREVLDQVER** GYR**MPCPPEC PESLHDLMCQ**

**501 CWRK**EPEERP TFEYLQAFLE DYFTSTEPQY QPGENL


 Residue Number Increasing Mass Decreasing Mass

**Start - End Observed Mr(expt) Mr(calc) Delta Miss Sequence**

**17 - 48 1034.5007 3100.4804 3099.4757 1.0046 0 R.SLEPAENVHGAGGGAFPASQTPSKPASADGHR.G**  ([Ions score 102](http://df3/mascot/cgi/peptide_view.pl?file=../data/20121119/F002569.dat&query=2832&hit=1&index=gi%7c00000001&px=1))

**17 - 48 1034.5428 3100.6067 3100.4597 0.1470 0 R.SLEPAENVHGAGGGAFPASQTPSKPASADGHR.G**  Deamidation (NQ) ([Ions score 92](http://df3/mascot/cgi/peptide_view.pl?file=../data/20121119/F002569.dat&query=2833&hit=1&index=gi%7c00000001&px=1))

**17 - 48 1034.7202 3101.1388 3100.4597 0.6791 0 R.SLEPAENVHGAGGGAFPASQTPSKPASADGHR.G**  Deamidation (NQ) ([Ions score 96](http://df3/mascot/cgi/peptide_view.pl?file=../data/20121119/F002569.dat&query=2834&hit=1&index=gi%7c00000001&px=1))

**49 - 62 642.3018 1282.5890 1283.6509 -1.0620 0 R.GPSAAFAPAAAEPK.L**  ([Ions score 92](http://df3/mascot/cgi/peptide_view.pl?file=../data/20121119/F002569.dat&query=1346&hit=1&index=gi%7c00000001&px=1))

**49 - 62 642.6071 1283.1996 1283.6509 -0.4514 0 R.GPSAAFAPAAAEPK.L**  ([Ions score 85](http://df3/mascot/cgi/peptide_view.pl?file=../data/20121119/F002569.dat&query=1347&hit=1&index=gi%7c00000001&px=1))

**49 - 62 642.8814 1283.7483 1283.6509 0.0973 0 R.GPSAAFAPAAAEPK.L**  ([Ions score 60](http://df3/mascot/cgi/peptide_view.pl?file=../data/20121119/F002569.dat&query=1348&hit=1&index=gi%7c00000001&px=1))

**49 - 62 643.3041 1284.5936 1283.6509 0.9427 0 R.GPSAAFAPAAAEPK.L**  ([Ions score 45](http://df3/mascot/cgi/peptide_view.pl?file=../data/20121119/F002569.dat&query=1350&hit=1&index=gi%7c00000001&px=1))

**49 - 62 643.3463 1284.6780 1283.6509 1.0270 0 R.GPSAAFAPAAAEPK.L**  ([Ions score 38](http://df3/mascot/cgi/peptide_view.pl?file=../data/20121119/F002569.dat&query=1351&hit=1&index=gi%7c00000001&px=1))

**49 - 62 643.5546 1285.0946 1283.6509 1.4436 0 R.GPSAAFAPAAAEPK.L**  ([Ions score 66](http://df3/mascot/cgi/peptide_view.pl?file=../data/20121119/F002569.dat&query=1352&hit=1&index=gi%7c00000001&px=1))

**49 - 62 643.5814 1285.1483 1283.6509 1.4974 0 R.GPSAAFAPAAAEPK.L**  ([Ions score 76](http://df3/mascot/cgi/peptide_view.pl?file=../data/20121119/F002569.dat&query=1353&hit=1&index=gi%7c00000001&px=1))

**63 - 78 855.9208 1709.8271 1711.8165 -1.9894 0 K.LFGGFNSSDTVTSPQR.A**  ([Ions score 67](http://df3/mascot/cgi/peptide_view.pl?file=../data/20121119/F002569.dat&query=2411&hit=1&index=gi%7c00000001&px=1))

**63 - 78 855.9243 1709.8340 1711.8165 -1.9826 0 K.LFGGFNSSDTVTSPQR.A**  ([Ions score 68](http://df3/mascot/cgi/peptide_view.pl?file=../data/20121119/F002569.dat&query=2412&hit=1&index=gi%7c00000001&px=1))

**63 - 78 855.9391 1709.8636 1711.8165 -1.9529 0 K.LFGGFNSSDTVTSPQR.A**  ([Ions score 33](http://df3/mascot/cgi/peptide_view.pl?file=../data/20121119/F002569.dat&query=2413&hit=1&index=gi%7c00000001&px=1))

**63 - 78 856.2296 1710.4446 1711.8165 -1.3720 0 K.LFGGFNSSDTVTSPQR.A**  ([Ions score 118](http://df3/mascot/cgi/peptide_view.pl?file=../data/20121119/F002569.dat&query=2414&hit=1&index=gi%7c00000001&px=1))

**63 - 78 856.3000 1710.5854 1711.8165 -1.2311 0 K.LFGGFNSSDTVTSPQR.A**  ([Ions score 116](http://df3/mascot/cgi/peptide_view.pl?file=../data/20121119/F002569.dat&query=2415&hit=1&index=gi%7c00000001&px=1))

**63 - 78 856.3538 1710.6930 1711.8165 -1.1235 0 K.LFGGFNSSDTVTSPQR.A**  ([Ions score 84](http://df3/mascot/cgi/peptide_view.pl?file=../data/20121119/F002569.dat&query=2416&hit=1&index=gi%7c00000001&px=1))

**63 - 78 856.3703 1710.7260 1711.8165 -1.0905 0 K.LFGGFNSSDTVTSPQR.A**  ([Ions score 75](http://df3/mascot/cgi/peptide_view.pl?file=../data/20121119/F002569.dat&query=2417&hit=1&index=gi%7c00000001&px=1))

**63 - 78 856.3768 1710.7390 1711.8165 -1.0775 0 K.LFGGFNSSDTVTSPQR.A**  ([Ions score 78](http://df3/mascot/cgi/peptide_view.pl?file=../data/20121119/F002569.dat&query=2418&hit=1&index=gi%7c00000001&px=1))

**63 - 78 856.3922 1710.7699 1711.8165 -1.0466 0 K.LFGGFNSSDTVTSPQR.A**  ([Ions score 59](http://df3/mascot/cgi/peptide_view.pl?file=../data/20121119/F002569.dat&query=2419&hit=1&index=gi%7c00000001&px=1))

**63 - 78 856.4297 1710.8449 1711.8165 -0.9716 0 K.LFGGFNSSDTVTSPQR.A**  ([Ions score 105](http://df3/mascot/cgi/peptide_view.pl?file=../data/20121119/F002569.dat&query=2420&hit=1&index=gi%7c00000001&px=1))

**63 - 78 856.4412 1710.8678 1711.8165 -0.9487 0 K.LFGGFNSSDTVTSPQR.A**  ([Ions score 98](http://df3/mascot/cgi/peptide_view.pl?file=../data/20121119/F002569.dat&query=2421&hit=1&index=gi%7c00000001&px=1))

**63 - 78 856.4602 1710.9059 1711.8165 -0.9107 0 K.LFGGFNSSDTVTSPQR.A**  ([Ions score 71](http://df3/mascot/cgi/peptide_view.pl?file=../data/20121119/F002569.dat&query=2422&hit=1&index=gi%7c00000001&px=1))

**63 - 78 856.4843 1710.9540 1711.8165 -0.8626 0 K.LFGGFNSSDTVTSPQR.A**  ([Ions score 98](http://df3/mascot/cgi/peptide_view.pl?file=../data/20121119/F002569.dat&query=2423&hit=1&index=gi%7c00000001&px=1))

**63 - 78 856.5194 1711.0243 1711.8165 -0.7922 0 K.LFGGFNSSDTVTSPQR.A**  ([Ions score 88](http://df3/mascot/cgi/peptide_view.pl?file=../data/20121119/F002569.dat&query=2424&hit=1&index=gi%7c00000001&px=1))

**63 - 78 856.5296 1711.0447 1711.8165 -0.7719 0 K.LFGGFNSSDTVTSPQR.A**  ([Ions score 105](http://df3/mascot/cgi/peptide_view.pl?file=../data/20121119/F002569.dat&query=2425&hit=1&index=gi%7c00000001&px=1))

**63 - 78 856.7506 1711.4867 1711.8165 -0.3298 0 K.LFGGFNSSDTVTSPQR.A**  ([Ions score 120](http://df3/mascot/cgi/peptide_view.pl?file=../data/20121119/F002569.dat&query=2426&hit=1&index=gi%7c00000001&px=1))

**63 - 78 856.7660 1711.5174 1711.8165 -0.2991 0 K.LFGGFNSSDTVTSPQR.A**  ([Ions score 90](http://df3/mascot/cgi/peptide_view.pl?file=../data/20121119/F002569.dat&query=2427&hit=1&index=gi%7c00000001&px=1))

**63 - 78 856.8199 1711.6253 1711.8165 -0.1912 0 K.LFGGFNSSDTVTSPQR.A**  ([Ions score 106](http://df3/mascot/cgi/peptide_view.pl?file=../data/20121119/F002569.dat&query=2428&hit=1&index=gi%7c00000001&px=1))

**63 - 78 856.8220 1711.6295 1711.8165 -0.1870 0 K.LFGGFNSSDTVTSPQR.A**  ([Ions score 89](http://df3/mascot/cgi/peptide_view.pl?file=../data/20121119/F002569.dat&query=2429&hit=1&index=gi%7c00000001&px=1))

**63 - 78 856.8235 1711.6324 1711.8165 -0.1841 0 K.LFGGFNSSDTVTSPQR.A**  ([Ions score 74](http://df3/mascot/cgi/peptide_view.pl?file=../data/20121119/F002569.dat&query=2430&hit=1&index=gi%7c00000001&px=1))

**63 - 78 856.8431 1711.6716 1711.8165 -0.1449 0 K.LFGGFNSSDTVTSPQR.A**  ([Ions score 114](http://df3/mascot/cgi/peptide_view.pl?file=../data/20121119/F002569.dat&query=2431&hit=1&index=gi%7c00000001&px=1))

**63 - 78 856.8486 1711.6826 1711.8165 -0.1339 0 K.LFGGFNSSDTVTSPQR.A**  ([Ions score 93](http://df3/mascot/cgi/peptide_view.pl?file=../data/20121119/F002569.dat&query=2432&hit=1&index=gi%7c00000001&px=1))

**63 - 78 856.8495 1711.6844 1711.8165 -0.1321 0 K.LFGGFNSSDTVTSPQR.A**  ([Ions score 93](http://df3/mascot/cgi/peptide_view.pl?file=../data/20121119/F002569.dat&query=2433&hit=1&index=gi%7c00000001&px=1))

**63 - 78 856.8703 1711.7260 1711.8165 -0.0905 0 K.LFGGFNSSDTVTSPQR.A**  ([Ions score 102](http://df3/mascot/cgi/peptide_view.pl?file=../data/20121119/F002569.dat&query=2434&hit=1&index=gi%7c00000001&px=1))

**63 - 78 856.8805 1711.7464 1711.8165 -0.0701 0 K.LFGGFNSSDTVTSPQR.A**  ([Ions score 108](http://df3/mascot/cgi/peptide_view.pl?file=../data/20121119/F002569.dat&query=2435&hit=1&index=gi%7c00000001&px=1))

**63 - 78 856.8973 1711.7801 1711.8165 -0.0364 0 K.LFGGFNSSDTVTSPQR.A**  ([Ions score 108](http://df3/mascot/cgi/peptide_view.pl?file=../data/20121119/F002569.dat&query=2436&hit=1&index=gi%7c00000001&px=1))

**63 - 78 856.9088 1711.8030 1711.8165 -0.0136 0 K.LFGGFNSSDTVTSPQR.A**  ([Ions score 80](http://df3/mascot/cgi/peptide_view.pl?file=../data/20121119/F002569.dat&query=2437&hit=1&index=gi%7c00000001&px=1))

**63 - 78 856.9158 1711.8170 1711.8165 0.0005 0 K.LFGGFNSSDTVTSPQR.A**  ([Ions score 117](http://df3/mascot/cgi/peptide_view.pl?file=../data/20121119/F002569.dat&query=2438&hit=1&index=gi%7c00000001&px=1))

**63 - 78 856.9355 1711.8565 1711.8165 0.0400 0 K.LFGGFNSSDTVTSPQR.A**  ([Ions score 94](http://df3/mascot/cgi/peptide_view.pl?file=../data/20121119/F002569.dat&query=2439&hit=1&index=gi%7c00000001&px=1))

**63 - 78 856.9388 1711.8631 1711.8165 0.0466 0 K.LFGGFNSSDTVTSPQR.A**  ([Ions score 95](http://df3/mascot/cgi/peptide_view.pl?file=../data/20121119/F002569.dat&query=2440&hit=1&index=gi%7c00000001&px=1))

**63 - 78 856.9390 1711.8634 1711.8165 0.0469 0 K.LFGGFNSSDTVTSPQR.A**  ([Ions score 119](http://df3/mascot/cgi/peptide_view.pl?file=../data/20121119/F002569.dat&query=2441&hit=1&index=gi%7c00000001&px=1))

**63 - 78 856.9415 1711.8684 1711.8165 0.0519 0 K.LFGGFNSSDTVTSPQR.A**  ([Ions score 117](http://df3/mascot/cgi/peptide_view.pl?file=../data/20121119/F002569.dat&query=2442&hit=1&index=gi%7c00000001&px=1))

**63 - 78 856.9440 1711.8734 1711.8165 0.0569 0 K.LFGGFNSSDTVTSPQR.A**  ([Ions score 92](http://df3/mascot/cgi/peptide_view.pl?file=../data/20121119/F002569.dat&query=2443&hit=1&index=gi%7c00000001&px=1))

**63 - 78 856.9460 1711.8774 1711.8165 0.0609 0 K.LFGGFNSSDTVTSPQR.A**  ([Ions score 97](http://df3/mascot/cgi/peptide_view.pl?file=../data/20121119/F002569.dat&query=2444&hit=1&index=gi%7c00000001&px=1))

**63 - 78 856.9506 1711.8866 1711.8165 0.0701 0 K.LFGGFNSSDTVTSPQR.A**  ([Ions score 108](http://df3/mascot/cgi/peptide_view.pl?file=../data/20121119/F002569.dat&query=2445&hit=1&index=gi%7c00000001&px=1))

**63 - 78 856.9534 1711.8922 1711.8165 0.0757 0 K.LFGGFNSSDTVTSPQR.A**  ([Ions score 96](http://df3/mascot/cgi/peptide_view.pl?file=../data/20121119/F002569.dat&query=2446&hit=1&index=gi%7c00000001&px=1))

**63 - 78 856.9620 1711.9095 1711.8165 0.0930 0 K.LFGGFNSSDTVTSPQR.A**  ([Ions score 112](http://df3/mascot/cgi/peptide_view.pl?file=../data/20121119/F002569.dat&query=2447&hit=1&index=gi%7c00000001&px=1))

**63 - 78 856.9651 1711.9156 1711.8165 0.0991 0 K.LFGGFNSSDTVTSPQR.A**  ([Ions score 107](http://df3/mascot/cgi/peptide_view.pl?file=../data/20121119/F002569.dat&query=2448&hit=1&index=gi%7c00000001&px=1))

**63 - 78 856.9666 1711.9186 1711.8165 0.1020 0 K.LFGGFNSSDTVTSPQR.A**  ([Ions score 108](http://df3/mascot/cgi/peptide_view.pl?file=../data/20121119/F002569.dat&query=2449&hit=1&index=gi%7c00000001&px=1))

**63 - 78 856.9690 1711.9234 1711.8165 0.1069 0 K.LFGGFNSSDTVTSPQR.A**  ([Ions score 101](http://df3/mascot/cgi/peptide_view.pl?file=../data/20121119/F002569.dat&query=2450&hit=1&index=gi%7c00000001&px=1))

**63 - 78 856.9791 1711.9437 1711.8165 0.1272 0 K.LFGGFNSSDTVTSPQR.A**  ([Ions score 105](http://df3/mascot/cgi/peptide_view.pl?file=../data/20121119/F002569.dat&query=2451&hit=1&index=gi%7c00000001&px=1))

**63 - 78 857.0081 1712.0016 1711.8165 0.1850 0 K.LFGGFNSSDTVTSPQR.A**  ([Ions score 105](http://df3/mascot/cgi/peptide_view.pl?file=../data/20121119/F002569.dat&query=2452&hit=1&index=gi%7c00000001&px=1))

**63 - 78 857.0129 1712.0113 1711.8165 0.1948 0 K.LFGGFNSSDTVTSPQR.A**  ([Ions score 100](http://df3/mascot/cgi/peptide_view.pl?file=../data/20121119/F002569.dat&query=2453&hit=1&index=gi%7c00000001&px=1))

**63 - 78 857.0180 1712.0215 1711.8165 0.2049 0 K.LFGGFNSSDTVTSPQR.A**  ([Ions score 111](http://df3/mascot/cgi/peptide_view.pl?file=../data/20121119/F002569.dat&query=2454&hit=1&index=gi%7c00000001&px=1))

**63 - 78 857.0216 1712.0287 1711.8165 0.2121 0 K.LFGGFNSSDTVTSPQR.A**  ([Ions score 106](http://df3/mascot/cgi/peptide_view.pl?file=../data/20121119/F002569.dat&query=2455&hit=1&index=gi%7c00000001&px=1))

**63 - 78 857.0267 1712.0389 1711.8165 0.2224 0 K.LFGGFNSSDTVTSPQR.A**  ([Ions score 100](http://df3/mascot/cgi/peptide_view.pl?file=../data/20121119/F002569.dat&query=2456&hit=1&index=gi%7c00000001&px=1))

**63 - 78 857.0487 1712.0829 1711.8165 0.2663 0 K.LFGGFNSSDTVTSPQR.A**  ([Ions score 117](http://df3/mascot/cgi/peptide_view.pl?file=../data/20121119/F002569.dat&query=2457&hit=1&index=gi%7c00000001&px=1))

**63 - 78 857.0681 1712.1217 1711.8165 0.3052 0 K.LFGGFNSSDTVTSPQR.A**  ([Ions score 112](http://df3/mascot/cgi/peptide_view.pl?file=../data/20121119/F002569.dat&query=2458&hit=1&index=gi%7c00000001&px=1))

**63 - 78 857.0765 1712.1385 1711.8165 0.3220 0 K.LFGGFNSSDTVTSPQR.A**  ([Ions score 72](http://df3/mascot/cgi/peptide_view.pl?file=../data/20121119/F002569.dat&query=2459&hit=1&index=gi%7c00000001&px=1))

**63 - 78 857.1361 1712.2577 1711.8165 0.4412 0 K.LFGGFNSSDTVTSPQR.A**  ([Ions score 102](http://df3/mascot/cgi/peptide_view.pl?file=../data/20121119/F002569.dat&query=2461&hit=1&index=gi%7c00000001&px=1))

**63 - 78 857.1476 1712.2806 1711.8165 0.4641 0 K.LFGGFNSSDTVTSPQR.A**  ([Ions score 115](http://df3/mascot/cgi/peptide_view.pl?file=../data/20121119/F002569.dat&query=2462&hit=1&index=gi%7c00000001&px=1))

**63 - 78 857.1680 1712.3214 1711.8165 0.5049 0 K.LFGGFNSSDTVTSPQR.A**  ([Ions score 103](http://df3/mascot/cgi/peptide_view.pl?file=../data/20121119/F002569.dat&query=2463&hit=1&index=gi%7c00000001&px=1))

**63 - 78 857.1720 1712.3294 1711.8165 0.5129 0 K.LFGGFNSSDTVTSPQR.A**  ([Ions score 103](http://df3/mascot/cgi/peptide_view.pl?file=../data/20121119/F002569.dat&query=2464&hit=1&index=gi%7c00000001&px=1))

**63 - 78 857.1926 1712.3707 1711.8165 0.5542 0 K.LFGGFNSSDTVTSPQR.A**  ([Ions score 83](http://df3/mascot/cgi/peptide_view.pl?file=../data/20121119/F002569.dat&query=2465&hit=1&index=gi%7c00000001&px=1))

**63 - 78 857.2124 1712.4103 1711.8165 0.5937 0 K.LFGGFNSSDTVTSPQR.A**  ([Ions score 119](http://df3/mascot/cgi/peptide_view.pl?file=../data/20121119/F002569.dat&query=2466&hit=1&index=gi%7c00000001&px=1))

**63 - 78 857.3098 1712.6050 1711.8165 0.7884 0 K.LFGGFNSSDTVTSPQR.A**  ([Ions score 113](http://df3/mascot/cgi/peptide_view.pl?file=../data/20121119/F002569.dat&query=2467&hit=1&index=gi%7c00000001&px=1))

**63 - 78 857.3318 1712.6490 1711.8165 0.8325 0 K.LFGGFNSSDTVTSPQR.A**  ([Ions score 117](http://df3/mascot/cgi/peptide_view.pl?file=../data/20121119/F002569.dat&query=2468&hit=1&index=gi%7c00000001&px=1))

**63 - 78 857.3425 1712.6705 1711.8165 0.8540 0 K.LFGGFNSSDTVTSPQR.A**  ([Ions score 111](http://df3/mascot/cgi/peptide_view.pl?file=../data/20121119/F002569.dat&query=2469&hit=1&index=gi%7c00000001&px=1))

**63 - 78 857.3490 1712.6834 1711.8165 0.8669 0 K.LFGGFNSSDTVTSPQR.A**  ([Ions score 108](http://df3/mascot/cgi/peptide_view.pl?file=../data/20121119/F002569.dat&query=2470&hit=1&index=gi%7c00000001&px=1))

**63 - 78 857.3517 1712.6888 1711.8165 0.8723 0 K.LFGGFNSSDTVTSPQR.A**  ([Ions score 120](http://df3/mascot/cgi/peptide_view.pl?file=../data/20121119/F002569.dat&query=2471&hit=1&index=gi%7c00000001&px=1))

**63 - 78 857.3555 1712.6964 1711.8165 0.8799 0 K.LFGGFNSSDTVTSPQR.A**  ([Ions score 102](http://df3/mascot/cgi/peptide_view.pl?file=../data/20121119/F002569.dat&query=2472&hit=1&index=gi%7c00000001&px=1))

**63 - 78 857.3625 1712.7105 1711.8165 0.8940 0 K.LFGGFNSSDTVTSPQR.A**  ([Ions score 114](http://df3/mascot/cgi/peptide_view.pl?file=../data/20121119/F002569.dat&query=2473&hit=1&index=gi%7c00000001&px=1))

**63 - 78 857.3640 1712.7134 1711.8165 0.8968 0 K.LFGGFNSSDTVTSPQR.A**  ([Ions score 110](http://df3/mascot/cgi/peptide_view.pl?file=../data/20121119/F002569.dat&query=2474&hit=1&index=gi%7c00000001&px=1))

**63 - 78 857.3681 1712.7217 1711.8165 0.9051 0 K.LFGGFNSSDTVTSPQR.A**  ([Ions score 103](http://df3/mascot/cgi/peptide_view.pl?file=../data/20121119/F002569.dat&query=2475&hit=1&index=gi%7c00000001&px=1))

**63 - 78 857.3715 1712.7284 1711.8165 0.9119 0 K.LFGGFNSSDTVTSPQR.A**  ([Ions score 108](http://df3/mascot/cgi/peptide_view.pl?file=../data/20121119/F002569.dat&query=2476&hit=1&index=gi%7c00000001&px=1))

**63 - 78 857.3774 1712.7402 1711.8165 0.9237 0 K.LFGGFNSSDTVTSPQR.A**  ([Ions score 108](http://df3/mascot/cgi/peptide_view.pl?file=../data/20121119/F002569.dat&query=2477&hit=1&index=gi%7c00000001&px=1))

**63 - 78 857.3795 1712.7444 1711.8165 0.9278 0 K.LFGGFNSSDTVTSPQR.A**  ([Ions score 105](http://df3/mascot/cgi/peptide_view.pl?file=../data/20121119/F002569.dat&query=2478&hit=1&index=gi%7c00000001&px=1))

**63 - 78 857.3810 1712.7475 1711.8165 0.9310 0 K.LFGGFNSSDTVTSPQR.A**  ([Ions score 95](http://df3/mascot/cgi/peptide_view.pl?file=../data/20121119/F002569.dat&query=2479&hit=1&index=gi%7c00000001&px=1))

**63 - 78 857.3817 1712.7488 1711.8165 0.9322 0 K.LFGGFNSSDTVTSPQR.A**  ([Ions score 119](http://df3/mascot/cgi/peptide_view.pl?file=../data/20121119/F002569.dat&query=2480&hit=1&index=gi%7c00000001&px=1))

**63 - 78 857.3926 1712.7706 1711.8165 0.9541 0 K.LFGGFNSSDTVTSPQR.A**  ([Ions score 118](http://df3/mascot/cgi/peptide_view.pl?file=../data/20121119/F002569.dat&query=2481&hit=1&index=gi%7c00000001&px=1))

**63 - 78 571.9382 1712.7929 1711.8165 0.9764 0 K.LFGGFNSSDTVTSPQR.A**  ([Ions score 67](http://df3/mascot/cgi/peptide_view.pl?file=../data/20121119/F002569.dat&query=998&hit=1&index=gi%7c00000001&px=1))

**63 - 78 857.4199 1712.8253 1711.8165 1.0088 0 K.LFGGFNSSDTVTSPQR.A**  ([Ions score 114](http://df3/mascot/cgi/peptide_view.pl?file=../data/20121119/F002569.dat&query=2482&hit=1&index=gi%7c00000001&px=1))

**63 - 78 857.4218 1712.8291 1711.8165 1.0126 0 K.LFGGFNSSDTVTSPQR.A**  ([Ions score 93](http://df3/mascot/cgi/peptide_view.pl?file=../data/20121119/F002569.dat&query=2483&hit=1&index=gi%7c00000001&px=1))

**63 - 78 857.4234 1712.8322 1711.8165 1.0157 0 K.LFGGFNSSDTVTSPQR.A**  ([Ions score 120](http://df3/mascot/cgi/peptide_view.pl?file=../data/20121119/F002569.dat&query=2484&hit=1&index=gi%7c00000001&px=1))

**63 - 78 857.4241 1712.8337 1711.8165 1.0172 0 K.LFGGFNSSDTVTSPQR.A**  ([Ions score 100](http://df3/mascot/cgi/peptide_view.pl?file=../data/20121119/F002569.dat&query=2485&hit=1&index=gi%7c00000001&px=1))

**63 - 78 857.4253 1712.8360 1711.8165 1.0195 0 K.LFGGFNSSDTVTSPQR.A**  ([Ions score 121](http://df3/mascot/cgi/peptide_view.pl?file=../data/20121119/F002569.dat&query=2486&hit=1&index=gi%7c00000001&px=1))

**63 - 78 857.4310 1712.8475 1711.8165 1.0310 0 K.LFGGFNSSDTVTSPQR.A**  ([Ions score 96](http://df3/mascot/cgi/peptide_view.pl?file=../data/20121119/F002569.dat&query=2487&hit=1&index=gi%7c00000001&px=1))

**63 - 78 857.4374 1712.8602 1711.8165 1.0437 0 K.LFGGFNSSDTVTSPQR.A**  ([Ions score 106](http://df3/mascot/cgi/peptide_view.pl?file=../data/20121119/F002569.dat&query=2488&hit=1&index=gi%7c00000001&px=1))

**63 - 78 857.4493 1712.8840 1711.8165 1.0675 0 K.LFGGFNSSDTVTSPQR.A**  ([Ions score 88](http://df3/mascot/cgi/peptide_view.pl?file=../data/20121119/F002569.dat&query=2489&hit=1&index=gi%7c00000001&px=1))

**63 - 78 857.4586 1712.9026 1711.8165 1.0860 0 K.LFGGFNSSDTVTSPQR.A**  ([Ions score 101](http://df3/mascot/cgi/peptide_view.pl?file=../data/20121119/F002569.dat&query=2490&hit=1&index=gi%7c00000001&px=1))

**63 - 78 857.4638 1712.9131 1711.8165 1.0965 0 K.LFGGFNSSDTVTSPQR.A**  ([Ions score 90](http://df3/mascot/cgi/peptide_view.pl?file=../data/20121119/F002569.dat&query=2491&hit=1&index=gi%7c00000001&px=1))

**63 - 78 857.4678 1712.9210 1711.8165 1.1045 0 K.LFGGFNSSDTVTSPQR.A**  ([Ions score 103](http://df3/mascot/cgi/peptide_view.pl?file=../data/20121119/F002569.dat&query=2492&hit=1&index=gi%7c00000001&px=1))

**63 - 78 857.5132 1713.0118 1711.8165 1.1953 0 K.LFGGFNSSDTVTSPQR.A**  ([Ions score 140](http://df3/mascot/cgi/peptide_view.pl?file=../data/20121119/F002569.dat&query=2493&hit=1&index=gi%7c00000001&px=1))

**63 - 78 857.5231 1713.0316 1711.8165 1.2151 0 K.LFGGFNSSDTVTSPQR.A**  ([Ions score 91](http://df3/mascot/cgi/peptide_view.pl?file=../data/20121119/F002569.dat&query=2494&hit=1&index=gi%7c00000001&px=1))

**63 - 78 857.5325 1713.0504 1711.8165 1.2339 0 K.LFGGFNSSDTVTSPQR.A**  ([Ions score 90](http://df3/mascot/cgi/peptide_view.pl?file=../data/20121119/F002569.dat&query=2495&hit=1&index=gi%7c00000001&px=1))

**63 - 78 857.5608 1713.1070 1711.8165 1.2905 0 K.LFGGFNSSDTVTSPQR.A**  ([Ions score 107](http://df3/mascot/cgi/peptide_view.pl?file=../data/20121119/F002569.dat&query=2496&hit=1&index=gi%7c00000001&px=1))

**63 - 78 857.5682 1713.1218 1711.8165 1.3053 0 K.LFGGFNSSDTVTSPQR.A**  ([Ions score 85](http://df3/mascot/cgi/peptide_view.pl?file=../data/20121119/F002569.dat&query=2497&hit=1&index=gi%7c00000001&px=1))

**63 - 78 857.5778 1713.1410 1711.8165 1.3245 0 K.LFGGFNSSDTVTSPQR.A**  ([Ions score 86](http://df3/mascot/cgi/peptide_view.pl?file=../data/20121119/F002569.dat&query=2498&hit=1&index=gi%7c00000001&px=1))

**63 - 78 572.0544 1713.1415 1711.8165 1.3250 0 K.LFGGFNSSDTVTSPQR.A**  ([Ions score 63](http://df3/mascot/cgi/peptide_view.pl?file=../data/20121119/F002569.dat&query=999&hit=1&index=gi%7c00000001&px=1))

**63 - 78 572.0605 1713.1596 1711.8165 1.3431 0 K.LFGGFNSSDTVTSPQR.A**  ([Ions score 72](http://df3/mascot/cgi/peptide_view.pl?file=../data/20121119/F002569.dat&query=1000&hit=1&index=gi%7c00000001&px=1))

**63 - 78 572.0786 1713.2140 1711.8165 1.3975 0 K.LFGGFNSSDTVTSPQR.A**  ([Ions score 41](http://df3/mascot/cgi/peptide_view.pl?file=../data/20121119/F002569.dat&query=1001&hit=1&index=gi%7c00000001&px=1))

**63 - 78 572.1398 1713.3975 1711.8165 1.5810 0 K.LFGGFNSSDTVTSPQR.A**  ([Ions score 61](http://df3/mascot/cgi/peptide_view.pl?file=../data/20121119/F002569.dat&query=1002&hit=1&index=gi%7c00000001&px=1))

**63 - 78 857.7314 1713.4483 1711.8165 1.6318 0 K.LFGGFNSSDTVTSPQR.A**  ([Ions score 103](http://df3/mascot/cgi/peptide_view.pl?file=../data/20121119/F002569.dat&query=2499&hit=1&index=gi%7c00000001&px=1))

**63 - 78 857.8226 1713.6307 1711.8165 1.8142 0 K.LFGGFNSSDTVTSPQR.A**  ([Ions score 94](http://df3/mascot/cgi/peptide_view.pl?file=../data/20121119/F002569.dat&query=2500&hit=1&index=gi%7c00000001&px=1))

**63 - 78 572.2260 1713.6560 1711.8165 1.8395 0 K.LFGGFNSSDTVTSPQR.A**  ([Ions score 54](http://df3/mascot/cgi/peptide_view.pl?file=../data/20121119/F002569.dat&query=1005&hit=1&index=gi%7c00000001&px=1))

**63 - 78 857.8795 1713.7445 1711.8165 1.9280 0 K.LFGGFNSSDTVTSPQR.A**  ([Ions score 95](http://df3/mascot/cgi/peptide_view.pl?file=../data/20121119/F002569.dat&query=2501&hit=1&index=gi%7c00000001&px=1))

**63 - 78 857.9128 1713.8110 1711.8165 1.9945 0 K.LFGGFNSSDTVTSPQR.A**  ([Ions score 110](http://df3/mascot/cgi/peptide_view.pl?file=../data/20121119/F002569.dat&query=2502&hit=1&index=gi%7c00000001&px=1))

**63 - 78 857.1071 1712.1997 1712.8005 -0.6008 0 K.LFGGFNSSDTVTSPQR.A**  Deamidation (NQ) ([Ions score 134](http://df3/mascot/cgi/peptide_view.pl?file=../data/20121119/F002569.dat&query=2460&hit=1&index=gi%7c00000001&px=1))

**63 - 78 857.9232 1713.8319 1712.8005 1.0314 0 K.LFGGFNSSDTVTSPQR.A**  Deamidation (NQ) ([Ions score 74](http://df3/mascot/cgi/peptide_view.pl?file=../data/20121119/F002569.dat&query=2503&hit=1&index=gi%7c00000001&px=1))

**63 - 78 857.9396 1713.8646 1712.8005 1.0641 0 K.LFGGFNSSDTVTSPQR.A**  Deamidation (NQ) ([Ions score 87](http://df3/mascot/cgi/peptide_view.pl?file=../data/20121119/F002569.dat&query=2504&hit=1&index=gi%7c00000001&px=1))

**63 - 78 857.9680 1713.9214 1712.8005 1.1208 0 K.LFGGFNSSDTVTSPQR.A**  Deamidation (NQ) ([Ions score 83](http://df3/mascot/cgi/peptide_view.pl?file=../data/20121119/F002569.dat&query=2505&hit=1&index=gi%7c00000001&px=1))

**63 - 78 857.9929 1713.9713 1712.8005 1.1708 0 K.LFGGFNSSDTVTSPQR.A**  Deamidation (NQ) ([Ions score 96](http://df3/mascot/cgi/peptide_view.pl?file=../data/20121119/F002569.dat&query=2506&hit=1&index=gi%7c00000001&px=1))

**63 - 78 572.3337 1713.9794 1712.8005 1.1789 0 K.LFGGFNSSDTVTSPQR.A**  Deamidation (NQ) ([Ions score 76](http://df3/mascot/cgi/peptide_view.pl?file=../data/20121119/F002569.dat&query=1007&hit=1&index=gi%7c00000001&px=1))

**63 - 78 858.0063 1713.9980 1712.8005 1.1975 0 K.LFGGFNSSDTVTSPQR.A**  Deamidation (NQ) ([Ions score 90](http://df3/mascot/cgi/peptide_view.pl?file=../data/20121119/F002569.dat&query=2507&hit=1&index=gi%7c00000001&px=1))

**63 - 78 858.0122 1714.0099 1712.8005 1.2093 0 K.LFGGFNSSDTVTSPQR.A**  Deamidation (NQ) ([Ions score 120](http://df3/mascot/cgi/peptide_view.pl?file=../data/20121119/F002569.dat&query=2508&hit=1&index=gi%7c00000001&px=1))

**63 - 78 858.0233 1714.0321 1712.8005 1.2316 0 K.LFGGFNSSDTVTSPQR.A**  Deamidation (NQ) ([Ions score 72](http://df3/mascot/cgi/peptide_view.pl?file=../data/20121119/F002569.dat&query=2509&hit=1&index=gi%7c00000001&px=1))

**63 - 78 858.0728 1714.1311 1712.8005 1.3306 0 K.LFGGFNSSDTVTSPQR.A**  Deamidation (NQ) ([Ions score 84](http://df3/mascot/cgi/peptide_view.pl?file=../data/20121119/F002569.dat&query=2510&hit=1&index=gi%7c00000001&px=1))

**63 - 78 858.3502 1714.6859 1712.8005 1.8854 0 K.LFGGFNSSDTVTSPQR.A**  Deamidation (NQ) ([Ions score 102](http://df3/mascot/cgi/peptide_view.pl?file=../data/20121119/F002569.dat&query=2511&hit=1&index=gi%7c00000001&px=1))

**63 - 78 858.4548 1714.8951 1713.7845 1.1106 0 K.LFGGFNSSDTVTSPQR.A**  2 Deamidation (NQ) ([Ions score 82](http://df3/mascot/cgi/peptide_view.pl?file=../data/20121119/F002569.dat&query=2512&hit=1&index=gi%7c00000001&px=1))

**63 - 78 858.4617 1714.9088 1713.7845 1.1243 0 K.LFGGFNSSDTVTSPQR.A**  2 Deamidation (NQ) ([Ions score 88](http://df3/mascot/cgi/peptide_view.pl?file=../data/20121119/F002569.dat&query=2513&hit=1&index=gi%7c00000001&px=1))

**63 - 78 858.5239 1715.0333 1713.7845 1.2488 0 K.LFGGFNSSDTVTSPQR.A**  2 Deamidation (NQ) ([Ions score 93](http://df3/mascot/cgi/peptide_view.pl?file=../data/20121119/F002569.dat&query=2515&hit=1&index=gi%7c00000001&px=1))

**63 - 78 858.6077 1715.2008 1713.7845 1.4162 0 K.LFGGFNSSDTVTSPQR.A**  2 Deamidation (NQ) ([Ions score 98](http://df3/mascot/cgi/peptide_view.pl?file=../data/20121119/F002569.dat&query=2516&hit=1&index=gi%7c00000001&px=1))

**63 - 78 858.6101 1715.2057 1713.7845 1.4211 0 K.LFGGFNSSDTVTSPQR.A**  2 Deamidation (NQ) ([Ions score 95](http://df3/mascot/cgi/peptide_view.pl?file=../data/20121119/F002569.dat&query=2517&hit=1&index=gi%7c00000001&px=1))

**79 - 98 696.4525 2086.3357 2086.0370 0.2987 0 R.AGPLAGGVTTFVALYDYESR.T**  ([Ions score 28](http://df3/mascot/cgi/peptide_view.pl?file=../data/20121119/F002569.dat&query=1551&hit=1&index=gi%7c00000001&px=1))

**79 - 98 696.7629 2087.2670 2086.0370 1.2300 0 R.AGPLAGGVTTFVALYDYESR.T**  ([Ions score 26](http://df3/mascot/cgi/peptide_view.pl?file=../data/20121119/F002569.dat&query=1554&hit=1&index=gi%7c00000001&px=1))

**160 - 172 770.9617 1539.9088 1539.8005 0.1083 1 R.ESERLLLNAENPR.G**  ([Ions score 30](http://df3/mascot/cgi/peptide_view.pl?file=../data/20121119/F002569.dat&query=2140&hit=1&index=gi%7c00000001&px=1))

**160 - 172 514.4980 1540.4723 1539.8005 0.6719 1 R.ESERLLLNAENPR.G**  ([Ions score 49](http://df3/mascot/cgi/peptide_view.pl?file=../data/20121119/F002569.dat&query=763&hit=1&index=gi%7c00000001&px=1))

**160 - 172 514.6069 1540.7990 1539.8005 0.9985 1 R.ESERLLLNAENPR.G**  ([Ions score 51](http://df3/mascot/cgi/peptide_view.pl?file=../data/20121119/F002569.dat&query=765&hit=1&index=gi%7c00000001&px=1))

**160 - 172 771.5399 1541.0653 1539.8005 1.2648 1 R.ESERLLLNAENPR.G**  ([Ions score 51](http://df3/mascot/cgi/peptide_view.pl?file=../data/20121119/F002569.dat&query=2144&hit=1&index=gi%7c00000001&px=1))

**164 - 172 519.9821 1037.9496 1038.5822 -0.6326 0 R.LLLNAENPR.G**  ([Ions score 33](http://df3/mascot/cgi/peptide_view.pl?file=../data/20121119/F002569.dat&query=803&hit=1&index=gi%7c00000001&px=1))

**164 - 172 519.9973 1037.9801 1038.5822 -0.6021 0 R.LLLNAENPR.G**  ([Ions score 23](http://df3/mascot/cgi/peptide_view.pl?file=../data/20121119/F002569.dat&query=804&hit=1&index=gi%7c00000001&px=1))

**164 - 172 520.0958 1038.1771 1038.5822 -0.4051 0 R.LLLNAENPR.G**  ([Ions score 63](http://df3/mascot/cgi/peptide_view.pl?file=../data/20121119/F002569.dat&query=805&hit=1&index=gi%7c00000001&px=1))

**164 - 172 520.1521 1038.2896 1038.5822 -0.2925 0 R.LLLNAENPR.G**  ([Ions score 62](http://df3/mascot/cgi/peptide_view.pl?file=../data/20121119/F002569.dat&query=807&hit=1&index=gi%7c00000001&px=1))

**164 - 172 520.2513 1038.4881 1038.5822 -0.0940 0 R.LLLNAENPR.G**  ([Ions score 69](http://df3/mascot/cgi/peptide_view.pl?file=../data/20121119/F002569.dat&query=808&hit=1&index=gi%7c00000001&px=1))

**164 - 172 520.2668 1038.5191 1038.5822 -0.0630 0 R.LLLNAENPR.G**  ([Ions score 70](http://df3/mascot/cgi/peptide_view.pl?file=../data/20121119/F002569.dat&query=809&hit=1&index=gi%7c00000001&px=1))

**164 - 172 520.3851 1038.7557 1038.5822 0.1736 0 R.LLLNAENPR.G**  ([Ions score 44](http://df3/mascot/cgi/peptide_view.pl?file=../data/20121119/F002569.dat&query=810&hit=1&index=gi%7c00000001&px=1))

**164 - 172 520.4672 1038.9198 1038.5822 0.3376 0 R.LLLNAENPR.G**  ([Ions score 41](http://df3/mascot/cgi/peptide_view.pl?file=../data/20121119/F002569.dat&query=811&hit=1&index=gi%7c00000001&px=1))

**164 - 172 520.4690 1038.9234 1038.5822 0.3413 0 R.LLLNAENPR.G**  ([Ions score 57](http://df3/mascot/cgi/peptide_view.pl?file=../data/20121119/F002569.dat&query=812&hit=1&index=gi%7c00000001&px=1))

**164 - 172 520.4906 1038.9667 1038.5822 0.3845 0 R.LLLNAENPR.G**  ([Ions score 13](http://df3/mascot/cgi/peptide_view.pl?file=../data/20121119/F002569.dat&query=813&hit=1&index=gi%7c00000001&px=1))

**164 - 172 520.5491 1039.0837 1038.5822 0.5016 0 R.LLLNAENPR.G**  ([Ions score 51](http://df3/mascot/cgi/peptide_view.pl?file=../data/20121119/F002569.dat&query=814&hit=1&index=gi%7c00000001&px=1))

**164 - 172 520.6259 1039.2372 1038.5822 0.6550 0 R.LLLNAENPR.G**  ([Ions score 65](http://df3/mascot/cgi/peptide_view.pl?file=../data/20121119/F002569.dat&query=815&hit=1&index=gi%7c00000001&px=1))

**164 - 172 520.7757 1039.5368 1038.5822 0.9547 0 R.LLLNAENPR.G**  ([Ions score 31](http://df3/mascot/cgi/peptide_view.pl?file=../data/20121119/F002569.dat&query=820&hit=1&index=gi%7c00000001&px=1))

**164 - 172 520.8201 1039.6257 1038.5822 1.0436 0 R.LLLNAENPR.G**  ([Ions score 39](http://df3/mascot/cgi/peptide_view.pl?file=../data/20121119/F002569.dat&query=821&hit=1&index=gi%7c00000001&px=1))

**164 - 172 520.8352 1039.6559 1038.5822 1.0737 0 R.LLLNAENPR.G**  ([Ions score 17](http://df3/mascot/cgi/peptide_view.pl?file=../data/20121119/F002569.dat&query=822&hit=1&index=gi%7c00000001&px=1))

**164 - 172 520.8918 1039.7691 1038.5822 1.1870 0 R.LLLNAENPR.G**  ([Ions score 27](http://df3/mascot/cgi/peptide_view.pl?file=../data/20121119/F002569.dat&query=824&hit=1&index=gi%7c00000001&px=1))

**164 - 172 520.9993 1039.9840 1038.5822 1.4018 0 R.LLLNAENPR.G**  ([Ions score 21](http://df3/mascot/cgi/peptide_view.pl?file=../data/20121119/F002569.dat&query=828&hit=1&index=gi%7c00000001&px=1))

**164 - 172 521.1649 1040.3152 1038.5822 1.7330 0 R.LLLNAENPR.G**  ([Ions score 62](http://df3/mascot/cgi/peptide_view.pl?file=../data/20121119/F002569.dat&query=829&hit=1&index=gi%7c00000001&px=1))

**164 - 172 521.2802 1040.5458 1039.5662 0.9796 0 R.LLLNAENPR.G**  Deamidation (NQ) ([Ions score 14](http://df3/mascot/cgi/peptide_view.pl?file=../data/20121119/F002569.dat&query=830&hit=1&index=gi%7c00000001&px=1))

**164 - 172 521.4045 1040.7944 1039.5662 1.2282 0 R.LLLNAENPR.G**  Deamidation (NQ) ([Ions score 61](http://df3/mascot/cgi/peptide_view.pl?file=../data/20121119/F002569.dat&query=831&hit=1&index=gi%7c00000001&px=1))

**164 - 172 521.6654 1041.3163 1039.5662 1.7501 0 R.LLLNAENPR.G**  Deamidation (NQ) ([Ions score 10](http://df3/mascot/cgi/peptide_view.pl?file=../data/20121119/F002569.dat&query=834&hit=1&index=gi%7c00000001&px=1))

**164 - 178 572.1540 1713.4401 1711.9733 1.4669 1 R.LLLNAENPRGTFLVR.E**  ([Ions score 36](http://df3/mascot/cgi/peptide_view.pl?file=../data/20121119/F002569.dat&query=1003&hit=1&index=gi%7c00000001&px=1))

**164 - 178 572.1555 1713.4445 1711.9733 1.4713 1 R.LLLNAENPRGTFLVR.E**  ([Ions score 37](http://df3/mascot/cgi/peptide_view.pl?file=../data/20121119/F002569.dat&query=1004&hit=1&index=gi%7c00000001&px=1))

**179 - 198 741.0604 2220.1593 2220.9844 -0.8252 1 R.ESETTKGAYCLSVSDFDNAK.G**  Carbamidomethyl (C) ([Ions score 15](http://df3/mascot/cgi/peptide_view.pl?file=../data/20121119/F002569.dat&query=1870&hit=1&index=gi%7c00000001&px=1))

**179 - 198 741.3905 2221.1497 2220.9844 0.1652 1 R.ESETTKGAYCLSVSDFDNAK.G**  Carbamidomethyl (C) ([Ions score 61](http://df3/mascot/cgi/peptide_view.pl?file=../data/20121119/F002569.dat&query=1877&hit=1&index=gi%7c00000001&px=1))

**179 - 198 741.4304 2221.2694 2220.9844 0.2850 1 R.ESETTKGAYCLSVSDFDNAK.G**  Carbamidomethyl (C) ([Ions score 12](http://df3/mascot/cgi/peptide_view.pl?file=../data/20121119/F002569.dat&query=1879&hit=1&index=gi%7c00000001&px=1))

**179 - 198 741.4424 2221.3053 2220.9844 0.3209 1 R.ESETTKGAYCLSVSDFDNAK.G**  Carbamidomethyl (C) ([Ions score 13](http://df3/mascot/cgi/peptide_view.pl?file=../data/20121119/F002569.dat&query=1881&hit=1&index=gi%7c00000001&px=1))

**179 - 198 741.5100 2221.5082 2220.9844 0.5238 1 R.ESETTKGAYCLSVSDFDNAK.G**  Carbamidomethyl (C) ([Ions score 43](http://df3/mascot/cgi/peptide_view.pl?file=../data/20121119/F002569.dat&query=1885&hit=1&index=gi%7c00000001&px=1))

**179 - 198 741.6075 2221.8008 2220.9844 0.8164 1 R.ESETTKGAYCLSVSDFDNAK.G**  Carbamidomethyl (C) ([Ions score 12](http://df3/mascot/cgi/peptide_view.pl?file=../data/20121119/F002569.dat&query=1887&hit=1&index=gi%7c00000001&px=1))

**179 - 198 741.7228 2222.1467 2220.9844 1.1623 1 R.ESETTKGAYCLSVSDFDNAK.G**  Carbamidomethyl (C) ([Ions score 60](http://df3/mascot/cgi/peptide_view.pl?file=../data/20121119/F002569.dat&query=1889&hit=1&index=gi%7c00000001&px=1))

**179 - 198 741.7636 2222.2688 2220.9844 1.2844 1 R.ESETTKGAYCLSVSDFDNAK.G**  Carbamidomethyl (C) ([Ions score 34](http://df3/mascot/cgi/peptide_view.pl?file=../data/20121119/F002569.dat&query=1890&hit=1&index=gi%7c00000001&px=1))

**179 - 198 741.8004 2222.3794 2220.9844 1.3950 1 R.ESETTKGAYCLSVSDFDNAK.G**  Carbamidomethyl (C) ([Ions score 65](http://df3/mascot/cgi/peptide_view.pl?file=../data/20121119/F002569.dat&query=1892&hit=1&index=gi%7c00000001&px=1))

**185 - 198 745.9460 1489.8775 1488.6554 1.2221 0 K.GAYCLSVSDFDNAK.G**  ([Ions score 10](http://df3/mascot/cgi/peptide_view.pl?file=../data/20121119/F002569.dat&query=1925&hit=1&index=gi%7c00000001&px=1))

**185 - 198 773.8282 1545.6419 1545.6769 -0.0350 0 K.GAYCLSVSDFDNAK.G**  Carbamidomethyl (C) ([Ions score 102](http://df3/mascot/cgi/peptide_view.pl?file=../data/20121119/F002569.dat&query=2149&hit=1&index=gi%7c00000001&px=1))

**185 - 198 773.9008 1545.7871 1545.6769 0.1102 0 K.GAYCLSVSDFDNAK.G**  Carbamidomethyl (C) ([Ions score 106](http://df3/mascot/cgi/peptide_view.pl?file=../data/20121119/F002569.dat&query=2151&hit=1&index=gi%7c00000001&px=1))

**185 - 198 774.4362 1546.8578 1545.6769 1.1809 0 K.GAYCLSVSDFDNAK.G**  Carbamidomethyl (C) ([Ions score 100](http://df3/mascot/cgi/peptide_view.pl?file=../data/20121119/F002569.dat&query=2152&hit=1&index=gi%7c00000001&px=1))

**185 - 198 774.9086 1547.8026 1546.6609 1.1417 0 K.GAYCLSVSDFDNAK.G**  Carbamidomethyl (C); Deamidation (NQ) ([Ions score 74](http://df3/mascot/cgi/peptide_view.pl?file=../data/20121119/F002569.dat&query=2153&hit=1&index=gi%7c00000001&px=1))

**185 - 198 774.9593 1547.9040 1546.6609 1.2431 0 K.GAYCLSVSDFDNAK.G**  Carbamidomethyl (C); Deamidation (NQ) ([Ions score 13](http://df3/mascot/cgi/peptide_view.pl?file=../data/20121119/F002569.dat&query=2154&hit=1&index=gi%7c00000001&px=1))

**185 - 198 775.0218 1548.0290 1546.6609 1.3681 0 K.GAYCLSVSDFDNAK.G**  Carbamidomethyl (C); Deamidation (NQ) ([Ions score 80](http://df3/mascot/cgi/peptide_view.pl?file=../data/20121119/F002569.dat&query=2155&hit=1&index=gi%7c00000001&px=1))

**185 - 203 686.9508 2057.8306 2056.9887 0.8419 1 K.GAYCLSVSDFDNAKGLNVK.H**  Carbamidomethyl (C) ([Ions score 61](http://df3/mascot/cgi/peptide_view.pl?file=../data/20121119/F002569.dat&query=1521&hit=1&index=gi%7c00000001&px=1))

**185 - 203 687.3584 2059.0534 2057.9727 1.0806 1 K.GAYCLSVSDFDNAKGLNVK.H**  Carbamidomethyl (C); Deamidation (NQ) ([Ions score 60](http://df3/mascot/cgi/peptide_view.pl?file=../data/20121119/F002569.dat&query=1524&hit=1&index=gi%7c00000001&px=1))

**209 - 220 672.2754 1342.5362 1342.6881 -0.1518 1 R.KLDSGGFYITSR.T**  ([Ions score 108](http://df3/mascot/cgi/peptide_view.pl?file=../data/20121119/F002569.dat&query=1468&hit=1&index=gi%7c00000001&px=1))

**209 - 220 672.4230 1342.8314 1342.6881 0.1433 1 R.KLDSGGFYITSR.T**  ([Ions score 21](http://df3/mascot/cgi/peptide_view.pl?file=../data/20121119/F002569.dat&query=1469&hit=1&index=gi%7c00000001&px=1))

**209 - 220 672.5264 1343.0382 1342.6881 0.3501 1 R.KLDSGGFYITSR.T**  ([Ions score 30](http://df3/mascot/cgi/peptide_view.pl?file=../data/20121119/F002569.dat&query=1470&hit=1&index=gi%7c00000001&px=1))

**209 - 220 448.9995 1343.9767 1342.6881 1.2886 1 R.KLDSGGFYITSR.T**  ([Ions score 31](http://df3/mascot/cgi/peptide_view.pl?file=../data/20121119/F002569.dat&query=451&hit=1&index=gi%7c00000001&px=1))

**209 - 220 673.1077 1344.2008 1342.6881 1.5127 1 R.KLDSGGFYITSR.T**  ([Ions score 87](http://df3/mascot/cgi/peptide_view.pl?file=../data/20121119/F002569.dat&query=1471&hit=1&index=gi%7c00000001&px=1))

**209 - 220 449.0884 1344.2433 1342.6881 1.5552 1 R.KLDSGGFYITSR.T**  ([Ions score 38](http://df3/mascot/cgi/peptide_view.pl?file=../data/20121119/F002569.dat&query=454&hit=1&index=gi%7c00000001&px=1))

**210 - 220 608.5112 1215.0079 1214.5931 0.4148 0 K.LDSGGFYITSR.T**  ([Ions score 98](http://df3/mascot/cgi/peptide_view.pl?file=../data/20121119/F002569.dat&query=1206&hit=1&index=gi%7c00000001&px=1))

**210 - 220 608.8934 1215.7722 1214.5931 1.1791 0 K.LDSGGFYITSR.T**  ([Ions score 90](http://df3/mascot/cgi/peptide_view.pl?file=../data/20121119/F002569.dat&query=1207&hit=1&index=gi%7c00000001&px=1))

**210 - 220 609.0460 1216.0775 1214.5931 1.4844 0 K.LDSGGFYITSR.T**  ([Ions score 92](http://df3/mascot/cgi/peptide_view.pl?file=../data/20121119/F002569.dat&query=1208&hit=1&index=gi%7c00000001&px=1))

**221 - 235 894.7627 1787.5108 1788.9046 -1.3938 0 R.TQFNSLQQLVAYYSK.H**  ([Ions score 87](http://df3/mascot/cgi/peptide_view.pl?file=../data/20121119/F002569.dat&query=2720&hit=1&index=gi%7c00000001&px=1))

**221 - 235 895.2770 1788.5394 1788.9046 -0.3652 0 R.TQFNSLQQLVAYYSK.H**  ([Ions score 28](http://df3/mascot/cgi/peptide_view.pl?file=../data/20121119/F002569.dat&query=2721&hit=1&index=gi%7c00000001&px=1))

**221 - 235 895.3251 1788.6356 1788.9046 -0.2690 0 R.TQFNSLQQLVAYYSK.H**  ([Ions score 91](http://df3/mascot/cgi/peptide_view.pl?file=../data/20121119/F002569.dat&query=2722&hit=1&index=gi%7c00000001&px=1))

**221 - 235 895.4171 1788.8197 1788.9046 -0.0849 0 R.TQFNSLQQLVAYYSK.H**  ([Ions score 108](http://df3/mascot/cgi/peptide_view.pl?file=../data/20121119/F002569.dat&query=2723&hit=1&index=gi%7c00000001&px=1))

**221 - 235 895.4440 1788.8734 1788.9046 -0.0312 0 R.TQFNSLQQLVAYYSK.H**  ([Ions score 95](http://df3/mascot/cgi/peptide_view.pl?file=../data/20121119/F002569.dat&query=2724&hit=1&index=gi%7c00000001&px=1))

**221 - 235 895.4670 1788.9195 1788.9046 0.0149 0 R.TQFNSLQQLVAYYSK.H**  ([Ions score 82](http://df3/mascot/cgi/peptide_view.pl?file=../data/20121119/F002569.dat&query=2725&hit=1&index=gi%7c00000001&px=1))

**221 - 235 895.4736 1788.9327 1788.9046 0.0281 0 R.TQFNSLQQLVAYYSK.H**  ([Ions score 87](http://df3/mascot/cgi/peptide_view.pl?file=../data/20121119/F002569.dat&query=2726&hit=1&index=gi%7c00000001&px=1))

**221 - 235 895.4948 1788.9750 1788.9046 0.0703 0 R.TQFNSLQQLVAYYSK.H**  ([Ions score 95](http://df3/mascot/cgi/peptide_view.pl?file=../data/20121119/F002569.dat&query=2727&hit=1&index=gi%7c00000001&px=1))

**221 - 235 895.6890 1789.3634 1788.9046 0.4588 0 R.TQFNSLQQLVAYYSK.H**  ([Ions score 93](http://df3/mascot/cgi/peptide_view.pl?file=../data/20121119/F002569.dat&query=2728&hit=1&index=gi%7c00000001&px=1))

**221 - 235 895.8110 1789.6075 1788.9046 0.7029 0 R.TQFNSLQQLVAYYSK.H**  ([Ions score 86](http://df3/mascot/cgi/peptide_view.pl?file=../data/20121119/F002569.dat&query=2729&hit=1&index=gi%7c00000001&px=1))

**221 - 235 895.8477 1789.6808 1788.9046 0.7762 0 R.TQFNSLQQLVAYYSK.H**  ([Ions score 83](http://df3/mascot/cgi/peptide_view.pl?file=../data/20121119/F002569.dat&query=2730&hit=1&index=gi%7c00000001&px=1))

**221 - 235 597.6059 1789.7959 1788.9046 0.8913 0 R.TQFNSLQQLVAYYSK.H**  ([Ions score 70](http://df3/mascot/cgi/peptide_view.pl?file=../data/20121119/F002569.dat&query=1161&hit=1&index=gi%7c00000001&px=1))

**221 - 235 597.6832 1790.0277 1788.9046 1.1231 0 R.TQFNSLQQLVAYYSK.H**  ([Ions score 80](http://df3/mascot/cgi/peptide_view.pl?file=../data/20121119/F002569.dat&query=1162&hit=1&index=gi%7c00000001&px=1))

**221 - 235 597.6951 1790.0634 1788.9046 1.1588 0 R.TQFNSLQQLVAYYSK.H**  ([Ions score 96](http://df3/mascot/cgi/peptide_view.pl?file=../data/20121119/F002569.dat&query=1163&hit=1&index=gi%7c00000001&px=1))

**221 - 235 597.6975 1790.0707 1788.9046 1.1661 0 R.TQFNSLQQLVAYYSK.H**  ([Ions score 85](http://df3/mascot/cgi/peptide_view.pl?file=../data/20121119/F002569.dat&query=1164&hit=1&index=gi%7c00000001&px=1))

**221 - 235 597.7917 1790.3534 1788.9046 1.4488 0 R.TQFNSLQQLVAYYSK.H**  ([Ions score 77](http://df3/mascot/cgi/peptide_view.pl?file=../data/20121119/F002569.dat&query=1165&hit=1&index=gi%7c00000001&px=1))

**221 - 235 896.3246 1790.6346 1788.9046 1.7300 0 R.TQFNSLQQLVAYYSK.H**  ([Ions score 90](http://df3/mascot/cgi/peptide_view.pl?file=../data/20121119/F002569.dat&query=2734&hit=1&index=gi%7c00000001&px=1))

**221 - 235 896.3282 1790.6419 1788.9046 1.7373 0 R.TQFNSLQQLVAYYSK.H**  ([Ions score 38](http://df3/mascot/cgi/peptide_view.pl?file=../data/20121119/F002569.dat&query=2735&hit=1&index=gi%7c00000001&px=1))

**221 - 235 597.9519 1790.8339 1788.9046 1.9293 0 R.TQFNSLQQLVAYYSK.H**  ([Ions score 63](http://df3/mascot/cgi/peptide_view.pl?file=../data/20121119/F002569.dat&query=1166&hit=1&index=gi%7c00000001&px=1))

**221 - 235 896.3142 1790.6139 1789.8886 0.7253 0 R.TQFNSLQQLVAYYSK.H**  Deamidation (NQ) ([Ions score 90](http://df3/mascot/cgi/peptide_view.pl?file=../data/20121119/F002569.dat&query=2733&hit=1&index=gi%7c00000001&px=1))

**221 - 235 896.4188 1790.8231 1789.8886 0.9345 0 R.TQFNSLQQLVAYYSK.H**  Deamidation (NQ) ([Ions score 83](http://df3/mascot/cgi/peptide_view.pl?file=../data/20121119/F002569.dat&query=2736&hit=1&index=gi%7c00000001&px=1))

**221 - 235 896.4709 1790.9273 1789.8886 1.0387 0 R.TQFNSLQQLVAYYSK.H**  Deamidation (NQ) ([Ions score 65](http://df3/mascot/cgi/peptide_view.pl?file=../data/20121119/F002569.dat&query=2737&hit=1&index=gi%7c00000001&px=1))

**221 - 235 896.4789 1790.9432 1789.8886 1.0546 0 R.TQFNSLQQLVAYYSK.H**  Deamidation (NQ) ([Ions score 87](http://df3/mascot/cgi/peptide_view.pl?file=../data/20121119/F002569.dat&query=2738&hit=1&index=gi%7c00000001&px=1))

**221 - 235 896.5441 1791.0736 1789.8886 1.1850 0 R.TQFNSLQQLVAYYSK.H**  Deamidation (NQ) ([Ions score 68](http://df3/mascot/cgi/peptide_view.pl?file=../data/20121119/F002569.dat&query=2739&hit=1&index=gi%7c00000001&px=1))

**221 - 235 896.7118 1791.4090 1789.8886 1.5204 0 R.TQFNSLQQLVAYYSK.H**  Deamidation (NQ) ([Ions score 74](http://df3/mascot/cgi/peptide_view.pl?file=../data/20121119/F002569.dat&query=2741&hit=1&index=gi%7c00000001&px=1))

**221 - 235 598.1725 1791.4956 1789.8886 1.6070 0 R.TQFNSLQQLVAYYSK.H**  Deamidation (NQ) ([Ions score 84](http://df3/mascot/cgi/peptide_view.pl?file=../data/20121119/F002569.dat&query=1168&hit=1&index=gi%7c00000001&px=1))

**221 - 235 896.8068 1791.5990 1789.8886 1.7104 0 R.TQFNSLQQLVAYYSK.H**  Deamidation (NQ) ([Ions score 31](http://df3/mascot/cgi/peptide_view.pl?file=../data/20121119/F002569.dat&query=2742&hit=1&index=gi%7c00000001&px=1))

**221 - 235 896.9482 1791.8819 1789.8886 1.9933 0 R.TQFNSLQQLVAYYSK.H**  Deamidation (NQ) ([Ions score 86](http://df3/mascot/cgi/peptide_view.pl?file=../data/20121119/F002569.dat&query=2744&hit=1&index=gi%7c00000001&px=1))

**221 - 235 897.0759 1792.1373 1790.8726 1.2647 0 R.TQFNSLQQLVAYYSK.H**  2 Deamidation (NQ) ([Ions score 63](http://df3/mascot/cgi/peptide_view.pl?file=../data/20121119/F002569.dat&query=2745&hit=1&index=gi%7c00000001&px=1))

**221 - 235 598.4594 1792.3562 1790.8726 1.4836 0 R.TQFNSLQQLVAYYSK.H**  2 Deamidation (NQ) ([Ions score 67](http://df3/mascot/cgi/peptide_view.pl?file=../data/20121119/F002569.dat&query=1171&hit=1&index=gi%7c00000001&px=1))

**236 - 260 923.5371 2767.5895 2767.5596 0.0299 1 K.HADGLCHRLTTVCPTSKPQTQGLAK.D**  2 HNE (H C) ([Ions score 24](http://df3/mascot/cgi/peptide_view.pl?file=../data/20121119/F002569.dat&query=2793&hit=1&index=gi%7c00000001&px=1))

**236 - 260 923.2971 2766.8695 2768.5436 -1.6740 1 K.HADGLCHRLTTVCPTSKPQTQGLAK.D**  Deamidation (NQ); 2 HNE (H C) ([Ions score 14](http://df3/mascot/cgi/peptide_view.pl?file=../data/20121119/F002569.dat&query=2792&hit=1&index=gi%7c00000001&px=1))

**244 - 260 592.0341 1773.0803 1771.9502 1.1302 0 R.LTTVCPTSKPQTQGLAK.D**  ([Ions score 27](http://df3/mascot/cgi/peptide_view.pl?file=../data/20121119/F002569.dat&query=1104&hit=1&index=gi%7c00000001&px=1))

**244 - 260 610.2113 1827.6121 1828.9717 -1.3596 0 R.LTTVCPTSKPQTQGLAK.D**  Carbamidomethyl (C) ([Ions score 28](http://df3/mascot/cgi/peptide_view.pl?file=../data/20121119/F002569.dat&query=1212&hit=1&index=gi%7c00000001&px=1))

**244 - 260 915.0437 1828.0729 1828.9717 -0.8988 0 R.LTTVCPTSKPQTQGLAK.D**  Carbamidomethyl (C) ([Ions score 86](http://df3/mascot/cgi/peptide_view.pl?file=../data/20121119/F002569.dat&query=2780&hit=1&index=gi%7c00000001&px=1))

**244 - 260 610.3782 1828.1129 1828.9717 -0.8588 0 R.LTTVCPTSKPQTQGLAK.D**  Carbamidomethyl (C) ([Ions score 32](http://df3/mascot/cgi/peptide_view.pl?file=../data/20121119/F002569.dat&query=1214&hit=1&index=gi%7c00000001&px=1))

**244 - 260 915.4446 1828.8746 1828.9717 -0.0971 0 R.LTTVCPTSKPQTQGLAK.D**  Carbamidomethyl (C) ([Ions score 46](http://df3/mascot/cgi/peptide_view.pl?file=../data/20121119/F002569.dat&query=2781&hit=1&index=gi%7c00000001&px=1))

**244 - 260 915.8782 1829.7418 1828.9717 0.7701 0 R.LTTVCPTSKPQTQGLAK.D**  Carbamidomethyl (C) ([Ions score 72](http://df3/mascot/cgi/peptide_view.pl?file=../data/20121119/F002569.dat&query=2783&hit=1&index=gi%7c00000001&px=1))

**244 - 260 610.9368 1829.7887 1828.9717 0.8170 0 R.LTTVCPTSKPQTQGLAK.D**  Carbamidomethyl (C) ([Ions score 20](http://df3/mascot/cgi/peptide_view.pl?file=../data/20121119/F002569.dat&query=1223&hit=1&index=gi%7c00000001&px=1))

**244 - 260 611.0532 1830.1378 1828.9717 1.1662 0 R.LTTVCPTSKPQTQGLAK.D**  Carbamidomethyl (C) ([Ions score 23](http://df3/mascot/cgi/peptide_view.pl?file=../data/20121119/F002569.dat&query=1228&hit=1&index=gi%7c00000001&px=1))

**244 - 260 611.0542 1830.1408 1828.9717 1.1691 0 R.LTTVCPTSKPQTQGLAK.D**  Carbamidomethyl (C) ([Ions score 10](http://df3/mascot/cgi/peptide_view.pl?file=../data/20121119/F002569.dat&query=1229&hit=1&index=gi%7c00000001&px=1))

**244 - 260 916.0935 1830.1725 1828.9717 1.2008 0 R.LTTVCPTSKPQTQGLAK.D**  Carbamidomethyl (C) ([Ions score 83](http://df3/mascot/cgi/peptide_view.pl?file=../data/20121119/F002569.dat&query=2784&hit=1&index=gi%7c00000001&px=1))

**244 - 260 611.2593 1830.7560 1828.9717 1.7843 0 R.LTTVCPTSKPQTQGLAK.D**  Carbamidomethyl (C) ([Ions score 17](http://df3/mascot/cgi/peptide_view.pl?file=../data/20121119/F002569.dat&query=1231&hit=1&index=gi%7c00000001&px=1))

**244 - 260 610.5305 1828.5695 1829.9557 -1.3861 0 R.LTTVCPTSKPQTQGLAK.D**  Carbamidomethyl (C); Deamidation (NQ) ([Ions score 14](http://df3/mascot/cgi/peptide_view.pl?file=../data/20121119/F002569.dat&query=1215&hit=1&index=gi%7c00000001&px=1))

**244 - 260 610.5869 1828.7389 1829.9557 -1.2168 0 R.LTTVCPTSKPQTQGLAK.D**  Carbamidomethyl (C); Deamidation (NQ) ([Ions score 15](http://df3/mascot/cgi/peptide_view.pl?file=../data/20121119/F002569.dat&query=1216&hit=1&index=gi%7c00000001&px=1))

**244 - 260 610.8674 1829.5803 1829.9557 -0.3754 0 R.LTTVCPTSKPQTQGLAK.D**  Carbamidomethyl (C); Deamidation (NQ) ([Ions score 15](http://df3/mascot/cgi/peptide_view.pl?file=../data/20121119/F002569.dat&query=1219&hit=1&index=gi%7c00000001&px=1))

**244 - 260 610.8674 1829.5805 1829.9557 -0.3752 0 R.LTTVCPTSKPQTQGLAK.D**  Carbamidomethyl (C); Deamidation (NQ) ([Ions score 23](http://df3/mascot/cgi/peptide_view.pl?file=../data/20121119/F002569.dat&query=1220&hit=1&index=gi%7c00000001&px=1))

**244 - 260 610.8767 1829.6083 1829.9557 -0.3474 0 R.LTTVCPTSKPQTQGLAK.D**  Carbamidomethyl (C); Deamidation (NQ) ([Ions score 22](http://df3/mascot/cgi/peptide_view.pl?file=../data/20121119/F002569.dat&query=1221&hit=1&index=gi%7c00000001&px=1))

**244 - 260 915.8296 1829.6446 1829.9557 -0.3110 0 R.LTTVCPTSKPQTQGLAK.D**  Carbamidomethyl (C); Deamidation (NQ) ([Ions score 73](http://df3/mascot/cgi/peptide_view.pl?file=../data/20121119/F002569.dat&query=2782&hit=1&index=gi%7c00000001&px=1))

**244 - 260 610.9306 1829.7700 1829.9557 -0.1857 0 R.LTTVCPTSKPQTQGLAK.D**  Carbamidomethyl (C); Deamidation (NQ) ([Ions score 15](http://df3/mascot/cgi/peptide_view.pl?file=../data/20121119/F002569.dat&query=1222&hit=1&index=gi%7c00000001&px=1))

**244 - 260 610.9396 1829.7969 1829.9557 -0.1588 0 R.LTTVCPTSKPQTQGLAK.D**  Carbamidomethyl (C); Deamidation (NQ) ([Ions score 14](http://df3/mascot/cgi/peptide_view.pl?file=../data/20121119/F002569.dat&query=1224&hit=1&index=gi%7c00000001&px=1))

**244 - 260 610.9477 1829.8213 1829.9557 -0.1344 0 R.LTTVCPTSKPQTQGLAK.D**  Carbamidomethyl (C); Deamidation (NQ) ([Ions score 14](http://df3/mascot/cgi/peptide_view.pl?file=../data/20121119/F002569.dat&query=1225&hit=1&index=gi%7c00000001&px=1))

**244 - 260 610.9688 1829.8844 1829.9557 -0.0713 0 R.LTTVCPTSKPQTQGLAK.D**  Carbamidomethyl (C); Deamidation (NQ) ([Ions score 13](http://df3/mascot/cgi/peptide_view.pl?file=../data/20121119/F002569.dat&query=1226&hit=1&index=gi%7c00000001&px=1))

**244 - 260 610.9900 1829.9481 1829.9557 -0.0075 0 R.LTTVCPTSKPQTQGLAK.D**  Carbamidomethyl (C); Deamidation (NQ) ([Ions score 21](http://df3/mascot/cgi/peptide_view.pl?file=../data/20121119/F002569.dat&query=1227&hit=1&index=gi%7c00000001&px=1))

**244 - 260 611.6581 1831.9526 1829.9557 1.9969 0 R.LTTVCPTSKPQTQGLAK.D**  Carbamidomethyl (C); Deamidation (NQ) ([Ions score 33](http://df3/mascot/cgi/peptide_view.pl?file=../data/20121119/F002569.dat&query=1232&hit=1&index=gi%7c00000001&px=1))

**261 - 267 443.4898 884.9651 885.4344 -0.4694 0 K.DAWEIPR.E**  ([Ions score 16](http://df3/mascot/cgi/peptide_view.pl?file=../data/20121119/F002569.dat&query=399&hit=1&index=gi%7c00000001&px=1))

**261 - 267 443.6636 885.3126 885.4344 -0.1219 0 K.DAWEIPR.E**  ([Ions score 32](http://df3/mascot/cgi/peptide_view.pl?file=../data/20121119/F002569.dat&query=400&hit=1&index=gi%7c00000001&px=1))

**261 - 267 443.6965 885.3785 885.4344 -0.0560 0 K.DAWEIPR.E**  ([Ions score 33](http://df3/mascot/cgi/peptide_view.pl?file=../data/20121119/F002569.dat&query=401&hit=1&index=gi%7c00000001&px=1))

**261 - 267 443.9162 885.8178 885.4344 0.3834 0 K.DAWEIPR.E**  ([Ions score 51](http://df3/mascot/cgi/peptide_view.pl?file=../data/20121119/F002569.dat&query=403&hit=1&index=gi%7c00000001&px=1))

**261 - 267 444.1174 886.2202 885.4344 0.7857 0 K.DAWEIPR.E**  ([Ions score 35](http://df3/mascot/cgi/peptide_view.pl?file=../data/20121119/F002569.dat&query=405&hit=1&index=gi%7c00000001&px=1))

**261 - 271 686.4974 1370.9802 1370.6942 0.2860 1 K.DAWEIPRESLR.L**  ([Ions score 46](http://df3/mascot/cgi/peptide_view.pl?file=../data/20121119/F002569.dat&query=1518&hit=1&index=gi%7c00000001&px=1))

**261 - 271 686.5864 1371.1583 1370.6942 0.4641 1 K.DAWEIPRESLR.L**  ([Ions score 53](http://df3/mascot/cgi/peptide_view.pl?file=../data/20121119/F002569.dat&query=1519&hit=1&index=gi%7c00000001&px=1))

**261 - 271 686.9426 1371.8707 1370.6942 1.1765 1 K.DAWEIPRESLR.L**  ([Ions score 44](http://df3/mascot/cgi/peptide_view.pl?file=../data/20121119/F002569.dat&query=1520&hit=1&index=gi%7c00000001&px=1))

**261 - 271 458.3368 1371.9885 1370.6942 1.2943 1 K.DAWEIPRESLR.L**  ([Ions score 21](http://df3/mascot/cgi/peptide_view.pl?file=../data/20121119/F002569.dat&query=488&hit=1&index=gi%7c00000001&px=1))

**261 - 271 458.3512 1372.0319 1370.6942 1.3376 1 K.DAWEIPRESLR.L**  ([Ions score 22](http://df3/mascot/cgi/peptide_view.pl?file=../data/20121119/F002569.dat&query=489&hit=1&index=gi%7c00000001&px=1))

**261 - 271 687.0680 1372.1214 1370.6942 1.4272 1 K.DAWEIPRESLR.L**  ([Ions score 45](http://df3/mascot/cgi/peptide_view.pl?file=../data/20121119/F002569.dat&query=1522&hit=1&index=gi%7c00000001&px=1))

**261 - 271 458.4110 1372.2112 1370.6942 1.5170 1 K.DAWEIPRESLR.L**  ([Ions score 19](http://df3/mascot/cgi/peptide_view.pl?file=../data/20121119/F002569.dat&query=490&hit=1&index=gi%7c00000001&px=1))

**261 - 271 458.4293 1372.2660 1370.6942 1.5718 1 K.DAWEIPRESLR.L**  ([Ions score 27](http://df3/mascot/cgi/peptide_view.pl?file=../data/20121119/F002569.dat&query=491&hit=1&index=gi%7c00000001&px=1))

**276 - 294 1086.9824 2171.9503 2171.9516 -0.0013 0 K.LGQGCFGEVWMGTWNGTTR.V**  Carbamidomethyl (C); Oxidation (M) ([Ions score 106](http://df3/mascot/cgi/peptide_view.pl?file=../data/20121119/F002569.dat&query=2841&hit=1&index=gi%7c00000001&px=1))

**276 - 294 725.1686 2172.4841 2171.9516 0.5325 0 K.LGQGCFGEVWMGTWNGTTR.V**  Carbamidomethyl (C); Oxidation (M) ([Ions score 50](http://df3/mascot/cgi/peptide_view.pl?file=../data/20121119/F002569.dat&query=1654&hit=1&index=gi%7c00000001&px=1))

**276 - 294 725.4063 2173.1969 2171.9516 1.2453 0 K.LGQGCFGEVWMGTWNGTTR.V**  Carbamidomethyl (C); Oxidation (M) ([Ions score 53](http://df3/mascot/cgi/peptide_view.pl?file=../data/20121119/F002569.dat&query=1655&hit=1&index=gi%7c00000001&px=1))

**276 - 294 725.4327 2173.2764 2171.9516 1.3248 0 K.LGQGCFGEVWMGTWNGTTR.V**  Carbamidomethyl (C); Oxidation (M) ([Ions score 41](http://df3/mascot/cgi/peptide_view.pl?file=../data/20121119/F002569.dat&query=1656&hit=1&index=gi%7c00000001&px=1))

**276 - 294 1087.7852 2173.5558 2171.9516 1.6041 0 K.LGQGCFGEVWMGTWNGTTR.V**  Carbamidomethyl (C); Oxidation (M) ([Ions score 105](http://df3/mascot/cgi/peptide_view.pl?file=../data/20121119/F002569.dat&query=2842&hit=1&index=gi%7c00000001&px=1))

**276 - 294 1087.8414 2173.6683 2172.9356 0.7327 0 K.LGQGCFGEVWMGTWNGTTR.V**  Carbamidomethyl (C); Deamidation (NQ); Oxidation (M) ([Ions score 113](http://df3/mascot/cgi/peptide_view.pl?file=../data/20121119/F002569.dat&query=2843&hit=1&index=gi%7c00000001&px=1))

**276 - 294 1087.8423 2173.6700 2172.9356 0.7344 0 K.LGQGCFGEVWMGTWNGTTR.V**  Carbamidomethyl (C); Deamidation (NQ); Oxidation (M) ([Ions score 102](http://df3/mascot/cgi/peptide_view.pl?file=../data/20121119/F002569.dat&query=2844&hit=1&index=gi%7c00000001&px=1))

**276 - 294 725.7700 2174.2882 2172.9356 1.3526 0 K.LGQGCFGEVWMGTWNGTTR.V**  Carbamidomethyl (C); Deamidation (NQ); Oxidation (M) ([Ions score 61](http://df3/mascot/cgi/peptide_view.pl?file=../data/20121119/F002569.dat&query=1657&hit=1&index=gi%7c00000001&px=1))

**295 - 318 874.0651 2619.1734 2617.3858 1.7875 1 R.VAIKTLKPGTMSPEAFLQEAQVMK.K**  Deamidation (NQ) ([Ions score 18](http://df3/mascot/cgi/peptide_view.pl?file=../data/20121119/F002569.dat&query=2571&hit=1&index=gi%7c00000001&px=1))

**295 - 318 878.6342 2632.8806 2632.3967 0.4839 1 R.VAIKTLKPGTMSPEAFLQEAQVMK.K**  Oxidation (M) ([Ions score 47](http://df3/mascot/cgi/peptide_view.pl?file=../data/20121119/F002569.dat&query=2645&hit=1&index=gi%7c00000001&px=1))

**295 - 318 884.1475 2649.4206 2648.3917 1.0289 1 R.VAIKTLKPGTMSPEAFLQEAQVMK.K**  2 Oxidation (M) ([Ions score 65](http://df3/mascot/cgi/peptide_view.pl?file=../data/20121119/F002569.dat&query=2687&hit=1&index=gi%7c00000001&px=1))

**295 - 318 884.6426 2650.9059 2649.3757 1.5302 1 R.VAIKTLKPGTMSPEAFLQEAQVMK.K**  Deamidation (NQ); 2 Oxidation (M) ([Ions score 53](http://df3/mascot/cgi/peptide_view.pl?file=../data/20121119/F002569.dat&query=2693&hit=1&index=gi%7c00000001&px=1))

**299 - 318 735.6364 2203.8874 2205.1173 -1.2299 0 K.TLKPGTMSPEAFLQEAQVMK.K**  ([Ions score 14](http://df3/mascot/cgi/peptide_view.pl?file=../data/20121119/F002569.dat&query=1805&hit=1&index=gi%7c00000001&px=1))

**299 - 318 736.3164 2205.9274 2205.1173 0.8101 0 K.TLKPGTMSPEAFLQEAQVMK.K**  ([Ions score 41](http://df3/mascot/cgi/peptide_view.pl?file=../data/20121119/F002569.dat&query=1811&hit=1&index=gi%7c00000001&px=1))

**299 - 318 736.6193 2206.8360 2205.1173 1.7187 0 K.TLKPGTMSPEAFLQEAQVMK.K**  ([Ions score 27](http://df3/mascot/cgi/peptide_view.pl?file=../data/20121119/F002569.dat&query=1812&hit=1&index=gi%7c00000001&px=1))

**299 - 318 740.8018 2219.3836 2221.1122 -1.7286 0 K.TLKPGTMSPEAFLQEAQVMK.K**  Oxidation (M) ([Ions score 56](http://df3/mascot/cgi/peptide_view.pl?file=../data/20121119/F002569.dat&query=1869&hit=1&index=gi%7c00000001&px=1))

**299 - 318 741.2125 2220.6157 2221.1122 -0.4965 0 K.TLKPGTMSPEAFLQEAQVMK.K**  Oxidation (M) ([Ions score 14](http://df3/mascot/cgi/peptide_view.pl?file=../data/20121119/F002569.dat&query=1873&hit=1&index=gi%7c00000001&px=1))

**299 - 318 741.3467 2221.0184 2221.1122 -0.0938 0 K.TLKPGTMSPEAFLQEAQVMK.K**  Oxidation (M) ([Ions score 22](http://df3/mascot/cgi/peptide_view.pl?file=../data/20121119/F002569.dat&query=1875&hit=1&index=gi%7c00000001&px=1))

**299 - 318 1111.9387 2221.8629 2221.1122 0.7507 0 K.TLKPGTMSPEAFLQEAQVMK.K**  Oxidation (M) ([Ions score 59](http://df3/mascot/cgi/peptide_view.pl?file=../data/20121119/F002569.dat&query=2891&hit=1&index=gi%7c00000001&px=1))

**299 - 318 741.8710 2222.5911 2221.1122 1.4789 0 K.TLKPGTMSPEAFLQEAQVMK.K**  Oxidation (M) ([Ions score 35](http://df3/mascot/cgi/peptide_view.pl?file=../data/20121119/F002569.dat&query=1896&hit=1&index=gi%7c00000001&px=1))

**299 - 318 742.0092 2223.0056 2221.1122 1.8934 0 K.TLKPGTMSPEAFLQEAQVMK.K**  Oxidation (M) ([Ions score 57](http://df3/mascot/cgi/peptide_view.pl?file=../data/20121119/F002569.dat&query=1897&hit=1&index=gi%7c00000001&px=1))

**299 - 318 741.7750 2222.3031 2222.0962 0.2068 0 K.TLKPGTMSPEAFLQEAQVMK.K**  Deamidation (NQ); Oxidation (M) ([Ions score 12](http://df3/mascot/cgi/peptide_view.pl?file=../data/20121119/F002569.dat&query=1891&hit=1&index=gi%7c00000001&px=1))

**299 - 318 741.8648 2222.5726 2222.0962 0.4764 0 K.TLKPGTMSPEAFLQEAQVMK.K**  Deamidation (NQ); Oxidation (M) ([Ions score 56](http://df3/mascot/cgi/peptide_view.pl?file=../data/20121119/F002569.dat&query=1895&hit=1&index=gi%7c00000001&px=1))

**299 - 318 742.0841 2223.2305 2222.0962 1.1343 0 K.TLKPGTMSPEAFLQEAQVMK.K**  Deamidation (NQ); Oxidation (M) ([Ions score 15](http://df3/mascot/cgi/peptide_view.pl?file=../data/20121119/F002569.dat&query=1898&hit=1&index=gi%7c00000001&px=1))

**299 - 318 742.1614 2223.4623 2222.0962 1.3661 0 K.TLKPGTMSPEAFLQEAQVMK.K**  Deamidation (NQ); Oxidation (M) ([Ions score 29](http://df3/mascot/cgi/peptide_view.pl?file=../data/20121119/F002569.dat&query=1899&hit=1&index=gi%7c00000001&px=1))

**299 - 318 742.2937 2223.8593 2222.0962 1.7631 0 K.TLKPGTMSPEAFLQEAQVMK.K**  Deamidation (NQ); Oxidation (M) ([Ions score 42](http://df3/mascot/cgi/peptide_view.pl?file=../data/20121119/F002569.dat&query=1901&hit=1&index=gi%7c00000001&px=1))

**299 - 318 746.0497 2235.1272 2237.1071 -1.9799 0 K.TLKPGTMSPEAFLQEAQVMK.K**  2 Oxidation (M) ([Ions score 61](http://df3/mascot/cgi/peptide_view.pl?file=../data/20121119/F002569.dat&query=1927&hit=1&index=gi%7c00000001&px=1))

**299 - 318 746.1116 2235.3129 2237.1071 -1.7942 0 K.TLKPGTMSPEAFLQEAQVMK.K**  2 Oxidation (M) ([Ions score 14](http://df3/mascot/cgi/peptide_view.pl?file=../data/20121119/F002569.dat&query=1930&hit=1&index=gi%7c00000001&px=1))

**299 - 318 746.2763 2235.8071 2237.1071 -1.3000 0 K.TLKPGTMSPEAFLQEAQVMK.K**  2 Oxidation (M) ([Ions score 56](http://df3/mascot/cgi/peptide_view.pl?file=../data/20121119/F002569.dat&query=1934&hit=1&index=gi%7c00000001&px=1))

**299 - 318 746.3713 2236.0922 2237.1071 -1.0149 0 K.TLKPGTMSPEAFLQEAQVMK.K**  2 Oxidation (M) ([Ions score 18](http://df3/mascot/cgi/peptide_view.pl?file=../data/20121119/F002569.dat&query=1941&hit=1&index=gi%7c00000001&px=1))

**299 - 318 1119.4061 2236.7977 2237.1071 -0.3094 0 K.TLKPGTMSPEAFLQEAQVMK.K**  2 Oxidation (M) ([Ions score 55](http://df3/mascot/cgi/peptide_view.pl?file=../data/20121119/F002569.dat&query=2896&hit=1&index=gi%7c00000001&px=1))

**299 - 318 746.8713 2237.5922 2237.1071 0.4851 0 K.TLKPGTMSPEAFLQEAQVMK.K**  2 Oxidation (M) ([Ions score 21](http://df3/mascot/cgi/peptide_view.pl?file=../data/20121119/F002569.dat&query=1943&hit=1&index=gi%7c00000001&px=1))

**299 - 318 1119.8865 2237.7584 2237.1071 0.6513 0 K.TLKPGTMSPEAFLQEAQVMK.K**  2 Oxidation (M) ([Ions score 59](http://df3/mascot/cgi/peptide_view.pl?file=../data/20121119/F002569.dat&query=2897&hit=1&index=gi%7c00000001&px=1))

**299 - 318 747.0510 2238.1312 2237.1071 1.0241 0 K.TLKPGTMSPEAFLQEAQVMK.K**  2 Oxidation (M) ([Ions score 30](http://df3/mascot/cgi/peptide_view.pl?file=../data/20121119/F002569.dat&query=1946&hit=1&index=gi%7c00000001&px=1))

**299 - 318 747.0745 2238.2016 2237.1071 1.0944 0 K.TLKPGTMSPEAFLQEAQVMK.K**  2 Oxidation (M) ([Ions score 22](http://df3/mascot/cgi/peptide_view.pl?file=../data/20121119/F002569.dat&query=1947&hit=1&index=gi%7c00000001&px=1))

**299 - 318 747.2546 2238.7421 2237.1071 1.6350 0 K.TLKPGTMSPEAFLQEAQVMK.K**  2 Oxidation (M) ([Ions score 35](http://df3/mascot/cgi/peptide_view.pl?file=../data/20121119/F002569.dat&query=1951&hit=1&index=gi%7c00000001&px=1))

**299 - 318 747.3149 2238.9230 2237.1071 1.8159 0 K.TLKPGTMSPEAFLQEAQVMK.K**  2 Oxidation (M) ([Ions score 37](http://df3/mascot/cgi/peptide_view.pl?file=../data/20121119/F002569.dat&query=1954&hit=1&index=gi%7c00000001&px=1))

**299 - 318 747.3693 2239.0860 2237.1071 1.9788 0 K.TLKPGTMSPEAFLQEAQVMK.K**  2 Oxidation (M) ([Ions score 14](http://df3/mascot/cgi/peptide_view.pl?file=../data/20121119/F002569.dat&query=1956&hit=1&index=gi%7c00000001&px=1))

**299 - 318 746.9279 2237.7617 2238.0911 -0.3294 0 K.TLKPGTMSPEAFLQEAQVMK.K**  Deamidation (NQ); 2 Oxidation (M) ([Ions score 18](http://df3/mascot/cgi/peptide_view.pl?file=../data/20121119/F002569.dat&query=1944&hit=1&index=gi%7c00000001&px=1))

**299 - 318 747.0457 2238.1151 2238.0911 0.0240 0 K.TLKPGTMSPEAFLQEAQVMK.K**  Deamidation (NQ); 2 Oxidation (M) ([Ions score 15](http://df3/mascot/cgi/peptide_view.pl?file=../data/20121119/F002569.dat&query=1945&hit=1&index=gi%7c00000001&px=1))

**299 - 318 747.1702 2238.4887 2238.0911 0.3975 0 K.TLKPGTMSPEAFLQEAQVMK.K**  Deamidation (NQ); 2 Oxidation (M) ([Ions score 56](http://df3/mascot/cgi/peptide_view.pl?file=../data/20121119/F002569.dat&query=1948&hit=1&index=gi%7c00000001&px=1))

**299 - 318 747.2107 2238.6103 2238.0911 0.5191 0 K.TLKPGTMSPEAFLQEAQVMK.K**  Deamidation (NQ); 2 Oxidation (M) ([Ions score 51](http://df3/mascot/cgi/peptide_view.pl?file=../data/20121119/F002569.dat&query=1949&hit=1&index=gi%7c00000001&px=1))

**299 - 318 747.2480 2238.7221 2238.0911 0.6310 0 K.TLKPGTMSPEAFLQEAQVMK.K**  Deamidation (NQ); 2 Oxidation (M) ([Ions score 46](http://df3/mascot/cgi/peptide_view.pl?file=../data/20121119/F002569.dat&query=1950&hit=1&index=gi%7c00000001&px=1))

**299 - 318 747.2777 2238.8113 2238.0911 0.7202 0 K.TLKPGTMSPEAFLQEAQVMK.K**  Deamidation (NQ); 2 Oxidation (M) ([Ions score 58](http://df3/mascot/cgi/peptide_view.pl?file=../data/20121119/F002569.dat&query=1952&hit=1&index=gi%7c00000001&px=1))

**299 - 318 747.3028 2238.8866 2238.0911 0.7954 0 K.TLKPGTMSPEAFLQEAQVMK.K**  Deamidation (NQ); 2 Oxidation (M) ([Ions score 53](http://df3/mascot/cgi/peptide_view.pl?file=../data/20121119/F002569.dat&query=1953&hit=1&index=gi%7c00000001&px=1))

**299 - 318 747.3609 2239.0609 2238.0911 0.9697 0 K.TLKPGTMSPEAFLQEAQVMK.K**  Deamidation (NQ); 2 Oxidation (M) ([Ions score 46](http://df3/mascot/cgi/peptide_view.pl?file=../data/20121119/F002569.dat&query=1955&hit=1&index=gi%7c00000001&px=1))

**299 - 318 747.4995 2239.4765 2238.0911 1.3854 0 K.TLKPGTMSPEAFLQEAQVMK.K**  Deamidation (NQ); 2 Oxidation (M) ([Ions score 11](http://df3/mascot/cgi/peptide_view.pl?file=../data/20121119/F002569.dat&query=1957&hit=1&index=gi%7c00000001&px=1))

**299 - 318 747.6024 2239.7854 2238.0911 1.6943 0 K.TLKPGTMSPEAFLQEAQVMK.K**  Deamidation (NQ); 2 Oxidation (M) ([Ions score 23](http://df3/mascot/cgi/peptide_view.pl?file=../data/20121119/F002569.dat&query=1958&hit=1&index=gi%7c00000001&px=1))

**299 - 319 778.8444 2333.5114 2333.2123 0.2992 1 K.TLKPGTMSPEAFLQEAQVMKK.L**  ([Ions score 63](http://df3/mascot/cgi/peptide_view.pl?file=../data/20121119/F002569.dat&query=2207&hit=1&index=gi%7c00000001&px=1))

**299 - 319 783.5038 2347.4895 2349.2072 -1.7176 1 K.TLKPGTMSPEAFLQEAQVMKK.L**  Oxidation (M) ([Ions score 50](http://df3/mascot/cgi/peptide_view.pl?file=../data/20121119/F002569.dat&query=2245&hit=1&index=gi%7c00000001&px=1))

**299 - 319 784.3940 2350.1603 2349.2072 0.9531 1 K.TLKPGTMSPEAFLQEAQVMKK.L**  Oxidation (M) ([Ions score 60](http://df3/mascot/cgi/peptide_view.pl?file=../data/20121119/F002569.dat&query=2251&hit=1&index=gi%7c00000001&px=1))

**299 - 319 784.5248 2350.5525 2349.2072 1.3453 1 K.TLKPGTMSPEAFLQEAQVMKK.L**  Oxidation (M) ([Ions score 52](http://df3/mascot/cgi/peptide_view.pl?file=../data/20121119/F002569.dat&query=2255&hit=1&index=gi%7c00000001&px=1))

**299 - 319 784.4019 2350.1837 2350.1912 -0.0074 1 K.TLKPGTMSPEAFLQEAQVMKK.L**  Deamidation (NQ); Oxidation (M) ([Ions score 35](http://df3/mascot/cgi/peptide_view.pl?file=../data/20121119/F002569.dat&query=2252&hit=1&index=gi%7c00000001&px=1))

**299 - 319 784.5188 2350.5346 2350.1912 0.3434 1 K.TLKPGTMSPEAFLQEAQVMKK.L**  Deamidation (NQ); Oxidation (M) ([Ions score 64](http://df3/mascot/cgi/peptide_view.pl?file=../data/20121119/F002569.dat&query=2254&hit=1&index=gi%7c00000001&px=1))

**299 - 319 785.0137 2352.0192 2350.1912 1.8280 1 K.TLKPGTMSPEAFLQEAQVMKK.L**  Deamidation (NQ); Oxidation (M) ([Ions score 52](http://df3/mascot/cgi/peptide_view.pl?file=../data/20121119/F002569.dat&query=2257&hit=1&index=gi%7c00000001&px=1))

**299 - 319 788.8229 2363.4470 2365.2021 -1.7551 1 K.TLKPGTMSPEAFLQEAQVMKK.L**  2 Oxidation (M) ([Ions score 74](http://df3/mascot/cgi/peptide_view.pl?file=../data/20121119/F002569.dat&query=2281&hit=1&index=gi%7c00000001&px=1))

**299 - 319 789.5388 2365.5946 2365.2021 0.3925 1 K.TLKPGTMSPEAFLQEAQVMKK.L**  2 Oxidation (M) ([Ions score 54](http://df3/mascot/cgi/peptide_view.pl?file=../data/20121119/F002569.dat&query=2293&hit=1&index=gi%7c00000001&px=1))

**299 - 319 789.8584 2366.5534 2365.2021 1.3513 1 K.TLKPGTMSPEAFLQEAQVMKK.L**  2 Oxidation (M) ([Ions score 58](http://df3/mascot/cgi/peptide_view.pl?file=../data/20121119/F002569.dat&query=2302&hit=1&index=gi%7c00000001&px=1))

**299 - 319 789.8641 2366.5706 2365.2021 1.3685 1 K.TLKPGTMSPEAFLQEAQVMKK.L**  2 Oxidation (M) ([Ions score 56](http://df3/mascot/cgi/peptide_view.pl?file=../data/20121119/F002569.dat&query=2303&hit=1&index=gi%7c00000001&px=1))

**299 - 319 789.8826 2366.6261 2365.2021 1.4240 1 K.TLKPGTMSPEAFLQEAQVMKK.L**  2 Oxidation (M) ([Ions score 60](http://df3/mascot/cgi/peptide_view.pl?file=../data/20121119/F002569.dat&query=2304&hit=1&index=gi%7c00000001&px=1))

**299 - 319 789.9331 2366.7775 2365.2021 1.5754 1 K.TLKPGTMSPEAFLQEAQVMKK.L**  2 Oxidation (M) ([Ions score 64](http://df3/mascot/cgi/peptide_view.pl?file=../data/20121119/F002569.dat&query=2306&hit=1&index=gi%7c00000001&px=1))

**299 - 319 790.2197 2367.6374 2366.1861 1.4513 1 K.TLKPGTMSPEAFLQEAQVMKK.L**  Deamidation (NQ); 2 Oxidation (M) ([Ions score 55](http://df3/mascot/cgi/peptide_view.pl?file=../data/20121119/F002569.dat&query=2307&hit=1&index=gi%7c00000001&px=1))

**347 - 354 446.7255 891.4364 891.5065 -0.0701 0 K.GSLLDFLK.G**  ([Ions score 69](http://df3/mascot/cgi/peptide_view.pl?file=../data/20121119/F002569.dat&query=416&hit=1&index=gi%7c00000001&px=1))

**347 - 354 446.9837 891.9529 891.5065 0.4463 0 K.GSLLDFLK.G**  ([Ions score 15](http://df3/mascot/cgi/peptide_view.pl?file=../data/20121119/F002569.dat&query=418&hit=1&index=gi%7c00000001&px=1))

**347 - 354 446.9986 891.9827 891.5065 0.4762 0 K.GSLLDFLK.G**  ([Ions score 58](http://df3/mascot/cgi/peptide_view.pl?file=../data/20121119/F002569.dat&query=419&hit=1&index=gi%7c00000001&px=1))

**347 - 354 447.0090 892.0035 891.5065 0.4970 0 K.GSLLDFLK.G**  ([Ions score 34](http://df3/mascot/cgi/peptide_view.pl?file=../data/20121119/F002569.dat&query=420&hit=1&index=gi%7c00000001&px=1))

**347 - 354 447.0323 892.0500 891.5065 0.5434 0 K.GSLLDFLK.G**  ([Ions score 25](http://df3/mascot/cgi/peptide_view.pl?file=../data/20121119/F002569.dat&query=422&hit=1&index=gi%7c00000001&px=1))

**347 - 354 447.1049 892.1953 891.5065 0.6888 0 K.GSLLDFLK.G**  ([Ions score 43](http://df3/mascot/cgi/peptide_view.pl?file=../data/20121119/F002569.dat&query=436&hit=1&index=gi%7c00000001&px=1))

**347 - 359 682.5089 1363.0031 1363.7347 -0.7315 1 K.GSLLDFLKGETGK.Y**  ([Ions score 55](http://df3/mascot/cgi/peptide_view.pl?file=../data/20121119/F002569.dat&query=1499&hit=1&index=gi%7c00000001&px=1))

**347 - 359 682.5455 1363.0765 1363.7347 -0.6582 1 K.GSLLDFLKGETGK.Y**  ([Ions score 51](http://df3/mascot/cgi/peptide_view.pl?file=../data/20121119/F002569.dat&query=1500&hit=1&index=gi%7c00000001&px=1))

**347 - 359 455.4129 1363.2170 1363.7347 -0.5177 1 K.GSLLDFLKGETGK.Y**  ([Ions score 29](http://df3/mascot/cgi/peptide_view.pl?file=../data/20121119/F002569.dat&query=477&hit=1&index=gi%7c00000001&px=1))

**347 - 359 682.6859 1363.3573 1363.7347 -0.3774 1 K.GSLLDFLKGETGK.Y**  ([Ions score 94](http://df3/mascot/cgi/peptide_view.pl?file=../data/20121119/F002569.dat&query=1501&hit=1&index=gi%7c00000001&px=1))

**347 - 359 682.7213 1363.4281 1363.7347 -0.3066 1 K.GSLLDFLKGETGK.Y**  ([Ions score 91](http://df3/mascot/cgi/peptide_view.pl?file=../data/20121119/F002569.dat&query=1502&hit=1&index=gi%7c00000001&px=1))

**347 - 359 683.1906 1364.3666 1363.7347 0.6319 1 K.GSLLDFLKGETGK.Y**  ([Ions score 23](http://df3/mascot/cgi/peptide_view.pl?file=../data/20121119/F002569.dat&query=1505&hit=1&index=gi%7c00000001&px=1))

**347 - 359 683.2598 1364.5050 1363.7347 0.7703 1 K.GSLLDFLKGETGK.Y**  ([Ions score 20](http://df3/mascot/cgi/peptide_view.pl?file=../data/20121119/F002569.dat&query=1506&hit=1&index=gi%7c00000001&px=1))

**347 - 359 456.0699 1365.1878 1363.7347 1.4532 1 K.GSLLDFLKGETGK.Y**  ([Ions score 47](http://df3/mascot/cgi/peptide_view.pl?file=../data/20121119/F002569.dat&query=478&hit=1&index=gi%7c00000001&px=1))

**347 - 359 456.0825 1365.2255 1363.7347 1.4909 1 K.GSLLDFLKGETGK.Y**  ([Ions score 37](http://df3/mascot/cgi/peptide_view.pl?file=../data/20121119/F002569.dat&query=479&hit=1&index=gi%7c00000001&px=1))

**347 - 359 456.0972 1365.2697 1363.7347 1.5350 1 K.GSLLDFLKGETGK.Y**  ([Ions score 15](http://df3/mascot/cgi/peptide_view.pl?file=../data/20121119/F002569.dat&query=480&hit=1&index=gi%7c00000001&px=1))

**363 - 382 726.8452 2177.5136 2178.0812 -0.5676 0 R.LPQLVDMAAQIASGMAYVER.M**  Oxidation (M) ([Ions score 60](http://df3/mascot/cgi/peptide_view.pl?file=../data/20121119/F002569.dat&query=1663&hit=1&index=gi%7c00000001&px=1))

**363 - 382 1089.8688 2177.7230 2178.0812 -0.3582 0 R.LPQLVDMAAQIASGMAYVER.M**  Oxidation (M) ([Ions score 125](http://df3/mascot/cgi/peptide_view.pl?file=../data/20121119/F002569.dat&query=2846&hit=1&index=gi%7c00000001&px=1))

**363 - 382 1089.9465 2177.8785 2178.0812 -0.2027 0 R.LPQLVDMAAQIASGMAYVER.M**  Oxidation (M) ([Ions score 139](http://df3/mascot/cgi/peptide_view.pl?file=../data/20121119/F002569.dat&query=2847&hit=1&index=gi%7c00000001&px=1))

**363 - 382 1089.9573 2177.9000 2178.0812 -0.1812 0 R.LPQLVDMAAQIASGMAYVER.M**  Oxidation (M) ([Ions score 123](http://df3/mascot/cgi/peptide_view.pl?file=../data/20121119/F002569.dat&query=2848&hit=1&index=gi%7c00000001&px=1))

**363 - 382 1089.9612 2177.9078 2178.0812 -0.1734 0 R.LPQLVDMAAQIASGMAYVER.M**  Oxidation (M) ([Ions score 142](http://df3/mascot/cgi/peptide_view.pl?file=../data/20121119/F002569.dat&query=2849&hit=1&index=gi%7c00000001&px=1))

**363 - 382 1089.9736 2177.9327 2178.0812 -0.1485 0 R.LPQLVDMAAQIASGMAYVER.M**  Oxidation (M) ([Ions score 141](http://df3/mascot/cgi/peptide_view.pl?file=../data/20121119/F002569.dat&query=2850&hit=1&index=gi%7c00000001&px=1))

**363 - 382 1090.0078 2178.0011 2178.0812 -0.0802 0 R.LPQLVDMAAQIASGMAYVER.M**  Oxidation (M) ([Ions score 158](http://df3/mascot/cgi/peptide_view.pl?file=../data/20121119/F002569.dat&query=2851&hit=1&index=gi%7c00000001&px=1))

**363 - 382 1090.0114 2178.0082 2178.0812 -0.0731 0 R.LPQLVDMAAQIASGMAYVER.M**  Oxidation (M) ([Ions score 146](http://df3/mascot/cgi/peptide_view.pl?file=../data/20121119/F002569.dat&query=2852&hit=1&index=gi%7c00000001&px=1))

**363 - 382 727.0201 2178.0384 2178.0812 -0.0428 0 R.LPQLVDMAAQIASGMAYVER.M**  Oxidation (M) ([Ions score 89](http://df3/mascot/cgi/peptide_view.pl?file=../data/20121119/F002569.dat&query=1664&hit=1&index=gi%7c00000001&px=1))

**363 - 382 727.0599 2178.1580 2178.0812 0.0767 0 R.LPQLVDMAAQIASGMAYVER.M**  Oxidation (M) ([Ions score 51](http://df3/mascot/cgi/peptide_view.pl?file=../data/20121119/F002569.dat&query=1665&hit=1&index=gi%7c00000001&px=1))

**363 - 382 1090.3279 2178.6412 2178.0812 0.5600 0 R.LPQLVDMAAQIASGMAYVER.M**  Oxidation (M) ([Ions score 131](http://df3/mascot/cgi/peptide_view.pl?file=../data/20121119/F002569.dat&query=2853&hit=1&index=gi%7c00000001&px=1))

**363 - 382 727.2584 2178.7533 2178.0812 0.6720 0 R.LPQLVDMAAQIASGMAYVER.M**  Oxidation (M) ([Ions score 82](http://df3/mascot/cgi/peptide_view.pl?file=../data/20121119/F002569.dat&query=1666&hit=1&index=gi%7c00000001&px=1))

**363 - 382 1090.3867 2178.7589 2178.0812 0.6776 0 R.LPQLVDMAAQIASGMAYVER.M**  Oxidation (M) ([Ions score 140](http://df3/mascot/cgi/peptide_view.pl?file=../data/20121119/F002569.dat&query=2854&hit=1&index=gi%7c00000001&px=1))

**363 - 382 727.3008 2178.8805 2178.0812 0.7993 0 R.LPQLVDMAAQIASGMAYVER.M**  Oxidation (M) ([Ions score 85](http://df3/mascot/cgi/peptide_view.pl?file=../data/20121119/F002569.dat&query=1667&hit=1&index=gi%7c00000001&px=1))

**363 - 382 1090.4825 2178.9505 2178.0812 0.8693 0 R.LPQLVDMAAQIASGMAYVER.M**  Oxidation (M) ([Ions score 138](http://df3/mascot/cgi/peptide_view.pl?file=../data/20121119/F002569.dat&query=2855&hit=1&index=gi%7c00000001&px=1))

**363 - 382 727.3544 2179.0413 2178.0812 0.9600 0 R.LPQLVDMAAQIASGMAYVER.M**  Oxidation (M) ([Ions score 73](http://df3/mascot/cgi/peptide_view.pl?file=../data/20121119/F002569.dat&query=1668&hit=1&index=gi%7c00000001&px=1))

**363 - 382 727.3605 2179.0596 2178.0812 0.9784 0 R.LPQLVDMAAQIASGMAYVER.M**  Oxidation (M) ([Ions score 101](http://df3/mascot/cgi/peptide_view.pl?file=../data/20121119/F002569.dat&query=1669&hit=1&index=gi%7c00000001&px=1))

**363 - 382 727.3721 2179.0946 2178.0812 1.0133 0 R.LPQLVDMAAQIASGMAYVER.M**  Oxidation (M) ([Ions score 64](http://df3/mascot/cgi/peptide_view.pl?file=../data/20121119/F002569.dat&query=1670&hit=1&index=gi%7c00000001&px=1))

**363 - 382 727.3861 2179.1365 2178.0812 1.0553 0 R.LPQLVDMAAQIASGMAYVER.M**  Oxidation (M) ([Ions score 84](http://df3/mascot/cgi/peptide_view.pl?file=../data/20121119/F002569.dat&query=1671&hit=1&index=gi%7c00000001&px=1))

**363 - 382 727.5321 2179.5745 2178.0812 1.4932 0 R.LPQLVDMAAQIASGMAYVER.M**  Oxidation (M) ([Ions score 73](http://df3/mascot/cgi/peptide_view.pl?file=../data/20121119/F002569.dat&query=1672&hit=1&index=gi%7c00000001&px=1))

**363 - 382 1090.9622 2179.9098 2178.0812 1.8285 0 R.LPQLVDMAAQIASGMAYVER.M**  Oxidation (M) ([Ions score 125](http://df3/mascot/cgi/peptide_view.pl?file=../data/20121119/F002569.dat&query=2856&hit=1&index=gi%7c00000001&px=1))

**363 - 382 1090.9961 2179.9776 2178.0812 1.8964 0 R.LPQLVDMAAQIASGMAYVER.M**  Oxidation (M) ([Ions score 126](http://df3/mascot/cgi/peptide_view.pl?file=../data/20121119/F002569.dat&query=2857&hit=1&index=gi%7c00000001&px=1))

**363 - 382 727.5482 2179.6226 2179.0652 0.5574 0 R.LPQLVDMAAQIASGMAYVER.M**  Deamidation (NQ); Oxidation (M) ([Ions score 71](http://df3/mascot/cgi/peptide_view.pl?file=../data/20121119/F002569.dat&query=1673&hit=1&index=gi%7c00000001&px=1))

**363 - 382 1091.5259 2181.0372 2179.0652 1.9720 0 R.LPQLVDMAAQIASGMAYVER.M**  Deamidation (NQ); Oxidation (M) ([Ions score 109](http://df3/mascot/cgi/peptide_view.pl?file=../data/20121119/F002569.dat&query=2858&hit=1&index=gi%7c00000001&px=1))

**363 - 382 1091.6580 2181.3014 2180.0493 1.2521 0 R.LPQLVDMAAQIASGMAYVER.M**  2 Deamidation (NQ); Oxidation (M) ([Ions score 102](http://df3/mascot/cgi/peptide_view.pl?file=../data/20121119/F002569.dat&query=2859&hit=1&index=gi%7c00000001&px=1))

**363 - 382 728.2524 2181.7355 2180.0493 1.6862 0 R.LPQLVDMAAQIASGMAYVER.M**  2 Deamidation (NQ); Oxidation (M) ([Ions score 15](http://df3/mascot/cgi/peptide_view.pl?file=../data/20121119/F002569.dat&query=1674&hit=1&index=gi%7c00000001&px=1))

**363 - 382 728.3461 2182.0166 2180.0493 1.9673 0 R.LPQLVDMAAQIASGMAYVER.M**  2 Deamidation (NQ); Oxidation (M) ([Ions score 15](http://df3/mascot/cgi/peptide_view.pl?file=../data/20121119/F002569.dat&query=1677&hit=1&index=gi%7c00000001&px=1))

**363 - 382 731.7423 2192.2051 2194.0762 -1.8711 0 R.LPQLVDMAAQIASGMAYVER.M**  2 Oxidation (M) ([Ions score 40](http://df3/mascot/cgi/peptide_view.pl?file=../data/20121119/F002569.dat&query=1743&hit=1&index=gi%7c00000001&px=1))

**363 - 382 731.7849 2192.3329 2194.0762 -1.7432 0 R.LPQLVDMAAQIASGMAYVER.M**  2 Oxidation (M) ([Ions score 22](http://df3/mascot/cgi/peptide_view.pl?file=../data/20121119/F002569.dat&query=1744&hit=1&index=gi%7c00000001&px=1))

**363 - 382 731.9576 2192.8509 2194.0762 -1.2252 0 R.LPQLVDMAAQIASGMAYVER.M**  2 Oxidation (M) ([Ions score 31](http://df3/mascot/cgi/peptide_view.pl?file=../data/20121119/F002569.dat&query=1745&hit=1&index=gi%7c00000001&px=1))

**363 - 382 731.9883 2192.9430 2194.0762 -1.1331 0 R.LPQLVDMAAQIASGMAYVER.M**  2 Oxidation (M) ([Ions score 60](http://df3/mascot/cgi/peptide_view.pl?file=../data/20121119/F002569.dat&query=1747&hit=1&index=gi%7c00000001&px=1))

**363 - 382 732.0997 2193.2774 2194.0762 -0.7988 0 R.LPQLVDMAAQIASGMAYVER.M**  2 Oxidation (M) ([Ions score 45](http://df3/mascot/cgi/peptide_view.pl?file=../data/20121119/F002569.dat&query=1748&hit=1&index=gi%7c00000001&px=1))

**363 - 382 732.1735 2193.4986 2194.0762 -0.5776 0 R.LPQLVDMAAQIASGMAYVER.M**  2 Oxidation (M) ([Ions score 52](http://df3/mascot/cgi/peptide_view.pl?file=../data/20121119/F002569.dat&query=1749&hit=1&index=gi%7c00000001&px=1))

**363 - 382 732.1747 2193.5022 2194.0762 -0.5739 0 R.LPQLVDMAAQIASGMAYVER.M**  2 Oxidation (M) ([Ions score 53](http://df3/mascot/cgi/peptide_view.pl?file=../data/20121119/F002569.dat&query=1750&hit=1&index=gi%7c00000001&px=1))

**363 - 382 732.1760 2193.5062 2194.0762 -0.5699 0 R.LPQLVDMAAQIASGMAYVER.M**  2 Oxidation (M) ([Ions score 55](http://df3/mascot/cgi/peptide_view.pl?file=../data/20121119/F002569.dat&query=1751&hit=1&index=gi%7c00000001&px=1))

**363 - 382 732.1868 2193.5385 2194.0762 -0.5377 0 R.LPQLVDMAAQIASGMAYVER.M**  2 Oxidation (M) ([Ions score 49](http://df3/mascot/cgi/peptide_view.pl?file=../data/20121119/F002569.dat&query=1752&hit=1&index=gi%7c00000001&px=1))

**363 - 382 732.2188 2193.6344 2194.0762 -0.4417 0 R.LPQLVDMAAQIASGMAYVER.M**  2 Oxidation (M) ([Ions score 35](http://df3/mascot/cgi/peptide_view.pl?file=../data/20121119/F002569.dat&query=1753&hit=1&index=gi%7c00000001&px=1))

**363 - 382 1097.8734 2193.7323 2194.0762 -0.3439 0 R.LPQLVDMAAQIASGMAYVER.M**  2 Oxidation (M) ([Ions score 136](http://df3/mascot/cgi/peptide_view.pl?file=../data/20121119/F002569.dat&query=2867&hit=1&index=gi%7c00000001&px=1))

**363 - 382 732.2632 2193.7677 2194.0762 -0.3084 0 R.LPQLVDMAAQIASGMAYVER.M**  2 Oxidation (M) ([Ions score 64](http://df3/mascot/cgi/peptide_view.pl?file=../data/20121119/F002569.dat&query=1754&hit=1&index=gi%7c00000001&px=1))

**363 - 382 1097.9006 2193.7867 2194.0762 -0.2894 0 R.LPQLVDMAAQIASGMAYVER.M**  2 Oxidation (M) ([Ions score 147](http://df3/mascot/cgi/peptide_view.pl?file=../data/20121119/F002569.dat&query=2868&hit=1&index=gi%7c00000001&px=1))

**363 - 382 732.2734 2193.7985 2194.0762 -0.2777 0 R.LPQLVDMAAQIASGMAYVER.M**  2 Oxidation (M) ([Ions score 46](http://df3/mascot/cgi/peptide_view.pl?file=../data/20121119/F002569.dat&query=1755&hit=1&index=gi%7c00000001&px=1))

**363 - 382 732.3440 2194.0102 2194.0762 -0.0660 0 R.LPQLVDMAAQIASGMAYVER.M**  2 Oxidation (M) ([Ions score 54](http://df3/mascot/cgi/peptide_view.pl?file=../data/20121119/F002569.dat&query=1756&hit=1&index=gi%7c00000001&px=1))

**363 - 382 732.3566 2194.0479 2194.0762 -0.0283 0 R.LPQLVDMAAQIASGMAYVER.M**  2 Oxidation (M) ([Ions score 55](http://df3/mascot/cgi/peptide_view.pl?file=../data/20121119/F002569.dat&query=1757&hit=1&index=gi%7c00000001&px=1))

**363 - 382 1098.0540 2194.0934 2194.0762 0.0172 0 R.LPQLVDMAAQIASGMAYVER.M**  2 Oxidation (M) ([Ions score 145](http://df3/mascot/cgi/peptide_view.pl?file=../data/20121119/F002569.dat&query=2869&hit=1&index=gi%7c00000001&px=1))

**363 - 382 732.4276 2194.2608 2194.0762 0.1847 0 R.LPQLVDMAAQIASGMAYVER.M**  2 Oxidation (M) ([Ions score 61](http://df3/mascot/cgi/peptide_view.pl?file=../data/20121119/F002569.dat&query=1758&hit=1&index=gi%7c00000001&px=1))

**363 - 382 1098.1718 2194.3290 2194.0762 0.2528 0 R.LPQLVDMAAQIASGMAYVER.M**  2 Oxidation (M) ([Ions score 145](http://df3/mascot/cgi/peptide_view.pl?file=../data/20121119/F002569.dat&query=2870&hit=1&index=gi%7c00000001&px=1))

**363 - 382 732.4551 2194.3436 2194.0762 0.2674 0 R.LPQLVDMAAQIASGMAYVER.M**  2 Oxidation (M) ([Ions score 56](http://df3/mascot/cgi/peptide_view.pl?file=../data/20121119/F002569.dat&query=1759&hit=1&index=gi%7c00000001&px=1))

**363 - 382 732.4836 2194.4291 2194.0762 0.3529 0 R.LPQLVDMAAQIASGMAYVER.M**  2 Oxidation (M) ([Ions score 66](http://df3/mascot/cgi/peptide_view.pl?file=../data/20121119/F002569.dat&query=1760&hit=1&index=gi%7c00000001&px=1))

**363 - 382 732.4861 2194.4364 2194.0762 0.3603 0 R.LPQLVDMAAQIASGMAYVER.M**  2 Oxidation (M) ([Ions score 68](http://df3/mascot/cgi/peptide_view.pl?file=../data/20121119/F002569.dat&query=1761&hit=1&index=gi%7c00000001&px=1))

**363 - 382 732.5079 2194.5020 2194.0762 0.4258 0 R.LPQLVDMAAQIASGMAYVER.M**  2 Oxidation (M) ([Ions score 75](http://df3/mascot/cgi/peptide_view.pl?file=../data/20121119/F002569.dat&query=1762&hit=1&index=gi%7c00000001&px=1))

**363 - 382 732.5167 2194.5283 2194.0762 0.4522 0 R.LPQLVDMAAQIASGMAYVER.M**  2 Oxidation (M) ([Ions score 77](http://df3/mascot/cgi/peptide_view.pl?file=../data/20121119/F002569.dat&query=1763&hit=1&index=gi%7c00000001&px=1))

**363 - 382 732.5179 2194.5320 2194.0762 0.4558 0 R.LPQLVDMAAQIASGMAYVER.M**  2 Oxidation (M) ([Ions score 78](http://df3/mascot/cgi/peptide_view.pl?file=../data/20121119/F002569.dat&query=1764&hit=1&index=gi%7c00000001&px=1))

**363 - 382 1098.3643 2194.7140 2194.0762 0.6378 0 R.LPQLVDMAAQIASGMAYVER.M**  2 Oxidation (M) ([Ions score 126](http://df3/mascot/cgi/peptide_view.pl?file=../data/20121119/F002569.dat&query=2871&hit=1&index=gi%7c00000001&px=1))

**363 - 382 732.5851 2194.7334 2194.0762 0.6573 0 R.LPQLVDMAAQIASGMAYVER.M**  2 Oxidation (M) ([Ions score 64](http://df3/mascot/cgi/peptide_view.pl?file=../data/20121119/F002569.dat&query=1766&hit=1&index=gi%7c00000001&px=1))

**363 - 382 1098.3833 2194.7521 2194.0762 0.6759 0 R.LPQLVDMAAQIASGMAYVER.M**  2 Oxidation (M) ([Ions score 136](http://df3/mascot/cgi/peptide_view.pl?file=../data/20121119/F002569.dat&query=2872&hit=1&index=gi%7c00000001&px=1))

**363 - 382 732.5942 2194.7607 2194.0762 0.6845 0 R.LPQLVDMAAQIASGMAYVER.M**  2 Oxidation (M) ([Ions score 58](http://df3/mascot/cgi/peptide_view.pl?file=../data/20121119/F002569.dat&query=1767&hit=1&index=gi%7c00000001&px=1))

**363 - 382 732.6206 2194.8400 2194.0762 0.7638 0 R.LPQLVDMAAQIASGMAYVER.M**  2 Oxidation (M) ([Ions score 66](http://df3/mascot/cgi/peptide_view.pl?file=../data/20121119/F002569.dat&query=1768&hit=1&index=gi%7c00000001&px=1))

**363 - 382 732.6644 2194.9715 2194.0762 0.8953 0 R.LPQLVDMAAQIASGMAYVER.M**  2 Oxidation (M) ([Ions score 56](http://df3/mascot/cgi/peptide_view.pl?file=../data/20121119/F002569.dat&query=1769&hit=1&index=gi%7c00000001&px=1))

**363 - 382 732.7224 2195.1454 2194.0762 1.0693 0 R.LPQLVDMAAQIASGMAYVER.M**  2 Oxidation (M) ([Ions score 66](http://df3/mascot/cgi/peptide_view.pl?file=../data/20121119/F002569.dat&query=1771&hit=1&index=gi%7c00000001&px=1))

**363 - 382 732.7256 2195.1551 2194.0762 1.0790 0 R.LPQLVDMAAQIASGMAYVER.M**  2 Oxidation (M) ([Ions score 70](http://df3/mascot/cgi/peptide_view.pl?file=../data/20121119/F002569.dat&query=1772&hit=1&index=gi%7c00000001&px=1))

**363 - 382 732.7754 2195.3043 2194.0762 1.2282 0 R.LPQLVDMAAQIASGMAYVER.M**  2 Oxidation (M) ([Ions score 85](http://df3/mascot/cgi/peptide_view.pl?file=../data/20121119/F002569.dat&query=1774&hit=1&index=gi%7c00000001&px=1))

**363 - 382 732.7867 2195.3384 2194.0762 1.2622 0 R.LPQLVDMAAQIASGMAYVER.M**  2 Oxidation (M) ([Ions score 77](http://df3/mascot/cgi/peptide_view.pl?file=../data/20121119/F002569.dat&query=1776&hit=1&index=gi%7c00000001&px=1))

**363 - 382 732.7892 2195.3457 2194.0762 1.2696 0 R.LPQLVDMAAQIASGMAYVER.M**  2 Oxidation (M) ([Ions score 65](http://df3/mascot/cgi/peptide_view.pl?file=../data/20121119/F002569.dat&query=1777&hit=1&index=gi%7c00000001&px=1))

**363 - 382 732.8126 2195.4160 2194.0762 1.3399 0 R.LPQLVDMAAQIASGMAYVER.M**  2 Oxidation (M) ([Ions score 74](http://df3/mascot/cgi/peptide_view.pl?file=../data/20121119/F002569.dat&query=1779&hit=1&index=gi%7c00000001&px=1))

**363 - 382 732.8160 2195.4263 2194.0762 1.3501 0 R.LPQLVDMAAQIASGMAYVER.M**  2 Oxidation (M) ([Ions score 88](http://df3/mascot/cgi/peptide_view.pl?file=../data/20121119/F002569.dat&query=1780&hit=1&index=gi%7c00000001&px=1))

**363 - 382 732.8290 2195.4651 2194.0762 1.3890 0 R.LPQLVDMAAQIASGMAYVER.M**  2 Oxidation (M) ([Ions score 69](http://df3/mascot/cgi/peptide_view.pl?file=../data/20121119/F002569.dat&query=1781&hit=1&index=gi%7c00000001&px=1))

**363 - 382 732.8740 2195.6002 2194.0762 1.5241 0 R.LPQLVDMAAQIASGMAYVER.M**  2 Oxidation (M) ([Ions score 62](http://df3/mascot/cgi/peptide_view.pl?file=../data/20121119/F002569.dat&query=1782&hit=1&index=gi%7c00000001&px=1))

**363 - 382 732.8960 2195.6662 2194.0762 1.5900 0 R.LPQLVDMAAQIASGMAYVER.M**  2 Oxidation (M) ([Ions score 61](http://df3/mascot/cgi/peptide_view.pl?file=../data/20121119/F002569.dat&query=1783&hit=1&index=gi%7c00000001&px=1))

**363 - 382 732.9095 2195.7066 2194.0762 1.6305 0 R.LPQLVDMAAQIASGMAYVER.M**  2 Oxidation (M) ([Ions score 56](http://df3/mascot/cgi/peptide_view.pl?file=../data/20121119/F002569.dat&query=1784&hit=1&index=gi%7c00000001&px=1))

**363 - 382 732.9170 2195.7291 2194.0762 1.6530 0 R.LPQLVDMAAQIASGMAYVER.M**  2 Oxidation (M) ([Ions score 40](http://df3/mascot/cgi/peptide_view.pl?file=../data/20121119/F002569.dat&query=1785&hit=1&index=gi%7c00000001&px=1))

**363 - 382 732.9463 2195.8170 2194.0762 1.7409 0 R.LPQLVDMAAQIASGMAYVER.M**  2 Oxidation (M) ([Ions score 69](http://df3/mascot/cgi/peptide_view.pl?file=../data/20121119/F002569.dat&query=1786&hit=1&index=gi%7c00000001&px=1))

**363 - 382 1098.9633 2195.9120 2194.0762 1.8358 0 R.LPQLVDMAAQIASGMAYVER.M**  2 Oxidation (M) ([Ions score 125](http://df3/mascot/cgi/peptide_view.pl?file=../data/20121119/F002569.dat&query=2873&hit=1&index=gi%7c00000001&px=1))

**363 - 382 732.9888 2195.9445 2194.0762 1.8683 0 R.LPQLVDMAAQIASGMAYVER.M**  2 Oxidation (M) ([Ions score 65](http://df3/mascot/cgi/peptide_view.pl?file=../data/20121119/F002569.dat&query=1787&hit=1&index=gi%7c00000001&px=1))

**363 - 382 733.0156 2196.0250 2194.0762 1.9489 0 R.LPQLVDMAAQIASGMAYVER.M**  2 Oxidation (M) ([Ions score 76](http://df3/mascot/cgi/peptide_view.pl?file=../data/20121119/F002569.dat&query=1788&hit=1&index=gi%7c00000001&px=1))

**363 - 382 732.7570 2195.2490 2195.0602 0.1889 0 R.LPQLVDMAAQIASGMAYVER.M**  Deamidation (NQ); 2 Oxidation (M) ([Ions score 71](http://df3/mascot/cgi/peptide_view.pl?file=../data/20121119/F002569.dat&query=1773&hit=1&index=gi%7c00000001&px=1))

**363 - 382 732.7755 2195.3045 2195.0602 0.2444 0 R.LPQLVDMAAQIASGMAYVER.M**  Deamidation (NQ); 2 Oxidation (M) ([Ions score 62](http://df3/mascot/cgi/peptide_view.pl?file=../data/20121119/F002569.dat&query=1775&hit=1&index=gi%7c00000001&px=1))

**363 - 382 732.7954 2195.3644 2195.0602 0.3042 0 R.LPQLVDMAAQIASGMAYVER.M**  Deamidation (NQ); 2 Oxidation (M) ([Ions score 77](http://df3/mascot/cgi/peptide_view.pl?file=../data/20121119/F002569.dat&query=1778&hit=1&index=gi%7c00000001&px=1))

**363 - 382 733.0380 2196.0921 2195.0602 1.0319 0 R.LPQLVDMAAQIASGMAYVER.M**  Deamidation (NQ); 2 Oxidation (M) ([Ions score 69](http://df3/mascot/cgi/peptide_view.pl?file=../data/20121119/F002569.dat&query=1789&hit=1&index=gi%7c00000001&px=1))

**363 - 382 733.0436 2196.1089 2195.0602 1.0487 0 R.LPQLVDMAAQIASGMAYVER.M**  Deamidation (NQ); 2 Oxidation (M) ([Ions score 42](http://df3/mascot/cgi/peptide_view.pl?file=../data/20121119/F002569.dat&query=1790&hit=1&index=gi%7c00000001&px=1))

**363 - 382 733.1802 2196.5189 2195.0602 1.4587 0 R.LPQLVDMAAQIASGMAYVER.M**  Deamidation (NQ); 2 Oxidation (M) ([Ions score 59](http://df3/mascot/cgi/peptide_view.pl?file=../data/20121119/F002569.dat&query=1792&hit=1&index=gi%7c00000001&px=1))

**363 - 382 733.1807 2196.5202 2195.0602 1.4600 0 R.LPQLVDMAAQIASGMAYVER.M**  Deamidation (NQ); 2 Oxidation (M) ([Ions score 65](http://df3/mascot/cgi/peptide_view.pl?file=../data/20121119/F002569.dat&query=1793&hit=1&index=gi%7c00000001&px=1))

**363 - 382 1099.4785 2196.9425 2195.0602 1.8823 0 R.LPQLVDMAAQIASGMAYVER.M**  Deamidation (NQ); 2 Oxidation (M) ([Ions score 116](http://df3/mascot/cgi/peptide_view.pl?file=../data/20121119/F002569.dat&query=2875&hit=1&index=gi%7c00000001&px=1))

**363 - 382 733.3821 2197.1244 2196.0442 1.0802 0 R.LPQLVDMAAQIASGMAYVER.M**  2 Deamidation (NQ); 2 Oxidation (M) ([Ions score 48](http://df3/mascot/cgi/peptide_view.pl?file=../data/20121119/F002569.dat&query=1794&hit=1&index=gi%7c00000001&px=1))

**363 - 382 1099.6676 2197.3207 2196.0442 1.2765 0 R.LPQLVDMAAQIASGMAYVER.M**  2 Deamidation (NQ); 2 Oxidation (M) ([Ions score 88](http://df3/mascot/cgi/peptide_view.pl?file=../data/20121119/F002569.dat&query=2876&hit=1&index=gi%7c00000001&px=1))

**363 - 382 1099.8866 2197.7586 2196.0442 1.7145 0 R.LPQLVDMAAQIASGMAYVER.M**  2 Deamidation (NQ); 2 Oxidation (M) ([Ions score 106](http://df3/mascot/cgi/peptide_view.pl?file=../data/20121119/F002569.dat&query=2878&hit=1&index=gi%7c00000001&px=1))

**389 - 404 595.8918 1784.6535 1783.9614 0.6921 1 R.DLRAANILVGENLVCK.V**  Carbamidomethyl (C) ([Ions score 49](http://df3/mascot/cgi/peptide_view.pl?file=../data/20121119/F002569.dat&query=1149&hit=1&index=gi%7c00000001&px=1))

**392 - 404 449.4088 1345.2047 1344.6958 0.5089 0 R.AANILVGENLVCK.V**  2 Deamidation (NQ) ([Ions score 11](http://df3/mascot/cgi/peptide_view.pl?file=../data/20121119/F002569.dat&query=459&hit=1&index=gi%7c00000001&px=1))

**392 - 404 700.3456 1398.6767 1399.7493 -1.0725 0 R.AANILVGENLVCK.V**  Carbamidomethyl (C) ([Ions score 73](http://df3/mascot/cgi/peptide_view.pl?file=../data/20121119/F002569.dat&query=1568&hit=1&index=gi%7c00000001&px=1))

**392 - 404 467.3723 1399.0950 1399.7493 -0.6542 0 R.AANILVGENLVCK.V**  Carbamidomethyl (C) ([Ions score 15](http://df3/mascot/cgi/peptide_view.pl?file=../data/20121119/F002569.dat&query=584&hit=1&index=gi%7c00000001&px=1))

**392 - 404 700.9343 1399.8541 1399.7493 0.1048 0 R.AANILVGENLVCK.V**  Carbamidomethyl (C) ([Ions score 64](http://df3/mascot/cgi/peptide_view.pl?file=../data/20121119/F002569.dat&query=1570&hit=1&index=gi%7c00000001&px=1))

**392 - 404 700.9647 1399.9148 1399.7493 0.1655 0 R.AANILVGENLVCK.V**  Carbamidomethyl (C) ([Ions score 86](http://df3/mascot/cgi/peptide_view.pl?file=../data/20121119/F002569.dat&query=1571&hit=1&index=gi%7c00000001&px=1))

**392 - 404 701.0250 1400.0355 1399.7493 0.2862 0 R.AANILVGENLVCK.V**  Carbamidomethyl (C) ([Ions score 97](http://df3/mascot/cgi/peptide_view.pl?file=../data/20121119/F002569.dat&query=1572&hit=1&index=gi%7c00000001&px=1))

**392 - 404 701.0995 1400.1844 1399.7493 0.4352 0 R.AANILVGENLVCK.V**  Carbamidomethyl (C) ([Ions score 49](http://df3/mascot/cgi/peptide_view.pl?file=../data/20121119/F002569.dat&query=1573&hit=1&index=gi%7c00000001&px=1))

**392 - 404 701.2979 1400.5813 1399.7493 0.8320 0 R.AANILVGENLVCK.V**  Carbamidomethyl (C) ([Ions score 53](http://df3/mascot/cgi/peptide_view.pl?file=../data/20121119/F002569.dat&query=1574&hit=1&index=gi%7c00000001&px=1))

**392 - 404 701.3491 1400.6837 1399.7493 0.9344 0 R.AANILVGENLVCK.V**  Carbamidomethyl (C) ([Ions score 94](http://df3/mascot/cgi/peptide_view.pl?file=../data/20121119/F002569.dat&query=1575&hit=1&index=gi%7c00000001&px=1))

**392 - 404 467.9330 1400.7773 1399.7493 1.0280 0 R.AANILVGENLVCK.V**  Carbamidomethyl (C) ([Ions score 56](http://df3/mascot/cgi/peptide_view.pl?file=../data/20121119/F002569.dat&query=593&hit=1&index=gi%7c00000001&px=1))

**392 - 404 467.9520 1400.8343 1399.7493 1.0850 0 R.AANILVGENLVCK.V**  Carbamidomethyl (C) ([Ions score 70](http://df3/mascot/cgi/peptide_view.pl?file=../data/20121119/F002569.dat&query=595&hit=1&index=gi%7c00000001&px=1))

**392 - 404 701.6073 1401.2000 1399.7493 1.4508 0 R.AANILVGENLVCK.V**  Carbamidomethyl (C) ([Ions score 77](http://df3/mascot/cgi/peptide_view.pl?file=../data/20121119/F002569.dat&query=1576&hit=1&index=gi%7c00000001&px=1))

**392 - 404 701.6185 1401.2224 1399.7493 1.4731 0 R.AANILVGENLVCK.V**  Carbamidomethyl (C) ([Ions score 79](http://df3/mascot/cgi/peptide_view.pl?file=../data/20121119/F002569.dat&query=1577&hit=1&index=gi%7c00000001&px=1))

**392 - 404 701.8220 1401.6294 1399.7493 1.8801 0 R.AANILVGENLVCK.V**  Carbamidomethyl (C) ([Ions score 19](http://df3/mascot/cgi/peptide_view.pl?file=../data/20121119/F002569.dat&query=1579&hit=1&index=gi%7c00000001&px=1))

**392 - 404 701.8707 1401.7269 1399.7493 1.9776 0 R.AANILVGENLVCK.V**  Carbamidomethyl (C) ([Ions score 35](http://df3/mascot/cgi/peptide_view.pl?file=../data/20121119/F002569.dat&query=1581&hit=1&index=gi%7c00000001&px=1))

**392 - 404 701.8370 1401.6595 1400.7333 0.9262 0 R.AANILVGENLVCK.V**  Carbamidomethyl (C); Deamidation (NQ) ([Ions score 37](http://df3/mascot/cgi/peptide_view.pl?file=../data/20121119/F002569.dat&query=1580&hit=1&index=gi%7c00000001&px=1))

**392 - 404 702.0137 1402.0129 1400.7333 1.2796 0 R.AANILVGENLVCK.V**  Carbamidomethyl (C); Deamidation (NQ) ([Ions score 73](http://df3/mascot/cgi/peptide_view.pl?file=../data/20121119/F002569.dat&query=1582&hit=1&index=gi%7c00000001&px=1))

**392 - 404 702.0282 1402.0418 1400.7333 1.3086 0 R.AANILVGENLVCK.V**  Carbamidomethyl (C); Deamidation (NQ) ([Ions score 15](http://df3/mascot/cgi/peptide_view.pl?file=../data/20121119/F002569.dat&query=1583&hit=1&index=gi%7c00000001&px=1))

**392 - 404 702.1246 1402.2346 1400.7333 1.5013 0 R.AANILVGENLVCK.V**  Carbamidomethyl (C); Deamidation (NQ) ([Ions score 33](http://df3/mascot/cgi/peptide_view.pl?file=../data/20121119/F002569.dat&query=1587&hit=1&index=gi%7c00000001&px=1))

**392 - 404 702.2091 1402.4037 1400.7333 1.6704 0 R.AANILVGENLVCK.V**  Carbamidomethyl (C); Deamidation (NQ) ([Ions score 73](http://df3/mascot/cgi/peptide_view.pl?file=../data/20121119/F002569.dat&query=1589&hit=1&index=gi%7c00000001&px=1))

**392 - 412 744.0626 2229.1659 2229.1939 -0.0280 1 R.AANILVGENLVCKVADFGLAR.L**  Carbamidomethyl (C) ([Ions score 44](http://df3/mascot/cgi/peptide_view.pl?file=../data/20121119/F002569.dat&query=1915&hit=1&index=gi%7c00000001&px=1))

**392 - 412 744.5799 2230.7179 2229.1939 1.5240 1 R.AANILVGENLVCKVADFGLAR.L**  Carbamidomethyl (C) ([Ions score 54](http://df3/mascot/cgi/peptide_view.pl?file=../data/20121119/F002569.dat&query=1919&hit=1&index=gi%7c00000001&px=1))

**405 - 412 424.9315 847.8485 847.4552 0.3933 0 K.VADFGLAR.L**  ([Ions score 75](http://df3/mascot/cgi/peptide_view.pl?file=../data/20121119/F002569.dat&query=334&hit=1&index=gi%7c00000001&px=1))

**405 - 412 425.1493 848.2840 847.4552 0.8289 0 K.VADFGLAR.L**  ([Ions score 73](http://df3/mascot/cgi/peptide_view.pl?file=../data/20121119/F002569.dat&query=335&hit=1&index=gi%7c00000001&px=1))

**413 - 422 612.0584 1222.1023 1222.5829 -0.4807 0 R.LIEDNEYTAR.Q**  ([Ions score 44](http://df3/mascot/cgi/peptide_view.pl?file=../data/20121119/F002569.dat&query=1233&hit=1&index=gi%7c00000001&px=1))

**413 - 422 612.4805 1222.9465 1222.5829 0.3636 0 R.LIEDNEYTAR.Q**  ([Ions score 48](http://df3/mascot/cgi/peptide_view.pl?file=../data/20121119/F002569.dat&query=1234&hit=1&index=gi%7c00000001&px=1))

**413 - 426 537.2448 1608.7124 1607.7791 0.9334 1 R.LIEDNEYTARQGAK.F**  Deamidation (NQ) ([Ions score 16](http://df3/mascot/cgi/peptide_view.pl?file=../data/20121119/F002569.dat&query=892&hit=1&index=gi%7c00000001&px=1))

**423 - 430 444.8148 887.6150 888.5069 -0.8918 1 R.QGAKFPIK.W**  Deamidation (NQ) ([Ions score 12](http://df3/mascot/cgi/peptide_view.pl?file=../data/20121119/F002569.dat&query=406&hit=1&index=gi%7c00000001&px=1))

**427 - 441 860.5399 1719.0652 1718.9144 0.1508 1 K.FPIKWTAPEAALYGR.F**  ([Ions score 99](http://df3/mascot/cgi/peptide_view.pl?file=../data/20121119/F002569.dat&query=2522&hit=1&index=gi%7c00000001&px=1))

**427 - 441 574.0848 1719.2327 1718.9144 0.3183 1 K.FPIKWTAPEAALYGR.F**  ([Ions score 47](http://df3/mascot/cgi/peptide_view.pl?file=../data/20121119/F002569.dat&query=1015&hit=1&index=gi%7c00000001&px=1))

**427 - 441 574.2278 1719.6615 1718.9144 0.7472 1 K.FPIKWTAPEAALYGR.F**  ([Ions score 60](http://df3/mascot/cgi/peptide_view.pl?file=../data/20121119/F002569.dat&query=1016&hit=1&index=gi%7c00000001&px=1))

**427 - 441 860.8983 1719.7821 1718.9144 0.8677 1 K.FPIKWTAPEAALYGR.F**  ([Ions score 78](http://df3/mascot/cgi/peptide_view.pl?file=../data/20121119/F002569.dat&query=2524&hit=1&index=gi%7c00000001&px=1))

**427 - 441 574.3997 1720.1771 1718.9144 1.2628 1 K.FPIKWTAPEAALYGR.F**  ([Ions score 78](http://df3/mascot/cgi/peptide_view.pl?file=../data/20121119/F002569.dat&query=1017&hit=1&index=gi%7c00000001&px=1))

**427 - 441 574.4329 1720.2769 1718.9144 1.3626 1 K.FPIKWTAPEAALYGR.F**  ([Ions score 64](http://df3/mascot/cgi/peptide_view.pl?file=../data/20121119/F002569.dat&query=1018&hit=1&index=gi%7c00000001&px=1))

**427 - 441 574.4470 1720.3192 1718.9144 1.4049 1 K.FPIKWTAPEAALYGR.F**  ([Ions score 62](http://df3/mascot/cgi/peptide_view.pl?file=../data/20121119/F002569.dat&query=1019&hit=1&index=gi%7c00000001&px=1))

**442 - 461 767.2318 2298.6736 2298.2511 0.4225 1 R.FTIKSDVWSFGILLTELTTK.G**  ([Ions score 11](http://df3/mascot/cgi/peptide_view.pl?file=../data/20121119/F002569.dat&query=2109&hit=1&index=gi%7c00000001&px=1))

**462 - 472 623.2428 1244.4710 1244.6447 -0.1737 1 K.GRVPYPGMVNR.E**  ([Ions score 50](http://df3/mascot/cgi/peptide_view.pl?file=../data/20121119/F002569.dat&query=1286&hit=1&index=gi%7c00000001&px=1))

**462 - 472 416.2553 1245.7441 1244.6447 1.0994 1 K.GRVPYPGMVNR.E**  ([Ions score 51](http://df3/mascot/cgi/peptide_view.pl?file=../data/20121119/F002569.dat&query=299&hit=1&index=gi%7c00000001&px=1))

**462 - 472 416.2784 1245.8133 1244.6447 1.1686 1 K.GRVPYPGMVNR.E**  ([Ions score 46](http://df3/mascot/cgi/peptide_view.pl?file=../data/20121119/F002569.dat&query=300&hit=1&index=gi%7c00000001&px=1))

**462 - 472 416.3375 1245.9906 1244.6447 1.3458 1 K.GRVPYPGMVNR.E**  ([Ions score 44](http://df3/mascot/cgi/peptide_view.pl?file=../data/20121119/F002569.dat&query=301&hit=1&index=gi%7c00000001&px=1))

**462 - 472 624.1410 1246.2674 1244.6447 1.6227 1 K.GRVPYPGMVNR.E**  ([Ions score 46](http://df3/mascot/cgi/peptide_view.pl?file=../data/20121119/F002569.dat&query=1290&hit=1&index=gi%7c00000001&px=1))

**462 - 472 421.1348 1260.3827 1260.6397 -0.2570 1 K.GRVPYPGMVNR.E**  Oxidation (M) ([Ions score 50](http://df3/mascot/cgi/peptide_view.pl?file=../data/20121119/F002569.dat&query=321&hit=1&index=gi%7c00000001&px=1))

**462 - 472 631.4047 1260.7948 1260.6397 0.1551 1 K.GRVPYPGMVNR.E**  Oxidation (M) ([Ions score 26](http://df3/mascot/cgi/peptide_view.pl?file=../data/20121119/F002569.dat&query=1326&hit=1&index=gi%7c00000001&px=1))

**462 - 472 631.4521 1260.8897 1260.6397 0.2501 1 K.GRVPYPGMVNR.E**  Oxidation (M) ([Ions score 16](http://df3/mascot/cgi/peptide_view.pl?file=../data/20121119/F002569.dat&query=1328&hit=1&index=gi%7c00000001&px=1))

**462 - 472 421.5079 1261.5019 1260.6397 0.8622 1 K.GRVPYPGMVNR.E**  Oxidation (M) ([Ions score 37](http://df3/mascot/cgi/peptide_view.pl?file=../data/20121119/F002569.dat&query=322&hit=1&index=gi%7c00000001&px=1))

**462 - 472 421.5272 1261.5598 1260.6397 0.9202 1 K.GRVPYPGMVNR.E**  Oxidation (M) ([Ions score 13](http://df3/mascot/cgi/peptide_view.pl?file=../data/20121119/F002569.dat&query=323&hit=1&index=gi%7c00000001&px=1))

**462 - 472 421.6211 1261.8414 1260.6397 1.2017 1 K.GRVPYPGMVNR.E**  Oxidation (M) ([Ions score 51](http://df3/mascot/cgi/peptide_view.pl?file=../data/20121119/F002569.dat&query=325&hit=1&index=gi%7c00000001&px=1))

**462 - 472 421.9912 1262.9516 1261.6237 1.3280 1 K.GRVPYPGMVNR.E**  Deamidation (NQ); Oxidation (M) ([Ions score 39](http://df3/mascot/cgi/peptide_view.pl?file=../data/20121119/F002569.dat&query=326&hit=1&index=gi%7c00000001&px=1))

**462 - 472 632.5048 1262.9950 1261.6237 1.3713 1 K.GRVPYPGMVNR.E**  Deamidation (NQ); Oxidation (M) ([Ions score 20](http://df3/mascot/cgi/peptide_view.pl?file=../data/20121119/F002569.dat&query=1330&hit=1&index=gi%7c00000001&px=1))

**464 - 472 517.2426 1032.4706 1031.5222 0.9484 0 R.VPYPGMVNR.E**  ([Ions score 22](http://df3/mascot/cgi/peptide_view.pl?file=../data/20121119/F002569.dat&query=772&hit=1&index=gi%7c00000001&px=1))

**464 - 472 517.5664 1033.1183 1031.5222 1.5961 0 R.VPYPGMVNR.E**  ([Ions score 48](http://df3/mascot/cgi/peptide_view.pl?file=../data/20121119/F002569.dat&query=775&hit=1&index=gi%7c00000001&px=1))

**464 - 472 524.8906 1047.7666 1047.5171 0.2495 0 R.VPYPGMVNR.E**  Oxidation (M) ([Ions score 15](http://df3/mascot/cgi/peptide_view.pl?file=../data/20121119/F002569.dat&query=841&hit=1&index=gi%7c00000001&px=1))

**464 - 472 524.9754 1047.9363 1047.5171 0.4191 0 R.VPYPGMVNR.E**  Oxidation (M) ([Ions score 32](http://df3/mascot/cgi/peptide_view.pl?file=../data/20121119/F002569.dat&query=842&hit=1&index=gi%7c00000001&px=1))

**464 - 472 525.0139 1048.0133 1047.5171 0.4962 0 R.VPYPGMVNR.E**  Oxidation (M) ([Ions score 12](http://df3/mascot/cgi/peptide_view.pl?file=../data/20121119/F002569.dat&query=843&hit=1&index=gi%7c00000001&px=1))

**464 - 480 667.6910 2000.0513 2000.0149 0.0364 1 R.VPYPGMVNREVLDQVER.G**  ([Ions score 36](http://df3/mascot/cgi/peptide_view.pl?file=../data/20121119/F002569.dat&query=1444&hit=1&index=gi%7c00000001&px=1))

**464 - 480 673.3644 2017.0715 2016.0098 1.0617 1 R.VPYPGMVNREVLDQVER.G**  Oxidation (M) ([Ions score 27](http://df3/mascot/cgi/peptide_view.pl?file=../data/20121119/F002569.dat&query=1472&hit=1&index=gi%7c00000001&px=1))

**464 - 480 673.4668 2017.3786 2016.0098 1.3688 1 R.VPYPGMVNREVLDQVER.G**  Oxidation (M) ([Ions score 33](http://df3/mascot/cgi/peptide_view.pl?file=../data/20121119/F002569.dat&query=1473&hit=1&index=gi%7c00000001&px=1))

**464 - 480 673.6189 2017.8349 2016.0098 1.8251 1 R.VPYPGMVNREVLDQVER.G**  Oxidation (M) ([Ions score 34](http://df3/mascot/cgi/peptide_view.pl?file=../data/20121119/F002569.dat&query=1475&hit=1&index=gi%7c00000001&px=1))

**484 - 503 869.1798 2604.5176 2603.0194 1.4982 0 R.MPCPPECPESLHDLMCQCWR.K**  4 Carbamidomethyl (C); Deamidation (NQ) ([Ions score 60](http://df3/mascot/cgi/peptide_view.pl?file=../data/20121119/F002569.dat&query=2549&hit=1&index=gi%7c00000001&px=1))

**484 - 503 873.1738 2616.4997 2618.0303 -1.5306 0 R.MPCPPECPESLHDLMCQCWR.K**  4 Carbamidomethyl (C); Oxidation (M) ([Ions score 52](http://df3/mascot/cgi/peptide_view.pl?file=../data/20121119/F002569.dat&query=2563&hit=1&index=gi%7c00000001&px=1))

**484 - 503 873.7039 2618.0897 2618.0303 0.0594 0 R.MPCPPECPESLHDLMCQCWR.K**  4 Carbamidomethyl (C); Oxidation (M) ([Ions score 61](http://df3/mascot/cgi/peptide_view.pl?file=../data/20121119/F002569.dat&query=2566&hit=1&index=gi%7c00000001&px=1))

**484 - 503 873.7200 2618.1381 2618.0303 0.1078 0 R.MPCPPECPESLHDLMCQCWR.K**  4 Carbamidomethyl (C); Oxidation (M) ([Ions score 44](http://df3/mascot/cgi/peptide_view.pl?file=../data/20121119/F002569.dat&query=2567&hit=1&index=gi%7c00000001&px=1))

**484 - 503 874.0563 2619.1470 2618.0303 1.1167 0 R.MPCPPECPESLHDLMCQCWR.K**  4 Carbamidomethyl (C); Oxidation (M) ([Ions score 25](http://df3/mascot/cgi/peptide_view.pl?file=../data/20121119/F002569.dat&query=2570&hit=1&index=gi%7c00000001&px=1))

**484 - 503 874.2067 2619.5982 2618.0303 1.5679 0 R.MPCPPECPESLHDLMCQCWR.K**  4 Carbamidomethyl (C); Oxidation (M) ([Ions score 11](http://df3/mascot/cgi/peptide_view.pl?file=../data/20121119/F002569.dat&query=2586&hit=1&index=gi%7c00000001&px=1))

**484 - 503 874.4757 2620.4053 2619.0143 1.3910 0 R.MPCPPECPESLHDLMCQCWR.K**  4 Carbamidomethyl (C); Deamidation (NQ); Oxidation (M) ([Ions score 31](http://df3/mascot/cgi/peptide_view.pl?file=../data/20121119/F002569.dat&query=2609&hit=1&index=gi%7c00000001&px=1))

**484 - 503 874.5104 2620.5093 2619.0143 1.4950 0 R.MPCPPECPESLHDLMCQCWR.K**  4 Carbamidomethyl (C); Deamidation (NQ); Oxidation (M) ([Ions score 13](http://df3/mascot/cgi/peptide_view.pl?file=../data/20121119/F002569.dat&query=2612&hit=1&index=gi%7c00000001&px=1))

**484 - 503 874.6229 2620.8469 2619.0143 1.8326 0 R.MPCPPECPESLHDLMCQCWR.K**  4 Carbamidomethyl (C); Deamidation (NQ); Oxidation (M) ([Ions score 36](http://df3/mascot/cgi/peptide_view.pl?file=../data/20121119/F002569.dat&query=2618&hit=1&index=gi%7c00000001&px=1))

**484 - 503 876.0945 2625.2616 2626.1940 -0.9323 0 R.MPCPPECPESLHDLMCQCWR.K**  2 Carbamidomethyl (C); 2 Oxidation (M); 2 HNE (H C) ([Ions score 14](http://df3/mascot/cgi/peptide_view.pl?file=../data/20121119/F002569.dat&query=2628&hit=1&index=gi%7c00000001&px=1))

**484 - 503 878.8530 2633.5373 2634.0252 -0.4880 0 R.MPCPPECPESLHDLMCQCWR.K**  4 Carbamidomethyl (C); 2 Oxidation (M) ([Ions score 32](http://df3/mascot/cgi/peptide_view.pl?file=../data/20121119/F002569.dat&query=2647&hit=1&index=gi%7c00000001&px=1))

**484 - 503 879.3771 2635.1094 2634.0252 1.0842 0 R.MPCPPECPESLHDLMCQCWR.K**  4 Carbamidomethyl (C); 2 Oxidation (M) ([Ions score 34](http://df3/mascot/cgi/peptide_view.pl?file=../data/20121119/F002569.dat&query=2655&hit=1&index=gi%7c00000001&px=1))

**484 - 503 879.5692 2635.6858 2634.0252 1.6606 0 R.MPCPPECPESLHDLMCQCWR.K**  4 Carbamidomethyl (C); 2 Oxidation (M) ([Ions score 43](http://df3/mascot/cgi/peptide_view.pl?file=../data/20121119/F002569.dat&query=2656&hit=1&index=gi%7c00000001&px=1))

**484 - 503 879.8784 2636.6134 2635.0092 1.6042 0 R.MPCPPECPESLHDLMCQCWR.K**  4 Carbamidomethyl (C); Deamidation (NQ); 2 Oxidation (M) ([Ions score 33](http://df3/mascot/cgi/peptide_view.pl?file=../data/20121119/F002569.dat&query=2661&hit=1&index=gi%7c00000001&px=1))

**484 - 504 879.0406 2634.1001 2633.0663 1.0338 1 R.MPCPPECPESLHDLMCQCWRK.E**  2 Carbamidomethyl (C); Deamidation (NQ); Oxidation (M) ([Ions score 17](http://df3/mascot/cgi/peptide_view.pl?file=../data/20121119/F002569.dat&query=2649&hit=1&index=gi%7c00000001&px=1))

**484 - 504 879.1395 2634.3967 2633.0663 1.3304 1 R.MPCPPECPESLHDLMCQCWRK.E**  2 Carbamidomethyl (C); Deamidation (NQ); Oxidation (M) ([Ions score 11](http://df3/mascot/cgi/peptide_view.pl?file=../data/20121119/F002569.dat&query=2652&hit=1&index=gi%7c00000001&px=1))

**484 - 504 883.6641 2647.9704 2649.0612 -1.0909 1 R.MPCPPECPESLHDLMCQCWRK.E**  2 Carbamidomethyl (C); Deamidation (NQ); 2 Oxidation (M) ([Ions score 10](http://df3/mascot/cgi/peptide_view.pl?file=../data/20121119/F002569.dat&query=2684&hit=1&index=gi%7c00000001&px=1))

**484 - 504 884.5068 2650.4987 2649.0612 1.4374 1 R.MPCPPECPESLHDLMCQCWRK.E**  2 Carbamidomethyl (C); Deamidation (NQ); 2 Oxidation (M) ([Ions score 36](http://df3/mascot/cgi/peptide_view.pl?file=../data/20121119/F002569.dat&query=2691&hit=1&index=gi%7c00000001&px=1))


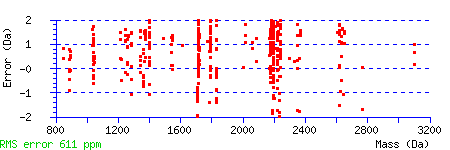


양식의 맨 아래

| **Mascot:**  <http://www.matrixscience.com/> |
| --- |
