# Supplementary material for: Src Tyrosine Kinase Activation by 4-Hydroxynonenal Upregulates p38, ERK/AP-1 Signaling and COX-2 Expression in YPEN-1 Cells
Source: PLoS One. 2015 Oct 14;10(10):e0129244. doi: 10.1371/journal.pone.0129244 (PMC4605600; doi:10.1371/journal.pone.0129244)
Supplement: S2 Data — (DOCX) [file pone.0129244.s002.docx]

**
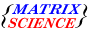
Mascot Search Results**

**User : admin**

**Email : mina@proteometech.com**

**Search title : 12.11.19 Busan JEJ**

**MS data file : JEJ.xml**

**Database : mina50 20121115 (1 sequences; 536 residues)**

**Timestamp : 19 Nov 2012 at 06:34:04 GMT**

**Significant hits:** [**gi|00000001**](http://df3/mascot/cgi/master_results.pl?file=..%2Fdata%2F20121119%2FF002569.dat&REPTYPE=peptide&_sigthreshold=0.05&REPORT=20&_mudpit=1&_ignoreionsscorebelow=10&_showsubsets=0&_showpopups=TRUE&_sortunassigned=scoredown&rbrchkbox=on&_requireboldred=1#Hit1) **Busan JEJ**

**Probability Based Mowse Score**

Ions score is -10*Log(P), where P is the probability that the observed match is a random event.
Individual ions scores > 6 indicate identity or extensive homology (p<0.05).
Protein scores are derived from ions scores as a non-probabilistic basis for ranking protein hits.


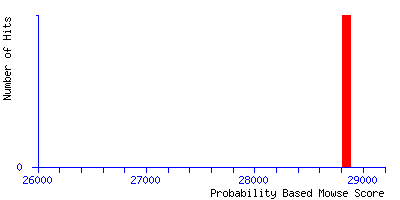


양식의 맨 위

**Peptide Summary Report**

|  |  |  | [Help](http://df3/mascot/help/results_help.html#FORMAT) |
| --- | --- | --- | --- |
|  | Significance threshold p< | Max. number of hits |  |
|  | Standard scoring  MudPIT scoring | Ions score cut-off | Show sub-sets |
|  | Show pop-ups  Suppress pop-ups | Sort unassigned | Require bold red |

양식의 맨 아래

양식의 맨 위

**Error tolerant**

| **1.** | [gi|00000001](http://df3/mascot/cgi/protein_view.pl?file=../data/20121119/F002569.dat&hit=gi%7c00000001&px=1&protscore=28842.3238398357&_mudpit=1&_ignoreionsscorebelow=10)       **Mass:** 59797    **Score:** 28842  **Queries matched:** 487 |
| --- | --- |
|  | Busan JEJ |

|  | Check to include this hit in error tolerant search or archive report |
| --- | --- |
|  |  |

|  | **Query** | **Observed** | **Mr(expt)** | **Mr(calc)** | **Delta** | **Miss** | **Score** | **Expect** | **Rank** | **Peptide** |
| --- | --- | --- | --- | --- | --- | --- | --- | --- | --- | --- |
|  | [334](http://df3/mascot/cgi/peptide_view.pl?file=../data/20121119/F002569.dat&query=334&hit=1&index=gi%7c00000001&px=1) | **424.9315** | **847.8485** | **847.4552** | **0.3933** | **0** | **75** | **4.3e-009** | **1** | **K.VADFGLAR.L** |
|  | [335](http://df3/mascot/cgi/peptide_view.pl?file=../data/20121119/F002569.dat&query=335&hit=1&index=gi%7c00000001&px=1) | **425.1493** | **848.2840** | **847.4552** | **0.8289** | **0** | **(73)** | **7.9e-009** | **1** | **K.VADFGLAR.L** |
|  | [399](http://df3/mascot/cgi/peptide_view.pl?file=../data/20121119/F002569.dat&query=399&hit=1&index=gi%7c00000001&px=1) | **443.4898** | **884.9651** | **885.4344** | **-0.4694** | **0** | **(16)** | **0.0013** | **1** | **K.DAWEIPR.E** |
|  | [400](http://df3/mascot/cgi/peptide_view.pl?file=../data/20121119/F002569.dat&query=400&hit=1&index=gi%7c00000001&px=1) | **443.6636** | **885.3126** | **885.4344** | **-0.1219** | **0** | **(32)** | **3.4e-005** | **1** | **K.DAWEIPR.E** |
|  | [401](http://df3/mascot/cgi/peptide_view.pl?file=../data/20121119/F002569.dat&query=401&hit=1&index=gi%7c00000001&px=1) | **443.6965** | **885.3785** | **885.4344** | **-0.0560** | **0** | **(33)** | **2.5e-005** | **1** | **K.DAWEIPR.E** |
|  | [403](http://df3/mascot/cgi/peptide_view.pl?file=../data/20121119/F002569.dat&query=403&hit=1&index=gi%7c00000001&px=1) | **443.9162** | **885.8178** | **885.4344** | **0.3834** | **0** | **51** | **1.3e-006** | **1** | **K.DAWEIPR.E** |
|  | [405](http://df3/mascot/cgi/peptide_view.pl?file=../data/20121119/F002569.dat&query=405&hit=1&index=gi%7c00000001&px=1) | **444.1174** | **886.2202** | **885.4344** | **0.7857** | **0** | **(35)** | **4.3e-005** | **1** | **K.DAWEIPR.E** |
|  | [406](http://df3/mascot/cgi/peptide_view.pl?file=../data/20121119/F002569.dat&query=406&hit=1&index=gi%7c00000001&px=1) | **444.8148** | **887.6150** | **888.5069** | **-0.8918** | **1** | **12** | **0.014** | **1** | **R.QGAKFPIK.W + Deamidation (NQ)** |
|  | [416](http://df3/mascot/cgi/peptide_view.pl?file=../data/20121119/F002569.dat&query=416&hit=1&index=gi%7c00000001&px=1) | **446.7255** | **891.4364** | **891.5065** | **-0.0701** | **0** | **69** | **6.1e-009** | **1** | **K.GSLLDFLK.G** |
|  | [418](http://df3/mascot/cgi/peptide_view.pl?file=../data/20121119/F002569.dat&query=418&hit=1&index=gi%7c00000001&px=1) | **446.9837** | **891.9529** | **891.5065** | **0.4463** | **0** | **(15)** | **0.0015** | **1** | **K.GSLLDFLK.G** |
|  | [419](http://df3/mascot/cgi/peptide_view.pl?file=../data/20121119/F002569.dat&query=419&hit=1&index=gi%7c00000001&px=1) | **446.9986** | **891.9827** | **891.5065** | **0.4762** | **0** | **(58)** | **7.7e-008** | **1** | **K.GSLLDFLK.G** |
|  | [420](http://df3/mascot/cgi/peptide_view.pl?file=../data/20121119/F002569.dat&query=420&hit=1&index=gi%7c00000001&px=1) | **447.0090** | **892.0035** | **891.5065** | **0.4970** | **0** | **(34)** | **2.1e-005** | **1** | **K.GSLLDFLK.G** |
|  | [422](http://df3/mascot/cgi/peptide_view.pl?file=../data/20121119/F002569.dat&query=422&hit=1&index=gi%7c00000001&px=1) | **447.0323** | **892.0500** | **891.5065** | **0.5434** | **0** | **(25)** | **0.00017** | **1** | **K.GSLLDFLK.G** |
|  | [436](http://df3/mascot/cgi/peptide_view.pl?file=../data/20121119/F002569.dat&query=436&hit=1&index=gi%7c00000001&px=1) | **447.1049** | **892.1953** | **891.5065** | **0.6888** | **0** | **(43)** | **2.7e-006** | **1** | **K.GSLLDFLK.G** |
|  | [772](http://df3/mascot/cgi/peptide_view.pl?file=../data/20121119/F002569.dat&query=772&hit=1&index=gi%7c00000001&px=1) | **517.2426** | **1032.4706** | **1031.5222** | **0.9484** | **0** | **(22)** | **0.00067** | **1** | **R.VPYPGMVNR.E** |
|  | [775](http://df3/mascot/cgi/peptide_view.pl?file=../data/20121119/F002569.dat&query=775&hit=1&index=gi%7c00000001&px=1) | **517.5664** | **1033.1183** | **1031.5222** | **1.5961** | **0** | **48** | **1.4e-006** | **1** | **R.VPYPGMVNR.E** |
|  | [803](http://df3/mascot/cgi/peptide_view.pl?file=../data/20121119/F002569.dat&query=803&hit=1&index=gi%7c00000001&px=1) | **519.9821** | **1037.9496** | **1038.5822** | **-0.6326** | **0** | **(33)** | **5e-005** | **1** | **R.LLLNAENPR.G** |
|  | [804](http://df3/mascot/cgi/peptide_view.pl?file=../data/20121119/F002569.dat&query=804&hit=1&index=gi%7c00000001&px=1) | **519.9973** | **1037.9801** | **1038.5822** | **-0.6021** | **0** | **(23)** | **0.00054** | **1** | **R.LLLNAENPR.G** |
|  | [805](http://df3/mascot/cgi/peptide_view.pl?file=../data/20121119/F002569.dat&query=805&hit=1&index=gi%7c00000001&px=1) | **520.0958** | **1038.1771** | **1038.5822** | **-0.4051** | **0** | **(63)** | **4.9e-008** | **1** | **R.LLLNAENPR.G** |
|  | [807](http://df3/mascot/cgi/peptide_view.pl?file=../data/20121119/F002569.dat&query=807&hit=1&index=gi%7c00000001&px=1) | **520.1521** | **1038.2896** | **1038.5822** | **-0.2925** | **0** | **(62)** | **6.4e-008** | **1** | **R.LLLNAENPR.G** |
|  | [808](http://df3/mascot/cgi/peptide_view.pl?file=../data/20121119/F002569.dat&query=808&hit=1&index=gi%7c00000001&px=1) | **520.2513** | **1038.4881** | **1038.5822** | **-0.0940** | **0** | **(69)** | **1.2e-008** | **1** | **R.LLLNAENPR.G** |
|  | [809](http://df3/mascot/cgi/peptide_view.pl?file=../data/20121119/F002569.dat&query=809&hit=1&index=gi%7c00000001&px=1) | **520.2668** | **1038.5191** | **1038.5822** | **-0.0630** | **0** | **70** | **9.9e-009** | **1** | **R.LLLNAENPR.G** |
|  | [810](http://df3/mascot/cgi/peptide_view.pl?file=../data/20121119/F002569.dat&query=810&hit=1&index=gi%7c00000001&px=1) | **520.3851** | **1038.7557** | **1038.5822** | **0.1736** | **0** | **(44)** | **5.8e-006** | **1** | **R.LLLNAENPR.G** |
|  | [811](http://df3/mascot/cgi/peptide_view.pl?file=../data/20121119/F002569.dat&query=811&hit=1&index=gi%7c00000001&px=1) | **520.4672** | **1038.9198** | **1038.5822** | **0.3376** | **0** | **(41)** | **1.3e-005** | **1** | **R.LLLNAENPR.G** |
|  | [812](http://df3/mascot/cgi/peptide_view.pl?file=../data/20121119/F002569.dat&query=812&hit=1&index=gi%7c00000001&px=1) | **520.4690** | **1038.9234** | **1038.5822** | **0.3413** | **0** | **(57)** | **2.9e-007** | **1** | **R.LLLNAENPR.G** |
|  | [813](http://df3/mascot/cgi/peptide_view.pl?file=../data/20121119/F002569.dat&query=813&hit=1&index=gi%7c00000001&px=1) | **520.4906** | **1038.9667** | **1038.5822** | **0.3845** | **0** | **(13)** | **0.0069** | **1** | **R.LLLNAENPR.G** |
|  | [814](http://df3/mascot/cgi/peptide_view.pl?file=../data/20121119/F002569.dat&query=814&hit=1&index=gi%7c00000001&px=1) | **520.5491** | **1039.0837** | **1038.5822** | **0.5016** | **0** | **(51)** | **1.1e-006** | **1** | **R.LLLNAENPR.G** |
|  | [815](http://df3/mascot/cgi/peptide_view.pl?file=../data/20121119/F002569.dat&query=815&hit=1&index=gi%7c00000001&px=1) | **520.6259** | **1039.2372** | **1038.5822** | **0.6550** | **0** | **(65)** | **4.4e-008** | **1** | **R.LLLNAENPR.G** |
|  | [820](http://df3/mascot/cgi/peptide_view.pl?file=../data/20121119/F002569.dat&query=820&hit=1&index=gi%7c00000001&px=1) | **520.7757** | **1039.5368** | **1038.5822** | **0.9547** | **0** | **(31)** | **0.00013** | **1** | **R.LLLNAENPR.G** |
|  | [821](http://df3/mascot/cgi/peptide_view.pl?file=../data/20121119/F002569.dat&query=821&hit=1&index=gi%7c00000001&px=1) | **520.8201** | **1039.6257** | **1038.5822** | **1.0436** | **0** | **(39)** | **1.9e-005** | **1** | **R.LLLNAENPR.G** |
|  | [822](http://df3/mascot/cgi/peptide_view.pl?file=../data/20121119/F002569.dat&query=822&hit=1&index=gi%7c00000001&px=1) | **520.8352** | **1039.6559** | **1038.5822** | **1.0737** | **0** | **(17)** | **0.0033** | **1** | **R.LLLNAENPR.G** |
|  | [824](http://df3/mascot/cgi/peptide_view.pl?file=../data/20121119/F002569.dat&query=824&hit=1&index=gi%7c00000001&px=1) | **520.8918** | **1039.7691** | **1038.5822** | **1.1870** | **0** | **(27)** | **0.00031** | **1** | **R.LLLNAENPR.G** |
|  | [828](http://df3/mascot/cgi/peptide_view.pl?file=../data/20121119/F002569.dat&query=828&hit=1&index=gi%7c00000001&px=1) | **520.9993** | **1039.9840** | **1038.5822** | **1.4018** | **0** | **(21)** | **0.0011** | **1** | **R.LLLNAENPR.G** |
|  | [829](http://df3/mascot/cgi/peptide_view.pl?file=../data/20121119/F002569.dat&query=829&hit=1&index=gi%7c00000001&px=1) | **521.1649** | **1040.3152** | **1038.5822** | **1.7330** | **0** | **(62)** | **9.5e-008** | **1** | **R.LLLNAENPR.G** |
|  | [830](http://df3/mascot/cgi/peptide_view.pl?file=../data/20121119/F002569.dat&query=830&hit=1&index=gi%7c00000001&px=1) | **521.2802** | **1040.5458** | **1039.5662** | **0.9796** | **0** | **(14)** | **0.0055** | **1** | **R.LLLNAENPR.G + Deamidation (NQ)** |
|  | [831](http://df3/mascot/cgi/peptide_view.pl?file=../data/20121119/F002569.dat&query=831&hit=1&index=gi%7c00000001&px=1) | **521.4045** | **1040.7944** | **1039.5662** | **1.2282** | **0** | **(61)** | **8.1e-008** | **1** | **R.LLLNAENPR.G + Deamidation (NQ)** |
|  | [834](http://df3/mascot/cgi/peptide_view.pl?file=../data/20121119/F002569.dat&query=834&hit=1&index=gi%7c00000001&px=1) | **521.6654** | **1041.3163** | **1039.5662** | **1.7501** | **0** | **(10)** | **0.0091** | **1** | **R.LLLNAENPR.G + Deamidation (NQ)** |
|  | [841](http://df3/mascot/cgi/peptide_view.pl?file=../data/20121119/F002569.dat&query=841&hit=1&index=gi%7c00000001&px=1) | **524.8906** | **1047.7666** | **1047.5171** | **0.2495** | **0** | **(15)** | **0.0032** | **1** | **R.VPYPGMVNR.E + Oxidation (M)** |
|  | [842](http://df3/mascot/cgi/peptide_view.pl?file=../data/20121119/F002569.dat&query=842&hit=1&index=gi%7c00000001&px=1) | **524.9754** | **1047.9363** | **1047.5171** | **0.4191** | **0** | **(32)** | **6e-005** | **1** | **R.VPYPGMVNR.E + Oxidation (M)** |
|  | [843](http://df3/mascot/cgi/peptide_view.pl?file=../data/20121119/F002569.dat&query=843&hit=1&index=gi%7c00000001&px=1) | **525.0139** | **1048.0133** | **1047.5171** | **0.4962** | **0** | **(12)** | **0.006** | **1** | **R.VPYPGMVNR.E + Oxidation (M)** |
|  | [1206](http://df3/mascot/cgi/peptide_view.pl?file=../data/20121119/F002569.dat&query=1206&hit=1&index=gi%7c00000001&px=1) | **608.5112** | **1215.0079** | **1214.5931** | **0.4148** | **0** | **98** | **7.2e-012** | **1** | **K.LDSGGFYITSR.T** |
|  | [1207](http://df3/mascot/cgi/peptide_view.pl?file=../data/20121119/F002569.dat&query=1207&hit=1&index=gi%7c00000001&px=1) | **608.8934** | **1215.7722** | **1214.5931** | **1.1791** | **0** | **(90)** | **9.5e-011** | **1** | **K.LDSGGFYITSR.T** |
|  | [1208](http://df3/mascot/cgi/peptide_view.pl?file=../data/20121119/F002569.dat&query=1208&hit=1&index=gi%7c00000001&px=1) | **609.0460** | **1216.0775** | **1214.5931** | **1.4844** | **0** | **(92)** | **9.5e-011** | **1** | **K.LDSGGFYITSR.T** |
|  | [1233](http://df3/mascot/cgi/peptide_view.pl?file=../data/20121119/F002569.dat&query=1233&hit=1&index=gi%7c00000001&px=1) | **612.0584** | **1222.1023** | **1222.5829** | **-0.4807** | **0** | **(44)** | **4.1e-006** | **1** | **R.LIEDNEYTAR.Q** |
|  | [1234](http://df3/mascot/cgi/peptide_view.pl?file=../data/20121119/F002569.dat&query=1234&hit=1&index=gi%7c00000001&px=1) | **612.4805** | **1222.9465** | **1222.5829** | **0.3636** | **0** | **48** | **1.7e-006** | **1** | **R.LIEDNEYTAR.Q** |
|  | [1286](http://df3/mascot/cgi/peptide_view.pl?file=../data/20121119/F002569.dat&query=1286&hit=1&index=gi%7c00000001&px=1) | **623.2428** | **1244.4710** | **1244.6447** | **-0.1737** | **1** | **(50)** | **9.7e-007** | **1** | **K.GRVPYPGMVNR.E** |
|  | [299](http://df3/mascot/cgi/peptide_view.pl?file=../data/20121119/F002569.dat&query=299&hit=1&index=gi%7c00000001&px=1) | **416.2553** | **1245.7441** | **1244.6447** | **1.0994** | **1** | **51** | **8.2e-007** | **1** | **K.GRVPYPGMVNR.E** |
|  | [300](http://df3/mascot/cgi/peptide_view.pl?file=../data/20121119/F002569.dat&query=300&hit=1&index=gi%7c00000001&px=1) | **416.2784** | **1245.8133** | **1244.6447** | **1.1686** | **1** | **(46)** | **2.3e-006** | **1** | **K.GRVPYPGMVNR.E** |
|  | [301](http://df3/mascot/cgi/peptide_view.pl?file=../data/20121119/F002569.dat&query=301&hit=1&index=gi%7c00000001&px=1) | **416.3375** | **1245.9906** | **1244.6447** | **1.3458** | **1** | **(44)** | **3.6e-006** | **1** | **K.GRVPYPGMVNR.E** |
|  | [1290](http://df3/mascot/cgi/peptide_view.pl?file=../data/20121119/F002569.dat&query=1290&hit=1&index=gi%7c00000001&px=1) | **624.1410** | **1246.2674** | **1244.6447** | **1.6227** | **1** | **(46)** | **2.3e-006** | **1** | **K.GRVPYPGMVNR.E** |
|  | [321](http://df3/mascot/cgi/peptide_view.pl?file=../data/20121119/F002569.dat&query=321&hit=1&index=gi%7c00000001&px=1) | **421.1348** | **1260.3827** | **1260.6397** | **-0.2570** | **1** | **(50)** | **1e-006** | **1** | **K.GRVPYPGMVNR.E + Oxidation (M)** |
|  | [1326](http://df3/mascot/cgi/peptide_view.pl?file=../data/20121119/F002569.dat&query=1326&hit=1&index=gi%7c00000001&px=1) | **631.4047** | **1260.7948** | **1260.6397** | **0.1551** | **1** | **(26)** | **0.00024** | **1** | **K.GRVPYPGMVNR.E + Oxidation (M)** |
|  | [1328](http://df3/mascot/cgi/peptide_view.pl?file=../data/20121119/F002569.dat&query=1328&hit=1&index=gi%7c00000001&px=1) | **631.4521** | **1260.8897** | **1260.6397** | **0.2501** | **1** | **(16)** | **0.0024** | **1** | **K.GRVPYPGMVNR.E + Oxidation (M)** |
|  | [322](http://df3/mascot/cgi/peptide_view.pl?file=../data/20121119/F002569.dat&query=322&hit=1&index=gi%7c00000001&px=1) | **421.5079** | **1261.5019** | **1260.6397** | **0.8622** | **1** | **(37)** | **1.9e-005** | **1** | **K.GRVPYPGMVNR.E + Oxidation (M)** |
|  | [323](http://df3/mascot/cgi/peptide_view.pl?file=../data/20121119/F002569.dat&query=323&hit=1&index=gi%7c00000001&px=1) | **421.5272** | **1261.5598** | **1260.6397** | **0.9202** | **1** | **(13)** | **0.0055** | **1** | **K.GRVPYPGMVNR.E + Oxidation (M)** |
|  | [325](http://df3/mascot/cgi/peptide_view.pl?file=../data/20121119/F002569.dat&query=325&hit=1&index=gi%7c00000001&px=1) | **421.6211** | **1261.8414** | **1260.6397** | **1.2017** | **1** | **(51)** | **8.5e-007** | **1** | **K.GRVPYPGMVNR.E + Oxidation (M)** |
|  | [326](http://df3/mascot/cgi/peptide_view.pl?file=../data/20121119/F002569.dat&query=326&hit=1&index=gi%7c00000001&px=1) | **421.9912** | **1262.9516** | **1261.6237** | **1.3280** | **1** | **(39)** | **5.8e-006** | **1** | **K.GRVPYPGMVNR.E + Deamidation (NQ); Oxidation (M)** |
|  | [1330](http://df3/mascot/cgi/peptide_view.pl?file=../data/20121119/F002569.dat&query=1330&hit=1&index=gi%7c00000001&px=1) | **632.5048** | **1262.9950** | **1261.6237** | **1.3713** | **1** | **(20)** | **0.0005** | **1** | **K.GRVPYPGMVNR.E + Deamidation (NQ); Oxidation (M)** |
|  | [1346](http://df3/mascot/cgi/peptide_view.pl?file=../data/20121119/F002569.dat&query=1346&hit=1&index=gi%7c00000001&px=1) | **642.3018** | **1282.5890** | **1283.6509** | **-1.0620** | **0** | **92** | **2.8e-011** | **1** | **R.GPSAAFAPAAAEPK.L** |
|  | [1347](http://df3/mascot/cgi/peptide_view.pl?file=../data/20121119/F002569.dat&query=1347&hit=1&index=gi%7c00000001&px=1) | **642.6071** | **1283.1996** | **1283.6509** | **-0.4514** | **0** | **(85)** | **1.5e-010** | **1** | **R.GPSAAFAPAAAEPK.L** |
|  | [1348](http://df3/mascot/cgi/peptide_view.pl?file=../data/20121119/F002569.dat&query=1348&hit=1&index=gi%7c00000001&px=1) | **642.8814** | **1283.7483** | **1283.6509** | **0.0973** | **0** | **(60)** | **4.9e-008** | **1** | **R.GPSAAFAPAAAEPK.L** |
|  | [1350](http://df3/mascot/cgi/peptide_view.pl?file=../data/20121119/F002569.dat&query=1350&hit=1&index=gi%7c00000001&px=1) | **643.3041** | **1284.5936** | **1283.6509** | **0.9427** | **0** | **(45)** | **1.8e-006** | **1** | **R.GPSAAFAPAAAEPK.L** |
|  | [1351](http://df3/mascot/cgi/peptide_view.pl?file=../data/20121119/F002569.dat&query=1351&hit=1&index=gi%7c00000001&px=1) | **643.3463** | **1284.6780** | **1283.6509** | **1.0270** | **0** | **(38)** | **8.8e-006** | **1** | **R.GPSAAFAPAAAEPK.L** |
|  | [1352](http://df3/mascot/cgi/peptide_view.pl?file=../data/20121119/F002569.dat&query=1352&hit=1&index=gi%7c00000001&px=1) | **643.5546** | **1285.0946** | **1283.6509** | **1.4436** | **0** | **(66)** | **1.2e-008** | **1** | **R.GPSAAFAPAAAEPK.L** |
|  | [1353](http://df3/mascot/cgi/peptide_view.pl?file=../data/20121119/F002569.dat&query=1353&hit=1&index=gi%7c00000001&px=1) | **643.5814** | **1285.1483** | **1283.6509** | **1.4974** | **0** | **(76)** | **1.3e-009** | **1** | **R.GPSAAFAPAAAEPK.L** |
|  | [1468](http://df3/mascot/cgi/peptide_view.pl?file=../data/20121119/F002569.dat&query=1468&hit=1&index=gi%7c00000001&px=1) | **672.2754** | **1342.5362** | **1342.6881** | **-0.1518** | **1** | **108** | **2.4e-012** | **1** | **R.KLDSGGFYITSR.T** |
|  | [1469](http://df3/mascot/cgi/peptide_view.pl?file=../data/20121119/F002569.dat&query=1469&hit=1&index=gi%7c00000001&px=1) | **672.4230** | **1342.8314** | **1342.6881** | **0.1433** | **1** | **(21)** | **0.0021** | **1** | **R.KLDSGGFYITSR.T** |
|  | [1470](http://df3/mascot/cgi/peptide_view.pl?file=../data/20121119/F002569.dat&query=1470&hit=1&index=gi%7c00000001&px=1) | **672.5264** | **1343.0382** | **1342.6881** | **0.3501** | **1** | **(30)** | **0.00027** | **1** | **R.KLDSGGFYITSR.T** |
|  | [451](http://df3/mascot/cgi/peptide_view.pl?file=../data/20121119/F002569.dat&query=451&hit=1&index=gi%7c00000001&px=1) | **448.9995** | **1343.9767** | **1342.6881** | **1.2886** | **1** | **(31)** | **0.00016** | **1** | **R.KLDSGGFYITSR.T** |
|  | [1471](http://df3/mascot/cgi/peptide_view.pl?file=../data/20121119/F002569.dat&query=1471&hit=1&index=gi%7c00000001&px=1) | **673.1077** | **1344.2008** | **1342.6881** | **1.5127** | **1** | **(87)** | **6.5e-010** | **1** | **R.KLDSGGFYITSR.T** |
|  | [454](http://df3/mascot/cgi/peptide_view.pl?file=../data/20121119/F002569.dat&query=454&hit=1&index=gi%7c00000001&px=1) | **449.0884** | **1344.2433** | **1342.6881** | **1.5552** | **1** | **(38)** | **3.4e-005** | **1** | **R.KLDSGGFYITSR.T** |
|  | [459](http://df3/mascot/cgi/peptide_view.pl?file=../data/20121119/F002569.dat&query=459&hit=1&index=gi%7c00000001&px=1) | **449.4088** | **1345.2047** | **1344.6958** | **0.5089** | **0** | **(11)** | **0.0073** | **1** | **R.AANILVGENLVCK.V + 2 Deamidation (NQ)** |
|  | [1499](http://df3/mascot/cgi/peptide_view.pl?file=../data/20121119/F002569.dat&query=1499&hit=1&index=gi%7c00000001&px=1) | **682.5089** | **1363.0031** | **1363.7347** | **-0.7315** | **1** | **(55)** | **5.1e-007** | **1** | **K.GSLLDFLKGETGK.Y** |
|  | [1500](http://df3/mascot/cgi/peptide_view.pl?file=../data/20121119/F002569.dat&query=1500&hit=1&index=gi%7c00000001&px=1) | **682.5455** | **1363.0765** | **1363.7347** | **-0.6582** | **1** | **(51)** | **1.2e-006** | **1** | **K.GSLLDFLKGETGK.Y** |
|  | [477](http://df3/mascot/cgi/peptide_view.pl?file=../data/20121119/F002569.dat&query=477&hit=1&index=gi%7c00000001&px=1) | **455.4129** | **1363.2170** | **1363.7347** | **-0.5177** | **1** | **(29)** | **0.00024** | **1** | **K.GSLLDFLKGETGK.Y** |
|  | [1501](http://df3/mascot/cgi/peptide_view.pl?file=../data/20121119/F002569.dat&query=1501&hit=1&index=gi%7c00000001&px=1) | **682.6859** | **1363.3573** | **1363.7347** | **-0.3774** | **1** | **94** | **6.4e-011** | **1** | **K.GSLLDFLKGETGK.Y** |
|  | [1502](http://df3/mascot/cgi/peptide_view.pl?file=../data/20121119/F002569.dat&query=1502&hit=1&index=gi%7c00000001&px=1) | **682.7213** | **1363.4281** | **1363.7347** | **-0.3066** | **1** | **(91)** | **1.1e-010** | **1** | **K.GSLLDFLKGETGK.Y** |
|  | [1505](http://df3/mascot/cgi/peptide_view.pl?file=../data/20121119/F002569.dat&query=1505&hit=1&index=gi%7c00000001&px=1) | **683.1906** | **1364.3666** | **1363.7347** | **0.6319** | **1** | **(23)** | **0.00083** | **1** | **K.GSLLDFLKGETGK.Y** |
|  | [1506](http://df3/mascot/cgi/peptide_view.pl?file=../data/20121119/F002569.dat&query=1506&hit=1&index=gi%7c00000001&px=1) | **683.2598** | **1364.5050** | **1363.7347** | **0.7703** | **1** | **(20)** | **0.0016** | **1** | **K.GSLLDFLKGETGK.Y** |
|  | [478](http://df3/mascot/cgi/peptide_view.pl?file=../data/20121119/F002569.dat&query=478&hit=1&index=gi%7c00000001&px=1) | **456.0699** | **1365.1878** | **1363.7347** | **1.4532** | **1** | **(47)** | **2.7e-006** | **1** | **K.GSLLDFLKGETGK.Y** |
|  | [479](http://df3/mascot/cgi/peptide_view.pl?file=../data/20121119/F002569.dat&query=479&hit=1&index=gi%7c00000001&px=1) | **456.0825** | **1365.2255** | **1363.7347** | **1.4909** | **1** | **(37)** | **2.8e-005** | **1** | **K.GSLLDFLKGETGK.Y** |
|  | [480](http://df3/mascot/cgi/peptide_view.pl?file=../data/20121119/F002569.dat&query=480&hit=1&index=gi%7c00000001&px=1) | **456.0972** | **1365.2697** | **1363.7347** | **1.5350** | **1** | **(15)** | **0.0046** | **1** | **K.GSLLDFLKGETGK.Y** |
|  | [1518](http://df3/mascot/cgi/peptide_view.pl?file=../data/20121119/F002569.dat&query=1518&hit=1&index=gi%7c00000001&px=1) | **686.4974** | **1370.9802** | **1370.6942** | **0.2860** | **1** | **(46)** | **4.7e-006** | **1** | **K.DAWEIPRESLR.L** |
|  | [1519](http://df3/mascot/cgi/peptide_view.pl?file=../data/20121119/F002569.dat&query=1519&hit=1&index=gi%7c00000001&px=1) | **686.5864** | **1371.1583** | **1370.6942** | **0.4641** | **1** | **53** | **9.4e-007** | **1** | **K.DAWEIPRESLR.L** |
|  | [1520](http://df3/mascot/cgi/peptide_view.pl?file=../data/20121119/F002569.dat&query=1520&hit=1&index=gi%7c00000001&px=1) | **686.9426** | **1371.8707** | **1370.6942** | **1.1765** | **1** | **(44)** | **8.9e-006** | **1** | **K.DAWEIPRESLR.L** |
|  | [488](http://df3/mascot/cgi/peptide_view.pl?file=../data/20121119/F002569.dat&query=488&hit=1&index=gi%7c00000001&px=1) | **458.3368** | **1371.9885** | **1370.6942** | **1.2943** | **1** | **(21)** | **0.00037** | **1** | **K.DAWEIPRESLR.L** |
|  | [489](http://df3/mascot/cgi/peptide_view.pl?file=../data/20121119/F002569.dat&query=489&hit=1&index=gi%7c00000001&px=1) | **458.3512** | **1372.0319** | **1370.6942** | **1.3376** | **1** | **(22)** | **0.00034** | **1** | **K.DAWEIPRESLR.L** |
|  | [1522](http://df3/mascot/cgi/peptide_view.pl?file=../data/20121119/F002569.dat&query=1522&hit=1&index=gi%7c00000001&px=1) | **687.0680** | **1372.1214** | **1370.6942** | **1.4272** | **1** | **(45)** | **6.2e-006** | **1** | **K.DAWEIPRESLR.L** |
|  | [490](http://df3/mascot/cgi/peptide_view.pl?file=../data/20121119/F002569.dat&query=490&hit=1&index=gi%7c00000001&px=1) | **458.4110** | **1372.2112** | **1370.6942** | **1.5170** | **1** | **(19)** | **0.00061** | **1** | **K.DAWEIPRESLR.L** |
|  | [491](http://df3/mascot/cgi/peptide_view.pl?file=../data/20121119/F002569.dat&query=491&hit=1&index=gi%7c00000001&px=1) | **458.4293** | **1372.2660** | **1370.6942** | **1.5718** | **1** | **(27)** | **9.6e-005** | **1** | **K.DAWEIPRESLR.L** |
|  | [1568](http://df3/mascot/cgi/peptide_view.pl?file=../data/20121119/F002569.dat&query=1568&hit=1&index=gi%7c00000001&px=1) | **700.3456** | **1398.6767** | **1399.7493** | **-1.0725** | **0** | **(73)** | **1.2e-008** | **1** | **R.AANILVGENLVCK.V + Carbamidomethyl (C)** |
|  | [584](http://df3/mascot/cgi/peptide_view.pl?file=../data/20121119/F002569.dat&query=584&hit=1&index=gi%7c00000001&px=1) | **467.3723** | **1399.0950** | **1399.7493** | **-0.6542** | **0** | **(15)** | **0.005** | **1** | **R.AANILVGENLVCK.V + Carbamidomethyl (C)** |
|  | [1570](http://df3/mascot/cgi/peptide_view.pl?file=../data/20121119/F002569.dat&query=1570&hit=1&index=gi%7c00000001&px=1) | **700.9343** | **1399.8541** | **1399.7493** | **0.1048** | **0** | **(64)** | **1.3e-007** | **1** | **R.AANILVGENLVCK.V + Carbamidomethyl (C)** |
|  | [1571](http://df3/mascot/cgi/peptide_view.pl?file=../data/20121119/F002569.dat&query=1571&hit=1&index=gi%7c00000001&px=1) | **700.9647** | **1399.9148** | **1399.7493** | **0.1655** | **0** | **(86)** | **6.8e-010** | **1** | **R.AANILVGENLVCK.V + Carbamidomethyl (C)** |
|  | [1572](http://df3/mascot/cgi/peptide_view.pl?file=../data/20121119/F002569.dat&query=1572&hit=1&index=gi%7c00000001&px=1) | **701.0250** | **1400.0355** | **1399.7493** | **0.2862** | **0** | **97** | **5.6e-011** | **1** | **R.AANILVGENLVCK.V + Carbamidomethyl (C)** |
|  | [1573](http://df3/mascot/cgi/peptide_view.pl?file=../data/20121119/F002569.dat&query=1573&hit=1&index=gi%7c00000001&px=1) | **701.0995** | **1400.1844** | **1399.7493** | **0.4352** | **0** | **(49)** | **4.2e-006** | **1** | **R.AANILVGENLVCK.V + Carbamidomethyl (C)** |
|  | [1574](http://df3/mascot/cgi/peptide_view.pl?file=../data/20121119/F002569.dat&query=1574&hit=1&index=gi%7c00000001&px=1) | **701.2979** | **1400.5813** | **1399.7493** | **0.8320** | **0** | **(53)** | **1.5e-006** | **1** | **R.AANILVGENLVCK.V + Carbamidomethyl (C)** |
|  | [1575](http://df3/mascot/cgi/peptide_view.pl?file=../data/20121119/F002569.dat&query=1575&hit=1&index=gi%7c00000001&px=1) | **701.3491** | **1400.6837** | **1399.7493** | **0.9344** | **0** | **(94)** | **9.2e-011** | **1** | **R.AANILVGENLVCK.V + Carbamidomethyl (C)** |
|  | [593](http://df3/mascot/cgi/peptide_view.pl?file=../data/20121119/F002569.dat&query=593&hit=1&index=gi%7c00000001&px=1) | **467.9330** | **1400.7773** | **1399.7493** | **1.0280** | **0** | **(56)** | **3.5e-007** | **1** | **R.AANILVGENLVCK.V + Carbamidomethyl (C)** |
|  | [595](http://df3/mascot/cgi/peptide_view.pl?file=../data/20121119/F002569.dat&query=595&hit=1&index=gi%7c00000001&px=1) | **467.9520** | **1400.8343** | **1399.7493** | **1.0850** | **0** | **(70)** | **1.7e-008** | **1** | **R.AANILVGENLVCK.V + Carbamidomethyl (C)** |
|  | [1576](http://df3/mascot/cgi/peptide_view.pl?file=../data/20121119/F002569.dat&query=1576&hit=1&index=gi%7c00000001&px=1) | **701.6073** | **1401.2000** | **1399.7493** | **1.4508** | **0** | **(77)** | **4.9e-009** | **1** | **R.AANILVGENLVCK.V + Carbamidomethyl (C)** |
|  | [1577](http://df3/mascot/cgi/peptide_view.pl?file=../data/20121119/F002569.dat&query=1577&hit=1&index=gi%7c00000001&px=1) | **701.6185** | **1401.2224** | **1399.7493** | **1.4731** | **0** | **(79)** | **3.4e-009** | **1** | **R.AANILVGENLVCK.V + Carbamidomethyl (C)** |
|  | [1579](http://df3/mascot/cgi/peptide_view.pl?file=../data/20121119/F002569.dat&query=1579&hit=1&index=gi%7c00000001&px=1) | **701.8220** | **1401.6294** | **1399.7493** | **1.8801** | **0** | **(19)** | **0.0023** | **1** | **R.AANILVGENLVCK.V + Carbamidomethyl (C)** |
|  | [1580](http://df3/mascot/cgi/peptide_view.pl?file=../data/20121119/F002569.dat&query=1580&hit=1&index=gi%7c00000001&px=1) | **701.8370** | **1401.6595** | **1400.7333** | **0.9262** | **0** | **(37)** | **4.5e-005** | **1** | **R.AANILVGENLVCK.V + Carbamidomethyl (C); Deamidation (NQ)** |
|  | [1581](http://df3/mascot/cgi/peptide_view.pl?file=../data/20121119/F002569.dat&query=1581&hit=1&index=gi%7c00000001&px=1) | **701.8707** | **1401.7269** | **1399.7493** | **1.9776** | **0** | **(35)** | **6.9e-005** | **1** | **R.AANILVGENLVCK.V + Carbamidomethyl (C)** |
|  | [1582](http://df3/mascot/cgi/peptide_view.pl?file=../data/20121119/F002569.dat&query=1582&hit=1&index=gi%7c00000001&px=1) | **702.0137** | **1402.0129** | **1400.7333** | **1.2796** | **0** | **(73)** | **5.2e-009** | **1** | **R.AANILVGENLVCK.V + Carbamidomethyl (C); Deamidation (NQ)** |
|  | [1583](http://df3/mascot/cgi/peptide_view.pl?file=../data/20121119/F002569.dat&query=1583&hit=1&index=gi%7c00000001&px=1) | **702.0282** | **1402.0418** | **1400.7333** | **1.3086** | **0** | **(15)** | **0.0031** | **1** | **R.AANILVGENLVCK.V + Carbamidomethyl (C); Deamidation (NQ)** |
|  | [1587](http://df3/mascot/cgi/peptide_view.pl?file=../data/20121119/F002569.dat&query=1587&hit=1&index=gi%7c00000001&px=1) | **702.1246** | **1402.2346** | **1400.7333** | **1.5013** | **0** | **(33)** | **4.7e-005** | **1** | **R.AANILVGENLVCK.V + Carbamidomethyl (C); Deamidation (NQ)** |
|  | [1589](http://df3/mascot/cgi/peptide_view.pl?file=../data/20121119/F002569.dat&query=1589&hit=1&index=gi%7c00000001&px=1) | **702.2091** | **1402.4037** | **1400.7333** | **1.6704** | **0** | **(73)** | **5e-009** | **1** | **R.AANILVGENLVCK.V + Carbamidomethyl (C); Deamidation (NQ)** |
|  | [1925](http://df3/mascot/cgi/peptide_view.pl?file=../data/20121119/F002569.dat&query=1925&hit=1&index=gi%7c00000001&px=1) | **745.9460** | **1489.8775** | **1488.6554** | **1.2221** | **0** | **(10)** | **0.009** | **1** | **K.GAYCLSVSDFDNAK.G** |
|  | [2140](http://df3/mascot/cgi/peptide_view.pl?file=../data/20121119/F002569.dat&query=2140&hit=1&index=gi%7c00000001&px=1) | **770.9617** | **1539.9088** | **1539.8005** | **0.1083** | **1** | **(30)** | **0.00022** | **1** | **R.ESERLLLNAENPR.G** |
|  | [763](http://df3/mascot/cgi/peptide_view.pl?file=../data/20121119/F002569.dat&query=763&hit=1&index=gi%7c00000001&px=1) | **514.4980** | **1540.4723** | **1539.8005** | **0.6719** | **1** | **(49)** | **2.4e-006** | **1** | **R.ESERLLLNAENPR.G** |
|  | [765](http://df3/mascot/cgi/peptide_view.pl?file=../data/20121119/F002569.dat&query=765&hit=1&index=gi%7c00000001&px=1) | **514.6069** | **1540.7990** | **1539.8005** | **0.9985** | **1** | **(51)** | **2e-006** | **1** | **R.ESERLLLNAENPR.G** |
|  | [2144](http://df3/mascot/cgi/peptide_view.pl?file=../data/20121119/F002569.dat&query=2144&hit=1&index=gi%7c00000001&px=1) | **771.5399** | **1541.0653** | **1539.8005** | **1.2648** | **1** | **51** | **1.9e-006** | **1** | **R.ESERLLLNAENPR.G** |
|  | [2149](http://df3/mascot/cgi/peptide_view.pl?file=../data/20121119/F002569.dat&query=2149&hit=1&index=gi%7c00000001&px=1) | **773.8282** | **1545.6419** | **1545.6769** | **-0.0350** | **0** | **(102)** | **6.5e-012** | **1** | **K.GAYCLSVSDFDNAK.G + Carbamidomethyl (C)** |
|  | [2151](http://df3/mascot/cgi/peptide_view.pl?file=../data/20121119/F002569.dat&query=2151&hit=1&index=gi%7c00000001&px=1) | **773.9008** | **1545.7871** | **1545.6769** | **0.1102** | **0** | **106** | **2.7e-012** | **1** | **K.GAYCLSVSDFDNAK.G + Carbamidomethyl (C)** |
|  | [2152](http://df3/mascot/cgi/peptide_view.pl?file=../data/20121119/F002569.dat&query=2152&hit=1&index=gi%7c00000001&px=1) | **774.4362** | **1546.8578** | **1545.6769** | **1.1809** | **0** | **(100)** | **1e-011** | **1** | **K.GAYCLSVSDFDNAK.G + Carbamidomethyl (C)** |
|  | [2153](http://df3/mascot/cgi/peptide_view.pl?file=../data/20121119/F002569.dat&query=2153&hit=1&index=gi%7c00000001&px=1) | **774.9086** | **1547.8026** | **1546.6609** | **1.1417** | **0** | **(74)** | **2.2e-009** | **1** | **K.GAYCLSVSDFDNAK.G + Carbamidomethyl (C); Deamidation (NQ)** |
|  | [2154](http://df3/mascot/cgi/peptide_view.pl?file=../data/20121119/F002569.dat&query=2154&hit=1&index=gi%7c00000001&px=1) | **774.9593** | **1547.9040** | **1546.6609** | **1.2431** | **0** | **(13)** | **0.0027** | **1** | **K.GAYCLSVSDFDNAK.G + Carbamidomethyl (C); Deamidation (NQ)** |
|  | [2155](http://df3/mascot/cgi/peptide_view.pl?file=../data/20121119/F002569.dat&query=2155&hit=1&index=gi%7c00000001&px=1) | **775.0218** | **1548.0290** | **1546.6609** | **1.3681** | **0** | **(80)** | **4.5e-010** | **1** | **K.GAYCLSVSDFDNAK.G + Carbamidomethyl (C); Deamidation (NQ)** |
|  | [892](http://df3/mascot/cgi/peptide_view.pl?file=../data/20121119/F002569.dat&query=892&hit=1&index=gi%7c00000001&px=1) | **537.2448** | **1608.7124** | **1607.7791** | **0.9334** | **1** | **16** | **0.0051** | **1** | **R.LIEDNEYTARQGAK.F + Deamidation (NQ)** |
|  | [2411](http://df3/mascot/cgi/peptide_view.pl?file=../data/20121119/F002569.dat&query=2411&hit=1&index=gi%7c00000001&px=1) | **855.9208** | **1709.8271** | **1711.8165** | **-1.9894** | **0** | **(67)** | **8.1e-008** | **1** | **K.LFGGFNSSDTVTSPQR.A** |
|  | [2412](http://df3/mascot/cgi/peptide_view.pl?file=../data/20121119/F002569.dat&query=2412&hit=1&index=gi%7c00000001&px=1) | **855.9243** | **1709.8340** | **1711.8165** | **-1.9826** | **0** | **(68)** | **6.6e-008** | **1** | **K.LFGGFNSSDTVTSPQR.A** |
|  | [2413](http://df3/mascot/cgi/peptide_view.pl?file=../data/20121119/F002569.dat&query=2413&hit=1&index=gi%7c00000001&px=1) | **855.9391** | **1709.8636** | **1711.8165** | **-1.9529** | **0** | **(33)** | **0.00021** | **1** | **K.LFGGFNSSDTVTSPQR.A** |
|  | [2414](http://df3/mascot/cgi/peptide_view.pl?file=../data/20121119/F002569.dat&query=2414&hit=1&index=gi%7c00000001&px=1) | **856.2296** | **1710.4446** | **1711.8165** | **-1.3720** | **0** | **(118)** | **7.5e-013** | **1** | **K.LFGGFNSSDTVTSPQR.A** |
|  | [2415](http://df3/mascot/cgi/peptide_view.pl?file=../data/20121119/F002569.dat&query=2415&hit=1&index=gi%7c00000001&px=1) | **856.3000** | **1710.5854** | **1711.8165** | **-1.2311** | **0** | **(116)** | **1.2e-012** | **1** | **K.LFGGFNSSDTVTSPQR.A** |
|  | [2416](http://df3/mascot/cgi/peptide_view.pl?file=../data/20121119/F002569.dat&query=2416&hit=1&index=gi%7c00000001&px=1) | **856.3538** | **1710.6930** | **1711.8165** | **-1.1235** | **0** | **(84)** | **1.7e-009** | **1** | **K.LFGGFNSSDTVTSPQR.A** |
|  | [2417](http://df3/mascot/cgi/peptide_view.pl?file=../data/20121119/F002569.dat&query=2417&hit=1&index=gi%7c00000001&px=1) | **856.3703** | **1710.7260** | **1711.8165** | **-1.0905** | **0** | **(75)** | **1.4e-008** | **1** | **K.LFGGFNSSDTVTSPQR.A** |
|  | [2418](http://df3/mascot/cgi/peptide_view.pl?file=../data/20121119/F002569.dat&query=2418&hit=1&index=gi%7c00000001&px=1) | **856.3768** | **1710.7390** | **1711.8165** | **-1.0775** | **0** | **(78)** | **6.5e-009** | **1** | **K.LFGGFNSSDTVTSPQR.A** |
|  | [2419](http://df3/mascot/cgi/peptide_view.pl?file=../data/20121119/F002569.dat&query=2419&hit=1&index=gi%7c00000001&px=1) | **856.3922** | **1710.7699** | **1711.8165** | **-1.0466** | **0** | **(59)** | **5.8e-007** | **1** | **K.LFGGFNSSDTVTSPQR.A** |
|  | [2420](http://df3/mascot/cgi/peptide_view.pl?file=../data/20121119/F002569.dat&query=2420&hit=1&index=gi%7c00000001&px=1) | **856.4297** | **1710.8449** | **1711.8165** | **-0.9716** | **0** | **(105)** | **1.8e-011** | **1** | **K.LFGGFNSSDTVTSPQR.A** |
|  | [2421](http://df3/mascot/cgi/peptide_view.pl?file=../data/20121119/F002569.dat&query=2421&hit=1&index=gi%7c00000001&px=1) | **856.4412** | **1710.8678** | **1711.8165** | **-0.9487** | **0** | **(98)** | **1e-010** | **1** | **K.LFGGFNSSDTVTSPQR.A** |
|  | [2422](http://df3/mascot/cgi/peptide_view.pl?file=../data/20121119/F002569.dat&query=2422&hit=1&index=gi%7c00000001&px=1) | **856.4602** | **1710.9059** | **1711.8165** | **-0.9107** | **0** | **(71)** | **4.8e-008** | **1** | **K.LFGGFNSSDTVTSPQR.A** |
|  | [2423](http://df3/mascot/cgi/peptide_view.pl?file=../data/20121119/F002569.dat&query=2423&hit=1&index=gi%7c00000001&px=1) | **856.4843** | **1710.9540** | **1711.8165** | **-0.8626** | **0** | **(98)** | **8e-011** | **1** | **K.LFGGFNSSDTVTSPQR.A** |
|  | [2424](http://df3/mascot/cgi/peptide_view.pl?file=../data/20121119/F002569.dat&query=2424&hit=1&index=gi%7c00000001&px=1) | **856.5194** | **1711.0243** | **1711.8165** | **-0.7922** | **0** | **(88)** | **9.5e-010** | **1** | **K.LFGGFNSSDTVTSPQR.A** |
|  | [2425](http://df3/mascot/cgi/peptide_view.pl?file=../data/20121119/F002569.dat&query=2425&hit=1&index=gi%7c00000001&px=1) | **856.5296** | **1711.0447** | **1711.8165** | **-0.7719** | **0** | **(105)** | **1.9e-011** | **1** | **K.LFGGFNSSDTVTSPQR.A** |
|  | [2426](http://df3/mascot/cgi/peptide_view.pl?file=../data/20121119/F002569.dat&query=2426&hit=1&index=gi%7c00000001&px=1) | **856.7506** | **1711.4867** | **1711.8165** | **-0.3298** | **0** | **(120)** | **7.7e-013** | **1** | **K.LFGGFNSSDTVTSPQR.A** |
|  | [2427](http://df3/mascot/cgi/peptide_view.pl?file=../data/20121119/F002569.dat&query=2427&hit=1&index=gi%7c00000001&px=1) | **856.7660** | **1711.5174** | **1711.8165** | **-0.2991** | **0** | **(90)** | **7e-010** | **1** | **K.LFGGFNSSDTVTSPQR.A** |
|  | [2428](http://df3/mascot/cgi/peptide_view.pl?file=../data/20121119/F002569.dat&query=2428&hit=1&index=gi%7c00000001&px=1) | **856.8199** | **1711.6253** | **1711.8165** | **-0.1912** | **0** | **(106)** | **1.5e-011** | **1** | **K.LFGGFNSSDTVTSPQR.A** |
|  | [2429](http://df3/mascot/cgi/peptide_view.pl?file=../data/20121119/F002569.dat&query=2429&hit=1&index=gi%7c00000001&px=1) | **856.8220** | **1711.6295** | **1711.8165** | **-0.1870** | **0** | **(89)** | **8.6e-010** | **1** | **K.LFGGFNSSDTVTSPQR.A** |
|  | [2430](http://df3/mascot/cgi/peptide_view.pl?file=../data/20121119/F002569.dat&query=2430&hit=1&index=gi%7c00000001&px=1) | **856.8235** | **1711.6324** | **1711.8165** | **-0.1841** | **0** | **(74)** | **2.8e-008** | **1** | **K.LFGGFNSSDTVTSPQR.A** |
|  | [2431](http://df3/mascot/cgi/peptide_view.pl?file=../data/20121119/F002569.dat&query=2431&hit=1&index=gi%7c00000001&px=1) | **856.8431** | **1711.6716** | **1711.8165** | **-0.1449** | **0** | **(114)** | **2.4e-012** | **1** | **K.LFGGFNSSDTVTSPQR.A** |
|  | [2432](http://df3/mascot/cgi/peptide_view.pl?file=../data/20121119/F002569.dat&query=2432&hit=1&index=gi%7c00000001&px=1) | **856.8486** | **1711.6826** | **1711.8165** | **-0.1339** | **0** | **(93)** | **3.5e-010** | **1** | **K.LFGGFNSSDTVTSPQR.A** |
|  | [2433](http://df3/mascot/cgi/peptide_view.pl?file=../data/20121119/F002569.dat&query=2433&hit=1&index=gi%7c00000001&px=1) | **856.8495** | **1711.6844** | **1711.8165** | **-0.1321** | **0** | **(93)** | **3.2e-010** | **1** | **K.LFGGFNSSDTVTSPQR.A** |
|  | [2434](http://df3/mascot/cgi/peptide_view.pl?file=../data/20121119/F002569.dat&query=2434&hit=1&index=gi%7c00000001&px=1) | **856.8703** | **1711.7260** | **1711.8165** | **-0.0905** | **0** | **(102)** | **4.3e-011** | **1** | **K.LFGGFNSSDTVTSPQR.A** |
|  | [2435](http://df3/mascot/cgi/peptide_view.pl?file=../data/20121119/F002569.dat&query=2435&hit=1&index=gi%7c00000001&px=1) | **856.8805** | **1711.7464** | **1711.8165** | **-0.0701** | **0** | **(108)** | **9.9e-012** | **1** | **K.LFGGFNSSDTVTSPQR.A** |
|  | [2436](http://df3/mascot/cgi/peptide_view.pl?file=../data/20121119/F002569.dat&query=2436&hit=1&index=gi%7c00000001&px=1) | **856.8973** | **1711.7801** | **1711.8165** | **-0.0364** | **0** | **(108)** | **1e-011** | **1** | **K.LFGGFNSSDTVTSPQR.A** |
|  | [2437](http://df3/mascot/cgi/peptide_view.pl?file=../data/20121119/F002569.dat&query=2437&hit=1&index=gi%7c00000001&px=1) | **856.9088** | **1711.8030** | **1711.8165** | **-0.0136** | **0** | **(80)** | **6.4e-009** | **1** | **K.LFGGFNSSDTVTSPQR.A** |
|  | [2438](http://df3/mascot/cgi/peptide_view.pl?file=../data/20121119/F002569.dat&query=2438&hit=1&index=gi%7c00000001&px=1) | **856.9158** | **1711.8170** | **1711.8165** | **0.0005** | **0** | **(117)** | **1.4e-012** | **1** | **K.LFGGFNSSDTVTSPQR.A** |
|  | [2439](http://df3/mascot/cgi/peptide_view.pl?file=../data/20121119/F002569.dat&query=2439&hit=1&index=gi%7c00000001&px=1) | **856.9355** | **1711.8565** | **1711.8165** | **0.0400** | **0** | **(94)** | **2.5e-010** | **1** | **K.LFGGFNSSDTVTSPQR.A** |
|  | [2440](http://df3/mascot/cgi/peptide_view.pl?file=../data/20121119/F002569.dat&query=2440&hit=1&index=gi%7c00000001&px=1) | **856.9388** | **1711.8631** | **1711.8165** | **0.0466** | **0** | **(95)** | **2.5e-010** | **1** | **K.LFGGFNSSDTVTSPQR.A** |
|  | [2441](http://df3/mascot/cgi/peptide_view.pl?file=../data/20121119/F002569.dat&query=2441&hit=1&index=gi%7c00000001&px=1) | **856.9390** | **1711.8634** | **1711.8165** | **0.0469** | **0** | **(119)** | **8.9e-013** | **1** | **K.LFGGFNSSDTVTSPQR.A** |
|  | [2442](http://df3/mascot/cgi/peptide_view.pl?file=../data/20121119/F002569.dat&query=2442&hit=1&index=gi%7c00000001&px=1) | **856.9415** | **1711.8684** | **1711.8165** | **0.0519** | **0** | **(117)** | **1.5e-012** | **1** | **K.LFGGFNSSDTVTSPQR.A** |
|  | [2443](http://df3/mascot/cgi/peptide_view.pl?file=../data/20121119/F002569.dat&query=2443&hit=1&index=gi%7c00000001&px=1) | **856.9440** | **1711.8734** | **1711.8165** | **0.0569** | **0** | **(92)** | **4.6e-010** | **1** | **K.LFGGFNSSDTVTSPQR.A** |
|  | [2444](http://df3/mascot/cgi/peptide_view.pl?file=../data/20121119/F002569.dat&query=2444&hit=1&index=gi%7c00000001&px=1) | **856.9460** | **1711.8774** | **1711.8165** | **0.0609** | **0** | **(97)** | **1.3e-010** | **1** | **K.LFGGFNSSDTVTSPQR.A** |
|  | [2445](http://df3/mascot/cgi/peptide_view.pl?file=../data/20121119/F002569.dat&query=2445&hit=1&index=gi%7c00000001&px=1) | **856.9506** | **1711.8866** | **1711.8165** | **0.0701** | **0** | **(108)** | **1.1e-011** | **1** | **K.LFGGFNSSDTVTSPQR.A** |
|  | [2446](http://df3/mascot/cgi/peptide_view.pl?file=../data/20121119/F002569.dat&query=2446&hit=1&index=gi%7c00000001&px=1) | **856.9534** | **1711.8922** | **1711.8165** | **0.0757** | **0** | **(96)** | **1.9e-010** | **1** | **K.LFGGFNSSDTVTSPQR.A** |
|  | [2447](http://df3/mascot/cgi/peptide_view.pl?file=../data/20121119/F002569.dat&query=2447&hit=1&index=gi%7c00000001&px=1) | **856.9620** | **1711.9095** | **1711.8165** | **0.0930** | **0** | **(112)** | **4.8e-012** | **1** | **K.LFGGFNSSDTVTSPQR.A** |
|  | [2448](http://df3/mascot/cgi/peptide_view.pl?file=../data/20121119/F002569.dat&query=2448&hit=1&index=gi%7c00000001&px=1) | **856.9651** | **1711.9156** | **1711.8165** | **0.0991** | **0** | **(107)** | **1.4e-011** | **1** | **K.LFGGFNSSDTVTSPQR.A** |
|  | [2449](http://df3/mascot/cgi/peptide_view.pl?file=../data/20121119/F002569.dat&query=2449&hit=1&index=gi%7c00000001&px=1) | **856.9666** | **1711.9186** | **1711.8165** | **0.1020** | **0** | **(108)** | **1.1e-011** | **1** | **K.LFGGFNSSDTVTSPQR.A** |
|  | [2450](http://df3/mascot/cgi/peptide_view.pl?file=../data/20121119/F002569.dat&query=2450&hit=1&index=gi%7c00000001&px=1) | **856.9690** | **1711.9234** | **1711.8165** | **0.1069** | **0** | **(101)** | **5.5e-011** | **1** | **K.LFGGFNSSDTVTSPQR.A** |
|  | [2451](http://df3/mascot/cgi/peptide_view.pl?file=../data/20121119/F002569.dat&query=2451&hit=1&index=gi%7c00000001&px=1) | **856.9791** | **1711.9437** | **1711.8165** | **0.1272** | **0** | **(105)** | **2.2e-011** | **1** | **K.LFGGFNSSDTVTSPQR.A** |
|  | [2452](http://df3/mascot/cgi/peptide_view.pl?file=../data/20121119/F002569.dat&query=2452&hit=1&index=gi%7c00000001&px=1) | **857.0081** | **1712.0016** | **1711.8165** | **0.1850** | **0** | **(105)** | **2.4e-011** | **1** | **K.LFGGFNSSDTVTSPQR.A** |
|  | [2453](http://df3/mascot/cgi/peptide_view.pl?file=../data/20121119/F002569.dat&query=2453&hit=1&index=gi%7c00000001&px=1) | **857.0129** | **1712.0113** | **1711.8165** | **0.1948** | **0** | **(100)** | **7.1e-011** | **1** | **K.LFGGFNSSDTVTSPQR.A** |
|  | [2454](http://df3/mascot/cgi/peptide_view.pl?file=../data/20121119/F002569.dat&query=2454&hit=1&index=gi%7c00000001&px=1) | **857.0180** | **1712.0215** | **1711.8165** | **0.2049** | **0** | **(111)** | **6.3e-012** | **1** | **K.LFGGFNSSDTVTSPQR.A** |
|  | [2455](http://df3/mascot/cgi/peptide_view.pl?file=../data/20121119/F002569.dat&query=2455&hit=1&index=gi%7c00000001&px=1) | **857.0216** | **1712.0287** | **1711.8165** | **0.2121** | **0** | **(106)** | **2.1e-011** | **1** | **K.LFGGFNSSDTVTSPQR.A** |
|  | [2456](http://df3/mascot/cgi/peptide_view.pl?file=../data/20121119/F002569.dat&query=2456&hit=1&index=gi%7c00000001&px=1) | **857.0267** | **1712.0389** | **1711.8165** | **0.2224** | **0** | **(100)** | **7e-011** | **1** | **K.LFGGFNSSDTVTSPQR.A** |
|  | [2457](http://df3/mascot/cgi/peptide_view.pl?file=../data/20121119/F002569.dat&query=2457&hit=1&index=gi%7c00000001&px=1) | **857.0487** | **1712.0829** | **1711.8165** | **0.2663** | **0** | **(117)** | **1.5e-012** | **1** | **K.LFGGFNSSDTVTSPQR.A** |
|  | [2458](http://df3/mascot/cgi/peptide_view.pl?file=../data/20121119/F002569.dat&query=2458&hit=1&index=gi%7c00000001&px=1) | **857.0681** | **1712.1217** | **1711.8165** | **0.3052** | **0** | **(112)** | **5.8e-012** | **1** | **K.LFGGFNSSDTVTSPQR.A** |
|  | [2459](http://df3/mascot/cgi/peptide_view.pl?file=../data/20121119/F002569.dat&query=2459&hit=1&index=gi%7c00000001&px=1) | **857.0765** | **1712.1385** | **1711.8165** | **0.3220** | **0** | **(72)** | **5.6e-008** | **1** | **K.LFGGFNSSDTVTSPQR.A** |
|  | [2460](http://df3/mascot/cgi/peptide_view.pl?file=../data/20121119/F002569.dat&query=2460&hit=1&index=gi%7c00000001&px=1) | **857.1071** | **1712.1997** | **1712.8005** | **-0.6008** | **0** | **(134)** | **3.5e-014** | **1** | **K.LFGGFNSSDTVTSPQR.A + Deamidation (NQ)** |
|  | [2461](http://df3/mascot/cgi/peptide_view.pl?file=../data/20121119/F002569.dat&query=2461&hit=1&index=gi%7c00000001&px=1) | **857.1361** | **1712.2577** | **1711.8165** | **0.4412** | **0** | **(102)** | **4.8e-011** | **1** | **K.LFGGFNSSDTVTSPQR.A** |
|  | [2462](http://df3/mascot/cgi/peptide_view.pl?file=../data/20121119/F002569.dat&query=2462&hit=1&index=gi%7c00000001&px=1) | **857.1476** | **1712.2806** | **1711.8165** | **0.4641** | **0** | **(115)** | **2.8e-012** | **1** | **K.LFGGFNSSDTVTSPQR.A** |
|  | [2463](http://df3/mascot/cgi/peptide_view.pl?file=../data/20121119/F002569.dat&query=2463&hit=1&index=gi%7c00000001&px=1) | **857.1680** | **1712.3214** | **1711.8165** | **0.5049** | **0** | **(103)** | **3.6e-011** | **1** | **K.LFGGFNSSDTVTSPQR.A** |
|  | [2464](http://df3/mascot/cgi/peptide_view.pl?file=../data/20121119/F002569.dat&query=2464&hit=1&index=gi%7c00000001&px=1) | **857.1720** | **1712.3294** | **1711.8165** | **0.5129** | **0** | **(103)** | **4.2e-011** | **1** | **K.LFGGFNSSDTVTSPQR.A** |
|  | [2465](http://df3/mascot/cgi/peptide_view.pl?file=../data/20121119/F002569.dat&query=2465&hit=1&index=gi%7c00000001&px=1) | **857.1926** | **1712.3707** | **1711.8165** | **0.5542** | **0** | **(83)** | **4.3e-009** | **1** | **K.LFGGFNSSDTVTSPQR.A** |
|  | [2466](http://df3/mascot/cgi/peptide_view.pl?file=../data/20121119/F002569.dat&query=2466&hit=1&index=gi%7c00000001&px=1) | **857.2124** | **1712.4103** | **1711.8165** | **0.5937** | **0** | **(119)** | **1e-012** | **1** | **K.LFGGFNSSDTVTSPQR.A** |
|  | [2467](http://df3/mascot/cgi/peptide_view.pl?file=../data/20121119/F002569.dat&query=2467&hit=1&index=gi%7c00000001&px=1) | **857.3098** | **1712.6050** | **1711.8165** | **0.7884** | **0** | **(113)** | **3.6e-012** | **1** | **K.LFGGFNSSDTVTSPQR.A** |
|  | [2468](http://df3/mascot/cgi/peptide_view.pl?file=../data/20121119/F002569.dat&query=2468&hit=1&index=gi%7c00000001&px=1) | **857.3318** | **1712.6490** | **1711.8165** | **0.8325** | **0** | **(117)** | **1.8e-012** | **1** | **K.LFGGFNSSDTVTSPQR.A** |
|  | [2469](http://df3/mascot/cgi/peptide_view.pl?file=../data/20121119/F002569.dat&query=2469&hit=1&index=gi%7c00000001&px=1) | **857.3425** | **1712.6705** | **1711.8165** | **0.8540** | **0** | **(111)** | **5.7e-012** | **1** | **K.LFGGFNSSDTVTSPQR.A** |
|  | [2470](http://df3/mascot/cgi/peptide_view.pl?file=../data/20121119/F002569.dat&query=2470&hit=1&index=gi%7c00000001&px=1) | **857.3490** | **1712.6834** | **1711.8165** | **0.8669** | **0** | **(108)** | **1.3e-011** | **1** | **K.LFGGFNSSDTVTSPQR.A** |
|  | [2471](http://df3/mascot/cgi/peptide_view.pl?file=../data/20121119/F002569.dat&query=2471&hit=1&index=gi%7c00000001&px=1) | **857.3517** | **1712.6888** | **1711.8165** | **0.8723** | **0** | **(120)** | **7.3e-013** | **1** | **K.LFGGFNSSDTVTSPQR.A** |
|  | [2472](http://df3/mascot/cgi/peptide_view.pl?file=../data/20121119/F002569.dat&query=2472&hit=1&index=gi%7c00000001&px=1) | **857.3555** | **1712.6964** | **1711.8165** | **0.8799** | **0** | **(102)** | **5e-011** | **1** | **K.LFGGFNSSDTVTSPQR.A** |
|  | [2473](http://df3/mascot/cgi/peptide_view.pl?file=../data/20121119/F002569.dat&query=2473&hit=1&index=gi%7c00000001&px=1) | **857.3625** | **1712.7105** | **1711.8165** | **0.8940** | **0** | **(114)** | **3.3e-012** | **1** | **K.LFGGFNSSDTVTSPQR.A** |
|  | [2474](http://df3/mascot/cgi/peptide_view.pl?file=../data/20121119/F002569.dat&query=2474&hit=1&index=gi%7c00000001&px=1) | **857.3640** | **1712.7134** | **1711.8165** | **0.8968** | **0** | **(110)** | **7.4e-012** | **1** | **K.LFGGFNSSDTVTSPQR.A** |
|  | [2475](http://df3/mascot/cgi/peptide_view.pl?file=../data/20121119/F002569.dat&query=2475&hit=1&index=gi%7c00000001&px=1) | **857.3681** | **1712.7217** | **1711.8165** | **0.9051** | **0** | **(103)** | **4.4e-011** | **1** | **K.LFGGFNSSDTVTSPQR.A** |
|  | [2476](http://df3/mascot/cgi/peptide_view.pl?file=../data/20121119/F002569.dat&query=2476&hit=1&index=gi%7c00000001&px=1) | **857.3715** | **1712.7284** | **1711.8165** | **0.9119** | **0** | **(108)** | **1.3e-011** | **1** | **K.LFGGFNSSDTVTSPQR.A** |
|  | [2477](http://df3/mascot/cgi/peptide_view.pl?file=../data/20121119/F002569.dat&query=2477&hit=1&index=gi%7c00000001&px=1) | **857.3774** | **1712.7402** | **1711.8165** | **0.9237** | **0** | **(108)** | **1.2e-011** | **1** | **K.LFGGFNSSDTVTSPQR.A** |
|  | [2478](http://df3/mascot/cgi/peptide_view.pl?file=../data/20121119/F002569.dat&query=2478&hit=1&index=gi%7c00000001&px=1) | **857.3795** | **1712.7444** | **1711.8165** | **0.9278** | **0** | **(105)** | **2.5e-011** | **1** | **K.LFGGFNSSDTVTSPQR.A** |
|  | [2479](http://df3/mascot/cgi/peptide_view.pl?file=../data/20121119/F002569.dat&query=2479&hit=1&index=gi%7c00000001&px=1) | **857.3810** | **1712.7475** | **1711.8165** | **0.9310** | **0** | **(95)** | **2.5e-010** | **1** | **K.LFGGFNSSDTVTSPQR.A** |
|  | [2480](http://df3/mascot/cgi/peptide_view.pl?file=../data/20121119/F002569.dat&query=2480&hit=1&index=gi%7c00000001&px=1) | **857.3817** | **1712.7488** | **1711.8165** | **0.9322** | **0** | **(119)** | **1.1e-012** | **1** | **K.LFGGFNSSDTVTSPQR.A** |
|  | [2481](http://df3/mascot/cgi/peptide_view.pl?file=../data/20121119/F002569.dat&query=2481&hit=1&index=gi%7c00000001&px=1) | **857.3926** | **1712.7706** | **1711.8165** | **0.9541** | **0** | **(118)** | **1.4e-012** | **1** | **K.LFGGFNSSDTVTSPQR.A** |
|  | [998](http://df3/mascot/cgi/peptide_view.pl?file=../data/20121119/F002569.dat&query=998&hit=1&index=gi%7c00000001&px=1) | **571.9382** | **1712.7929** | **1711.8165** | **0.9764** | **0** | **(67)** | **6e-008** | **1** | **K.LFGGFNSSDTVTSPQR.A** |
|  | [2482](http://df3/mascot/cgi/peptide_view.pl?file=../data/20121119/F002569.dat&query=2482&hit=1&index=gi%7c00000001&px=1) | **857.4199** | **1712.8253** | **1711.8165** | **1.0088** | **0** | **(114)** | **3.5e-012** | **1** | **K.LFGGFNSSDTVTSPQR.A** |
|  | [2483](http://df3/mascot/cgi/peptide_view.pl?file=../data/20121119/F002569.dat&query=2483&hit=1&index=gi%7c00000001&px=1) | **857.4218** | **1712.8291** | **1711.8165** | **1.0126** | **0** | **(93)** | **4.6e-010** | **1** | **K.LFGGFNSSDTVTSPQR.A** |
|  | [2484](http://df3/mascot/cgi/peptide_view.pl?file=../data/20121119/F002569.dat&query=2484&hit=1&index=gi%7c00000001&px=1) | **857.4234** | **1712.8322** | **1711.8165** | **1.0157** | **0** | **(120)** | **7.7e-013** | **1** | **K.LFGGFNSSDTVTSPQR.A** |
|  | [2485](http://df3/mascot/cgi/peptide_view.pl?file=../data/20121119/F002569.dat&query=2485&hit=1&index=gi%7c00000001&px=1) | **857.4241** | **1712.8337** | **1711.8165** | **1.0172** | **0** | **(100)** | **8.2e-011** | **1** | **K.LFGGFNSSDTVTSPQR.A** |
|  | [2486](http://df3/mascot/cgi/peptide_view.pl?file=../data/20121119/F002569.dat&query=2486&hit=1&index=gi%7c00000001&px=1) | **857.4253** | **1712.8360** | **1711.8165** | **1.0195** | **0** | **(121)** | **7.1e-013** | **1** | **K.LFGGFNSSDTVTSPQR.A** |
|  | [2487](http://df3/mascot/cgi/peptide_view.pl?file=../data/20121119/F002569.dat&query=2487&hit=1&index=gi%7c00000001&px=1) | **857.4310** | **1712.8475** | **1711.8165** | **1.0310** | **0** | **(96)** | **2e-010** | **1** | **K.LFGGFNSSDTVTSPQR.A** |
|  | [2488](http://df3/mascot/cgi/peptide_view.pl?file=../data/20121119/F002569.dat&query=2488&hit=1&index=gi%7c00000001&px=1) | **857.4374** | **1712.8602** | **1711.8165** | **1.0437** | **0** | **(106)** | **2.4e-011** | **1** | **K.LFGGFNSSDTVTSPQR.A** |
|  | [2489](http://df3/mascot/cgi/peptide_view.pl?file=../data/20121119/F002569.dat&query=2489&hit=1&index=gi%7c00000001&px=1) | **857.4493** | **1712.8840** | **1711.8165** | **1.0675** | **0** | **(88)** | **1.3e-009** | **1** | **K.LFGGFNSSDTVTSPQR.A** |
|  | [2490](http://df3/mascot/cgi/peptide_view.pl?file=../data/20121119/F002569.dat&query=2490&hit=1&index=gi%7c00000001&px=1) | **857.4586** | **1712.9026** | **1711.8165** | **1.0860** | **0** | **(101)** | **6.7e-011** | **1** | **K.LFGGFNSSDTVTSPQR.A** |
|  | [2491](http://df3/mascot/cgi/peptide_view.pl?file=../data/20121119/F002569.dat&query=2491&hit=1&index=gi%7c00000001&px=1) | **857.4638** | **1712.9131** | **1711.8165** | **1.0965** | **0** | **(90)** | **8.4e-010** | **1** | **K.LFGGFNSSDTVTSPQR.A** |
|  | [2492](http://df3/mascot/cgi/peptide_view.pl?file=../data/20121119/F002569.dat&query=2492&hit=1&index=gi%7c00000001&px=1) | **857.4678** | **1712.9210** | **1711.8165** | **1.1045** | **0** | **(103)** | **4e-011** | **1** | **K.LFGGFNSSDTVTSPQR.A** |
|  | [2493](http://df3/mascot/cgi/peptide_view.pl?file=../data/20121119/F002569.dat&query=2493&hit=1&index=gi%7c00000001&px=1) | **857.5132** | **1713.0118** | **1711.8165** | **1.1953** | **0** | **140** | **9.4e-015** | **1** | **K.LFGGFNSSDTVTSPQR.A** |
|  | [2494](http://df3/mascot/cgi/peptide_view.pl?file=../data/20121119/F002569.dat&query=2494&hit=1&index=gi%7c00000001&px=1) | **857.5231** | **1713.0316** | **1711.8165** | **1.2151** | **0** | **(91)** | **6.5e-010** | **1** | **K.LFGGFNSSDTVTSPQR.A** |
|  | [2495](http://df3/mascot/cgi/peptide_view.pl?file=../data/20121119/F002569.dat&query=2495&hit=1&index=gi%7c00000001&px=1) | **857.5325** | **1713.0504** | **1711.8165** | **1.2339** | **0** | **(90)** | **7.6e-010** | **1** | **K.LFGGFNSSDTVTSPQR.A** |
|  | [2496](http://df3/mascot/cgi/peptide_view.pl?file=../data/20121119/F002569.dat&query=2496&hit=1&index=gi%7c00000001&px=1) | **857.5608** | **1713.1070** | **1711.8165** | **1.2905** | **0** | **(107)** | **1.9e-011** | **1** | **K.LFGGFNSSDTVTSPQR.A** |
|  | [2497](http://df3/mascot/cgi/peptide_view.pl?file=../data/20121119/F002569.dat&query=2497&hit=1&index=gi%7c00000001&px=1) | **857.5682** | **1713.1218** | **1711.8165** | **1.3053** | **0** | **(85)** | **2.9e-009** | **1** | **K.LFGGFNSSDTVTSPQR.A** |
|  | [2498](http://df3/mascot/cgi/peptide_view.pl?file=../data/20121119/F002569.dat&query=2498&hit=1&index=gi%7c00000001&px=1) | **857.5778** | **1713.1410** | **1711.8165** | **1.3245** | **0** | **(86)** | **1.9e-009** | **1** | **K.LFGGFNSSDTVTSPQR.A** |
|  | [999](http://df3/mascot/cgi/peptide_view.pl?file=../data/20121119/F002569.dat&query=999&hit=1&index=gi%7c00000001&px=1) | **572.0544** | **1713.1415** | **1711.8165** | **1.3250** | **0** | **(63)** | **1.5e-007** | **1** | **K.LFGGFNSSDTVTSPQR.A** |
|  | [1000](http://df3/mascot/cgi/peptide_view.pl?file=../data/20121119/F002569.dat&query=1000&hit=1&index=gi%7c00000001&px=1) | **572.0605** | **1713.1596** | **1711.8165** | **1.3431** | **0** | **(72)** | **2.1e-008** | **1** | **K.LFGGFNSSDTVTSPQR.A** |
|  | [1001](http://df3/mascot/cgi/peptide_view.pl?file=../data/20121119/F002569.dat&query=1001&hit=1&index=gi%7c00000001&px=1) | **572.0786** | **1713.2140** | **1711.8165** | **1.3975** | **0** | **(41)** | **2.3e-005** | **1** | **K.LFGGFNSSDTVTSPQR.A** |
|  | [1002](http://df3/mascot/cgi/peptide_view.pl?file=../data/20121119/F002569.dat&query=1002&hit=1&index=gi%7c00000001&px=1) | **572.1398** | **1713.3975** | **1711.8165** | **1.5810** | **0** | **(61)** | **2.3e-007** | **1** | **K.LFGGFNSSDTVTSPQR.A** |
|  | [1003](http://df3/mascot/cgi/peptide_view.pl?file=../data/20121119/F002569.dat&query=1003&hit=1&index=gi%7c00000001&px=1) | **572.1540** | **1713.4401** | **1711.9733** | **1.4669** | **1** | **(36)** | **8.4e-005** | **1** | **R.LLLNAENPRGTFLVR.E** |
|  | [1004](http://df3/mascot/cgi/peptide_view.pl?file=../data/20121119/F002569.dat&query=1004&hit=1&index=gi%7c00000001&px=1) | **572.1555** | **1713.4445** | **1711.9733** | **1.4713** | **1** | **37** | **6.5e-005** | **1** | **R.LLLNAENPRGTFLVR.E** |
|  | [2499](http://df3/mascot/cgi/peptide_view.pl?file=../data/20121119/F002569.dat&query=2499&hit=1&index=gi%7c00000001&px=1) | **857.7314** | **1713.4483** | **1711.8165** | **1.6318** | **0** | **(103)** | **4.6e-011** | **1** | **K.LFGGFNSSDTVTSPQR.A** |
|  | [2500](http://df3/mascot/cgi/peptide_view.pl?file=../data/20121119/F002569.dat&query=2500&hit=1&index=gi%7c00000001&px=1) | **857.8226** | **1713.6307** | **1711.8165** | **1.8142** | **0** | **(94)** | **3.1e-010** | **1** | **K.LFGGFNSSDTVTSPQR.A** |
|  | [1005](http://df3/mascot/cgi/peptide_view.pl?file=../data/20121119/F002569.dat&query=1005&hit=1&index=gi%7c00000001&px=1) | **572.2260** | **1713.6560** | **1711.8165** | **1.8395** | **0** | **(54)** | **1.3e-006** | **1** | **K.LFGGFNSSDTVTSPQR.A** |
|  | [2501](http://df3/mascot/cgi/peptide_view.pl?file=../data/20121119/F002569.dat&query=2501&hit=1&index=gi%7c00000001&px=1) | **857.8795** | **1713.7445** | **1711.8165** | **1.9280** | **0** | **(95)** | **2.8e-010** | **1** | **K.LFGGFNSSDTVTSPQR.A** |
|  | [2502](http://df3/mascot/cgi/peptide_view.pl?file=../data/20121119/F002569.dat&query=2502&hit=1&index=gi%7c00000001&px=1) | **857.9128** | **1713.8110** | **1711.8165** | **1.9945** | **0** | **(110)** | **8.6e-012** | **1** | **K.LFGGFNSSDTVTSPQR.A** |
|  | [2503](http://df3/mascot/cgi/peptide_view.pl?file=../data/20121119/F002569.dat&query=2503&hit=1&index=gi%7c00000001&px=1) | **857.9232** | **1713.8319** | **1712.8005** | **1.0314** | **0** | **(74)** | **3.5e-008** | **1** | **K.LFGGFNSSDTVTSPQR.A + Deamidation (NQ)** |
|  | [2504](http://df3/mascot/cgi/peptide_view.pl?file=../data/20121119/F002569.dat&query=2504&hit=1&index=gi%7c00000001&px=1) | **857.9396** | **1713.8646** | **1712.8005** | **1.0641** | **0** | **(87)** | **1.7e-009** | **1** | **K.LFGGFNSSDTVTSPQR.A + Deamidation (NQ)** |
|  | [2505](http://df3/mascot/cgi/peptide_view.pl?file=../data/20121119/F002569.dat&query=2505&hit=1&index=gi%7c00000001&px=1) | **857.9680** | **1713.9214** | **1712.8005** | **1.1208** | **0** | **(83)** | **4.3e-009** | **1** | **K.LFGGFNSSDTVTSPQR.A + Deamidation (NQ)** |
|  | [2506](http://df3/mascot/cgi/peptide_view.pl?file=../data/20121119/F002569.dat&query=2506&hit=1&index=gi%7c00000001&px=1) | **857.9929** | **1713.9713** | **1712.8005** | **1.1708** | **0** | **(96)** | **2e-010** | **1** | **K.LFGGFNSSDTVTSPQR.A + Deamidation (NQ)** |
|  | [1007](http://df3/mascot/cgi/peptide_view.pl?file=../data/20121119/F002569.dat&query=1007&hit=1&index=gi%7c00000001&px=1) | **572.3337** | **1713.9794** | **1712.8005** | **1.1789** | **0** | **(76)** | **5.1e-009** | **1** | **K.LFGGFNSSDTVTSPQR.A + Deamidation (NQ)** |
|  | [2507](http://df3/mascot/cgi/peptide_view.pl?file=../data/20121119/F002569.dat&query=2507&hit=1&index=gi%7c00000001&px=1) | **858.0063** | **1713.9980** | **1712.8005** | **1.1975** | **0** | **(90)** | **8e-010** | **1** | **K.LFGGFNSSDTVTSPQR.A + Deamidation (NQ)** |
|  | [2508](http://df3/mascot/cgi/peptide_view.pl?file=../data/20121119/F002569.dat&query=2508&hit=1&index=gi%7c00000001&px=1) | **858.0122** | **1714.0099** | **1712.8005** | **1.2093** | **0** | **(120)** | **6.9e-013** | **1** | **K.LFGGFNSSDTVTSPQR.A + Deamidation (NQ)** |
|  | [2509](http://df3/mascot/cgi/peptide_view.pl?file=../data/20121119/F002569.dat&query=2509&hit=1&index=gi%7c00000001&px=1) | **858.0233** | **1714.0321** | **1712.8005** | **1.2316** | **0** | **(72)** | **4.9e-008** | **1** | **K.LFGGFNSSDTVTSPQR.A + Deamidation (NQ)** |
|  | [2510](http://df3/mascot/cgi/peptide_view.pl?file=../data/20121119/F002569.dat&query=2510&hit=1&index=gi%7c00000001&px=1) | **858.0728** | **1714.1311** | **1712.8005** | **1.3306** | **0** | **(84)** | **2.9e-009** | **1** | **K.LFGGFNSSDTVTSPQR.A + Deamidation (NQ)** |
|  | [2511](http://df3/mascot/cgi/peptide_view.pl?file=../data/20121119/F002569.dat&query=2511&hit=1&index=gi%7c00000001&px=1) | **858.3502** | **1714.6859** | **1712.8005** | **1.8854** | **0** | **(102)** | **4.3e-011** | **1** | **K.LFGGFNSSDTVTSPQR.A + Deamidation (NQ)** |
|  | [2512](http://df3/mascot/cgi/peptide_view.pl?file=../data/20121119/F002569.dat&query=2512&hit=1&index=gi%7c00000001&px=1) | **858.4548** | **1714.8951** | **1713.7845** | **1.1106** | **0** | **(82)** | **4.1e-009** | **1** | **K.LFGGFNSSDTVTSPQR.A + 2 Deamidation (NQ)** |
|  | [2513](http://df3/mascot/cgi/peptide_view.pl?file=../data/20121119/F002569.dat&query=2513&hit=1&index=gi%7c00000001&px=1) | **858.4617** | **1714.9088** | **1713.7845** | **1.1243** | **0** | **(88)** | **9.9e-010** | **1** | **K.LFGGFNSSDTVTSPQR.A + 2 Deamidation (NQ)** |
|  | [2515](http://df3/mascot/cgi/peptide_view.pl?file=../data/20121119/F002569.dat&query=2515&hit=1&index=gi%7c00000001&px=1) | **858.5239** | **1715.0333** | **1713.7845** | **1.2488** | **0** | **(93)** | **3.1e-010** | **1** | **K.LFGGFNSSDTVTSPQR.A + 2 Deamidation (NQ)** |
|  | [2516](http://df3/mascot/cgi/peptide_view.pl?file=../data/20121119/F002569.dat&query=2516&hit=1&index=gi%7c00000001&px=1) | **858.6077** | **1715.2008** | **1713.7845** | **1.4162** | **0** | **(98)** | **8.6e-011** | **1** | **K.LFGGFNSSDTVTSPQR.A + 2 Deamidation (NQ)** |
|  | [2517](http://df3/mascot/cgi/peptide_view.pl?file=../data/20121119/F002569.dat&query=2517&hit=1&index=gi%7c00000001&px=1) | **858.6101** | **1715.2057** | **1713.7845** | **1.4211** | **0** | **(95)** | **2e-010** | **1** | **K.LFGGFNSSDTVTSPQR.A + 2 Deamidation (NQ)** |
|  | [2522](http://df3/mascot/cgi/peptide_view.pl?file=../data/20121119/F002569.dat&query=2522&hit=1&index=gi%7c00000001&px=1) | **860.5399** | **1719.0652** | **1718.9144** | **0.1508** | **1** | **99** | **3.3e-011** | **1** | **K.FPIKWTAPEAALYGR.F** |
|  | [1015](http://df3/mascot/cgi/peptide_view.pl?file=../data/20121119/F002569.dat&query=1015&hit=1&index=gi%7c00000001&px=1) | **574.0848** | **1719.2327** | **1718.9144** | **0.3183** | **1** | **(47)** | **9.6e-007** | **1** | **K.FPIKWTAPEAALYGR.F** |
|  | [1016](http://df3/mascot/cgi/peptide_view.pl?file=../data/20121119/F002569.dat&query=1016&hit=1&index=gi%7c00000001&px=1) | **574.2278** | **1719.6615** | **1718.9144** | **0.7472** | **1** | **(60)** | **4.8e-008** | **1** | **K.FPIKWTAPEAALYGR.F** |
|  | [2524](http://df3/mascot/cgi/peptide_view.pl?file=../data/20121119/F002569.dat&query=2524&hit=1&index=gi%7c00000001&px=1) | **860.8983** | **1719.7821** | **1718.9144** | **0.8677** | **1** | **(78)** | **4e-009** | **1** | **K.FPIKWTAPEAALYGR.F** |
|  | [1017](http://df3/mascot/cgi/peptide_view.pl?file=../data/20121119/F002569.dat&query=1017&hit=1&index=gi%7c00000001&px=1) | **574.3997** | **1720.1771** | **1718.9144** | **1.2628** | **1** | **(78)** | **8e-010** | **1** | **K.FPIKWTAPEAALYGR.F** |
|  | [1018](http://df3/mascot/cgi/peptide_view.pl?file=../data/20121119/F002569.dat&query=1018&hit=1&index=gi%7c00000001&px=1) | **574.4329** | **1720.2769** | **1718.9144** | **1.3626** | **1** | **(64)** | **2.2e-008** | **1** | **K.FPIKWTAPEAALYGR.F** |
|  | [1019](http://df3/mascot/cgi/peptide_view.pl?file=../data/20121119/F002569.dat&query=1019&hit=1&index=gi%7c00000001&px=1) | **574.4470** | **1720.3192** | **1718.9144** | **1.4049** | **1** | **(62)** | **2.9e-008** | **1** | **K.FPIKWTAPEAALYGR.F** |
|  | [1104](http://df3/mascot/cgi/peptide_view.pl?file=../data/20121119/F002569.dat&query=1104&hit=1&index=gi%7c00000001&px=1) | **592.0341** | **1773.0803** | **1771.9502** | **1.1302** | **0** | **(27)** | **0.00028** | **1** | **R.LTTVCPTSKPQTQGLAK.D** |
|  | [1149](http://df3/mascot/cgi/peptide_view.pl?file=../data/20121119/F002569.dat&query=1149&hit=1&index=gi%7c00000001&px=1) | **595.8918** | **1784.6535** | **1783.9614** | **0.6921** | **1** | **49** | **1.8e-006** | **1** | **R.DLRAANILVGENLVCK.V + Carbamidomethyl (C)** |
|  | [2720](http://df3/mascot/cgi/peptide_view.pl?file=../data/20121119/F002569.dat&query=2720&hit=1&index=gi%7c00000001&px=1) | **894.7627** | **1787.5108** | **1788.9046** | **-1.3938** | **0** | **(87)** | **1.3e-009** | **1** | **R.TQFNSLQQLVAYYSK.H** |
|  | [2721](http://df3/mascot/cgi/peptide_view.pl?file=../data/20121119/F002569.dat&query=2721&hit=1&index=gi%7c00000001&px=1) | **895.2770** | **1788.5394** | **1788.9046** | **-0.3652** | **0** | **(28)** | **0.00082** | **1** | **R.TQFNSLQQLVAYYSK.H** |
|  | [2722](http://df3/mascot/cgi/peptide_view.pl?file=../data/20121119/F002569.dat&query=2722&hit=1&index=gi%7c00000001&px=1) | **895.3251** | **1788.6356** | **1788.9046** | **-0.2690** | **0** | **(91)** | **3.9e-010** | **1** | **R.TQFNSLQQLVAYYSK.H** |
|  | [2723](http://df3/mascot/cgi/peptide_view.pl?file=../data/20121119/F002569.dat&query=2723&hit=1&index=gi%7c00000001&px=1) | **895.4171** | **1788.8197** | **1788.9046** | **-0.0849** | **0** | **108** | **9.7e-012** | **1** | **R.TQFNSLQQLVAYYSK.H** |
|  | [2724](http://df3/mascot/cgi/peptide_view.pl?file=../data/20121119/F002569.dat&query=2724&hit=1&index=gi%7c00000001&px=1) | **895.4440** | **1788.8734** | **1788.9046** | **-0.0312** | **0** | **(95)** | **1.5e-010** | **1** | **R.TQFNSLQQLVAYYSK.H** |
|  | [2725](http://df3/mascot/cgi/peptide_view.pl?file=../data/20121119/F002569.dat&query=2725&hit=1&index=gi%7c00000001&px=1) | **895.4670** | **1788.9195** | **1788.9046** | **0.0149** | **0** | **(82)** | **2.9e-009** | **1** | **R.TQFNSLQQLVAYYSK.H** |
|  | [2726](http://df3/mascot/cgi/peptide_view.pl?file=../data/20121119/F002569.dat&query=2726&hit=1&index=gi%7c00000001&px=1) | **895.4736** | **1788.9327** | **1788.9046** | **0.0281** | **0** | **(87)** | **9e-010** | **1** | **R.TQFNSLQQLVAYYSK.H** |
|  | [2727](http://df3/mascot/cgi/peptide_view.pl?file=../data/20121119/F002569.dat&query=2727&hit=1&index=gi%7c00000001&px=1) | **895.4948** | **1788.9750** | **1788.9046** | **0.0703** | **0** | **(95)** | **1.5e-010** | **1** | **R.TQFNSLQQLVAYYSK.H** |
|  | [2728](http://df3/mascot/cgi/peptide_view.pl?file=../data/20121119/F002569.dat&query=2728&hit=1&index=gi%7c00000001&px=1) | **895.6890** | **1789.3634** | **1788.9046** | **0.4588** | **0** | **(93)** | **2.4e-010** | **1** | **R.TQFNSLQQLVAYYSK.H** |
|  | [2729](http://df3/mascot/cgi/peptide_view.pl?file=../data/20121119/F002569.dat&query=2729&hit=1&index=gi%7c00000001&px=1) | **895.8110** | **1789.6075** | **1788.9046** | **0.7029** | **0** | **(86)** | **1.2e-009** | **1** | **R.TQFNSLQQLVAYYSK.H** |
|  | [2730](http://df3/mascot/cgi/peptide_view.pl?file=../data/20121119/F002569.dat&query=2730&hit=1&index=gi%7c00000001&px=1) | **895.8477** | **1789.6808** | **1788.9046** | **0.7762** | **0** | **(83)** | **2.2e-009** | **1** | **R.TQFNSLQQLVAYYSK.H** |
|  | [1161](http://df3/mascot/cgi/peptide_view.pl?file=../data/20121119/F002569.dat&query=1161&hit=1&index=gi%7c00000001&px=1) | **597.6059** | **1789.7959** | **1788.9046** | **0.8913** | **0** | **(70)** | **1.4e-008** | **1** | **R.TQFNSLQQLVAYYSK.H** |
|  | [1162](http://df3/mascot/cgi/peptide_view.pl?file=../data/20121119/F002569.dat&query=1162&hit=1&index=gi%7c00000001&px=1) | **597.6832** | **1790.0277** | **1788.9046** | **1.1231** | **0** | **(80)** | **2.2e-009** | **1** | **R.TQFNSLQQLVAYYSK.H** |
|  | [1163](http://df3/mascot/cgi/peptide_view.pl?file=../data/20121119/F002569.dat&query=1163&hit=1&index=gi%7c00000001&px=1) | **597.6951** | **1790.0634** | **1788.9046** | **1.1588** | **0** | **(96)** | **4.8e-011** | **1** | **R.TQFNSLQQLVAYYSK.H** |
|  | [1164](http://df3/mascot/cgi/peptide_view.pl?file=../data/20121119/F002569.dat&query=1164&hit=1&index=gi%7c00000001&px=1) | **597.6975** | **1790.0707** | **1788.9046** | **1.1661** | **0** | **(85)** | **6.3e-010** | **1** | **R.TQFNSLQQLVAYYSK.H** |
|  | [1165](http://df3/mascot/cgi/peptide_view.pl?file=../data/20121119/F002569.dat&query=1165&hit=1&index=gi%7c00000001&px=1) | **597.7917** | **1790.3534** | **1788.9046** | **1.4488** | **0** | **(77)** | **3.8e-009** | **1** | **R.TQFNSLQQLVAYYSK.H** |
|  | [2733](http://df3/mascot/cgi/peptide_view.pl?file=../data/20121119/F002569.dat&query=2733&hit=1&index=gi%7c00000001&px=1) | **896.3142** | **1790.6139** | **1789.8886** | **0.7253** | **0** | **(90)** | **4.9e-010** | **1** | **R.TQFNSLQQLVAYYSK.H + Deamidation (NQ)** |
|  | [2734](http://df3/mascot/cgi/peptide_view.pl?file=../data/20121119/F002569.dat&query=2734&hit=1&index=gi%7c00000001&px=1) | **896.3246** | **1790.6346** | **1788.9046** | **1.7300** | **0** | **(90)** | **5.3e-010** | **1** | **R.TQFNSLQQLVAYYSK.H** |
|  | [2735](http://df3/mascot/cgi/peptide_view.pl?file=../data/20121119/F002569.dat&query=2735&hit=1&index=gi%7c00000001&px=1) | **896.3282** | **1790.6419** | **1788.9046** | **1.7373** | **0** | **(38)** | **8e-005** | **1** | **R.TQFNSLQQLVAYYSK.H** |
|  | [2736](http://df3/mascot/cgi/peptide_view.pl?file=../data/20121119/F002569.dat&query=2736&hit=1&index=gi%7c00000001&px=1) | **896.4188** | **1790.8231** | **1789.8886** | **0.9345** | **0** | **(83)** | **2.5e-009** | **1** | **R.TQFNSLQQLVAYYSK.H + Deamidation (NQ)** |
|  | [1166](http://df3/mascot/cgi/peptide_view.pl?file=../data/20121119/F002569.dat&query=1166&hit=1&index=gi%7c00000001&px=1) | **597.9519** | **1790.8339** | **1788.9046** | **1.9293** | **0** | **(63)** | **9e-008** | **1** | **R.TQFNSLQQLVAYYSK.H** |
|  | [2737](http://df3/mascot/cgi/peptide_view.pl?file=../data/20121119/F002569.dat&query=2737&hit=1&index=gi%7c00000001&px=1) | **896.4709** | **1790.9273** | **1789.8886** | **1.0387** | **0** | **(65)** | **1.7e-007** | **1** | **R.TQFNSLQQLVAYYSK.H + Deamidation (NQ)** |
|  | [2738](http://df3/mascot/cgi/peptide_view.pl?file=../data/20121119/F002569.dat&query=2738&hit=1&index=gi%7c00000001&px=1) | **896.4789** | **1790.9432** | **1789.8886** | **1.0546** | **0** | **(87)** | **1.1e-009** | **1** | **R.TQFNSLQQLVAYYSK.H + Deamidation (NQ)** |
|  | [2739](http://df3/mascot/cgi/peptide_view.pl?file=../data/20121119/F002569.dat&query=2739&hit=1&index=gi%7c00000001&px=1) | **896.5441** | **1791.0736** | **1789.8886** | **1.1850** | **0** | **(68)** | **7.3e-008** | **1** | **R.TQFNSLQQLVAYYSK.H + Deamidation (NQ)** |
|  | [2741](http://df3/mascot/cgi/peptide_view.pl?file=../data/20121119/F002569.dat&query=2741&hit=1&index=gi%7c00000001&px=1) | **896.7118** | **1791.4090** | **1789.8886** | **1.5204** | **0** | **(74)** | **2.1e-008** | **1** | **R.TQFNSLQQLVAYYSK.H + Deamidation (NQ)** |
|  | [1168](http://df3/mascot/cgi/peptide_view.pl?file=../data/20121119/F002569.dat&query=1168&hit=1&index=gi%7c00000001&px=1) | **598.1725** | **1791.4956** | **1789.8886** | **1.6070** | **0** | **(84)** | **7.1e-010** | **1** | **R.TQFNSLQQLVAYYSK.H + Deamidation (NQ)** |
|  | [2742](http://df3/mascot/cgi/peptide_view.pl?file=../data/20121119/F002569.dat&query=2742&hit=1&index=gi%7c00000001&px=1) | **896.8068** | **1791.5990** | **1789.8886** | **1.7104** | **0** | **(31)** | **0.00033** | **1** | **R.TQFNSLQQLVAYYSK.H + Deamidation (NQ)** |
|  | [2744](http://df3/mascot/cgi/peptide_view.pl?file=../data/20121119/F002569.dat&query=2744&hit=1&index=gi%7c00000001&px=1) | **896.9482** | **1791.8819** | **1789.8886** | **1.9933** | **0** | **(86)** | **1.1e-009** | **1** | **R.TQFNSLQQLVAYYSK.H + Deamidation (NQ)** |
|  | [2745](http://df3/mascot/cgi/peptide_view.pl?file=../data/20121119/F002569.dat&query=2745&hit=1&index=gi%7c00000001&px=1) | **897.0759** | **1792.1373** | **1790.8726** | **1.2647** | **0** | **(63)** | **1.9e-007** | **1** | **R.TQFNSLQQLVAYYSK.H + 2 Deamidation (NQ)** |
|  | [1171](http://df3/mascot/cgi/peptide_view.pl?file=../data/20121119/F002569.dat&query=1171&hit=1&index=gi%7c00000001&px=1) | **598.4594** | **1792.3562** | **1790.8726** | **1.4836** | **0** | **(67)** | **2.7e-008** | **1** | **R.TQFNSLQQLVAYYSK.H + 2 Deamidation (NQ)** |
|  | [1212](http://df3/mascot/cgi/peptide_view.pl?file=../data/20121119/F002569.dat&query=1212&hit=1&index=gi%7c00000001&px=1) | **610.2113** | **1827.6121** | **1828.9717** | **-1.3596** | **0** | **(28)** | **0.00042** | **1** | **R.LTTVCPTSKPQTQGLAK.D + Carbamidomethyl (C)** |
|  | [2780](http://df3/mascot/cgi/peptide_view.pl?file=../data/20121119/F002569.dat&query=2780&hit=1&index=gi%7c00000001&px=1) | **915.0437** | **1828.0729** | **1828.9717** | **-0.8988** | **0** | **86** | **8.9e-010** | **1** | **R.LTTVCPTSKPQTQGLAK.D + Carbamidomethyl (C)** |
|  | [1214](http://df3/mascot/cgi/peptide_view.pl?file=../data/20121119/F002569.dat&query=1214&hit=1&index=gi%7c00000001&px=1) | **610.3782** | **1828.1129** | **1828.9717** | **-0.8588** | **0** | **(32)** | **0.00016** | **1** | **R.LTTVCPTSKPQTQGLAK.D + Carbamidomethyl (C)** |
|  | [1215](http://df3/mascot/cgi/peptide_view.pl?file=../data/20121119/F002569.dat&query=1215&hit=1&index=gi%7c00000001&px=1) | **610.5305** | **1828.5695** | **1829.9557** | **-1.3861** | **0** | **(14)** | **0.01** | **1** | **R.LTTVCPTSKPQTQGLAK.D + Carbamidomethyl (C); Deamidation (NQ)** |
|  | [1216](http://df3/mascot/cgi/peptide_view.pl?file=../data/20121119/F002569.dat&query=1216&hit=1&index=gi%7c00000001&px=1) | **610.5869** | **1828.7389** | **1829.9557** | **-1.2168** | **0** | **(15)** | **0.0088** | **1** | **R.LTTVCPTSKPQTQGLAK.D + Carbamidomethyl (C); Deamidation (NQ)** |
|  | [2781](http://df3/mascot/cgi/peptide_view.pl?file=../data/20121119/F002569.dat&query=2781&hit=1&index=gi%7c00000001&px=1) | **915.4446** | **1828.8746** | **1828.9717** | **-0.0971** | **0** | **(46)** | **9.4e-006** | **1** | **R.LTTVCPTSKPQTQGLAK.D + Carbamidomethyl (C)** |
|  | [1219](http://df3/mascot/cgi/peptide_view.pl?file=../data/20121119/F002569.dat&query=1219&hit=1&index=gi%7c00000001&px=1) | **610.8674** | **1829.5803** | **1829.9557** | **-0.3754** | **0** | **(15)** | **0.0075** | **1** | **R.LTTVCPTSKPQTQGLAK.D + Carbamidomethyl (C); Deamidation (NQ)** |
|  | [1220](http://df3/mascot/cgi/peptide_view.pl?file=../data/20121119/F002569.dat&query=1220&hit=1&index=gi%7c00000001&px=1) | **610.8674** | **1829.5805** | **1829.9557** | **-0.3752** | **0** | **(23)** | **0.0012** | **1** | **R.LTTVCPTSKPQTQGLAK.D + Carbamidomethyl (C); Deamidation (NQ)** |
|  | [1221](http://df3/mascot/cgi/peptide_view.pl?file=../data/20121119/F002569.dat&query=1221&hit=1&index=gi%7c00000001&px=1) | **610.8767** | **1829.6083** | **1829.9557** | **-0.3474** | **0** | **(22)** | **0.0016** | **1** | **R.LTTVCPTSKPQTQGLAK.D + Carbamidomethyl (C); Deamidation (NQ)** |
|  | [2782](http://df3/mascot/cgi/peptide_view.pl?file=../data/20121119/F002569.dat&query=2782&hit=1&index=gi%7c00000001&px=1) | **915.8296** | **1829.6446** | **1829.9557** | **-0.3110** | **0** | **(73)** | **2.1e-008** | **1** | **R.LTTVCPTSKPQTQGLAK.D + Carbamidomethyl (C); Deamidation (NQ)** |
|  | [2783](http://df3/mascot/cgi/peptide_view.pl?file=../data/20121119/F002569.dat&query=2783&hit=1&index=gi%7c00000001&px=1) | **915.8782** | **1829.7418** | **1828.9717** | **0.7701** | **0** | **(72)** | **2.6e-008** | **1** | **R.LTTVCPTSKPQTQGLAK.D + Carbamidomethyl (C)** |
|  | [1222](http://df3/mascot/cgi/peptide_view.pl?file=../data/20121119/F002569.dat&query=1222&hit=1&index=gi%7c00000001&px=1) | **610.9306** | **1829.7700** | **1829.9557** | **-0.1857** | **0** | **(15)** | **0.0083** | **1** | **R.LTTVCPTSKPQTQGLAK.D + Carbamidomethyl (C); Deamidation (NQ)** |
|  | [1223](http://df3/mascot/cgi/peptide_view.pl?file=../data/20121119/F002569.dat&query=1223&hit=1&index=gi%7c00000001&px=1) | **610.9368** | **1829.7887** | **1828.9717** | **0.8170** | **0** | **(20)** | **0.0026** | **1** | **R.LTTVCPTSKPQTQGLAK.D + Carbamidomethyl (C)** |
|  | [1224](http://df3/mascot/cgi/peptide_view.pl?file=../data/20121119/F002569.dat&query=1224&hit=1&index=gi%7c00000001&px=1) | **610.9396** | **1829.7969** | **1829.9557** | **-0.1588** | **0** | **(14)** | **0.011** | **1** | **R.LTTVCPTSKPQTQGLAK.D + Carbamidomethyl (C); Deamidation (NQ)** |
|  | [1225](http://df3/mascot/cgi/peptide_view.pl?file=../data/20121119/F002569.dat&query=1225&hit=1&index=gi%7c00000001&px=1) | **610.9477** | **1829.8213** | **1829.9557** | **-0.1344** | **0** | **(14)** | **0.0096** | **1** | **R.LTTVCPTSKPQTQGLAK.D + Carbamidomethyl (C); Deamidation (NQ)** |
|  | [1226](http://df3/mascot/cgi/peptide_view.pl?file=../data/20121119/F002569.dat&query=1226&hit=1&index=gi%7c00000001&px=1) | **610.9688** | **1829.8844** | **1829.9557** | **-0.0713** | **0** | **(13)** | **0.011** | **1** | **R.LTTVCPTSKPQTQGLAK.D + Carbamidomethyl (C); Deamidation (NQ)** |
|  | [1227](http://df3/mascot/cgi/peptide_view.pl?file=../data/20121119/F002569.dat&query=1227&hit=1&index=gi%7c00000001&px=1) | **610.9900** | **1829.9481** | **1829.9557** | **-0.0075** | **0** | **(21)** | **0.002** | **1** | **R.LTTVCPTSKPQTQGLAK.D + Carbamidomethyl (C); Deamidation (NQ)** |
|  | [1228](http://df3/mascot/cgi/peptide_view.pl?file=../data/20121119/F002569.dat&query=1228&hit=1&index=gi%7c00000001&px=1) | **611.0532** | **1830.1378** | **1828.9717** | **1.1662** | **0** | **(23)** | **0.0013** | **1** | **R.LTTVCPTSKPQTQGLAK.D + Carbamidomethyl (C)** |
|  | [1229](http://df3/mascot/cgi/peptide_view.pl?file=../data/20121119/F002569.dat&query=1229&hit=1&index=gi%7c00000001&px=1) | **611.0542** | **1830.1408** | **1828.9717** | **1.1691** | **0** | **(10)** | **0.024** | **1** | **R.LTTVCPTSKPQTQGLAK.D + Carbamidomethyl (C)** |
|  | [2784](http://df3/mascot/cgi/peptide_view.pl?file=../data/20121119/F002569.dat&query=2784&hit=1&index=gi%7c00000001&px=1) | **916.0935** | **1830.1725** | **1828.9717** | **1.2008** | **0** | **(83)** | **1.8e-009** | **1** | **R.LTTVCPTSKPQTQGLAK.D + Carbamidomethyl (C)** |
|  | [1231](http://df3/mascot/cgi/peptide_view.pl?file=../data/20121119/F002569.dat&query=1231&hit=1&index=gi%7c00000001&px=1) | **611.2593** | **1830.7560** | **1828.9717** | **1.7843** | **0** | **(17)** | **0.0046** | **1** | **R.LTTVCPTSKPQTQGLAK.D + Carbamidomethyl (C)** |
|  | [1232](http://df3/mascot/cgi/peptide_view.pl?file=../data/20121119/F002569.dat&query=1232&hit=1&index=gi%7c00000001&px=1) | **611.6581** | **1831.9526** | **1829.9557** | **1.9969** | **0** | **(33)** | **0.00011** | **1** | **R.LTTVCPTSKPQTQGLAK.D + Carbamidomethyl (C); Deamidation (NQ)** |
|  | [1444](http://df3/mascot/cgi/peptide_view.pl?file=../data/20121119/F002569.dat&query=1444&hit=1&index=gi%7c00000001&px=1) | **667.6910** | **2000.0513** | **2000.0149** | **0.0364** | **1** | **36** | **6.9e-005** | **1** | **R.VPYPGMVNREVLDQVER.G** |
|  | [1472](http://df3/mascot/cgi/peptide_view.pl?file=../data/20121119/F002569.dat&query=1472&hit=1&index=gi%7c00000001&px=1) | **673.3644** | **2017.0715** | **2016.0098** | **1.0617** | **1** | **(27)** | **0.00063** | **1** | **R.VPYPGMVNREVLDQVER.G + Oxidation (M)** |
|  | [1473](http://df3/mascot/cgi/peptide_view.pl?file=../data/20121119/F002569.dat&query=1473&hit=1&index=gi%7c00000001&px=1) | **673.4668** | **2017.3786** | **2016.0098** | **1.3688** | **1** | **(33)** | **0.00014** | **1** | **R.VPYPGMVNREVLDQVER.G + Oxidation (M)** |
|  | [1475](http://df3/mascot/cgi/peptide_view.pl?file=../data/20121119/F002569.dat&query=1475&hit=1&index=gi%7c00000001&px=1) | **673.6189** | **2017.8349** | **2016.0098** | **1.8251** | **1** | **(34)** | **8.9e-005** | **1** | **R.VPYPGMVNREVLDQVER.G + Oxidation (M)** |
|  | [1521](http://df3/mascot/cgi/peptide_view.pl?file=../data/20121119/F002569.dat&query=1521&hit=1&index=gi%7c00000001&px=1) | **686.9508** | **2057.8306** | **2056.9887** | **0.8419** | **1** | **61** | **1.7e-007** | **1** | **K.GAYCLSVSDFDNAKGLNVK.H + Carbamidomethyl (C)** |
|  | [1524](http://df3/mascot/cgi/peptide_view.pl?file=../data/20121119/F002569.dat&query=1524&hit=1&index=gi%7c00000001&px=1) | **687.3584** | **2059.0534** | **2057.9727** | **1.0806** | **1** | **(60)** | **9.7e-008** | **1** | **K.GAYCLSVSDFDNAKGLNVK.H + Carbamidomethyl (C); Deamidation (NQ)** |
|  | [1551](http://df3/mascot/cgi/peptide_view.pl?file=../data/20121119/F002569.dat&query=1551&hit=1&index=gi%7c00000001&px=1) | **696.4525** | **2086.3357** | **2086.0370** | **0.2987** | **0** | **28** | **7.8e-005** | **1** | **R.AGPLAGGVTTFVALYDYESR.T** |
|  | [1554](http://df3/mascot/cgi/peptide_view.pl?file=../data/20121119/F002569.dat&query=1554&hit=1&index=gi%7c00000001&px=1) | **696.7629** | **2087.2670** | **2086.0370** | **1.2300** | **0** | **(26)** | **0.00012** | **1** | **R.AGPLAGGVTTFVALYDYESR.T** |
|  | [2841](http://df3/mascot/cgi/peptide_view.pl?file=../data/20121119/F002569.dat&query=2841&hit=1&index=gi%7c00000001&px=1) | **1086.9824** | **2171.9503** | **2171.9516** | **-0.0013** | **0** | **(106)** | **1e-011** | **1** | **K.LGQGCFGEVWMGTWNGTTR.V + Carbamidomethyl (C); Oxidation (M)** |
|  | [1654](http://df3/mascot/cgi/peptide_view.pl?file=../data/20121119/F002569.dat&query=1654&hit=1&index=gi%7c00000001&px=1) | **725.1686** | **2172.4841** | **2171.9516** | **0.5325** | **0** | **(50)** | **3e-006** | **1** | **K.LGQGCFGEVWMGTWNGTTR.V + Carbamidomethyl (C); Oxidation (M)** |
|  | [1655](http://df3/mascot/cgi/peptide_view.pl?file=../data/20121119/F002569.dat&query=1655&hit=1&index=gi%7c00000001&px=1) | **725.4063** | **2173.1969** | **2171.9516** | **1.2453** | **0** | **(53)** | **1.5e-006** | **1** | **K.LGQGCFGEVWMGTWNGTTR.V + Carbamidomethyl (C); Oxidation (M)** |
|  | [1656](http://df3/mascot/cgi/peptide_view.pl?file=../data/20121119/F002569.dat&query=1656&hit=1&index=gi%7c00000001&px=1) | **725.4327** | **2173.2764** | **2171.9516** | **1.3248** | **0** | **(41)** | **2.2e-005** | **1** | **K.LGQGCFGEVWMGTWNGTTR.V + Carbamidomethyl (C); Oxidation (M)** |
|  | [2842](http://df3/mascot/cgi/peptide_view.pl?file=../data/20121119/F002569.dat&query=2842&hit=1&index=gi%7c00000001&px=1) | **1087.7852** | **2173.5558** | **2171.9516** | **1.6041** | **0** | **(105)** | **8.7e-012** | **1** | **K.LGQGCFGEVWMGTWNGTTR.V + Carbamidomethyl (C); Oxidation (M)** |
|  | [2843](http://df3/mascot/cgi/peptide_view.pl?file=../data/20121119/F002569.dat&query=2843&hit=1&index=gi%7c00000001&px=1) | **1087.8414** | **2173.6683** | **2172.9356** | **0.7327** | **0** | **113** | **1.5e-012** | **1** | **K.LGQGCFGEVWMGTWNGTTR.V + Carbamidomethyl (C); Deamidation (NQ); Oxidation (M)** |
|  | [2844](http://df3/mascot/cgi/peptide_view.pl?file=../data/20121119/F002569.dat&query=2844&hit=1&index=gi%7c00000001&px=1) | **1087.8423** | **2173.6700** | **2172.9356** | **0.7344** | **0** | **(102)** | **1.8e-011** | **1** | **K.LGQGCFGEVWMGTWNGTTR.V + Carbamidomethyl (C); Deamidation (NQ); Oxidation (M)** |
|  | [1657](http://df3/mascot/cgi/peptide_view.pl?file=../data/20121119/F002569.dat&query=1657&hit=1&index=gi%7c00000001&px=1) | **725.7700** | **2174.2882** | **2172.9356** | **1.3526** | **0** | **(61)** | **1.7e-007** | **1** | **K.LGQGCFGEVWMGTWNGTTR.V + Carbamidomethyl (C); Deamidation (NQ); Oxidation (M)** |
|  | [1663](http://df3/mascot/cgi/peptide_view.pl?file=../data/20121119/F002569.dat&query=1663&hit=1&index=gi%7c00000001&px=1) | **726.8452** | **2177.5136** | **2178.0812** | **-0.5676** | **0** | **(60)** | **1.1e-007** | **1** | **R.LPQLVDMAAQIASGMAYVER.M + Oxidation (M)** |
|  | [2846](http://df3/mascot/cgi/peptide_view.pl?file=../data/20121119/F002569.dat&query=2846&hit=1&index=gi%7c00000001&px=1) | **1089.8688** | **2177.7230** | **2178.0812** | **-0.3582** | **0** | **(125)** | **3e-014** | **1** | **R.LPQLVDMAAQIASGMAYVER.M + Oxidation (M)** |
|  | [2847](http://df3/mascot/cgi/peptide_view.pl?file=../data/20121119/F002569.dat&query=2847&hit=1&index=gi%7c00000001&px=1) | **1089.9465** | **2177.8785** | **2178.0812** | **-0.2027** | **0** | **(139)** | **1.2e-015** | **1** | **R.LPQLVDMAAQIASGMAYVER.M + Oxidation (M)** |
|  | [2848](http://df3/mascot/cgi/peptide_view.pl?file=../data/20121119/F002569.dat&query=2848&hit=1&index=gi%7c00000001&px=1) | **1089.9573** | **2177.9000** | **2178.0812** | **-0.1812** | **0** | **(123)** | **5e-014** | **1** | **R.LPQLVDMAAQIASGMAYVER.M + Oxidation (M)** |
|  | [2849](http://df3/mascot/cgi/peptide_view.pl?file=../data/20121119/F002569.dat&query=2849&hit=1&index=gi%7c00000001&px=1) | **1089.9612** | **2177.9078** | **2178.0812** | **-0.1734** | **0** | **(142)** | **6.6e-016** | **1** | **R.LPQLVDMAAQIASGMAYVER.M + Oxidation (M)** |
|  | [2850](http://df3/mascot/cgi/peptide_view.pl?file=../data/20121119/F002569.dat&query=2850&hit=1&index=gi%7c00000001&px=1) | **1089.9736** | **2177.9327** | **2178.0812** | **-0.1485** | **0** | **(141)** | **8e-016** | **1** | **R.LPQLVDMAAQIASGMAYVER.M + Oxidation (M)** |
|  | [2851](http://df3/mascot/cgi/peptide_view.pl?file=../data/20121119/F002569.dat&query=2851&hit=1&index=gi%7c00000001&px=1) | **1090.0078** | **2178.0011** | **2178.0812** | **-0.0802** | **0** | **158** | **1.6e-017** | **1** | **R.LPQLVDMAAQIASGMAYVER.M + Oxidation (M)** |
|  | [2852](http://df3/mascot/cgi/peptide_view.pl?file=../data/20121119/F002569.dat&query=2852&hit=1&index=gi%7c00000001&px=1) | **1090.0114** | **2178.0082** | **2178.0812** | **-0.0731** | **0** | **(146)** | **2.5e-016** | **1** | **R.LPQLVDMAAQIASGMAYVER.M + Oxidation (M)** |
|  | [1664](http://df3/mascot/cgi/peptide_view.pl?file=../data/20121119/F002569.dat&query=1664&hit=1&index=gi%7c00000001&px=1) | **727.0201** | **2178.0384** | **2178.0812** | **-0.0428** | **0** | **(89)** | **1.4e-010** | **1** | **R.LPQLVDMAAQIASGMAYVER.M + Oxidation (M)** |
|  | [1665](http://df3/mascot/cgi/peptide_view.pl?file=../data/20121119/F002569.dat&query=1665&hit=1&index=gi%7c00000001&px=1) | **727.0599** | **2178.1580** | **2178.0812** | **0.0767** | **0** | **(51)** | **1.2e-006** | **1** | **R.LPQLVDMAAQIASGMAYVER.M + Oxidation (M)** |
|  | [2853](http://df3/mascot/cgi/peptide_view.pl?file=../data/20121119/F002569.dat&query=2853&hit=1&index=gi%7c00000001&px=1) | **1090.3279** | **2178.6412** | **2178.0812** | **0.5600** | **0** | **(131)** | **1.3e-014** | **1** | **R.LPQLVDMAAQIASGMAYVER.M + Oxidation (M)** |
|  | [1666](http://df3/mascot/cgi/peptide_view.pl?file=../data/20121119/F002569.dat&query=1666&hit=1&index=gi%7c00000001&px=1) | **727.2584** | **2178.7533** | **2178.0812** | **0.6720** | **0** | **(82)** | **9e-010** | **1** | **R.LPQLVDMAAQIASGMAYVER.M + Oxidation (M)** |
|  | [2854](http://df3/mascot/cgi/peptide_view.pl?file=../data/20121119/F002569.dat&query=2854&hit=1&index=gi%7c00000001&px=1) | **1090.3867** | **2178.7589** | **2178.0812** | **0.6776** | **0** | **(140)** | **1.5e-015** | **1** | **R.LPQLVDMAAQIASGMAYVER.M + Oxidation (M)** |
|  | [1667](http://df3/mascot/cgi/peptide_view.pl?file=../data/20121119/F002569.dat&query=1667&hit=1&index=gi%7c00000001&px=1) | **727.3008** | **2178.8805** | **2178.0812** | **0.7993** | **0** | **(85)** | **4.6e-010** | **1** | **R.LPQLVDMAAQIASGMAYVER.M + Oxidation (M)** |
|  | [2855](http://df3/mascot/cgi/peptide_view.pl?file=../data/20121119/F002569.dat&query=2855&hit=1&index=gi%7c00000001&px=1) | **1090.4825** | **2178.9505** | **2178.0812** | **0.8693** | **0** | **(138)** | **2.2e-015** | **1** | **R.LPQLVDMAAQIASGMAYVER.M + Oxidation (M)** |
|  | [1668](http://df3/mascot/cgi/peptide_view.pl?file=../data/20121119/F002569.dat&query=1668&hit=1&index=gi%7c00000001&px=1) | **727.3544** | **2179.0413** | **2178.0812** | **0.9600** | **0** | **(73)** | **6.8e-009** | **1** | **R.LPQLVDMAAQIASGMAYVER.M + Oxidation (M)** |
|  | [1669](http://df3/mascot/cgi/peptide_view.pl?file=../data/20121119/F002569.dat&query=1669&hit=1&index=gi%7c00000001&px=1) | **727.3605** | **2179.0596** | **2178.0812** | **0.9784** | **0** | **(101)** | **1.3e-011** | **1** | **R.LPQLVDMAAQIASGMAYVER.M + Oxidation (M)** |
|  | [1670](http://df3/mascot/cgi/peptide_view.pl?file=../data/20121119/F002569.dat&query=1670&hit=1&index=gi%7c00000001&px=1) | **727.3721** | **2179.0946** | **2178.0812** | **1.0133** | **0** | **(64)** | **5.4e-008** | **1** | **R.LPQLVDMAAQIASGMAYVER.M + Oxidation (M)** |
|  | [1671](http://df3/mascot/cgi/peptide_view.pl?file=../data/20121119/F002569.dat&query=1671&hit=1&index=gi%7c00000001&px=1) | **727.3861** | **2179.1365** | **2178.0812** | **1.0553** | **0** | **(84)** | **6.6e-010** | **1** | **R.LPQLVDMAAQIASGMAYVER.M + Oxidation (M)** |
|  | [1672](http://df3/mascot/cgi/peptide_view.pl?file=../data/20121119/F002569.dat&query=1672&hit=1&index=gi%7c00000001&px=1) | **727.5321** | **2179.5745** | **2178.0812** | **1.4932** | **0** | **(73)** | **7.4e-009** | **1** | **R.LPQLVDMAAQIASGMAYVER.M + Oxidation (M)** |
|  | [1673](http://df3/mascot/cgi/peptide_view.pl?file=../data/20121119/F002569.dat&query=1673&hit=1&index=gi%7c00000001&px=1) | **727.5482** | **2179.6226** | **2179.0652** | **0.5574** | **0** | **(71)** | **1.2e-008** | **1** | **R.LPQLVDMAAQIASGMAYVER.M + Deamidation (NQ); Oxidation (M)** |
|  | [2856](http://df3/mascot/cgi/peptide_view.pl?file=../data/20121119/F002569.dat&query=2856&hit=1&index=gi%7c00000001&px=1) | **1090.9622** | **2179.9098** | **2178.0812** | **1.8285** | **0** | **(125)** | **4.4e-014** | **1** | **R.LPQLVDMAAQIASGMAYVER.M + Oxidation (M)** |
|  | [2857](http://df3/mascot/cgi/peptide_view.pl?file=../data/20121119/F002569.dat&query=2857&hit=1&index=gi%7c00000001&px=1) | **1090.9961** | **2179.9776** | **2178.0812** | **1.8964** | **0** | **(126)** | **4e-014** | **1** | **R.LPQLVDMAAQIASGMAYVER.M + Oxidation (M)** |
|  | [2858](http://df3/mascot/cgi/peptide_view.pl?file=../data/20121119/F002569.dat&query=2858&hit=1&index=gi%7c00000001&px=1) | **1091.5259** | **2181.0372** | **2179.0652** | **1.9720** | **0** | **(109)** | **1.4e-012** | **1** | **R.LPQLVDMAAQIASGMAYVER.M + Deamidation (NQ); Oxidation (M)** |
|  | [2859](http://df3/mascot/cgi/peptide_view.pl?file=../data/20121119/F002569.dat&query=2859&hit=1&index=gi%7c00000001&px=1) | **1091.6580** | **2181.3014** | **2180.0493** | **1.2521** | **0** | **(102)** | **2.9e-012** | **1** | **R.LPQLVDMAAQIASGMAYVER.M + 2 Deamidation (NQ); Oxidation (M)** |
|  | [1674](http://df3/mascot/cgi/peptide_view.pl?file=../data/20121119/F002569.dat&query=1674&hit=1&index=gi%7c00000001&px=1) | **728.2524** | **2181.7355** | **2180.0493** | **1.6862** | **0** | **(15)** | **0.0018** | **1** | **R.LPQLVDMAAQIASGMAYVER.M + 2 Deamidation (NQ); Oxidation (M)** |
|  | [1677](http://df3/mascot/cgi/peptide_view.pl?file=../data/20121119/F002569.dat&query=1677&hit=1&index=gi%7c00000001&px=1) | **728.3461** | **2182.0166** | **2180.0493** | **1.9673** | **0** | **(15)** | **0.0016** | **1** | **R.LPQLVDMAAQIASGMAYVER.M + 2 Deamidation (NQ); Oxidation (M)** |
|  | [1743](http://df3/mascot/cgi/peptide_view.pl?file=../data/20121119/F002569.dat&query=1743&hit=1&index=gi%7c00000001&px=1) | **731.7423** | **2192.2051** | **2194.0762** | **-1.8711** | **0** | **(40)** | **5e-006** | **1** | **R.LPQLVDMAAQIASGMAYVER.M + 2 Oxidation (M)** |
|  | [1744](http://df3/mascot/cgi/peptide_view.pl?file=../data/20121119/F002569.dat&query=1744&hit=1&index=gi%7c00000001&px=1) | **731.7849** | **2192.3329** | **2194.0762** | **-1.7432** | **0** | **(22)** | **0.00032** | **1** | **R.LPQLVDMAAQIASGMAYVER.M + 2 Oxidation (M)** |
|  | [1745](http://df3/mascot/cgi/peptide_view.pl?file=../data/20121119/F002569.dat&query=1745&hit=1&index=gi%7c00000001&px=1) | **731.9576** | **2192.8509** | **2194.0762** | **-1.2252** | **0** | **(31)** | **4.4e-005** | **1** | **R.LPQLVDMAAQIASGMAYVER.M + 2 Oxidation (M)** |
|  | [1747](http://df3/mascot/cgi/peptide_view.pl?file=../data/20121119/F002569.dat&query=1747&hit=1&index=gi%7c00000001&px=1) | **731.9883** | **2192.9430** | **2194.0762** | **-1.1331** | **0** | **(60)** | **5.2e-008** | **1** | **R.LPQLVDMAAQIASGMAYVER.M + 2 Oxidation (M)** |
|  | [1748](http://df3/mascot/cgi/peptide_view.pl?file=../data/20121119/F002569.dat&query=1748&hit=1&index=gi%7c00000001&px=1) | **732.0997** | **2193.2774** | **2194.0762** | **-0.7988** | **0** | **(45)** | **3.2e-006** | **1** | **R.LPQLVDMAAQIASGMAYVER.M + 2 Oxidation (M)** |
|  | [1749](http://df3/mascot/cgi/peptide_view.pl?file=../data/20121119/F002569.dat&query=1749&hit=1&index=gi%7c00000001&px=1) | **732.1735** | **2193.4986** | **2194.0762** | **-0.5776** | **0** | **(52)** | **5.7e-007** | **1** | **R.LPQLVDMAAQIASGMAYVER.M + 2 Oxidation (M)** |
|  | [1750](http://df3/mascot/cgi/peptide_view.pl?file=../data/20121119/F002569.dat&query=1750&hit=1&index=gi%7c00000001&px=1) | **732.1747** | **2193.5022** | **2194.0762** | **-0.5739** | **0** | **(53)** | **4.5e-007** | **1** | **R.LPQLVDMAAQIASGMAYVER.M + 2 Oxidation (M)** |
|  | [1751](http://df3/mascot/cgi/peptide_view.pl?file=../data/20121119/F002569.dat&query=1751&hit=1&index=gi%7c00000001&px=1) | **732.1760** | **2193.5062** | **2194.0762** | **-0.5699** | **0** | **(55)** | **3.5e-007** | **1** | **R.LPQLVDMAAQIASGMAYVER.M + 2 Oxidation (M)** |
|  | [1752](http://df3/mascot/cgi/peptide_view.pl?file=../data/20121119/F002569.dat&query=1752&hit=1&index=gi%7c00000001&px=1) | **732.1868** | **2193.5385** | **2194.0762** | **-0.5377** | **0** | **(49)** | **1.3e-006** | **1** | **R.LPQLVDMAAQIASGMAYVER.M + 2 Oxidation (M)** |
|  | [1753](http://df3/mascot/cgi/peptide_view.pl?file=../data/20121119/F002569.dat&query=1753&hit=1&index=gi%7c00000001&px=1) | **732.2188** | **2193.6344** | **2194.0762** | **-0.4417** | **0** | **(35)** | **3.3e-005** | **1** | **R.LPQLVDMAAQIASGMAYVER.M + 2 Oxidation (M)** |
|  | [2867](http://df3/mascot/cgi/peptide_view.pl?file=../data/20121119/F002569.dat&query=2867&hit=1&index=gi%7c00000001&px=1) | **1097.8734** | **2193.7323** | **2194.0762** | **-0.3439** | **0** | **(136)** | **2.7e-015** | **1** | **R.LPQLVDMAAQIASGMAYVER.M + 2 Oxidation (M)** |
|  | [1754](http://df3/mascot/cgi/peptide_view.pl?file=../data/20121119/F002569.dat&query=1754&hit=1&index=gi%7c00000001&px=1) | **732.2632** | **2193.7677** | **2194.0762** | **-0.3084** | **0** | **(64)** | **3.9e-008** | **1** | **R.LPQLVDMAAQIASGMAYVER.M + 2 Oxidation (M)** |
|  | [2868](http://df3/mascot/cgi/peptide_view.pl?file=../data/20121119/F002569.dat&query=2868&hit=1&index=gi%7c00000001&px=1) | **1097.9006** | **2193.7867** | **2194.0762** | **-0.2894** | **0** | **(147)** | **1.9e-016** | **1** | **R.LPQLVDMAAQIASGMAYVER.M + 2 Oxidation (M)** |
|  | [1755](http://df3/mascot/cgi/peptide_view.pl?file=../data/20121119/F002569.dat&query=1755&hit=1&index=gi%7c00000001&px=1) | **732.2734** | **2193.7985** | **2194.0762** | **-0.2777** | **0** | **(46)** | **2.3e-006** | **1** | **R.LPQLVDMAAQIASGMAYVER.M + 2 Oxidation (M)** |
|  | [1756](http://df3/mascot/cgi/peptide_view.pl?file=../data/20121119/F002569.dat&query=1756&hit=1&index=gi%7c00000001&px=1) | **732.3440** | **2194.0102** | **2194.0762** | **-0.0660** | **0** | **(54)** | **3.7e-007** | **1** | **R.LPQLVDMAAQIASGMAYVER.M + 2 Oxidation (M)** |
|  | [1757](http://df3/mascot/cgi/peptide_view.pl?file=../data/20121119/F002569.dat&query=1757&hit=1&index=gi%7c00000001&px=1) | **732.3566** | **2194.0479** | **2194.0762** | **-0.0283** | **0** | **(55)** | **4.6e-007** | **1** | **R.LPQLVDMAAQIASGMAYVER.M + 2 Oxidation (M)** |
|  | [2869](http://df3/mascot/cgi/peptide_view.pl?file=../data/20121119/F002569.dat&query=2869&hit=1&index=gi%7c00000001&px=1) | **1098.0540** | **2194.0934** | **2194.0762** | **0.0172** | **0** | **(145)** | **4.9e-016** | **1** | **R.LPQLVDMAAQIASGMAYVER.M + 2 Oxidation (M)** |
|  | [1758](http://df3/mascot/cgi/peptide_view.pl?file=../data/20121119/F002569.dat&query=1758&hit=1&index=gi%7c00000001&px=1) | **732.4276** | **2194.2608** | **2194.0762** | **0.1847** | **0** | **(61)** | **1.2e-007** | **1** | **R.LPQLVDMAAQIASGMAYVER.M + 2 Oxidation (M)** |
|  | [2870](http://df3/mascot/cgi/peptide_view.pl?file=../data/20121119/F002569.dat&query=2870&hit=1&index=gi%7c00000001&px=1) | **1098.1718** | **2194.3290** | **2194.0762** | **0.2528** | **0** | **(145)** | **5.3e-016** | **1** | **R.LPQLVDMAAQIASGMAYVER.M + 2 Oxidation (M)** |
|  | [1759](http://df3/mascot/cgi/peptide_view.pl?file=../data/20121119/F002569.dat&query=1759&hit=1&index=gi%7c00000001&px=1) | **732.4551** | **2194.3436** | **2194.0762** | **0.2674** | **0** | **(56)** | **3.8e-007** | **1** | **R.LPQLVDMAAQIASGMAYVER.M + 2 Oxidation (M)** |
|  | [1760](http://df3/mascot/cgi/peptide_view.pl?file=../data/20121119/F002569.dat&query=1760&hit=1&index=gi%7c00000001&px=1) | **732.4836** | **2194.4291** | **2194.0762** | **0.3529** | **0** | **(66)** | **4e-008** | **1** | **R.LPQLVDMAAQIASGMAYVER.M + 2 Oxidation (M)** |
|  | [1761](http://df3/mascot/cgi/peptide_view.pl?file=../data/20121119/F002569.dat&query=1761&hit=1&index=gi%7c00000001&px=1) | **732.4861** | **2194.4364** | **2194.0762** | **0.3603** | **0** | **(68)** | **2.4e-008** | **1** | **R.LPQLVDMAAQIASGMAYVER.M + 2 Oxidation (M)** |
|  | [1762](http://df3/mascot/cgi/peptide_view.pl?file=../data/20121119/F002569.dat&query=1762&hit=1&index=gi%7c00000001&px=1) | **732.5079** | **2194.5020** | **2194.0762** | **0.4258** | **0** | **(75)** | **5.2e-009** | **1** | **R.LPQLVDMAAQIASGMAYVER.M + 2 Oxidation (M)** |
|  | [1763](http://df3/mascot/cgi/peptide_view.pl?file=../data/20121119/F002569.dat&query=1763&hit=1&index=gi%7c00000001&px=1) | **732.5167** | **2194.5283** | **2194.0762** | **0.4522** | **0** | **(77)** | **3.2e-009** | **1** | **R.LPQLVDMAAQIASGMAYVER.M + 2 Oxidation (M)** |
|  | [1764](http://df3/mascot/cgi/peptide_view.pl?file=../data/20121119/F002569.dat&query=1764&hit=1&index=gi%7c00000001&px=1) | **732.5179** | **2194.5320** | **2194.0762** | **0.4558** | **0** | **(78)** | **2.3e-009** | **1** | **R.LPQLVDMAAQIASGMAYVER.M + 2 Oxidation (M)** |
|  | [2871](http://df3/mascot/cgi/peptide_view.pl?file=../data/20121119/F002569.dat&query=2871&hit=1&index=gi%7c00000001&px=1) | **1098.3643** | **2194.7140** | **2194.0762** | **0.6378** | **0** | **(126)** | **4.1e-014** | **1** | **R.LPQLVDMAAQIASGMAYVER.M + 2 Oxidation (M)** |
|  | [1766](http://df3/mascot/cgi/peptide_view.pl?file=../data/20121119/F002569.dat&query=1766&hit=1&index=gi%7c00000001&px=1) | **732.5851** | **2194.7334** | **2194.0762** | **0.6573** | **0** | **(64)** | **6.4e-008** | **1** | **R.LPQLVDMAAQIASGMAYVER.M + 2 Oxidation (M)** |
|  | [2872](http://df3/mascot/cgi/peptide_view.pl?file=../data/20121119/F002569.dat&query=2872&hit=1&index=gi%7c00000001&px=1) | **1098.3833** | **2194.7521** | **2194.0762** | **0.6759** | **0** | **(136)** | **4.1e-015** | **1** | **R.LPQLVDMAAQIASGMAYVER.M + 2 Oxidation (M)** |
|  | [1767](http://df3/mascot/cgi/peptide_view.pl?file=../data/20121119/F002569.dat&query=1767&hit=1&index=gi%7c00000001&px=1) | **732.5942** | **2194.7607** | **2194.0762** | **0.6845** | **0** | **(58)** | **2.3e-007** | **1** | **R.LPQLVDMAAQIASGMAYVER.M + 2 Oxidation (M)** |
|  | [1768](http://df3/mascot/cgi/peptide_view.pl?file=../data/20121119/F002569.dat&query=1768&hit=1&index=gi%7c00000001&px=1) | **732.6206** | **2194.8400** | **2194.0762** | **0.7638** | **0** | **(66)** | **3.4e-008** | **1** | **R.LPQLVDMAAQIASGMAYVER.M + 2 Oxidation (M)** |
|  | [1769](http://df3/mascot/cgi/peptide_view.pl?file=../data/20121119/F002569.dat&query=1769&hit=1&index=gi%7c00000001&px=1) | **732.6644** | **2194.9715** | **2194.0762** | **0.8953** | **0** | **(56)** | **3.4e-007** | **1** | **R.LPQLVDMAAQIASGMAYVER.M + 2 Oxidation (M)** |
|  | [1771](http://df3/mascot/cgi/peptide_view.pl?file=../data/20121119/F002569.dat&query=1771&hit=1&index=gi%7c00000001&px=1) | **732.7224** | **2195.1454** | **2194.0762** | **1.0693** | **0** | **(66)** | **3.4e-008** | **1** | **R.LPQLVDMAAQIASGMAYVER.M + 2 Oxidation (M)** |
|  | [1772](http://df3/mascot/cgi/peptide_view.pl?file=../data/20121119/F002569.dat&query=1772&hit=1&index=gi%7c00000001&px=1) | **732.7256** | **2195.1551** | **2194.0762** | **1.0790** | **0** | **(70)** | **1.7e-008** | **1** | **R.LPQLVDMAAQIASGMAYVER.M + 2 Oxidation (M)** |
|  | [1773](http://df3/mascot/cgi/peptide_view.pl?file=../data/20121119/F002569.dat&query=1773&hit=1&index=gi%7c00000001&px=1) | **732.7570** | **2195.2490** | **2195.0602** | **0.1889** | **0** | **(71)** | **1.1e-008** | **1** | **R.LPQLVDMAAQIASGMAYVER.M + Deamidation (NQ); 2 Oxidation (M)** |
|  | [1774](http://df3/mascot/cgi/peptide_view.pl?file=../data/20121119/F002569.dat&query=1774&hit=1&index=gi%7c00000001&px=1) | **732.7754** | **2195.3043** | **2194.0762** | **1.2282** | **0** | **(85)** | **4.7e-010** | **1** | **R.LPQLVDMAAQIASGMAYVER.M + 2 Oxidation (M)** |
|  | [1775](http://df3/mascot/cgi/peptide_view.pl?file=../data/20121119/F002569.dat&query=1775&hit=1&index=gi%7c00000001&px=1) | **732.7755** | **2195.3045** | **2195.0602** | **0.2444** | **0** | **(62)** | **9.4e-008** | **1** | **R.LPQLVDMAAQIASGMAYVER.M + Deamidation (NQ); 2 Oxidation (M)** |
|  | [1776](http://df3/mascot/cgi/peptide_view.pl?file=../data/20121119/F002569.dat&query=1776&hit=1&index=gi%7c00000001&px=1) | **732.7867** | **2195.3384** | **2194.0762** | **1.2622** | **0** | **(77)** | **3.2e-009** | **1** | **R.LPQLVDMAAQIASGMAYVER.M + 2 Oxidation (M)** |
|  | [1777](http://df3/mascot/cgi/peptide_view.pl?file=../data/20121119/F002569.dat&query=1777&hit=1&index=gi%7c00000001&px=1) | **732.7892** | **2195.3457** | **2194.0762** | **1.2696** | **0** | **(65)** | **5.1e-008** | **1** | **R.LPQLVDMAAQIASGMAYVER.M + 2 Oxidation (M)** |
|  | [1778](http://df3/mascot/cgi/peptide_view.pl?file=../data/20121119/F002569.dat&query=1778&hit=1&index=gi%7c00000001&px=1) | **732.7954** | **2195.3644** | **2195.0602** | **0.3042** | **0** | **(77)** | **3.4e-009** | **1** | **R.LPQLVDMAAQIASGMAYVER.M + Deamidation (NQ); 2 Oxidation (M)** |
|  | [1779](http://df3/mascot/cgi/peptide_view.pl?file=../data/20121119/F002569.dat&query=1779&hit=1&index=gi%7c00000001&px=1) | **732.8126** | **2195.4160** | **2194.0762** | **1.3399** | **0** | **(74)** | **6.1e-009** | **1** | **R.LPQLVDMAAQIASGMAYVER.M + 2 Oxidation (M)** |
|  | [1780](http://df3/mascot/cgi/peptide_view.pl?file=../data/20121119/F002569.dat&query=1780&hit=1&index=gi%7c00000001&px=1) | **732.8160** | **2195.4263** | **2194.0762** | **1.3501** | **0** | **(88)** | **2.1e-010** | **1** | **R.LPQLVDMAAQIASGMAYVER.M + 2 Oxidation (M)** |
|  | [1781](http://df3/mascot/cgi/peptide_view.pl?file=../data/20121119/F002569.dat&query=1781&hit=1&index=gi%7c00000001&px=1) | **732.8290** | **2195.4651** | **2194.0762** | **1.3890** | **0** | **(69)** | **1.9e-008** | **1** | **R.LPQLVDMAAQIASGMAYVER.M + 2 Oxidation (M)** |
|  | [1782](http://df3/mascot/cgi/peptide_view.pl?file=../data/20121119/F002569.dat&query=1782&hit=1&index=gi%7c00000001&px=1) | **732.8740** | **2195.6002** | **2194.0762** | **1.5241** | **0** | **(62)** | **9.8e-008** | **1** | **R.LPQLVDMAAQIASGMAYVER.M + 2 Oxidation (M)** |
|  | [1783](http://df3/mascot/cgi/peptide_view.pl?file=../data/20121119/F002569.dat&query=1783&hit=1&index=gi%7c00000001&px=1) | **732.8960** | **2195.6662** | **2194.0762** | **1.5900** | **0** | **(61)** | **1.3e-007** | **1** | **R.LPQLVDMAAQIASGMAYVER.M + 2 Oxidation (M)** |
|  | [1784](http://df3/mascot/cgi/peptide_view.pl?file=../data/20121119/F002569.dat&query=1784&hit=1&index=gi%7c00000001&px=1) | **732.9095** | **2195.7066** | **2194.0762** | **1.6305** | **0** | **(56)** | **3.9e-007** | **1** | **R.LPQLVDMAAQIASGMAYVER.M + 2 Oxidation (M)** |
|  | [1785](http://df3/mascot/cgi/peptide_view.pl?file=../data/20121119/F002569.dat&query=1785&hit=1&index=gi%7c00000001&px=1) | **732.9170** | **2195.7291** | **2194.0762** | **1.6530** | **0** | **(40)** | **1.4e-005** | **1** | **R.LPQLVDMAAQIASGMAYVER.M + 2 Oxidation (M)** |
|  | [1786](http://df3/mascot/cgi/peptide_view.pl?file=../data/20121119/F002569.dat&query=1786&hit=1&index=gi%7c00000001&px=1) | **732.9463** | **2195.8170** | **2194.0762** | **1.7409** | **0** | **(69)** | **1.9e-008** | **1** | **R.LPQLVDMAAQIASGMAYVER.M + 2 Oxidation (M)** |
|  | [2873](http://df3/mascot/cgi/peptide_view.pl?file=../data/20121119/F002569.dat&query=2873&hit=1&index=gi%7c00000001&px=1) | **1098.9633** | **2195.9120** | **2194.0762** | **1.8358** | **0** | **(125)** | **5.1e-014** | **1** | **R.LPQLVDMAAQIASGMAYVER.M + 2 Oxidation (M)** |
|  | [1787](http://df3/mascot/cgi/peptide_view.pl?file=../data/20121119/F002569.dat&query=1787&hit=1&index=gi%7c00000001&px=1) | **732.9888** | **2195.9445** | **2194.0762** | **1.8683** | **0** | **(65)** | **4.6e-008** | **1** | **R.LPQLVDMAAQIASGMAYVER.M + 2 Oxidation (M)** |
|  | [1788](http://df3/mascot/cgi/peptide_view.pl?file=../data/20121119/F002569.dat&query=1788&hit=1&index=gi%7c00000001&px=1) | **733.0156** | **2196.0250** | **2194.0762** | **1.9489** | **0** | **(76)** | **3.8e-009** | **1** | **R.LPQLVDMAAQIASGMAYVER.M + 2 Oxidation (M)** |
|  | [1789](http://df3/mascot/cgi/peptide_view.pl?file=../data/20121119/F002569.dat&query=1789&hit=1&index=gi%7c00000001&px=1) | **733.0380** | **2196.0921** | **2195.0602** | **1.0319** | **0** | **(69)** | **1.3e-008** | **1** | **R.LPQLVDMAAQIASGMAYVER.M + Deamidation (NQ); 2 Oxidation (M)** |
|  | [1790](http://df3/mascot/cgi/peptide_view.pl?file=../data/20121119/F002569.dat&query=1790&hit=1&index=gi%7c00000001&px=1) | **733.0436** | **2196.1089** | **2195.0602** | **1.0487** | **0** | **(42)** | **6.9e-006** | **1** | **R.LPQLVDMAAQIASGMAYVER.M + Deamidation (NQ); 2 Oxidation (M)** |
|  | [1792](http://df3/mascot/cgi/peptide_view.pl?file=../data/20121119/F002569.dat&query=1792&hit=1&index=gi%7c00000001&px=1) | **733.1802** | **2196.5189** | **2195.0602** | **1.4587** | **0** | **(59)** | **1.3e-007** | **1** | **R.LPQLVDMAAQIASGMAYVER.M + Deamidation (NQ); 2 Oxidation (M)** |
|  | [1793](http://df3/mascot/cgi/peptide_view.pl?file=../data/20121119/F002569.dat&query=1793&hit=1&index=gi%7c00000001&px=1) | **733.1807** | **2196.5202** | **2195.0602** | **1.4600** | **0** | **(65)** | **3.1e-008** | **1** | **R.LPQLVDMAAQIASGMAYVER.M + Deamidation (NQ); 2 Oxidation (M)** |
|  | [2875](http://df3/mascot/cgi/peptide_view.pl?file=../data/20121119/F002569.dat&query=2875&hit=1&index=gi%7c00000001&px=1) | **1099.4785** | **2196.9425** | **2195.0602** | **1.8823** | **0** | **(116)** | **2.5e-013** | **1** | **R.LPQLVDMAAQIASGMAYVER.M + Deamidation (NQ); 2 Oxidation (M)** |
|  | [1794](http://df3/mascot/cgi/peptide_view.pl?file=../data/20121119/F002569.dat&query=1794&hit=1&index=gi%7c00000001&px=1) | **733.3821** | **2197.1244** | **2196.0442** | **1.0802** | **0** | **(48)** | **8.8e-007** | **1** | **R.LPQLVDMAAQIASGMAYVER.M + 2 Deamidation (NQ); 2 Oxidation (M)** |
|  | [2876](http://df3/mascot/cgi/peptide_view.pl?file=../data/20121119/F002569.dat&query=2876&hit=1&index=gi%7c00000001&px=1) | **1099.6676** | **2197.3207** | **2196.0442** | **1.2765** | **0** | **(88)** | **7.8e-011** | **1** | **R.LPQLVDMAAQIASGMAYVER.M + 2 Deamidation (NQ); 2 Oxidation (M)** |
|  | [2878](http://df3/mascot/cgi/peptide_view.pl?file=../data/20121119/F002569.dat&query=2878&hit=1&index=gi%7c00000001&px=1) | **1099.8866** | **2197.7586** | **2196.0442** | **1.7145** | **0** | **(106)** | **1.4e-012** | **1** | **R.LPQLVDMAAQIASGMAYVER.M + 2 Deamidation (NQ); 2 Oxidation (M)** |
|  | [1805](http://df3/mascot/cgi/peptide_view.pl?file=../data/20121119/F002569.dat&query=1805&hit=1&index=gi%7c00000001&px=1) | **735.6364** | **2203.8874** | **2205.1173** | **-1.2299** | **0** | **(14)** | **0.0019** | **1** | **K.TLKPGTMSPEAFLQEAQVMK.K** |
|  | [1811](http://df3/mascot/cgi/peptide_view.pl?file=../data/20121119/F002569.dat&query=1811&hit=1&index=gi%7c00000001&px=1) | **736.3164** | **2205.9274** | **2205.1173** | **0.8101** | **0** | **(41)** | **1.3e-005** | **1** | **K.TLKPGTMSPEAFLQEAQVMK.K** |
|  | [1812](http://df3/mascot/cgi/peptide_view.pl?file=../data/20121119/F002569.dat&query=1812&hit=1&index=gi%7c00000001&px=1) | **736.6193** | **2206.8360** | **2205.1173** | **1.7187** | **0** | **(27)** | **0.0003** | **1** | **K.TLKPGTMSPEAFLQEAQVMK.K** |
|  | [1869](http://df3/mascot/cgi/peptide_view.pl?file=../data/20121119/F002569.dat&query=1869&hit=1&index=gi%7c00000001&px=1) | **740.8018** | **2219.3836** | **2221.1122** | **-1.7286** | **0** | **(56)** | **3.4e-007** | **1** | **K.TLKPGTMSPEAFLQEAQVMK.K + Oxidation (M)** |
|  | [1870](http://df3/mascot/cgi/peptide_view.pl?file=../data/20121119/F002569.dat&query=1870&hit=1&index=gi%7c00000001&px=1) | **741.0604** | **2220.1593** | **2220.9844** | **-0.8252** | **1** | **(15)** | **0.0058** | **1** | **R.ESETTKGAYCLSVSDFDNAK.G + Carbamidomethyl (C)** |
|  | [1873](http://df3/mascot/cgi/peptide_view.pl?file=../data/20121119/F002569.dat&query=1873&hit=1&index=gi%7c00000001&px=1) | **741.2125** | **2220.6157** | **2221.1122** | **-0.4965** | **0** | **(14)** | **0.0088** | **1** | **K.TLKPGTMSPEAFLQEAQVMK.K + Oxidation (M)** |
|  | [1875](http://df3/mascot/cgi/peptide_view.pl?file=../data/20121119/F002569.dat&query=1875&hit=1&index=gi%7c00000001&px=1) | **741.3467** | **2221.0184** | **2221.1122** | **-0.0938** | **0** | **(22)** | **0.0012** | **1** | **K.TLKPGTMSPEAFLQEAQVMK.K + Oxidation (M)** |
|  | [1877](http://df3/mascot/cgi/peptide_view.pl?file=../data/20121119/F002569.dat&query=1877&hit=1&index=gi%7c00000001&px=1) | **741.3905** | **2221.1497** | **2220.9844** | **0.1652** | **1** | **(61)** | **1.8e-007** | **1** | **R.ESETTKGAYCLSVSDFDNAK.G + Carbamidomethyl (C)** |
|  | [1879](http://df3/mascot/cgi/peptide_view.pl?file=../data/20121119/F002569.dat&query=1879&hit=1&index=gi%7c00000001&px=1) | **741.4304** | **2221.2694** | **2220.9844** | **0.2850** | **1** | **(12)** | **0.016** | **1** | **R.ESETTKGAYCLSVSDFDNAK.G + Carbamidomethyl (C)** |
|  | [1881](http://df3/mascot/cgi/peptide_view.pl?file=../data/20121119/F002569.dat&query=1881&hit=1&index=gi%7c00000001&px=1) | **741.4424** | **2221.3053** | **2220.9844** | **0.3209** | **1** | **(13)** | **0.012** | **1** | **R.ESETTKGAYCLSVSDFDNAK.G + Carbamidomethyl (C)** |
|  | [1885](http://df3/mascot/cgi/peptide_view.pl?file=../data/20121119/F002569.dat&query=1885&hit=1&index=gi%7c00000001&px=1) | **741.5100** | **2221.5082** | **2220.9844** | **0.5238** | **1** | **(43)** | **1.2e-005** | **1** | **R.ESETTKGAYCLSVSDFDNAK.G + Carbamidomethyl (C)** |
|  | [1887](http://df3/mascot/cgi/peptide_view.pl?file=../data/20121119/F002569.dat&query=1887&hit=1&index=gi%7c00000001&px=1) | **741.6075** | **2221.8008** | **2220.9844** | **0.8164** | **1** | **(12)** | **0.017** | **1** | **R.ESETTKGAYCLSVSDFDNAK.G + Carbamidomethyl (C)** |
|  | [2891](http://df3/mascot/cgi/peptide_view.pl?file=../data/20121119/F002569.dat&query=2891&hit=1&index=gi%7c00000001&px=1) | **1111.9387** | **2221.8629** | **2221.1122** | **0.7507** | **0** | **(59)** | **3.3e-007** | **1** | **K.TLKPGTMSPEAFLQEAQVMK.K + Oxidation (M)** |
|  | [1889](http://df3/mascot/cgi/peptide_view.pl?file=../data/20121119/F002569.dat&query=1889&hit=1&index=gi%7c00000001&px=1) | **741.7228** | **2222.1467** | **2220.9844** | **1.1623** | **1** | **(60)** | **2.8e-007** | **1** | **R.ESETTKGAYCLSVSDFDNAK.G + Carbamidomethyl (C)** |
|  | [1890](http://df3/mascot/cgi/peptide_view.pl?file=../data/20121119/F002569.dat&query=1890&hit=1&index=gi%7c00000001&px=1) | **741.7636** | **2222.2688** | **2220.9844** | **1.2844** | **1** | **(34)** | **0.00011** | **1** | **R.ESETTKGAYCLSVSDFDNAK.G + Carbamidomethyl (C)** |
|  | [1891](http://df3/mascot/cgi/peptide_view.pl?file=../data/20121119/F002569.dat&query=1891&hit=1&index=gi%7c00000001&px=1) | **741.7750** | **2222.3031** | **2222.0962** | **0.2068** | **0** | **(12)** | **0.016** | **1** | **K.TLKPGTMSPEAFLQEAQVMK.K + Deamidation (NQ); Oxidation (M)** |
|  | [1892](http://df3/mascot/cgi/peptide_view.pl?file=../data/20121119/F002569.dat&query=1892&hit=1&index=gi%7c00000001&px=1) | **741.8004** | **2222.3794** | **2220.9844** | **1.3950** | **1** | **65** | **8.4e-008** | **1** | **R.ESETTKGAYCLSVSDFDNAK.G + Carbamidomethyl (C)** |
|  | [1895](http://df3/mascot/cgi/peptide_view.pl?file=../data/20121119/F002569.dat&query=1895&hit=1&index=gi%7c00000001&px=1) | **741.8648** | **2222.5726** | **2222.0962** | **0.4764** | **0** | **(56)** | **6.4e-007** | **1** | **K.TLKPGTMSPEAFLQEAQVMK.K + Deamidation (NQ); Oxidation (M)** |
|  | [1896](http://df3/mascot/cgi/peptide_view.pl?file=../data/20121119/F002569.dat&query=1896&hit=1&index=gi%7c00000001&px=1) | **741.8710** | **2222.5911** | **2221.1122** | **1.4789** | **0** | **(35)** | **7.7e-005** | **1** | **K.TLKPGTMSPEAFLQEAQVMK.K + Oxidation (M)** |
|  | [1897](http://df3/mascot/cgi/peptide_view.pl?file=../data/20121119/F002569.dat&query=1897&hit=1&index=gi%7c00000001&px=1) | **742.0092** | **2223.0056** | **2221.1122** | **1.8934** | **0** | **(57)** | **4.3e-007** | **1** | **K.TLKPGTMSPEAFLQEAQVMK.K + Oxidation (M)** |
|  | [1898](http://df3/mascot/cgi/peptide_view.pl?file=../data/20121119/F002569.dat&query=1898&hit=1&index=gi%7c00000001&px=1) | **742.0841** | **2223.2305** | **2222.0962** | **1.1343** | **0** | **(15)** | **0.0044** | **1** | **K.TLKPGTMSPEAFLQEAQVMK.K + Deamidation (NQ); Oxidation (M)** |
|  | [1899](http://df3/mascot/cgi/peptide_view.pl?file=../data/20121119/F002569.dat&query=1899&hit=1&index=gi%7c00000001&px=1) | **742.1614** | **2223.4623** | **2222.0962** | **1.3661** | **0** | **(29)** | **0.00023** | **1** | **K.TLKPGTMSPEAFLQEAQVMK.K + Deamidation (NQ); Oxidation (M)** |
|  | [1901](http://df3/mascot/cgi/peptide_view.pl?file=../data/20121119/F002569.dat&query=1901&hit=1&index=gi%7c00000001&px=1) | **742.2937** | **2223.8593** | **2222.0962** | **1.7631** | **0** | **(42)** | **1.4e-005** | **1** | **K.TLKPGTMSPEAFLQEAQVMK.K + Deamidation (NQ); Oxidation (M)** |
|  | [1915](http://df3/mascot/cgi/peptide_view.pl?file=../data/20121119/F002569.dat&query=1915&hit=1&index=gi%7c00000001&px=1) | **744.0626** | **2229.1659** | **2229.1939** | **-0.0280** | **1** | **(44)** | **7.2e-006** | **1** | **R.AANILVGENLVCKVADFGLAR.L + Carbamidomethyl (C)** |
|  | [1919](http://df3/mascot/cgi/peptide_view.pl?file=../data/20121119/F002569.dat&query=1919&hit=1&index=gi%7c00000001&px=1) | **744.5799** | **2230.7179** | **2229.1939** | **1.5240** | **1** | **54** | **7.7e-007** | **1** | **R.AANILVGENLVCKVADFGLAR.L + Carbamidomethyl (C)** |
|  | [1927](http://df3/mascot/cgi/peptide_view.pl?file=../data/20121119/F002569.dat&query=1927&hit=1&index=gi%7c00000001&px=1) | **746.0497** | **2235.1272** | **2237.1071** | **-1.9799** | **0** | **61** | **1.3e-007** | **1** | **K.TLKPGTMSPEAFLQEAQVMK.K + 2 Oxidation (M)** |
|  | [1930](http://df3/mascot/cgi/peptide_view.pl?file=../data/20121119/F002569.dat&query=1930&hit=1&index=gi%7c00000001&px=1) | **746.1116** | **2235.3129** | **2237.1071** | **-1.7942** | **0** | **(14)** | **0.0054** | **1** | **K.TLKPGTMSPEAFLQEAQVMK.K + 2 Oxidation (M)** |
|  | [1934](http://df3/mascot/cgi/peptide_view.pl?file=../data/20121119/F002569.dat&query=1934&hit=1&index=gi%7c00000001&px=1) | **746.2763** | **2235.8071** | **2237.1071** | **-1.3000** | **0** | **(56)** | **3.4e-007** | **1** | **K.TLKPGTMSPEAFLQEAQVMK.K + 2 Oxidation (M)** |
|  | [1941](http://df3/mascot/cgi/peptide_view.pl?file=../data/20121119/F002569.dat&query=1941&hit=1&index=gi%7c00000001&px=1) | **746.3713** | **2236.0922** | **2237.1071** | **-1.0149** | **0** | **(18)** | **0.0024** | **1** | **K.TLKPGTMSPEAFLQEAQVMK.K + 2 Oxidation (M)** |
|  | [2896](http://df3/mascot/cgi/peptide_view.pl?file=../data/20121119/F002569.dat&query=2896&hit=1&index=gi%7c00000001&px=1) | **1119.4061** | **2236.7977** | **2237.1071** | **-0.3094** | **0** | **(55)** | **2.9e-007** | **1** | **K.TLKPGTMSPEAFLQEAQVMK.K + 2 Oxidation (M)** |
|  | [1943](http://df3/mascot/cgi/peptide_view.pl?file=../data/20121119/F002569.dat&query=1943&hit=1&index=gi%7c00000001&px=1) | **746.8713** | **2237.5922** | **2237.1071** | **0.4851** | **0** | **(21)** | **0.0013** | **1** | **K.TLKPGTMSPEAFLQEAQVMK.K + 2 Oxidation (M)** |
|  | [2897](http://df3/mascot/cgi/peptide_view.pl?file=../data/20121119/F002569.dat&query=2897&hit=1&index=gi%7c00000001&px=1) | **1119.8865** | **2237.7584** | **2237.1071** | **0.6513** | **0** | **(59)** | **1.7e-007** | **1** | **K.TLKPGTMSPEAFLQEAQVMK.K + 2 Oxidation (M)** |
|  | [1944](http://df3/mascot/cgi/peptide_view.pl?file=../data/20121119/F002569.dat&query=1944&hit=1&index=gi%7c00000001&px=1) | **746.9279** | **2237.7617** | **2238.0911** | **-0.3294** | **0** | **(18)** | **0.0025** | **1** | **K.TLKPGTMSPEAFLQEAQVMK.K + Deamidation (NQ); 2 Oxidation (M)** |
|  | [1945](http://df3/mascot/cgi/peptide_view.pl?file=../data/20121119/F002569.dat&query=1945&hit=1&index=gi%7c00000001&px=1) | **747.0457** | **2238.1151** | **2238.0911** | **0.0240** | **0** | **(15)** | **0.0053** | **1** | **K.TLKPGTMSPEAFLQEAQVMK.K + Deamidation (NQ); 2 Oxidation (M)** |
|  | [1946](http://df3/mascot/cgi/peptide_view.pl?file=../data/20121119/F002569.dat&query=1946&hit=1&index=gi%7c00000001&px=1) | **747.0510** | **2238.1312** | **2237.1071** | **1.0241** | **0** | **(30)** | **0.00015** | **1** | **K.TLKPGTMSPEAFLQEAQVMK.K + 2 Oxidation (M)** |
|  | [1947](http://df3/mascot/cgi/peptide_view.pl?file=../data/20121119/F002569.dat&query=1947&hit=1&index=gi%7c00000001&px=1) | **747.0745** | **2238.2016** | **2237.1071** | **1.0944** | **0** | **(22)** | **0.00087** | **1** | **K.TLKPGTMSPEAFLQEAQVMK.K + 2 Oxidation (M)** |
|  | [1948](http://df3/mascot/cgi/peptide_view.pl?file=../data/20121119/F002569.dat&query=1948&hit=1&index=gi%7c00000001&px=1) | **747.1702** | **2238.4887** | **2238.0911** | **0.3975** | **0** | **(56)** | **4e-007** | **1** | **K.TLKPGTMSPEAFLQEAQVMK.K + Deamidation (NQ); 2 Oxidation (M)** |
|  | [1949](http://df3/mascot/cgi/peptide_view.pl?file=../data/20121119/F002569.dat&query=1949&hit=1&index=gi%7c00000001&px=1) | **747.2107** | **2238.6103** | **2238.0911** | **0.5191** | **0** | **(51)** | **1.1e-006** | **1** | **K.TLKPGTMSPEAFLQEAQVMK.K + Deamidation (NQ); 2 Oxidation (M)** |
|  | [1950](http://df3/mascot/cgi/peptide_view.pl?file=../data/20121119/F002569.dat&query=1950&hit=1&index=gi%7c00000001&px=1) | **747.2480** | **2238.7221** | **2238.0911** | **0.6310** | **0** | **(46)** | **3.7e-006** | **1** | **K.TLKPGTMSPEAFLQEAQVMK.K + Deamidation (NQ); 2 Oxidation (M)** |
|  | [1951](http://df3/mascot/cgi/peptide_view.pl?file=../data/20121119/F002569.dat&query=1951&hit=1&index=gi%7c00000001&px=1) | **747.2546** | **2238.7421** | **2237.1071** | **1.6350** | **0** | **(35)** | **4.9e-005** | **1** | **K.TLKPGTMSPEAFLQEAQVMK.K + 2 Oxidation (M)** |
|  | [1952](http://df3/mascot/cgi/peptide_view.pl?file=../data/20121119/F002569.dat&query=1952&hit=1&index=gi%7c00000001&px=1) | **747.2777** | **2238.8113** | **2238.0911** | **0.7202** | **0** | **(58)** | **2.2e-007** | **1** | **K.TLKPGTMSPEAFLQEAQVMK.K + Deamidation (NQ); 2 Oxidation (M)** |
|  | [1953](http://df3/mascot/cgi/peptide_view.pl?file=../data/20121119/F002569.dat&query=1953&hit=1&index=gi%7c00000001&px=1) | **747.3028** | **2238.8866** | **2238.0911** | **0.7954** | **0** | **(53)** | **7.6e-007** | **1** | **K.TLKPGTMSPEAFLQEAQVMK.K + Deamidation (NQ); 2 Oxidation (M)** |
|  | [1954](http://df3/mascot/cgi/peptide_view.pl?file=../data/20121119/F002569.dat&query=1954&hit=1&index=gi%7c00000001&px=1) | **747.3149** | **2238.9230** | **2237.1071** | **1.8159** | **0** | **(37)** | **3.1e-005** | **1** | **K.TLKPGTMSPEAFLQEAQVMK.K + 2 Oxidation (M)** |
|  | [1955](http://df3/mascot/cgi/peptide_view.pl?file=../data/20121119/F002569.dat&query=1955&hit=1&index=gi%7c00000001&px=1) | **747.3609** | **2239.0609** | **2238.0911** | **0.9697** | **0** | **(46)** | **3.6e-006** | **1** | **K.TLKPGTMSPEAFLQEAQVMK.K + Deamidation (NQ); 2 Oxidation (M)** |
|  | [1956](http://df3/mascot/cgi/peptide_view.pl?file=../data/20121119/F002569.dat&query=1956&hit=1&index=gi%7c00000001&px=1) | **747.3693** | **2239.0860** | **2237.1071** | **1.9788** | **0** | **(14)** | **0.0063** | **1** | **K.TLKPGTMSPEAFLQEAQVMK.K + 2 Oxidation (M)** |
|  | [1957](http://df3/mascot/cgi/peptide_view.pl?file=../data/20121119/F002569.dat&query=1957&hit=1&index=gi%7c00000001&px=1) | **747.4995** | **2239.4765** | **2238.0911** | **1.3854** | **0** | **(11)** | **0.0082** | **1** | **K.TLKPGTMSPEAFLQEAQVMK.K + Deamidation (NQ); 2 Oxidation (M)** |
|  | [1958](http://df3/mascot/cgi/peptide_view.pl?file=../data/20121119/F002569.dat&query=1958&hit=1&index=gi%7c00000001&px=1) | **747.6024** | **2239.7854** | **2238.0911** | **1.6943** | **0** | **(23)** | **0.00048** | **1** | **K.TLKPGTMSPEAFLQEAQVMK.K + Deamidation (NQ); 2 Oxidation (M)** |
|  | [2109](http://df3/mascot/cgi/peptide_view.pl?file=../data/20121119/F002569.dat&query=2109&hit=1&index=gi%7c00000001&px=1) | **767.2318** | **2298.6736** | **2298.2511** | **0.4225** | **1** | **11** | **0.0038** | **1** | **R.FTIKSDVWSFGILLTELTTK.G** |
|  | [2207](http://df3/mascot/cgi/peptide_view.pl?file=../data/20121119/F002569.dat&query=2207&hit=1&index=gi%7c00000001&px=1) | **778.8444** | **2333.5114** | **2333.2123** | **0.2992** | **1** | **(63)** | **6.9e-008** | **1** | **K.TLKPGTMSPEAFLQEAQVMKK.L** |
|  | [2245](http://df3/mascot/cgi/peptide_view.pl?file=../data/20121119/F002569.dat&query=2245&hit=1&index=gi%7c00000001&px=1) | **783.5038** | **2347.4895** | **2349.2072** | **-1.7176** | **1** | **(50)** | **5.4e-007** | **1** | **K.TLKPGTMSPEAFLQEAQVMKK.L + Oxidation (M)** |
|  | [2251](http://df3/mascot/cgi/peptide_view.pl?file=../data/20121119/F002569.dat&query=2251&hit=1&index=gi%7c00000001&px=1) | **784.3940** | **2350.1603** | **2349.2072** | **0.9531** | **1** | **(60)** | **1.6e-007** | **1** | **K.TLKPGTMSPEAFLQEAQVMKK.L + Oxidation (M)** |
|  | [2252](http://df3/mascot/cgi/peptide_view.pl?file=../data/20121119/F002569.dat&query=2252&hit=1&index=gi%7c00000001&px=1) | **784.4019** | **2350.1837** | **2350.1912** | **-0.0074** | **1** | **(35)** | **4.9e-005** | **1** | **K.TLKPGTMSPEAFLQEAQVMKK.L + Deamidation (NQ); Oxidation (M)** |
|  | [2254](http://df3/mascot/cgi/peptide_view.pl?file=../data/20121119/F002569.dat&query=2254&hit=1&index=gi%7c00000001&px=1) | **784.5188** | **2350.5346** | **2350.1912** | **0.3434** | **1** | **(64)** | **5.4e-008** | **1** | **K.TLKPGTMSPEAFLQEAQVMKK.L + Deamidation (NQ); Oxidation (M)** |
|  | [2255](http://df3/mascot/cgi/peptide_view.pl?file=../data/20121119/F002569.dat&query=2255&hit=1&index=gi%7c00000001&px=1) | **784.5248** | **2350.5525** | **2349.2072** | **1.3453** | **1** | **(52)** | **1e-006** | **1** | **K.TLKPGTMSPEAFLQEAQVMKK.L + Oxidation (M)** |
|  | [2257](http://df3/mascot/cgi/peptide_view.pl?file=../data/20121119/F002569.dat&query=2257&hit=1&index=gi%7c00000001&px=1) | **785.0137** | **2352.0192** | **2350.1912** | **1.8280** | **1** | **(52)** | **6.8e-007** | **1** | **K.TLKPGTMSPEAFLQEAQVMKK.L + Deamidation (NQ); Oxidation (M)** |
|  | [2281](http://df3/mascot/cgi/peptide_view.pl?file=../data/20121119/F002569.dat&query=2281&hit=1&index=gi%7c00000001&px=1) | **788.8229** | **2363.4470** | **2365.2021** | **-1.7551** | **1** | **74** | **1.9e-009** | **1** | **K.TLKPGTMSPEAFLQEAQVMKK.L + 2 Oxidation (M)** |
|  | [2293](http://df3/mascot/cgi/peptide_view.pl?file=../data/20121119/F002569.dat&query=2293&hit=1&index=gi%7c00000001&px=1) | **789.5388** | **2365.5946** | **2365.2021** | **0.3925** | **1** | **(54)** | **5.7e-007** | **1** | **K.TLKPGTMSPEAFLQEAQVMKK.L + 2 Oxidation (M)** |
|  | [2302](http://df3/mascot/cgi/peptide_view.pl?file=../data/20121119/F002569.dat&query=2302&hit=1&index=gi%7c00000001&px=1) | **789.8584** | **2366.5534** | **2365.2021** | **1.3513** | **1** | **(58)** | **2.5e-007** | **1** | **K.TLKPGTMSPEAFLQEAQVMKK.L + 2 Oxidation (M)** |
|  | [2303](http://df3/mascot/cgi/peptide_view.pl?file=../data/20121119/F002569.dat&query=2303&hit=1&index=gi%7c00000001&px=1) | **789.8641** | **2366.5706** | **2365.2021** | **1.3685** | **1** | **(56)** | **3.5e-007** | **1** | **K.TLKPGTMSPEAFLQEAQVMKK.L + 2 Oxidation (M)** |
|  | [2304](http://df3/mascot/cgi/peptide_view.pl?file=../data/20121119/F002569.dat&query=2304&hit=1&index=gi%7c00000001&px=1) | **789.8826** | **2366.6261** | **2365.2021** | **1.4240** | **1** | **(60)** | **1.4e-007** | **1** | **K.TLKPGTMSPEAFLQEAQVMKK.L + 2 Oxidation (M)** |
|  | [2306](http://df3/mascot/cgi/peptide_view.pl?file=../data/20121119/F002569.dat&query=2306&hit=1&index=gi%7c00000001&px=1) | **789.9331** | **2366.7775** | **2365.2021** | **1.5754** | **1** | **(64)** | **5.6e-008** | **1** | **K.TLKPGTMSPEAFLQEAQVMKK.L + 2 Oxidation (M)** |
|  | [2307](http://df3/mascot/cgi/peptide_view.pl?file=../data/20121119/F002569.dat&query=2307&hit=1&index=gi%7c00000001&px=1) | **790.2197** | **2367.6374** | **2366.1861** | **1.4513** | **1** | **(55)** | **2.8e-007** | **1** | **K.TLKPGTMSPEAFLQEAQVMKK.L + Deamidation (NQ); 2 Oxidation (M)** |
|  | [2549](http://df3/mascot/cgi/peptide_view.pl?file=../data/20121119/F002569.dat&query=2549&hit=1&index=gi%7c00000001&px=1) | **869.1798** | **2604.5176** | **2603.0194** | **1.4982** | **0** | **(60)** | **2.8e-007** | **1** | **R.MPCPPECPESLHDLMCQCWR.K + 4 Carbamidomethyl (C); Deamidation (NQ)** |
|  | [2563](http://df3/mascot/cgi/peptide_view.pl?file=../data/20121119/F002569.dat&query=2563&hit=1&index=gi%7c00000001&px=1) | **873.1738** | **2616.4997** | **2618.0303** | **-1.5306** | **0** | **(52)** | **3e-006** | **1** | **R.MPCPPECPESLHDLMCQCWR.K + 4 Carbamidomethyl (C); Oxidation (M)** |
|  | [2566](http://df3/mascot/cgi/peptide_view.pl?file=../data/20121119/F002569.dat&query=2566&hit=1&index=gi%7c00000001&px=1) | **873.7039** | **2618.0897** | **2618.0303** | **0.0594** | **0** | **61** | **3.7e-007** | **1** | **R.MPCPPECPESLHDLMCQCWR.K + 4 Carbamidomethyl (C); Oxidation (M)** |
|  | [2567](http://df3/mascot/cgi/peptide_view.pl?file=../data/20121119/F002569.dat&query=2567&hit=1&index=gi%7c00000001&px=1) | **873.7200** | **2618.1381** | **2618.0303** | **0.1078** | **0** | **(44)** | **2.2e-005** | **1** | **R.MPCPPECPESLHDLMCQCWR.K + 4 Carbamidomethyl (C); Oxidation (M)** |
|  | [2570](http://df3/mascot/cgi/peptide_view.pl?file=../data/20121119/F002569.dat&query=2570&hit=1&index=gi%7c00000001&px=1) | **874.0563** | **2619.1470** | **2618.0303** | **1.1167** | **0** | **(25)** | **0.0014** | **1** | **R.MPCPPECPESLHDLMCQCWR.K + 4 Carbamidomethyl (C); Oxidation (M)** |
|  | [2571](http://df3/mascot/cgi/peptide_view.pl?file=../data/20121119/F002569.dat&query=2571&hit=1&index=gi%7c00000001&px=1) | **874.0651** | **2619.1734** | **2617.3858** | **1.7875** | **1** | **(18)** | **0.0067** | **1** | **R.VAIKTLKPGTMSPEAFLQEAQVMK.K + Deamidation (NQ)** |
|  | [2586](http://df3/mascot/cgi/peptide_view.pl?file=../data/20121119/F002569.dat&query=2586&hit=1&index=gi%7c00000001&px=1) | **874.2067** | **2619.5982** | **2618.0303** | **1.5679** | **0** | **(11)** | **0.035** | **1** | **R.MPCPPECPESLHDLMCQCWR.K + 4 Carbamidomethyl (C); Oxidation (M)** |
|  | [2609](http://df3/mascot/cgi/peptide_view.pl?file=../data/20121119/F002569.dat&query=2609&hit=1&index=gi%7c00000001&px=1) | **874.4757** | **2620.4053** | **2619.0143** | **1.3910** | **0** | **(31)** | **0.0003** | **1** | **R.MPCPPECPESLHDLMCQCWR.K + 4 Carbamidomethyl (C); Deamidation (NQ); Oxidation (M)** |
|  | [2612](http://df3/mascot/cgi/peptide_view.pl?file=../data/20121119/F002569.dat&query=2612&hit=1&index=gi%7c00000001&px=1) | **874.5104** | **2620.5093** | **2619.0143** | **1.4950** | **0** | **(13)** | **0.016** | **1** | **R.MPCPPECPESLHDLMCQCWR.K + 4 Carbamidomethyl (C); Deamidation (NQ); Oxidation (M)** |
|  | [2618](http://df3/mascot/cgi/peptide_view.pl?file=../data/20121119/F002569.dat&query=2618&hit=1&index=gi%7c00000001&px=1) | **874.6229** | **2620.8469** | **2619.0143** | **1.8326** | **0** | **(36)** | **9.8e-005** | **1** | **R.MPCPPECPESLHDLMCQCWR.K + 4 Carbamidomethyl (C); Deamidation (NQ); Oxidation (M)** |
|  | [2628](http://df3/mascot/cgi/peptide_view.pl?file=../data/20121119/F002569.dat&query=2628&hit=1&index=gi%7c00000001&px=1) | **876.0945** | **2625.2616** | **2626.1940** | **-0.9323** | **0** | **(14)** | **0.028** | **1** | **R.MPCPPECPESLHDLMCQCWR.K + 2 Carbamidomethyl (C); 2 Oxidation (M); 2 HNE (H C)** |
|  | [2645](http://df3/mascot/cgi/peptide_view.pl?file=../data/20121119/F002569.dat&query=2645&hit=1&index=gi%7c00000001&px=1) | **878.6342** | **2632.8806** | **2632.3967** | **0.4839** | **1** | **(47)** | **7.9e-006** | **1** | **R.VAIKTLKPGTMSPEAFLQEAQVMK.K + Oxidation (M)** |
|  | [2647](http://df3/mascot/cgi/peptide_view.pl?file=../data/20121119/F002569.dat&query=2647&hit=1&index=gi%7c00000001&px=1) | **878.8530** | **2633.5373** | **2634.0252** | **-0.4880** | **0** | **(32)** | **0.00021** | **1** | **R.MPCPPECPESLHDLMCQCWR.K + 4 Carbamidomethyl (C); 2 Oxidation (M)** |
|  | [2649](http://df3/mascot/cgi/peptide_view.pl?file=../data/20121119/F002569.dat&query=2649&hit=1&index=gi%7c00000001&px=1) | **879.0406** | **2634.1001** | **2633.0663** | **1.0338** | **1** | **(17)** | **0.0056** | **1** | **R.MPCPPECPESLHDLMCQCWRK.E + 2 Carbamidomethyl (C); Deamidation (NQ); Oxidation (M)** |
|  | [2652](http://df3/mascot/cgi/peptide_view.pl?file=../data/20121119/F002569.dat&query=2652&hit=1&index=gi%7c00000001&px=1) | **879.1395** | **2634.3967** | **2633.0663** | **1.3304** | **1** | **(11)** | **0.023** | **1** | **R.MPCPPECPESLHDLMCQCWRK.E + 2 Carbamidomethyl (C); Deamidation (NQ); Oxidation (M)** |
|  | [2655](http://df3/mascot/cgi/peptide_view.pl?file=../data/20121119/F002569.dat&query=2655&hit=1&index=gi%7c00000001&px=1) | **879.3771** | **2635.1094** | **2634.0252** | **1.0842** | **0** | **(34)** | **8.7e-005** | **1** | **R.MPCPPECPESLHDLMCQCWR.K + 4 Carbamidomethyl (C); 2 Oxidation (M)** |
|  | [2656](http://df3/mascot/cgi/peptide_view.pl?file=../data/20121119/F002569.dat&query=2656&hit=1&index=gi%7c00000001&px=1) | **879.5692** | **2635.6858** | **2634.0252** | **1.6606** | **0** | **(43)** | **1e-005** | **1** | **R.MPCPPECPESLHDLMCQCWR.K + 4 Carbamidomethyl (C); 2 Oxidation (M)** |
|  | [2661](http://df3/mascot/cgi/peptide_view.pl?file=../data/20121119/F002569.dat&query=2661&hit=1&index=gi%7c00000001&px=1) | **879.8784** | **2636.6134** | **2635.0092** | **1.6042** | **0** | **(33)** | **8.4e-005** | **1** | **R.MPCPPECPESLHDLMCQCWR.K + 4 Carbamidomethyl (C); Deamidation (NQ); 2 Oxidation (M)** |
|  | [2684](http://df3/mascot/cgi/peptide_view.pl?file=../data/20121119/F002569.dat&query=2684&hit=1&index=gi%7c00000001&px=1) | **883.6641** | **2647.9704** | **2649.0612** | **-1.0909** | **1** | **(10)** | **0.03** | **1** | **R.MPCPPECPESLHDLMCQCWRK.E + 2 Carbamidomethyl (C); Deamidation (NQ); 2 Oxidation (M)** |
|  | [2687](http://df3/mascot/cgi/peptide_view.pl?file=../data/20121119/F002569.dat&query=2687&hit=1&index=gi%7c00000001&px=1) | **884.1475** | **2649.4206** | **2648.3917** | **1.0289** | **1** | **65** | **1.2e-007** | **1** | **R.VAIKTLKPGTMSPEAFLQEAQVMK.K + 2 Oxidation (M)** |
|  | [2691](http://df3/mascot/cgi/peptide_view.pl?file=../data/20121119/F002569.dat&query=2691&hit=1&index=gi%7c00000001&px=1) | **884.5068** | **2650.4987** | **2649.0612** | **1.4374** | **1** | **36** | **6.7e-005** | **1** | **R.MPCPPECPESLHDLMCQCWRK.E + 2 Carbamidomethyl (C); Deamidation (NQ); 2 Oxidation (M)** |
|  | [2693](http://df3/mascot/cgi/peptide_view.pl?file=../data/20121119/F002569.dat&query=2693&hit=1&index=gi%7c00000001&px=1) | **884.6426** | **2650.9059** | **2649.3757** | **1.5302** | **1** | **(53)** | **1.4e-006** | **1** | **R.VAIKTLKPGTMSPEAFLQEAQVMK.K + Deamidation (NQ); 2 Oxidation (M)** |
|  | [2792](http://df3/mascot/cgi/peptide_view.pl?file=../data/20121119/F002569.dat&query=2792&hit=1&index=gi%7c00000001&px=1) | **923.2971** | **2766.8695** | **2768.5436** | **-1.6740** | **1** | **(14)** | **0.019** | **1** | **K.HADGLCHRLTTVCPTSKPQTQGLAK.D + Deamidation (NQ); 2 HNE (H C)** |
|  | [2793](http://df3/mascot/cgi/peptide_view.pl?file=../data/20121119/F002569.dat&query=2793&hit=1&index=gi%7c00000001&px=1) | **923.5371** | **2767.5895** | **2767.5596** | **0.0299** | **1** | **24** | **0.0027** | **1** | **K.HADGLCHRLTTVCPTSKPQTQGLAK.D + 2 HNE (H C)** |
|  | [2832](http://df3/mascot/cgi/peptide_view.pl?file=../data/20121119/F002569.dat&query=2832&hit=1&index=gi%7c00000001&px=1) | **1034.5007** | **3100.4804** | **3099.4757** | **1.0046** | **0** | **102** | **9.1e-012** | **1** | **R.SLEPAENVHGAGGGAFPASQTPSKPASADGHR.G** |
|  | [2833](http://df3/mascot/cgi/peptide_view.pl?file=../data/20121119/F002569.dat&query=2833&hit=1&index=gi%7c00000001&px=1) | **1034.5428** | **3100.6067** | **3100.4597** | **0.1470** | **0** | **(92)** | **9e-011** | **1** | **R.SLEPAENVHGAGGGAFPASQTPSKPASADGHR.G + Deamidation (NQ)** |
|  | [2834](http://df3/mascot/cgi/peptide_view.pl?file=../data/20121119/F002569.dat&query=2834&hit=1&index=gi%7c00000001&px=1) | **1034.7202** | **3101.1388** | **3100.4597** | **0.6791** | **0** | **(96)** | **3.7e-011** | **1** | **R.SLEPAENVHGAGGGAFPASQTPSKPASADGHR.G + Deamidation (NQ)** |

**Peptide matches not assigned to protein hits:** (no details means no match)

|  | **Query** | **Observed** | **Mr(expt)** | **Mr(calc)** | **Delta** | **Miss** | **Score** | **Expect** | **Rank** | **Peptide** |
| --- | --- | --- | --- | --- | --- | --- | --- | --- | --- | --- |
|  | [1349](http://df3/mascot/cgi/peptide_view.pl?file=../data/20121119/F002569.dat&query=1349&hit=1&index=&px=1) | **643.0876** | **1284.1606** | **1283.6509** | **0.5097** | **0** | **10** | **0.005** | **1** | **GPSAAFAPAAAEPK** |
|  | [424](http://df3/mascot/cgi/peptide_view.pl?file=../data/20121119/F002569.dat&query=424&hit=1&index=&px=1) | **447.0421** | **892.0696** | **891.5065** | **0.5631** | **0** | **10** | **0.0051** | **1** | **GSLLDFLK** |
|  | [1902](http://df3/mascot/cgi/peptide_view.pl?file=../data/20121119/F002569.dat&query=1902&hit=1&index=&px=1) | **742.3690** | **2224.0850** | **2223.0802** | **1.0048** | **0** | **10** | **0.016** | **1** | **TLKPGTMSPEAFLQEAQVMK + 2 Deamidation (NQ); Oxidation (M)** |
|  | [2712](http://df3/mascot/cgi/peptide_view.pl?file=../data/20121119/F002569.dat&query=2712&hit=1&index=&px=1) | **889.4164** | **2665.2273** | **2664.2889** | **0.9384** | **0** | **9** | **0.049** | **1** | **MPCPPECPESLHDLMCQCWR + 2 Carbamidomethyl (C); Deamidation (NQ); Oxidation (M); 3 HNE (H C)** |
|  | [2305](http://df3/mascot/cgi/peptide_view.pl?file=../data/20121119/F002569.dat&query=2305&hit=1&index=&px=1) | **789.8954** | **2366.6643** | **2366.1861** | **0.4782** | **1** | **9** | **0.02** | **1** | **TLKPGTMSPEAFLQEAQVMKK + Deamidation (NQ); 2 Oxidation (M)** |
|  | [1939](http://df3/mascot/cgi/peptide_view.pl?file=../data/20121119/F002569.dat&query=1939&hit=1&index=&px=1) | **746.3306** | **2235.9699** | **2237.1071** | **-1.1373** | **0** | **9** | **0.02** | **1** | **TLKPGTMSPEAFLQEAQVMK + 2 Oxidation (M)** |
|  | [1218](http://df3/mascot/cgi/peptide_view.pl?file=../data/20121119/F002569.dat&query=1218&hit=1&index=&px=1) | **610.8628** | **1829.5666** | **1828.9717** | **0.5949** | **0** | **8** | **0.035** | **1** | **LTTVCPTSKPQTQGLAK + Carbamidomethyl (C)** |
|  | [1938](http://df3/mascot/cgi/peptide_view.pl?file=../data/20121119/F002569.dat&query=1938&hit=1&index=&px=1) | **746.3143** | **2235.9212** | **2237.1071** | **-1.1860** | **0** | **8** | **0.022** | **1** | **TLKPGTMSPEAFLQEAQVMK + 2 Oxidation (M)** |
|  | [1806](http://df3/mascot/cgi/peptide_view.pl?file=../data/20121119/F002569.dat&query=1806&hit=1&index=&px=1) | **735.6602** | **2203.9588** | **2205.1173** | **-1.1585** | **0** | **8** | **0.0072** | **1** | **TLKPGTMSPEAFLQEAQVMK** |
|  | [2578](http://df3/mascot/cgi/peptide_view.pl?file=../data/20121119/F002569.dat&query=2578&hit=1&index=&px=1) | **874.1437** | **2619.4092** | **2619.0143** | **0.3949** | **0** | **8** | **0.059** | **1** | **MPCPPECPESLHDLMCQCWR + 4 Carbamidomethyl (C); Deamidation (NQ); Oxidation (M)** |
|  | [1868](http://df3/mascot/cgi/peptide_view.pl?file=../data/20121119/F002569.dat&query=1868&hit=1&index=&px=1) | **740.7848** | **2219.3325** | **2221.1122** | **-1.7797** | **0** | **8** | **0.022** | **1** | **TLKPGTMSPEAFLQEAQVMK + Oxidation (M)** |
|  | [315](http://df3/mascot/cgi/peptide_view.pl?file=../data/20121119/F002569.dat&query=315&hit=1&index=&px=1) | **420.0253** | **1257.0541** | **1256.6877** | **0.3664** | **1** | **8** | **0.017** | **1** | **MNYVHRDLR + Deamidation (NQ); HNE (H C)** |
|  | [455](http://df3/mascot/cgi/peptide_view.pl?file=../data/20121119/F002569.dat&query=455&hit=1&index=&px=1) | **449.1220** | **1344.3442** | **1342.6881** | **1.6561** | **1** | **8** | **0.033** | **1** | **KLDSGGFYITSR** |
|  | [2569](http://df3/mascot/cgi/peptide_view.pl?file=../data/20121119/F002569.dat&query=2569&hit=1&index=&px=1) | **874.0189** | **2619.0349** | **2618.0303** | **1.0046** | **0** | **8** | **0.076** | **1** | **MPCPPECPESLHDLMCQCWR + 4 Carbamidomethyl (C); Oxidation (M)** |
|  | [1213](http://df3/mascot/cgi/peptide_view.pl?file=../data/20121119/F002569.dat&query=1213&hit=1&index=&px=1) | **610.2587** | **1827.7542** | **1828.9717** | **-1.2175** | **0** | **8** | **0.043** | **1** | **LTTVCPTSKPQTQGLAK + Carbamidomethyl (C)** |
|  | [1940](http://df3/mascot/cgi/peptide_view.pl?file=../data/20121119/F002569.dat&query=1940&hit=1&index=&px=1) | **746.3319** | **2235.9739** | **2237.1071** | **-1.1332** | **0** | **7** | **0.03** | **1** | **TLKPGTMSPEAFLQEAQVMK + 2 Oxidation (M)** |
|  | [1585](http://df3/mascot/cgi/peptide_view.pl?file=../data/20121119/F002569.dat&query=1585&hit=1&index=&px=1) | **702.0408** | **1402.0671** | **1400.7333** | **1.3338** | **0** | **7** | **0.021** | **1** | **AANILVGENLVCK + Carbamidomethyl (C); Deamidation (NQ)** |
|  | [2589](http://df3/mascot/cgi/peptide_view.pl?file=../data/20121119/F002569.dat&query=2589&hit=1&index=&px=1) | **874.2308** | **2619.6707** | **2618.0303** | **1.6404** | **0** | **7** | **0.088** | **1** | **MPCPPECPESLHDLMCQCWR + 4 Carbamidomethyl (C); Oxidation (M)** |
|  | [1808](http://df3/mascot/cgi/peptide_view.pl?file=../data/20121119/F002569.dat&query=1808&hit=1&index=&px=1) | **736.0942** | **2205.2609** | **2207.0853** | **-1.8244** | **0** | **6** | **0.035** | **1** | **TLKPGTMSPEAFLQEAQVMK + 2 Deamidation (NQ)** |
|  | [1931](http://df3/mascot/cgi/peptide_view.pl?file=../data/20121119/F002569.dat&query=1931&hit=1&index=&px=1) | **746.1218** | **2235.3436** | **2237.1071** | **-1.7635** | **0** | **6** | **0.036** | **1** | **TLKPGTMSPEAFLQEAQVMK + 2 Oxidation (M)** |
|  | [1586](http://df3/mascot/cgi/peptide_view.pl?file=../data/20121119/F002569.dat&query=1586&hit=1&index=&px=1) | **702.1003** | **1402.1861** | **1400.7333** | **1.4529** | **0** | **6** | **0.024** | **1** | **AANILVGENLVCK + Carbamidomethyl (C); Deamidation (NQ)** |
|  | [589](http://df3/mascot/cgi/peptide_view.pl?file=../data/20121119/F002569.dat&query=589&hit=1&index=&px=1) | **467.6053** | **1399.7941** | **1399.7493** | **0.0449** | **0** | **6** | **0.049** | **1** | **AANILVGENLVCK + Carbamidomethyl (C)** |
|  | [485](http://df3/mascot/cgi/peptide_view.pl?file=../data/20121119/F002569.dat&query=485&hit=1&index=&px=1) | **457.8634** | **1370.5685** | **1370.6942** | **-0.1258** | **1** | **6** | **0.013** | **1** | **DAWEIPRESLR** |
|  | [776](http://df3/mascot/cgi/peptide_view.pl?file=../data/20121119/F002569.dat&query=776&hit=1&index=&px=1) | **517.7076** | **1033.4006** | **1031.5222** | **1.8784** | **0** | **6** | **0.027** | **1** | **VPYPGMVNR** |
|  | [1569](http://df3/mascot/cgi/peptide_view.pl?file=../data/20121119/F002569.dat&query=1569&hit=1&index=&px=1) | **700.3864** | **1398.7582** | **1399.7493** | **-0.9911** | **0** | **5** | **0.085** | **1** | **AANILVGENLVCK + Carbamidomethyl (C)** |
|  | [2588](http://df3/mascot/cgi/peptide_view.pl?file=../data/20121119/F002569.dat&query=2588&hit=1&index=&px=1) | **874.2177** | **2619.6311** | **2619.3467** | **0.2844** | **0** | **5** | **0.11** | **1** | **MPCPPECPESLHDLMCQCWR + Deamidation (NQ); 2 Oxidation (M); 4 HNE (H C)** |
|  | [2759](http://df3/mascot/cgi/peptide_view.pl?file=../data/20121119/F002569.dat&query=2759&hit=1&index=&px=1) | **900.4445** | **2698.3115** | **2697.3796** | **0.9320** | **1** | **5** | **0.099** | **1** | **LTTVCPTSKPQTQGLAKDAWEIPR + Carbamidomethyl (C); Deamidation (NQ)** |
|  | [1880](http://df3/mascot/cgi/peptide_view.pl?file=../data/20121119/F002569.dat&query=1880&hit=1&index=&px=1) | **741.4321** | **2221.2746** | **2222.0962** | **-0.8217** | **0** | **5** | **0.071** | **1** | **TLKPGTMSPEAFLQEAQVMK + Deamidation (NQ); Oxidation (M)** |
|  | [2651](http://df3/mascot/cgi/peptide_view.pl?file=../data/20121119/F002569.dat&query=2651&hit=1&index=&px=1) | **879.1031** | **2634.2876** | **2634.0252** | **0.2624** | **0** | **5** | **0.092** | **1** | **MPCPPECPESLHDLMCQCWR + 4 Carbamidomethyl (C); 2 Oxidation (M)** |
|  | [585](http://df3/mascot/cgi/peptide_view.pl?file=../data/20121119/F002569.dat&query=585&hit=1&index=&px=1) | **467.3909** | **1399.1508** | **1400.7333** | **-1.5825** | **0** | **5** | **0.048** | **1** | **AANILVGENLVCK + Carbamidomethyl (C); Deamidation (NQ)** |
|  | [833](http://df3/mascot/cgi/peptide_view.pl?file=../data/20121119/F002569.dat&query=833&hit=1&index=&px=1) | **521.5059** | **1040.9973** | **1039.5662** | **1.4311** | **0** | **5** | **0.033** | **1** | **LLLNAENPR + Deamidation (NQ)** |
|  | [1935](http://df3/mascot/cgi/peptide_view.pl?file=../data/20121119/F002569.dat&query=1935&hit=1&index=&px=1) | **746.2965** | **1490.5785** | **1489.6394** | **0.9390** | **0** | **5** | **0.051** | **1** | **GAYCLSVSDFDNAK + Deamidation (NQ)** |
|  | [457](http://df3/mascot/cgi/peptide_view.pl?file=../data/20121119/F002569.dat&query=457&hit=1&index=&px=1) | **449.1304** | **1344.3694** | **1342.6881** | **1.6813** | **1** | **5** | **0.068** | **1** | **KLDSGGFYITSR** |
|  | [1813](http://df3/mascot/cgi/peptide_view.pl?file=../data/20121119/F002569.dat&query=1813&hit=1&index=&px=1) | **736.9771** | **2207.9093** | **2206.1013** | **1.8080** | **0** | **5** | **0.034** | **1** | **TLKPGTMSPEAFLQEAQVMK + Deamidation (NQ)** |
|  | [1883](http://df3/mascot/cgi/peptide_view.pl?file=../data/20121119/F002569.dat&query=1883&hit=1&index=&px=1) | **741.4519** | **2221.3339** | **2222.0962** | **-0.7623** | **0** | **5** | **0.087** | **1** | **TLKPGTMSPEAFLQEAQVMK + Deamidation (NQ); Oxidation (M)** |
|  | [2762](http://df3/mascot/cgi/peptide_view.pl?file=../data/20121119/F002569.dat&query=2762&hit=1&index=&px=1) | **901.7753** | **2702.3040** | **2701.1831** | **1.1209** | **1** | **4** | **0.072** | **1** | **MPCPPECPESLHDLMCQCWRK + 2 Carbamidomethyl (C); 2 Oxidation (M); HNE (H C)** |
|  | [2809](http://df3/mascot/cgi/peptide_view.pl?file=../data/20121119/F002569.dat&query=2809&hit=1&index=&px=1) | **946.9448** | **2837.8126** | **2837.6198** | **0.1928** | **1** | **4** | **0.11** | **1** | **TQFNSLQQLVAYYSKHADGLCHR + 3 HNE (H C)** |
|  | [514](http://df3/mascot/cgi/peptide_view.pl?file=../data/20121119/F002569.dat&query=514&hit=1&index=&px=1) | **461.7867** | **921.5589** | **922.4872** | **-0.9282** | **1** | **4** | **0.018** | **1** | **GETGKYLR** |
|  | [1933](http://df3/mascot/cgi/peptide_view.pl?file=../data/20121119/F002569.dat&query=1933&hit=1&index=&px=1) | **746.2486** | **1490.4826** | **1489.6394** | **0.8432** | **0** | **4** | **0.056** | **1** | **GAYCLSVSDFDNAK + Deamidation (NQ)** |
|  | [2247](http://df3/mascot/cgi/peptide_view.pl?file=../data/20121119/F002569.dat&query=2247&hit=1&index=&px=1) | **783.8417** | **2348.5034** | **2349.2072** | **-0.7038** | **1** | **4** | **0.038** | **1** | **TLKPGTMSPEAFLQEAQVMKK + Oxidation (M)** |
|  | [710](http://df3/mascot/cgi/peptide_view.pl?file=../data/20121119/F002569.dat&query=710&hit=1&index=&px=1) | **495.7395** | **989.4645** | **987.4872** | **1.9772** | **0** | **4** | **0.02** | **1** | **EVLDQVER + Deamidation (NQ)** |
|  | [2760](http://df3/mascot/cgi/peptide_view.pl?file=../data/20121119/F002569.dat&query=2760&hit=1&index=&px=1) | **900.8518** | **2699.5336** | **2698.2515** | **1.2821** | **1** | **4** | **0.1** | **1** | **MPCPPECPESLHDLMCQCWRK + Carbamidomethyl (C); Deamidation (NQ); 2 Oxidation (M); 2 HNE (H C)** |
|  | [452](http://df3/mascot/cgi/peptide_view.pl?file=../data/20121119/F002569.dat&query=452&hit=1&index=&px=1) | **449.0148** | **1344.0227** | **1342.6881** | **1.3346** | **1** | **4** | **0.082** | **1** | **KLDSGGFYITSR** |
|  | [412](http://df3/mascot/cgi/peptide_view.pl?file=../data/20121119/F002569.dat&query=412&hit=1&index=&px=1) | **445.1003** | **888.1859** | **888.4705** | **-0.2845** | **0** | **4** | **0.083** | **1** | **MNYVHR + Deamidation (NQ); Oxidation (M); HNE (H C)** |
|  | [448](http://df3/mascot/cgi/peptide_view.pl?file=../data/20121119/F002569.dat&query=448&hit=1&index=&px=1) | **448.2333** | **1341.6782** | **1342.7278** | **-1.0496** | **0** | **4** | **0.042** | **1** | **AANILVGENLVCK** |
|  | [835](http://df3/mascot/cgi/peptide_view.pl?file=../data/20121119/F002569.dat&query=835&hit=1&index=&px=1) | **522.0476** | **1042.0807** | **1040.5502** | **1.5305** | **0** | **4** | **0.021** | **1** | **LLLNAENPR + 2 Deamidation (NQ)** |
|  | [2112](http://df3/mascot/cgi/peptide_view.pl?file=../data/20121119/F002569.dat&query=2112&hit=1&index=&px=1) | **767.4974** | **2299.4703** | **2298.2511** | **1.2192** | **1** | **4** | **0.022** | **1** | **FTIKSDVWSFGILLTELTTK** |
|  | [832](http://df3/mascot/cgi/peptide_view.pl?file=../data/20121119/F002569.dat&query=832&hit=1&index=&px=1) | **521.4724** | **1040.9301** | **1039.5662** | **1.3640** | **0** | **4** | **0.044** | **1** | **LLLNAENPR + Deamidation (NQ)** |
|  | [2394](http://df3/mascot/cgi/peptide_view.pl?file=../data/20121119/F002569.dat&query=2394&hit=1&index=&px=1) | **847.9140** | **2540.7202** | **2541.0983** | **-0.3781** | **0** | **4** | **0.088** | **1** | **MPCPPECPESLHDLMCQCWR + 2 Carbamidomethyl (C); HNE (H C)** |
|  | [2686](http://df3/mascot/cgi/peptide_view.pl?file=../data/20121119/F002569.dat&query=2686&hit=1&index=&px=1) | **884.0714** | **2649.1924** | **2649.0612** | **0.1312** | **1** | **4** | **0.15** | **1** | **MPCPPECPESLHDLMCQCWRK + 2 Carbamidomethyl (C); Deamidation (NQ); 2 Oxidation (M)** |
|  | [1235](http://df3/mascot/cgi/peptide_view.pl?file=../data/20121119/F002569.dat&query=1235&hit=1&index=&px=1) | **613.3723** | **1224.7300** | **1223.5670** | **1.1630** | **0** | **3** | **0.022** | **1** | **LIEDNEYTAR + Deamidation (NQ)** |
|  | [1115](http://df3/mascot/cgi/peptide_view.pl?file=../data/20121119/F002569.dat&query=1115&hit=1&index=&px=1) | **592.6107** | **1774.8103** | **1772.9342** | **1.8761** | **0** | **3** | **0.045** | **1** | **LTTVCPTSKPQTQGLAK + Deamidation (NQ)** |
|  | [2785](http://df3/mascot/cgi/peptide_view.pl?file=../data/20121119/F002569.dat&query=2785&hit=1&index=&px=1) | **916.6152** | **1831.2159** | **1829.9557** | **1.2602** | **0** | **3** | **0.19** | **1** | **LTTVCPTSKPQTQGLAK + Carbamidomethyl (C); Deamidation (NQ)** |
|  | [651](http://df3/mascot/cgi/peptide_view.pl?file=../data/20121119/F002569.dat&query=651&hit=1&index=&px=1) | **480.8804** | **959.7462** | **960.5141** | **-0.7679** | **0** | **3** | **0.07** | **1** | **HADGLCHR + HNE (H C)** |
|  | [2695](http://df3/mascot/cgi/peptide_view.pl?file=../data/20121119/F002569.dat&query=2695&hit=1&index=&px=1) | **884.7198** | **2651.1377** | **2651.2256** | **-0.0879** | **0** | **3** | **0.094** | **1** | **MPCPPECPESLHDLMCQCWR + 3 Carbamidomethyl (C); 2 HNE (H C)** |
|  | [2404](http://df3/mascot/cgi/peptide_view.pl?file=../data/20121119/F002569.dat&query=2404&hit=1&index=&px=1) | **851.9438** | **2552.8097** | **2554.1616** | **-1.3519** | **0** | **3** | **0.097** | **1** | **MPCPPECPESLHDLMCQCWR + Carbamidomethyl (C); Deamidation (NQ); Oxidation (M); 2 HNE (H C)** |
|  | [1160](http://df3/mascot/cgi/peptide_view.pl?file=../data/20121119/F002569.dat&query=1160&hit=1&index=&px=1) | **597.4185** | **1789.2335** | **1790.8726** | **-1.6391** | **0** | **3** | **0.075** | **1** | **TQFNSLQQLVAYYSK + 2 Deamidation (NQ)** |
|  | [317](http://df3/mascot/cgi/peptide_view.pl?file=../data/20121119/F002569.dat&query=317&hit=1&index=&px=1) | **420.0847** | **1257.2321** | **1256.6877** | **0.5445** | **1** | **3** | **0.05** | **1** | **MNYVHRDLR + Deamidation (NQ); HNE (H C)** |
|  | [2539](http://df3/mascot/cgi/peptide_view.pl?file=../data/20121119/F002569.dat&query=2539&hit=1&index=&px=1) | **865.1669** | **2592.4788** | **2592.0398** | **0.4390** | **1** | **3** | **0.25** | **1** | **MPCPPECPESLHDLMCQCWRK + Carbamidomethyl (C); Deamidation (NQ); 2 Oxidation (M)** |
|  | [277](http://df3/mascot/cgi/peptide_view.pl?file=../data/20121119/F002569.dat&query=277&hit=1&index=&px=1) | **405.9259** | **1214.7560** | **1214.5931** | **0.1629** | **0** | **3** | **0.026** | **1** | **LDSGGFYITSR** |
|  | [2405](http://df3/mascot/cgi/peptide_view.pl?file=../data/20121119/F002569.dat&query=2405&hit=1&index=&px=1) | **852.4189** | **2554.2350** | **2554.1616** | **0.0734** | **0** | **3** | **0.21** | **1** | **MPCPPECPESLHDLMCQCWR + Carbamidomethyl (C); Deamidation (NQ); Oxidation (M); 2 HNE (H C)** |
|  | [2555](http://df3/mascot/cgi/peptide_view.pl?file=../data/20121119/F002569.dat&query=2555&hit=1&index=&px=1) | **871.3404** | **2610.9994** | **2611.3137** | **-0.3144** | **1** | **3** | **0.29** | **1** | **YLRLPQLVDMAAQIASGMAYVER + Deamidation (NQ); Oxidation (M)** |
|  | [2408](http://df3/mascot/cgi/peptide_view.pl?file=../data/20121119/F002569.dat&query=2408&hit=1&index=&px=1) | **853.1340** | **2556.3801** | **2555.1503** | **1.2298** | **1** | **3** | **0.22** | **1** | **MPCPPECPESLHDLMCQCWRK + HNE (H C)** |
|  | [2406](http://df3/mascot/cgi/peptide_view.pl?file=../data/20121119/F002569.dat&query=2406&hit=1&index=&px=1) | **853.0034** | **2555.9884** | **2554.1616** | **1.8269** | **0** | **3** | **0.22** | **1** | **MPCPPECPESLHDLMCQCWR + Carbamidomethyl (C); Deamidation (NQ); Oxidation (M); 2 HNE (H C)** |
|  | [1874](http://df3/mascot/cgi/peptide_view.pl?file=../data/20121119/F002569.dat&query=1874&hit=1&index=&px=1) | **741.2393** | **2220.6959** | **2222.0962** | **-1.4003** | **0** | **3** | **0.11** | **1** | **TLKPGTMSPEAFLQEAQVMK + Deamidation (NQ); Oxidation (M)** |
|  | [2545](http://df3/mascot/cgi/peptide_view.pl?file=../data/20121119/F002569.dat&query=2545&hit=1&index=&px=1) | **866.2222** | **2595.6447** | **2594.2041** | **1.4406** | **0** | **3** | **0.22** | **1** | **MPCPPECPESLHDLMCQCWR + 2 Carbamidomethyl (C); 2 HNE (H C)** |
|  | [2581](http://df3/mascot/cgi/peptide_view.pl?file=../data/20121119/F002569.dat&query=2581&hit=1&index=&px=1) | **874.1558** | **2619.4455** | **2618.0303** | **1.4152** | **0** | **2** | **0.23** | **1** | **MPCPPECPESLHDLMCQCWR + 4 Carbamidomethyl (C); Oxidation (M)** |
|  | [2596](http://df3/mascot/cgi/peptide_view.pl?file=../data/20121119/F002569.dat&query=2596&hit=1&index=&px=1) | **874.3206** | **2619.9398** | **2618.0303** | **1.9095** | **0** | **2** | **0.23** | **1** | **MPCPPECPESLHDLMCQCWR + 4 Carbamidomethyl (C); Oxidation (M)** |
|  | [819](http://df3/mascot/cgi/peptide_view.pl?file=../data/20121119/F002569.dat&query=819&hit=1&index=&px=1) | **520.7670** | **1039.5194** | **1038.5822** | **0.9372** | **0** | **2** | **0.086** | **1** | **LLLNAENPR** |
|  | [2556](http://df3/mascot/cgi/peptide_view.pl?file=../data/20121119/F002569.dat&query=2556&hit=1&index=&px=1) | **871.3477** | **2611.0211** | **2611.3137** | **-0.2926** | **1** | **2** | **0.32** | **1** | **YLRLPQLVDMAAQIASGMAYVER + Deamidation (NQ); Oxidation (M)** |
|  | [2594](http://df3/mascot/cgi/peptide_view.pl?file=../data/20121119/F002569.dat&query=2594&hit=1&index=&px=1) | **874.3040** | **2619.8900** | **2618.0303** | **1.8597** | **0** | **2** | **0.23** | **1** | **MPCPPECPESLHDLMCQCWR + 4 Carbamidomethyl (C); Oxidation (M)** |
|  | [1936](http://df3/mascot/cgi/peptide_view.pl?file=../data/20121119/F002569.dat&query=1936&hit=1&index=&px=1) | **746.3098** | **2235.9074** | **2237.1071** | **-1.1997** | **0** | **2** | **0.088** | **1** | **TLKPGTMSPEAFLQEAQVMK + 2 Oxidation (M)** |
|  | [2607](http://df3/mascot/cgi/peptide_view.pl?file=../data/20121119/F002569.dat&query=2607&hit=1&index=&px=1) | **874.4517** | **2620.3332** | **2618.3698** | **1.9633** | **1** | **2** | **0.31** | **1** | **VAIKTLKPGTMSPEAFLQEAQVMK + 2 Deamidation (NQ)** |
|  | [1146](http://df3/mascot/cgi/peptide_view.pl?file=../data/20121119/F002569.dat&query=1146&hit=1&index=&px=1) | **594.9341** | **1781.7804** | **1782.0138** | **-0.2333** | **1** | **2** | **0.094** | **1** | **DLRAANILVGENLVCK + 2 Deamidation (NQ); HNE (H C)** |
|  | [2646](http://df3/mascot/cgi/peptide_view.pl?file=../data/20121119/F002569.dat&query=2646&hit=1&index=&px=1) | **878.7070** | **2633.0993** | **2632.0823** | **1.0169** | **1** | **2** | **0.22** | **1** | **MPCPPECPESLHDLMCQCWRK + 2 Carbamidomethyl (C); Oxidation (M)** |
|  | [2587](http://df3/mascot/cgi/peptide_view.pl?file=../data/20121119/F002569.dat&query=2587&hit=1&index=&px=1) | **874.2135** | **2619.6187** | **2618.0303** | **1.5884** | **0** | **2** | **0.25** | **1** | **MPCPPECPESLHDLMCQCWR + 4 Carbamidomethyl (C); Oxidation (M)** |
|  | [2253](http://df3/mascot/cgi/peptide_view.pl?file=../data/20121119/F002569.dat&query=2253&hit=1&index=&px=1) | **784.4279** | **2350.2619** | **2349.2072** | **1.0548** | **1** | **2** | **0.096** | **1** | **TLKPGTMSPEAFLQEAQVMKK + Oxidation (M)** |
|  | [1872](http://df3/mascot/cgi/peptide_view.pl?file=../data/20121119/F002569.dat&query=1872&hit=1&index=&px=1) | **741.2039** | **2220.5897** | **2220.9844** | **-0.3947** | **1** | **2** | **0.13** | **1** | **ESETTKGAYCLSVSDFDNAK + Carbamidomethyl (C)** |
|  | [2599](http://df3/mascot/cgi/peptide_view.pl?file=../data/20121119/F002569.dat&query=2599&hit=1&index=&px=1) | **874.3413** | **2620.0021** | **2618.3698** | **1.6323** | **1** | **2** | **0.26** | **1** | **VAIKTLKPGTMSPEAFLQEAQVMK + 2 Deamidation (NQ)** |
|  | [2608](http://df3/mascot/cgi/peptide_view.pl?file=../data/20121119/F002569.dat&query=2608&hit=1&index=&px=1) | **874.4745** | **2620.4016** | **2619.3467** | **1.0549** | **0** | **2** | **0.23** | **1** | **MPCPPECPESLHDLMCQCWR + Deamidation (NQ); 2 Oxidation (M); 4 HNE (H C)** |
|  | [2743](http://df3/mascot/cgi/peptide_view.pl?file=../data/20121119/F002569.dat&query=2743&hit=1&index=&px=1) | **896.8688** | **2687.5845** | **2686.1722** | **1.4123** | **1** | **2** | **0.3** | **1** | **MPCPPECPESLHDLMCQCWRK + 2 Carbamidomethyl (C); Deamidation (NQ); Oxidation (M); HNE (H C)** |
|  | [1591](http://df3/mascot/cgi/peptide_view.pl?file=../data/20121119/F002569.dat&query=1591&hit=1&index=&px=1) | **702.3689** | **1402.7232** | **1400.7333** | **1.9900** | **0** | **2** | **0.067** | **1** | **AANILVGENLVCK + Carbamidomethyl (C); Deamidation (NQ)** |
|  | [2535](http://df3/mascot/cgi/peptide_view.pl?file=../data/20121119/F002569.dat&query=2535&hit=1&index=&px=1) | **864.9459** | **2591.8158** | **2591.0558** | **0.7600** | **1** | **2** | **0.34** | **1** | **MPCPPECPESLHDLMCQCWRK + Carbamidomethyl (C); 2 Oxidation (M)** |
|  | [2548](http://df3/mascot/cgi/peptide_view.pl?file=../data/20121119/F002569.dat&query=2548&hit=1&index=&px=1) | **869.0428** | **2604.1067** | **2602.3678** | **1.7389** | **0** | **2** | **0.17** | **1** | **MPCPPECPESLHDLMCQCWR + Oxidation (M); 4 HNE (H C)** |
|  | [2706](http://df3/mascot/cgi/peptide_view.pl?file=../data/20121119/F002569.dat&query=2706&hit=1&index=&px=1) | **885.9316** | **2654.7731** | **2655.4736** | **-0.7005** | **0** | **2** | **0.14** | **1** | **MPCPPECPESLHDLMCQCWR + Oxidation (M); 5 HNE (H C)** |
|  | [2301](http://df3/mascot/cgi/peptide_view.pl?file=../data/20121119/F002569.dat&query=2301&hit=1&index=&px=1) | **789.8034** | **2366.3884** | **2367.1701** | **-0.7817** | **1** | **2** | **0.1** | **1** | **TLKPGTMSPEAFLQEAQVMKK + 2 Deamidation (NQ); 2 Oxidation (M)** |
|  | [2597](http://df3/mascot/cgi/peptide_view.pl?file=../data/20121119/F002569.dat&query=2597&hit=1&index=&px=1) | **874.3243** | **2619.9512** | **2618.0303** | **1.9209** | **0** | **2** | **0.28** | **1** | **MPCPPECPESLHDLMCQCWR + 4 Carbamidomethyl (C); Oxidation (M)** |
|  | [2393](http://df3/mascot/cgi/peptide_view.pl?file=../data/20121119/F002569.dat&query=2393&hit=1&index=&px=1) | **847.9071** | **2540.6995** | **2541.0983** | **-0.3988** | **0** | **2** | **0.14** | **1** | **MPCPPECPESLHDLMCQCWR + 2 Carbamidomethyl (C); HNE (H C)** |
|  | [771](http://df3/mascot/cgi/peptide_view.pl?file=../data/20121119/F002569.dat&query=771&hit=1&index=&px=1) | **516.6567** | **1031.2989** | **1031.5222** | **-0.2233** | **0** | **1** | **0.14** | **1** | **VPYPGMVNR** |
|  | [293](http://df3/mascot/cgi/peptide_view.pl?file=../data/20121119/F002569.dat&query=293&hit=1&index=&px=1) | **412.6647** | **1234.9723** | **1233.6142** | **1.3581** | **0** | **1** | **0.036** | **1** | **WTAPEAALYGR** |
|  | [1942](http://df3/mascot/cgi/peptide_view.pl?file=../data/20121119/F002569.dat&query=1942&hit=1&index=&px=1) | **746.6018** | **1491.1891** | **1489.6394** | **1.5496** | **0** | **1** | **0.11** | **1** | **GAYCLSVSDFDNAK + Deamidation (NQ)** |
|  | [2610](http://df3/mascot/cgi/peptide_view.pl?file=../data/20121119/F002569.dat&query=2610&hit=1&index=&px=1) | **874.4812** | **2620.4218** | **2619.0143** | **1.4075** | **0** | **1** | **0.25** | **1** | **MPCPPECPESLHDLMCQCWR + 4 Carbamidomethyl (C); Deamidation (NQ); Oxidation (M)** |
|  | [1905](http://df3/mascot/cgi/peptide_view.pl?file=../data/20121119/F002569.dat&query=1905&hit=1&index=&px=1) | **742.5693** | **2224.6862** | **2223.0802** | **1.6059** | **0** | **1** | **0.11** | **1** | **TLKPGTMSPEAFLQEAQVMK + 2 Deamidation (NQ); Oxidation (M)** |
|  | [2613](http://df3/mascot/cgi/peptide_view.pl?file=../data/20121119/F002569.dat&query=2613&hit=1&index=&px=1) | **874.5107** | **2620.5104** | **2619.3467** | **1.1637** | **0** | **1** | **0.25** | **1** | **MPCPPECPESLHDLMCQCWR + Deamidation (NQ); 2 Oxidation (M); 4 HNE (H C)** |
|  | [1809](http://df3/mascot/cgi/peptide_view.pl?file=../data/20121119/F002569.dat&query=1809&hit=1&index=&px=1) | **736.1425** | **2205.4055** | **2205.1173** | **0.2882** | **0** | **1** | **0.11** | **1** | **TLKPGTMSPEAFLQEAQVMK** |
|  | [2604](http://df3/mascot/cgi/peptide_view.pl?file=../data/20121119/F002569.dat&query=2604&hit=1&index=&px=1) | **874.4153** | **2620.2240** | **2619.3467** | **0.8773** | **0** | **1** | **0.26** | **1** | **MPCPPECPESLHDLMCQCWR + Deamidation (NQ); 2 Oxidation (M); 4 HNE (H C)** |
|  | [2617](http://df3/mascot/cgi/peptide_view.pl?file=../data/20121119/F002569.dat&query=2617&hit=1&index=&px=1) | **874.6149** | **2620.8228** | **2619.3467** | **1.4761** | **0** | **1** | **0.26** | **1** | **MPCPPECPESLHDLMCQCWR + Deamidation (NQ); 2 Oxidation (M); 4 HNE (H C)** |
|  | [1474](http://df3/mascot/cgi/peptide_view.pl?file=../data/20121119/F002569.dat&query=1474&hit=1&index=&px=1) | **673.5096** | **2017.5071** | **2016.0098** | **1.4973** | **1** | **1** | **0.19** | **1** | **VPYPGMVNREVLDQVER + Oxidation (M)** |
|  | [2605](http://df3/mascot/cgi/peptide_view.pl?file=../data/20121119/F002569.dat&query=2605&hit=1&index=&px=1) | **874.4414** | **2620.3024** | **2618.3698** | **1.9325** | **1** | **1** | **0.34** | **1** | **VAIKTLKPGTMSPEAFLQEAQVMK + 2 Deamidation (NQ)** |
|  | [2653](http://df3/mascot/cgi/peptide_view.pl?file=../data/20121119/F002569.dat&query=2653&hit=1&index=&px=1) | **879.1630** | **2634.4671** | **2634.0252** | **0.4418** | **0** | **1** | **0.19** | **1** | **MPCPPECPESLHDLMCQCWR + 4 Carbamidomethyl (C); 2 Oxidation (M)** |
|  | [2593](http://df3/mascot/cgi/peptide_view.pl?file=../data/20121119/F002569.dat&query=2593&hit=1&index=&px=1) | **874.2722** | **2619.7948** | **2618.3698** | **1.4250** | **1** | **1** | **0.31** | **1** | **VAIKTLKPGTMSPEAFLQEAQVMK + 2 Deamidation (NQ)** |
|  | [2580](http://df3/mascot/cgi/peptide_view.pl?file=../data/20121119/F002569.dat&query=2580&hit=1&index=&px=1) | **874.1558** | **2619.4455** | **2618.0303** | **1.4152** | **0** | **1** | **0.32** | **1** | **MPCPPECPESLHDLMCQCWR + 4 Carbamidomethyl (C); Oxidation (M)** |
|  | [2592](http://df3/mascot/cgi/peptide_view.pl?file=../data/20121119/F002569.dat&query=2592&hit=1&index=&px=1) | **874.2598** | **2619.7575** | **2618.0303** | **1.7272** | **0** | **1** | **0.32** | **1** | **MPCPPECPESLHDLMCQCWR + 4 Carbamidomethyl (C); Oxidation (M)** |
|  | [2550](http://df3/mascot/cgi/peptide_view.pl?file=../data/20121119/F002569.dat&query=2550&hit=1&index=&px=1) | **870.8532** | **2609.5378** | **2611.3137** | **-1.7759** | **1** | **1** | **0.4** | **1** | **YLRLPQLVDMAAQIASGMAYVER + Deamidation (NQ); Oxidation (M)** |
|  | [2611](http://df3/mascot/cgi/peptide_view.pl?file=../data/20121119/F002569.dat&query=2611&hit=1&index=&px=1) | **874.4889** | **2620.4448** | **2619.3467** | **1.0982** | **0** | **1** | **0.28** | **1** | **MPCPPECPESLHDLMCQCWR + Deamidation (NQ); 2 Oxidation (M); 4 HNE (H C)** |
|  | [1807](http://df3/mascot/cgi/peptide_view.pl?file=../data/20121119/F002569.dat&query=1807&hit=1&index=&px=1) | **735.8276** | **2204.4611** | **2206.1013** | **-1.6402** | **0** | **1** | **0.081** | **1** | **TLKPGTMSPEAFLQEAQVMK + Deamidation (NQ)** |
|  | [2619](http://df3/mascot/cgi/peptide_view.pl?file=../data/20121119/F002569.dat&query=2619&hit=1&index=&px=1) | **874.6527** | **2620.9363** | **2619.3467** | **1.5896** | **0** | **1** | **0.29** | **1** | **MPCPPECPESLHDLMCQCWR + Deamidation (NQ); 2 Oxidation (M); 4 HNE (H C)** |
|  | [2644](http://df3/mascot/cgi/peptide_view.pl?file=../data/20121119/F002569.dat&query=2644&hit=1&index=&px=1) | **878.2471** | **2631.7194** | **2630.1096** | **1.6098** | **0** | **1** | **0.33** | **1** | **MPCPPECPESLHDLMCQCWR + 3 Carbamidomethyl (C); 2 Oxidation (M); HNE (H C)** |
|  | [2528](http://df3/mascot/cgi/peptide_view.pl?file=../data/20121119/F002569.dat&query=2528&hit=1&index=&px=1) | **864.0896** | **2589.2470** | **2590.2885** | **-1.0415** | **0** | **1** | **0.46** | **1** | **MPCPPECPESLHDLMCQCWR + Carbamidomethyl (C); 3 HNE (H C)** |
|  | [2576](http://df3/mascot/cgi/peptide_view.pl?file=../data/20121119/F002569.dat&query=2576&hit=1&index=&px=1) | **874.1271** | **2619.3594** | **2619.3467** | **0.0127** | **0** | **1** | **0.38** | **1** | **MPCPPECPESLHDLMCQCWR + Deamidation (NQ); 2 Oxidation (M); 4 HNE (H C)** |
|  | [2565](http://df3/mascot/cgi/peptide_view.pl?file=../data/20121119/F002569.dat&query=2565&hit=1&index=&px=1) | **873.6622** | **2617.9649** | **2619.3467** | **-1.3818** | **0** | **1** | **0.46** | **1** | **MPCPPECPESLHDLMCQCWR + Deamidation (NQ); 2 Oxidation (M); 4 HNE (H C)** |
|  | [2707](http://df3/mascot/cgi/peptide_view.pl?file=../data/20121119/F002569.dat&query=2707&hit=1&index=&px=1) | **886.5083** | **1771.0021** | **1772.9342** | **-1.9322** | **0** | **1** | **0.26** | **1** | **LTTVCPTSKPQTQGLAK + Deamidation (NQ)** |
|  | [2407](http://df3/mascot/cgi/peptide_view.pl?file=../data/20121119/F002569.dat&query=2407&hit=1&index=&px=1) | **853.0932** | **2556.2578** | **2557.0932** | **-0.8354** | **0** | **1** | **0.35** | **1** | **MPCPPECPESLHDLMCQCWR + 2 Carbamidomethyl (C); Oxidation (M); HNE (H C)** |
|  | [2638](http://df3/mascot/cgi/peptide_view.pl?file=../data/20121119/F002569.dat&query=2638&hit=1&index=&px=1) | **877.1451** | **2628.4136** | **2627.1780** | **1.2356** | **0** | **1** | **0.48** | **1** | **MPCPPECPESLHDLMCQCWR + 2 Carbamidomethyl (C); Deamidation (NQ); 2 Oxidation (M); 2 HNE (H C)** |
|  | [848](http://df3/mascot/cgi/peptide_view.pl?file=../data/20121119/F002569.dat&query=848&hit=1&index=&px=1) | **526.1479** | **1050.2813** | **1048.5011** | **1.7802** | **0** | **1** | **0.044** | **1** | **VPYPGMVNR + Deamidation (NQ); Oxidation (M)** |
|  | [2573](http://df3/mascot/cgi/peptide_view.pl?file=../data/20121119/F002569.dat&query=2573&hit=1&index=&px=1) | **874.0869** | **2619.2389** | **2618.0303** | **1.2086** | **0** | **1** | **0.36** | **1** | **MPCPPECPESLHDLMCQCWR + 4 Carbamidomethyl (C); Oxidation (M)** |
|  | [2577](http://df3/mascot/cgi/peptide_view.pl?file=../data/20121119/F002569.dat&query=2577&hit=1&index=&px=1) | **874.1335** | **2619.3788** | **2618.0303** | **1.3485** | **0** | **0** | **0.4** | **1** | **MPCPPECPESLHDLMCQCWR + 4 Carbamidomethyl (C); Oxidation (M)** |
|  | [2801](http://df3/mascot/cgi/peptide_view.pl?file=../data/20121119/F002569.dat&query=2801&hit=1&index=&px=1) | **930.6614** | **2788.9623** | **2788.4296** | **0.5327** | **1** | **0** | **0.4** | **1** | **TQFNSLQQLVAYYSKHADGLCHR + Carbamidomethyl (C); HNE (H C)** |
|  | [1138](http://df3/mascot/cgi/peptide_view.pl?file=../data/20121119/F002569.dat&query=1138&hit=1&index=&px=1) | **592.9326** | **1775.7760** | **1773.9182** | **1.8578** | **0** | **0** | **0.045** | **1** | **LTTVCPTSKPQTQGLAK + 2 Deamidation (NQ)** |
|  | [2585](http://df3/mascot/cgi/peptide_view.pl?file=../data/20121119/F002569.dat&query=2585&hit=1&index=&px=1) | **874.2065** | **2619.5978** | **2618.3698** | **1.2280** | **1** | **0** | **0.36** | **1** | **VAIKTLKPGTMSPEAFLQEAQVMK + 2 Deamidation (NQ)** |
|  | [2622](http://df3/mascot/cgi/peptide_view.pl?file=../data/20121119/F002569.dat&query=2622&hit=1&index=&px=1) | **875.1921** | **2622.5546** | **2623.2623** | **-0.7077** | **0** | **0** | **0.4** | **1** | **MPCPPECPESLHDLMCQCWR + Carbamidomethyl (C); Deamidation (NQ); 2 Oxidation (M); 3 HNE (H C)** |
|  | [2643](http://df3/mascot/cgi/peptide_view.pl?file=../data/20121119/F002569.dat&query=2643&hit=1&index=&px=1) | **877.9926** | **2630.9558** | **2632.3967** | **-1.4409** | **1** | **0** | **0.36** | **1** | **VAIKTLKPGTMSPEAFLQEAQVMK + Oxidation (M)** |
|  | [2787](http://df3/mascot/cgi/peptide_view.pl?file=../data/20121119/F002569.dat&query=2787&hit=1&index=&px=1) | **918.0277** | **2751.0613** | **2750.1354** | **0.9259** | **1** | **0** | **0.27** | **1** | **GYRMPCPPECPESLHDLMCQCWR** |
|  | [1516](http://df3/mascot/cgi/peptide_view.pl?file=../data/20121119/F002569.dat&query=1516&hit=1&index=&px=1) | **685.1899** | **2052.5478** | **2053.0731** | **-0.5253** | **1** | **0** | **0.23** | **1** | **GAYCLSVSDFDNAKGLNVK + HNE (H C)** |
|  | [2584](http://df3/mascot/cgi/peptide_view.pl?file=../data/20121119/F002569.dat&query=2584&hit=1&index=&px=1) | **874.2064** | **2619.5974** | **2618.3698** | **1.2276** | **1** | **0** | **0.36** | **1** | **VAIKTLKPGTMSPEAFLQEAQVMK + 2 Deamidation (NQ)** |
|  | [1098](http://df3/mascot/cgi/peptide_view.pl?file=../data/20121119/F002569.dat&query=1098&hit=1&index=&px=1) | **591.0911** | **1770.2514** | **1771.9502** | **-1.6988** | **0** | **0** | **0.046** | **1** | **LTTVCPTSKPQTQGLAK** |
|  | [1661](http://df3/mascot/cgi/peptide_view.pl?file=../data/20121119/F002569.dat&query=1661&hit=1&index=&px=1) | **726.3496** | **2176.0270** | **2174.1404** | **1.8866** | **1** | **0** | **0.046** | **1** | **AANILVGENLVCKVADFGLAR + 2 Deamidation (NQ)** |
|  | [1929](http://df3/mascot/cgi/peptide_view.pl?file=../data/20121119/F002569.dat&query=1929&hit=1&index=&px=1) | **746.0980** | **2235.2722** | **2237.1071** | **-1.8349** | **0** | **0** | **0.14** | **1** | **TLKPGTMSPEAFLQEAQVMK + 2 Oxidation (M)** |
|  | [1578](http://df3/mascot/cgi/peptide_view.pl?file=../data/20121119/F002569.dat&query=1578&hit=1&index=&px=1) | **701.6951** | **2102.0636** | **2100.9033** | **1.1603** | **0** | **0** | **0.19** | **1** | **LGQGCFGEVWMGTWNGTTR + 2 Deamidation (NQ)** |
|  | [2598](http://df3/mascot/cgi/peptide_view.pl?file=../data/20121119/F002569.dat&query=2598&hit=1&index=&px=1) | **874.3284** | **2619.9633** | **2618.0303** | **1.9330** | **0** | **0** | **0.38** | **1** | **MPCPPECPESLHDLMCQCWR + 4 Carbamidomethyl (C); Oxidation (M)** |
|  | [1322](http://df3/mascot/cgi/peptide_view.pl?file=../data/20121119/F002569.dat&query=1322&hit=1&index=&px=1) | **630.8973** | **1259.7800** | **1260.6397** | **-0.8597** | **1** | **0** | **0.095** | **1** | **GRVPYPGMVNR + Oxidation (M)** |
|  | [2568](http://df3/mascot/cgi/peptide_view.pl?file=../data/20121119/F002569.dat&query=2568&hit=1&index=&px=1) | **873.9069** | **2618.6988** | **2618.0303** | **0.6684** | **0** | **0** | **0.44** | **1** | **MPCPPECPESLHDLMCQCWR + 4 Carbamidomethyl (C); Oxidation (M)** |
|  | [1](http://df3/mascot/cgi/peptide_view.pl?file=../data/20121119/F002569.dat&query=1&hit=1&index=&px=1) | ***361.9645*** | ***721.9144*** |  |  |  |  |  |  |  |
|  | [2](http://df3/mascot/cgi/peptide_view.pl?file=../data/20121119/F002569.dat&query=2&hit=1&index=&px=1) | ***362.3455*** | ***722.6765*** |  |  |  |  |  |  |  |
|  | [3](http://df3/mascot/cgi/peptide_view.pl?file=../data/20121119/F002569.dat&query=3&hit=1&index=&px=1) | ***363.8998*** | ***725.7851*** |  |  |  |  |  |  |  |
|  | [4](http://df3/mascot/cgi/peptide_view.pl?file=../data/20121119/F002569.dat&query=4&hit=1&index=&px=1) | ***369.7718*** | ***737.5290*** |  |  |  |  |  |  |  |
|  | [5](http://df3/mascot/cgi/peptide_view.pl?file=../data/20121119/F002569.dat&query=5&hit=1&index=&px=1) | ***370.7930*** | ***739.5714*** |  |  |  |  |  |  |  |
|  | [6](http://df3/mascot/cgi/peptide_view.pl?file=../data/20121119/F002569.dat&query=6&hit=1&index=&px=1) | ***370.8833*** | ***739.7521*** |  |  |  |  |  |  |  |
|  | [7](http://df3/mascot/cgi/peptide_view.pl?file=../data/20121119/F002569.dat&query=7&hit=1&index=&px=1) | ***370.8940*** | ***739.7734*** |  |  |  |  |  |  |  |
|  | [8](http://df3/mascot/cgi/peptide_view.pl?file=../data/20121119/F002569.dat&query=8&hit=1&index=&px=1) | ***370.9034*** | ***739.7922*** |  |  |  |  |  |  |  |
|  | [9](http://df3/mascot/cgi/peptide_view.pl?file=../data/20121119/F002569.dat&query=9&hit=1&index=&px=1) | ***370.9092*** | ***739.8039*** |  |  |  |  |  |  |  |
|  | [10](http://df3/mascot/cgi/peptide_view.pl?file=../data/20121119/F002569.dat&query=10&hit=1&index=&px=1) | ***370.9444*** | ***739.8742*** |  |  |  |  |  |  |  |
|  | [11](http://df3/mascot/cgi/peptide_view.pl?file=../data/20121119/F002569.dat&query=11&hit=1&index=&px=1) | ***370.9861*** | ***739.9576*** |  |  |  |  |  |  |  |
|  | [12](http://df3/mascot/cgi/peptide_view.pl?file=../data/20121119/F002569.dat&query=12&hit=1&index=&px=1) | ***370.9884*** | ***739.9623*** |  |  |  |  |  |  |  |
|  | [13](http://df3/mascot/cgi/peptide_view.pl?file=../data/20121119/F002569.dat&query=13&hit=1&index=&px=1) | ***370.9993*** | ***739.9840*** |  |  |  |  |  |  |  |
|  | [14](http://df3/mascot/cgi/peptide_view.pl?file=../data/20121119/F002569.dat&query=14&hit=1&index=&px=1) | ***370.9999*** | ***739.9853*** |  |  |  |  |  |  |  |
|  | [15](http://df3/mascot/cgi/peptide_view.pl?file=../data/20121119/F002569.dat&query=15&hit=1&index=&px=1) | ***371.0029*** | ***739.9913*** |  |  |  |  |  |  |  |
|  | [16](http://df3/mascot/cgi/peptide_view.pl?file=../data/20121119/F002569.dat&query=16&hit=1&index=&px=1) | ***371.0071*** | ***739.9997*** |  |  |  |  |  |  |  |
|  | [17](http://df3/mascot/cgi/peptide_view.pl?file=../data/20121119/F002569.dat&query=17&hit=1&index=&px=1) | ***371.0079*** | ***740.0013*** |  |  |  |  |  |  |  |
|  | [18](http://df3/mascot/cgi/peptide_view.pl?file=../data/20121119/F002569.dat&query=18&hit=1&index=&px=1) | ***371.0081*** | ***740.0016*** |  |  |  |  |  |  |  |
|  | [19](http://df3/mascot/cgi/peptide_view.pl?file=../data/20121119/F002569.dat&query=19&hit=1&index=&px=1) | ***371.0093*** | ***740.0040*** |  |  |  |  |  |  |  |
|  | [20](http://df3/mascot/cgi/peptide_view.pl?file=../data/20121119/F002569.dat&query=20&hit=1&index=&px=1) | ***371.0414*** | ***740.0682*** |  |  |  |  |  |  |  |
|  | [21](http://df3/mascot/cgi/peptide_view.pl?file=../data/20121119/F002569.dat&query=21&hit=1&index=&px=1) | ***371.0591*** | ***740.1037*** |  |  |  |  |  |  |  |
|  | [22](http://df3/mascot/cgi/peptide_view.pl?file=../data/20121119/F002569.dat&query=22&hit=1&index=&px=1) | ***371.0641*** | ***740.1137*** |  |  |  |  |  |  |  |
|  | [23](http://df3/mascot/cgi/peptide_view.pl?file=../data/20121119/F002569.dat&query=23&hit=1&index=&px=1) | ***371.0956*** | ***740.1766*** |  |  |  |  |  |  |  |
|  | [24](http://df3/mascot/cgi/peptide_view.pl?file=../data/20121119/F002569.dat&query=24&hit=1&index=&px=1) | ***371.0970*** | ***740.1794*** |  |  |  |  |  |  |  |
|  | [25](http://df3/mascot/cgi/peptide_view.pl?file=../data/20121119/F002569.dat&query=25&hit=1&index=&px=1) | ***371.1022*** | ***740.1899*** |  |  |  |  |  |  |  |
|  | [26](http://df3/mascot/cgi/peptide_view.pl?file=../data/20121119/F002569.dat&query=26&hit=1&index=&px=1) | ***371.1047*** | ***740.1949*** |  |  |  |  |  |  |  |
|  | [27](http://df3/mascot/cgi/peptide_view.pl?file=../data/20121119/F002569.dat&query=27&hit=1&index=&px=1) | ***371.1085*** | ***740.2024*** |  |  |  |  |  |  |  |
|  | [28](http://df3/mascot/cgi/peptide_view.pl?file=../data/20121119/F002569.dat&query=28&hit=1&index=&px=1) | ***371.1353*** | ***740.2560*** |  |  |  |  |  |  |  |
|  | [29](http://df3/mascot/cgi/peptide_view.pl?file=../data/20121119/F002569.dat&query=29&hit=1&index=&px=1) | ***371.1435*** | ***740.2724*** |  |  |  |  |  |  |  |
|  | [30](http://df3/mascot/cgi/peptide_view.pl?file=../data/20121119/F002569.dat&query=30&hit=1&index=&px=1) | ***371.1592*** | ***740.3039*** |  |  |  |  |  |  |  |
|  | [31](http://df3/mascot/cgi/peptide_view.pl?file=../data/20121119/F002569.dat&query=31&hit=1&index=&px=1) | ***371.1896*** | ***740.3647*** |  |  |  |  |  |  |  |
|  | [32](http://df3/mascot/cgi/peptide_view.pl?file=../data/20121119/F002569.dat&query=32&hit=1&index=&px=1) | ***371.2290*** | ***740.4435*** |  |  |  |  |  |  |  |
|  | [33](http://df3/mascot/cgi/peptide_view.pl?file=../data/20121119/F002569.dat&query=33&hit=1&index=&px=1) | ***371.5610*** | ***741.1075*** |  |  |  |  |  |  |  |
|  | [34](http://df3/mascot/cgi/peptide_view.pl?file=../data/20121119/F002569.dat&query=34&hit=1&index=&px=1) | ***371.5832*** | ***741.1518*** |  |  |  |  |  |  |  |
|  | [35](http://df3/mascot/cgi/peptide_view.pl?file=../data/20121119/F002569.dat&query=35&hit=1&index=&px=1) | ***372.3692*** | ***742.7238*** |  |  |  |  |  |  |  |
|  | [36](http://df3/mascot/cgi/peptide_view.pl?file=../data/20121119/F002569.dat&query=36&hit=1&index=&px=1) | ***372.4398*** | ***742.8650*** |  |  |  |  |  |  |  |
|  | [37](http://df3/mascot/cgi/peptide_view.pl?file=../data/20121119/F002569.dat&query=37&hit=1&index=&px=1) | ***372.4404*** | ***742.8662*** |  |  |  |  |  |  |  |
|  | [38](http://df3/mascot/cgi/peptide_view.pl?file=../data/20121119/F002569.dat&query=38&hit=1&index=&px=1) | ***372.7160*** | ***743.4175*** |  |  |  |  |  |  |  |
|  | [39](http://df3/mascot/cgi/peptide_view.pl?file=../data/20121119/F002569.dat&query=39&hit=1&index=&px=1) | ***372.8658*** | ***743.7171*** |  |  |  |  |  |  |  |
|  | [40](http://df3/mascot/cgi/peptide_view.pl?file=../data/20121119/F002569.dat&query=40&hit=1&index=&px=1) | ***373.0140*** | ***744.0134*** |  |  |  |  |  |  |  |
|  | [41](http://df3/mascot/cgi/peptide_view.pl?file=../data/20121119/F002569.dat&query=41&hit=1&index=&px=1) | ***373.1537*** | ***744.2928*** |  |  |  |  |  |  |  |
|  | [42](http://df3/mascot/cgi/peptide_view.pl?file=../data/20121119/F002569.dat&query=42&hit=1&index=&px=1) | ***373.1758*** | ***744.3371*** |  |  |  |  |  |  |  |
|  | [43](http://df3/mascot/cgi/peptide_view.pl?file=../data/20121119/F002569.dat&query=43&hit=1&index=&px=1) | ***373.2341*** | ***744.4536*** |  |  |  |  |  |  |  |
|  | [44](http://df3/mascot/cgi/peptide_view.pl?file=../data/20121119/F002569.dat&query=44&hit=1&index=&px=1) | ***374.2265*** | ***746.4384*** |  |  |  |  |  |  |  |
|  | [45](http://df3/mascot/cgi/peptide_view.pl?file=../data/20121119/F002569.dat&query=45&hit=1&index=&px=1) | ***374.2942*** | ***746.5739*** |  |  |  |  |  |  |  |
|  | [46](http://df3/mascot/cgi/peptide_view.pl?file=../data/20121119/F002569.dat&query=46&hit=1&index=&px=1) | ***379.2119*** | ***756.4092*** |  |  |  |  |  |  |  |
|  | [47](http://df3/mascot/cgi/peptide_view.pl?file=../data/20121119/F002569.dat&query=47&hit=1&index=&px=1) | ***381.7942*** | ***761.5739*** |  |  |  |  |  |  |  |
|  | [48](http://df3/mascot/cgi/peptide_view.pl?file=../data/20121119/F002569.dat&query=48&hit=1&index=&px=1) | ***384.1966*** | ***766.3786*** |  |  |  |  |  |  |  |
|  | [49](http://df3/mascot/cgi/peptide_view.pl?file=../data/20121119/F002569.dat&query=49&hit=1&index=&px=1) | ***384.2554*** | ***766.4962*** |  |  |  |  |  |  |  |
|  | [50](http://df3/mascot/cgi/peptide_view.pl?file=../data/20121119/F002569.dat&query=50&hit=1&index=&px=1) | ***386.0913*** | ***770.1681*** |  |  |  |  |  |  |  |
|  | [51](http://df3/mascot/cgi/peptide_view.pl?file=../data/20121119/F002569.dat&query=51&hit=1&index=&px=1) | ***386.9207*** | ***771.8268*** |  |  |  |  |  |  |  |
|  | [52](http://df3/mascot/cgi/peptide_view.pl?file=../data/20121119/F002569.dat&query=52&hit=1&index=&px=1) | ***386.9998*** | ***771.9851*** |  |  |  |  |  |  |  |
|  | [53](http://df3/mascot/cgi/peptide_view.pl?file=../data/20121119/F002569.dat&query=53&hit=1&index=&px=1) | ***387.1586*** | ***772.3027*** |  |  |  |  |  |  |  |
|  | [54](http://df3/mascot/cgi/peptide_view.pl?file=../data/20121119/F002569.dat&query=54&hit=1&index=&px=1) | ***387.2419*** | ***772.4693*** |  |  |  |  |  |  |  |
|  | [55](http://df3/mascot/cgi/peptide_view.pl?file=../data/20121119/F002569.dat&query=55&hit=1&index=&px=1) | ***387.2847*** | ***772.5549*** |  |  |  |  |  |  |  |
|  | [56](http://df3/mascot/cgi/peptide_view.pl?file=../data/20121119/F002569.dat&query=56&hit=1&index=&px=1) | ***387.3384*** | ***772.6623*** |  |  |  |  |  |  |  |
|  | [57](http://df3/mascot/cgi/peptide_view.pl?file=../data/20121119/F002569.dat&query=57&hit=1&index=&px=1) | ***387.3499*** | ***772.6853*** |  |  |  |  |  |  |  |
|  | [58](http://df3/mascot/cgi/peptide_view.pl?file=../data/20121119/F002569.dat&query=58&hit=1&index=&px=1) | ***387.3813*** | ***772.7480*** |  |  |  |  |  |  |  |
|  | [59](http://df3/mascot/cgi/peptide_view.pl?file=../data/20121119/F002569.dat&query=59&hit=1&index=&px=1) | ***387.4005*** | ***772.7865*** |  |  |  |  |  |  |  |
|  | [60](http://df3/mascot/cgi/peptide_view.pl?file=../data/20121119/F002569.dat&query=60&hit=1&index=&px=1) | ***387.4130*** | ***772.8115*** |  |  |  |  |  |  |  |
|  | [61](http://df3/mascot/cgi/peptide_view.pl?file=../data/20121119/F002569.dat&query=61&hit=1&index=&px=1) | ***387.4167*** | ***772.8189*** |  |  |  |  |  |  |  |
|  | [62](http://df3/mascot/cgi/peptide_view.pl?file=../data/20121119/F002569.dat&query=62&hit=1&index=&px=1) | ***387.4190*** | ***772.8235*** |  |  |  |  |  |  |  |
|  | [63](http://df3/mascot/cgi/peptide_view.pl?file=../data/20121119/F002569.dat&query=63&hit=1&index=&px=1) | ***387.4420*** | ***772.8694*** |  |  |  |  |  |  |  |
|  | [64](http://df3/mascot/cgi/peptide_view.pl?file=../data/20121119/F002569.dat&query=64&hit=1&index=&px=1) | ***387.4453*** | ***772.8761*** |  |  |  |  |  |  |  |
|  | [65](http://df3/mascot/cgi/peptide_view.pl?file=../data/20121119/F002569.dat&query=65&hit=1&index=&px=1) | ***387.4622*** | ***772.9099*** |  |  |  |  |  |  |  |
|  | [66](http://df3/mascot/cgi/peptide_view.pl?file=../data/20121119/F002569.dat&query=66&hit=1&index=&px=1) | ***387.4949*** | ***772.9752*** |  |  |  |  |  |  |  |
|  | [67](http://df3/mascot/cgi/peptide_view.pl?file=../data/20121119/F002569.dat&query=67&hit=1&index=&px=1) | ***387.5088*** | ***773.0030*** |  |  |  |  |  |  |  |
|  | [68](http://df3/mascot/cgi/peptide_view.pl?file=../data/20121119/F002569.dat&query=68&hit=1&index=&px=1) | ***387.5746*** | ***773.1347*** |  |  |  |  |  |  |  |
|  | [69](http://df3/mascot/cgi/peptide_view.pl?file=../data/20121119/F002569.dat&query=69&hit=1&index=&px=1) | ***387.5804*** | ***773.1462*** |  |  |  |  |  |  |  |
|  | [70](http://df3/mascot/cgi/peptide_view.pl?file=../data/20121119/F002569.dat&query=70&hit=1&index=&px=1) | ***387.5872*** | ***773.1599*** |  |  |  |  |  |  |  |
|  | [71](http://df3/mascot/cgi/peptide_view.pl?file=../data/20121119/F002569.dat&query=71&hit=1&index=&px=1) | ***387.6553*** | ***773.2960*** |  |  |  |  |  |  |  |
|  | [72](http://df3/mascot/cgi/peptide_view.pl?file=../data/20121119/F002569.dat&query=72&hit=1&index=&px=1) | ***387.6639*** | ***773.3132*** |  |  |  |  |  |  |  |
|  | [73](http://df3/mascot/cgi/peptide_view.pl?file=../data/20121119/F002569.dat&query=73&hit=1&index=&px=1) | ***387.7060*** | ***773.3974*** |  |  |  |  |  |  |  |
|  | [74](http://df3/mascot/cgi/peptide_view.pl?file=../data/20121119/F002569.dat&query=74&hit=1&index=&px=1) | ***387.7099*** | ***773.4053*** |  |  |  |  |  |  |  |
|  | [75](http://df3/mascot/cgi/peptide_view.pl?file=../data/20121119/F002569.dat&query=75&hit=1&index=&px=1) | ***387.7249*** | ***773.4353*** |  |  |  |  |  |  |  |
|  | [76](http://df3/mascot/cgi/peptide_view.pl?file=../data/20121119/F002569.dat&query=76&hit=1&index=&px=1) | ***387.7264*** | ***773.4383*** |  |  |  |  |  |  |  |
|  | [77](http://df3/mascot/cgi/peptide_view.pl?file=../data/20121119/F002569.dat&query=77&hit=1&index=&px=1) | ***387.7372*** | ***773.4599*** |  |  |  |  |  |  |  |
|  | [78](http://df3/mascot/cgi/peptide_view.pl?file=../data/20121119/F002569.dat&query=78&hit=1&index=&px=1) | ***387.8065*** | ***773.5984*** |  |  |  |  |  |  |  |
|  | [79](http://df3/mascot/cgi/peptide_view.pl?file=../data/20121119/F002569.dat&query=79&hit=1&index=&px=1) | ***387.8197*** | ***773.6249*** |  |  |  |  |  |  |  |
|  | [80](http://df3/mascot/cgi/peptide_view.pl?file=../data/20121119/F002569.dat&query=80&hit=1&index=&px=1) | ***387.8252*** | ***773.6359*** |  |  |  |  |  |  |  |
|  | [81](http://df3/mascot/cgi/peptide_view.pl?file=../data/20121119/F002569.dat&query=81&hit=1&index=&px=1) | ***387.8472*** | ***773.6798*** |  |  |  |  |  |  |  |
|  | [82](http://df3/mascot/cgi/peptide_view.pl?file=../data/20121119/F002569.dat&query=82&hit=1&index=&px=1) | ***387.9686*** | ***773.9226*** |  |  |  |  |  |  |  |
|  | [83](http://df3/mascot/cgi/peptide_view.pl?file=../data/20121119/F002569.dat&query=83&hit=1&index=&px=1) | ***388.0368*** | ***774.0590*** |  |  |  |  |  |  |  |
|  | [84](http://df3/mascot/cgi/peptide_view.pl?file=../data/20121119/F002569.dat&query=84&hit=1&index=&px=1) | ***388.0472*** | ***774.0799*** |  |  |  |  |  |  |  |
|  | [85](http://df3/mascot/cgi/peptide_view.pl?file=../data/20121119/F002569.dat&query=85&hit=1&index=&px=1) | ***388.0680*** | ***774.1214*** |  |  |  |  |  |  |  |
|  | [86](http://df3/mascot/cgi/peptide_view.pl?file=../data/20121119/F002569.dat&query=86&hit=1&index=&px=1) | ***388.1579*** | ***774.3012*** |  |  |  |  |  |  |  |
|  | [87](http://df3/mascot/cgi/peptide_view.pl?file=../data/20121119/F002569.dat&query=87&hit=1&index=&px=1) | ***388.1692*** | ***774.3238*** |  |  |  |  |  |  |  |
|  | [88](http://df3/mascot/cgi/peptide_view.pl?file=../data/20121119/F002569.dat&query=88&hit=1&index=&px=1) | ***388.1810*** | ***774.3474*** |  |  |  |  |  |  |  |
|  | [89](http://df3/mascot/cgi/peptide_view.pl?file=../data/20121119/F002569.dat&query=89&hit=1&index=&px=1) | ***388.1874*** | ***774.3603*** |  |  |  |  |  |  |  |
|  | [90](http://df3/mascot/cgi/peptide_view.pl?file=../data/20121119/F002569.dat&query=90&hit=1&index=&px=1) | ***388.4924*** | ***774.9703*** |  |  |  |  |  |  |  |
|  | [91](http://df3/mascot/cgi/peptide_view.pl?file=../data/20121119/F002569.dat&query=91&hit=1&index=&px=1) | ***388.4955*** | ***774.9765*** |  |  |  |  |  |  |  |
|  | [92](http://df3/mascot/cgi/peptide_view.pl?file=../data/20121119/F002569.dat&query=92&hit=1&index=&px=1) | ***388.5621*** | ***775.1097*** |  |  |  |  |  |  |  |
|  | [93](http://df3/mascot/cgi/peptide_view.pl?file=../data/20121119/F002569.dat&query=93&hit=1&index=&px=1) | ***388.5751*** | ***775.1356*** |  |  |  |  |  |  |  |
|  | [94](http://df3/mascot/cgi/peptide_view.pl?file=../data/20121119/F002569.dat&query=94&hit=1&index=&px=1) | ***388.5865*** | ***775.1585*** |  |  |  |  |  |  |  |
|  | [95](http://df3/mascot/cgi/peptide_view.pl?file=../data/20121119/F002569.dat&query=95&hit=1&index=&px=1) | ***388.6017*** | ***775.1888*** |  |  |  |  |  |  |  |
|  | [96](http://df3/mascot/cgi/peptide_view.pl?file=../data/20121119/F002569.dat&query=96&hit=1&index=&px=1) | ***388.6143*** | ***775.2140*** |  |  |  |  |  |  |  |
|  | [97](http://df3/mascot/cgi/peptide_view.pl?file=../data/20121119/F002569.dat&query=97&hit=1&index=&px=1) | ***388.6261*** | ***775.2376*** |  |  |  |  |  |  |  |
|  | [98](http://df3/mascot/cgi/peptide_view.pl?file=../data/20121119/F002569.dat&query=98&hit=1&index=&px=1) | ***388.6435*** | ***775.2724*** |  |  |  |  |  |  |  |
|  | [99](http://df3/mascot/cgi/peptide_view.pl?file=../data/20121119/F002569.dat&query=99&hit=1&index=&px=1) | ***388.6537*** | ***775.2928*** |  |  |  |  |  |  |  |
|  | [100](http://df3/mascot/cgi/peptide_view.pl?file=../data/20121119/F002569.dat&query=100&hit=1&index=&px=1) | ***388.6545*** | ***775.2945*** |  |  |  |  |  |  |  |
|  | [101](http://df3/mascot/cgi/peptide_view.pl?file=../data/20121119/F002569.dat&query=101&hit=1&index=&px=1) | ***388.6696*** | ***775.3246*** |  |  |  |  |  |  |  |
|  | [102](http://df3/mascot/cgi/peptide_view.pl?file=../data/20121119/F002569.dat&query=102&hit=1&index=&px=1) | ***388.6905*** | ***775.3665*** |  |  |  |  |  |  |  |
|  | [103](http://df3/mascot/cgi/peptide_view.pl?file=../data/20121119/F002569.dat&query=103&hit=1&index=&px=1) | ***388.7268*** | ***775.4391*** |  |  |  |  |  |  |  |
|  | [104](http://df3/mascot/cgi/peptide_view.pl?file=../data/20121119/F002569.dat&query=104&hit=1&index=&px=1) | ***388.7530*** | ***775.4914*** |  |  |  |  |  |  |  |
|  | [105](http://df3/mascot/cgi/peptide_view.pl?file=../data/20121119/F002569.dat&query=105&hit=1&index=&px=1) | ***389.2287*** | ***776.4428*** |  |  |  |  |  |  |  |
|  | [106](http://df3/mascot/cgi/peptide_view.pl?file=../data/20121119/F002569.dat&query=106&hit=1&index=&px=1) | ***389.2573*** | ***776.5000*** |  |  |  |  |  |  |  |
|  | [107](http://df3/mascot/cgi/peptide_view.pl?file=../data/20121119/F002569.dat&query=107&hit=1&index=&px=1) | ***389.3471*** | ***776.6796*** |  |  |  |  |  |  |  |
|  | [108](http://df3/mascot/cgi/peptide_view.pl?file=../data/20121119/F002569.dat&query=108&hit=1&index=&px=1) | ***389.4103*** | ***776.8061*** |  |  |  |  |  |  |  |
|  | [109](http://df3/mascot/cgi/peptide_view.pl?file=../data/20121119/F002569.dat&query=109&hit=1&index=&px=1) | ***389.5350*** | ***777.0554*** |  |  |  |  |  |  |  |
|  | [110](http://df3/mascot/cgi/peptide_view.pl?file=../data/20121119/F002569.dat&query=110&hit=1&index=&px=1) | ***389.5789*** | ***777.1433*** |  |  |  |  |  |  |  |
|  | [111](http://df3/mascot/cgi/peptide_view.pl?file=../data/20121119/F002569.dat&query=111&hit=1&index=&px=1) | ***389.5945*** | ***777.1745*** |  |  |  |  |  |  |  |
|  | [112](http://df3/mascot/cgi/peptide_view.pl?file=../data/20121119/F002569.dat&query=112&hit=1&index=&px=1) | ***389.6240*** | ***777.2335*** |  |  |  |  |  |  |  |
|  | [113](http://df3/mascot/cgi/peptide_view.pl?file=../data/20121119/F002569.dat&query=113&hit=1&index=&px=1) | ***389.6581*** | ***777.3017*** |  |  |  |  |  |  |  |
|  | [114](http://df3/mascot/cgi/peptide_view.pl?file=../data/20121119/F002569.dat&query=114&hit=1&index=&px=1) | ***389.6855*** | ***777.3564*** |  |  |  |  |  |  |  |
|  | [115](http://df3/mascot/cgi/peptide_view.pl?file=../data/20121119/F002569.dat&query=115&hit=1&index=&px=1) | ***389.6971*** | ***777.3796*** |  |  |  |  |  |  |  |
|  | [116](http://df3/mascot/cgi/peptide_view.pl?file=../data/20121119/F002569.dat&query=116&hit=1&index=&px=1) | ***389.7334*** | ***777.4522*** |  |  |  |  |  |  |  |
|  | [117](http://df3/mascot/cgi/peptide_view.pl?file=../data/20121119/F002569.dat&query=117&hit=1&index=&px=1) | ***389.7530*** | ***777.4915*** |  |  |  |  |  |  |  |
|  | [118](http://df3/mascot/cgi/peptide_view.pl?file=../data/20121119/F002569.dat&query=118&hit=1&index=&px=1) | ***389.7621*** | ***777.5096*** |  |  |  |  |  |  |  |
|  | [119](http://df3/mascot/cgi/peptide_view.pl?file=../data/20121119/F002569.dat&query=119&hit=1&index=&px=1) | ***389.7677*** | ***777.5208*** |  |  |  |  |  |  |  |
|  | [120](http://df3/mascot/cgi/peptide_view.pl?file=../data/20121119/F002569.dat&query=120&hit=1&index=&px=1) | ***389.8202*** | ***777.6259*** |  |  |  |  |  |  |  |
|  | [121](http://df3/mascot/cgi/peptide_view.pl?file=../data/20121119/F002569.dat&query=121&hit=1&index=&px=1) | ***389.8203*** | ***777.6261*** |  |  |  |  |  |  |  |
|  | [122](http://df3/mascot/cgi/peptide_view.pl?file=../data/20121119/F002569.dat&query=122&hit=1&index=&px=1) | ***389.8546*** | ***777.6947*** |  |  |  |  |  |  |  |
|  | [123](http://df3/mascot/cgi/peptide_view.pl?file=../data/20121119/F002569.dat&query=123&hit=1&index=&px=1) | ***389.9578*** | ***777.9011*** |  |  |  |  |  |  |  |
|  | [124](http://df3/mascot/cgi/peptide_view.pl?file=../data/20121119/F002569.dat&query=124&hit=1&index=&px=1) | ***389.9917*** | ***777.9688*** |  |  |  |  |  |  |  |
|  | [125](http://df3/mascot/cgi/peptide_view.pl?file=../data/20121119/F002569.dat&query=125&hit=1&index=&px=1) | ***390.0052*** | ***777.9958*** |  |  |  |  |  |  |  |
|  | [126](http://df3/mascot/cgi/peptide_view.pl?file=../data/20121119/F002569.dat&query=126&hit=1&index=&px=1) | ***390.0131*** | ***778.0116*** |  |  |  |  |  |  |  |
|  | [127](http://df3/mascot/cgi/peptide_view.pl?file=../data/20121119/F002569.dat&query=127&hit=1&index=&px=1) | ***390.0257*** | ***778.0368*** |  |  |  |  |  |  |  |
|  | [128](http://df3/mascot/cgi/peptide_view.pl?file=../data/20121119/F002569.dat&query=128&hit=1&index=&px=1) | ***390.0392*** | ***778.0639*** |  |  |  |  |  |  |  |
|  | [129](http://df3/mascot/cgi/peptide_view.pl?file=../data/20121119/F002569.dat&query=129&hit=1&index=&px=1) | ***390.0441*** | ***778.0737*** |  |  |  |  |  |  |  |
|  | [130](http://df3/mascot/cgi/peptide_view.pl?file=../data/20121119/F002569.dat&query=130&hit=1&index=&px=1) | ***390.0645*** | ***778.1145*** |  |  |  |  |  |  |  |
|  | [131](http://df3/mascot/cgi/peptide_view.pl?file=../data/20121119/F002569.dat&query=131&hit=1&index=&px=1) | ***390.0813*** | ***778.1480*** |  |  |  |  |  |  |  |
|  | [132](http://df3/mascot/cgi/peptide_view.pl?file=../data/20121119/F002569.dat&query=132&hit=1&index=&px=1) | ***390.0847*** | ***778.1549*** |  |  |  |  |  |  |  |
|  | [133](http://df3/mascot/cgi/peptide_view.pl?file=../data/20121119/F002569.dat&query=133&hit=1&index=&px=1) | ***390.0883*** | ***778.1620*** |  |  |  |  |  |  |  |
|  | [134](http://df3/mascot/cgi/peptide_view.pl?file=../data/20121119/F002569.dat&query=134&hit=1&index=&px=1) | ***390.0922*** | ***778.1699*** |  |  |  |  |  |  |  |
|  | [135](http://df3/mascot/cgi/peptide_view.pl?file=../data/20121119/F002569.dat&query=135&hit=1&index=&px=1) | ***390.0966*** | ***778.1786*** |  |  |  |  |  |  |  |
|  | [136](http://df3/mascot/cgi/peptide_view.pl?file=../data/20121119/F002569.dat&query=136&hit=1&index=&px=1) | ***390.1031*** | ***778.1917*** |  |  |  |  |  |  |  |
|  | [137](http://df3/mascot/cgi/peptide_view.pl?file=../data/20121119/F002569.dat&query=137&hit=1&index=&px=1) | ***390.1218*** | ***778.2290*** |  |  |  |  |  |  |  |
|  | [138](http://df3/mascot/cgi/peptide_view.pl?file=../data/20121119/F002569.dat&query=138&hit=1&index=&px=1) | ***390.1442*** | ***778.2738*** |  |  |  |  |  |  |  |
|  | [139](http://df3/mascot/cgi/peptide_view.pl?file=../data/20121119/F002569.dat&query=139&hit=1&index=&px=1) | ***390.1515*** | ***778.2885*** |  |  |  |  |  |  |  |
|  | [140](http://df3/mascot/cgi/peptide_view.pl?file=../data/20121119/F002569.dat&query=140&hit=1&index=&px=1) | ***390.1593*** | ***778.3041*** |  |  |  |  |  |  |  |
|  | [141](http://df3/mascot/cgi/peptide_view.pl?file=../data/20121119/F002569.dat&query=141&hit=1&index=&px=1) | ***390.1756*** | ***778.3367*** |  |  |  |  |  |  |  |
|  | [142](http://df3/mascot/cgi/peptide_view.pl?file=../data/20121119/F002569.dat&query=142&hit=1&index=&px=1) | ***390.1856*** | ***778.3567*** |  |  |  |  |  |  |  |
|  | [143](http://df3/mascot/cgi/peptide_view.pl?file=../data/20121119/F002569.dat&query=143&hit=1&index=&px=1) | ***390.1937*** | ***778.3729*** |  |  |  |  |  |  |  |
|  | [144](http://df3/mascot/cgi/peptide_view.pl?file=../data/20121119/F002569.dat&query=144&hit=1&index=&px=1) | ***390.2140*** | ***778.4135*** |  |  |  |  |  |  |  |
|  | [145](http://df3/mascot/cgi/peptide_view.pl?file=../data/20121119/F002569.dat&query=145&hit=1&index=&px=1) | ***390.2295*** | ***778.4444*** |  |  |  |  |  |  |  |
|  | [146](http://df3/mascot/cgi/peptide_view.pl?file=../data/20121119/F002569.dat&query=146&hit=1&index=&px=1) | ***390.2391*** | ***778.4637*** |  |  |  |  |  |  |  |
|  | [147](http://df3/mascot/cgi/peptide_view.pl?file=../data/20121119/F002569.dat&query=147&hit=1&index=&px=1) | ***390.2849*** | ***778.5552*** |  |  |  |  |  |  |  |
|  | [148](http://df3/mascot/cgi/peptide_view.pl?file=../data/20121119/F002569.dat&query=148&hit=1&index=&px=1) | ***390.2885*** | ***778.5624*** |  |  |  |  |  |  |  |
|  | [149](http://df3/mascot/cgi/peptide_view.pl?file=../data/20121119/F002569.dat&query=149&hit=1&index=&px=1) | ***390.3368*** | ***778.6590*** |  |  |  |  |  |  |  |
|  | [150](http://df3/mascot/cgi/peptide_view.pl?file=../data/20121119/F002569.dat&query=150&hit=1&index=&px=1) | ***390.5334*** | ***779.0523*** |  |  |  |  |  |  |  |
|  | [151](http://df3/mascot/cgi/peptide_view.pl?file=../data/20121119/F002569.dat&query=151&hit=1&index=&px=1) | ***390.8477*** | ***779.6809*** |  |  |  |  |  |  |  |
|  | [152](http://df3/mascot/cgi/peptide_view.pl?file=../data/20121119/F002569.dat&query=152&hit=1&index=&px=1) | ***390.8711*** | ***779.7276*** |  |  |  |  |  |  |  |
|  | [153](http://df3/mascot/cgi/peptide_view.pl?file=../data/20121119/F002569.dat&query=153&hit=1&index=&px=1) | ***390.9011*** | ***779.7876*** |  |  |  |  |  |  |  |
|  | [154](http://df3/mascot/cgi/peptide_view.pl?file=../data/20121119/F002569.dat&query=154&hit=1&index=&px=1) | ***390.9061*** | ***779.7977*** |  |  |  |  |  |  |  |
|  | [155](http://df3/mascot/cgi/peptide_view.pl?file=../data/20121119/F002569.dat&query=155&hit=1&index=&px=1) | ***390.9380*** | ***779.8615*** |  |  |  |  |  |  |  |
|  | [156](http://df3/mascot/cgi/peptide_view.pl?file=../data/20121119/F002569.dat&query=156&hit=1&index=&px=1) | ***390.9389*** | ***779.8633*** |  |  |  |  |  |  |  |
|  | [157](http://df3/mascot/cgi/peptide_view.pl?file=../data/20121119/F002569.dat&query=157&hit=1&index=&px=1) | ***390.9580*** | ***779.9014*** |  |  |  |  |  |  |  |
|  | [158](http://df3/mascot/cgi/peptide_view.pl?file=../data/20121119/F002569.dat&query=158&hit=1&index=&px=1) | ***390.9604*** | ***779.9063*** |  |  |  |  |  |  |  |
|  | [159](http://df3/mascot/cgi/peptide_view.pl?file=../data/20121119/F002569.dat&query=159&hit=1&index=&px=1) | ***390.9816*** | ***779.9487*** |  |  |  |  |  |  |  |
|  | [160](http://df3/mascot/cgi/peptide_view.pl?file=../data/20121119/F002569.dat&query=160&hit=1&index=&px=1) | ***390.9869*** | ***779.9592*** |  |  |  |  |  |  |  |
|  | [161](http://df3/mascot/cgi/peptide_view.pl?file=../data/20121119/F002569.dat&query=161&hit=1&index=&px=1) | ***391.0055*** | ***779.9964*** |  |  |  |  |  |  |  |
|  | [162](http://df3/mascot/cgi/peptide_view.pl?file=../data/20121119/F002569.dat&query=162&hit=1&index=&px=1) | ***391.0059*** | ***779.9973*** |  |  |  |  |  |  |  |
|  | [163](http://df3/mascot/cgi/peptide_view.pl?file=../data/20121119/F002569.dat&query=163&hit=1&index=&px=1) | ***391.0101*** | ***780.0057*** |  |  |  |  |  |  |  |
|  | [164](http://df3/mascot/cgi/peptide_view.pl?file=../data/20121119/F002569.dat&query=164&hit=1&index=&px=1) | ***391.0154*** | ***780.0162*** |  |  |  |  |  |  |  |
|  | [165](http://df3/mascot/cgi/peptide_view.pl?file=../data/20121119/F002569.dat&query=165&hit=1&index=&px=1) | ***391.0157*** | ***780.0169*** |  |  |  |  |  |  |  |
|  | [166](http://df3/mascot/cgi/peptide_view.pl?file=../data/20121119/F002569.dat&query=166&hit=1&index=&px=1) | ***391.0208*** | ***780.0270*** |  |  |  |  |  |  |  |
|  | [167](http://df3/mascot/cgi/peptide_view.pl?file=../data/20121119/F002569.dat&query=167&hit=1&index=&px=1) | ***391.0242*** | ***780.0338*** |  |  |  |  |  |  |  |
|  | [168](http://df3/mascot/cgi/peptide_view.pl?file=../data/20121119/F002569.dat&query=168&hit=1&index=&px=1) | ***391.0246*** | ***780.0346*** |  |  |  |  |  |  |  |
|  | [169](http://df3/mascot/cgi/peptide_view.pl?file=../data/20121119/F002569.dat&query=169&hit=1&index=&px=1) | ***391.0320*** | ***780.0495*** |  |  |  |  |  |  |  |
|  | [170](http://df3/mascot/cgi/peptide_view.pl?file=../data/20121119/F002569.dat&query=170&hit=1&index=&px=1) | ***391.0322*** | ***780.0498*** |  |  |  |  |  |  |  |
|  | [171](http://df3/mascot/cgi/peptide_view.pl?file=../data/20121119/F002569.dat&query=171&hit=1&index=&px=1) | ***391.0742*** | ***780.1338*** |  |  |  |  |  |  |  |
|  | [172](http://df3/mascot/cgi/peptide_view.pl?file=../data/20121119/F002569.dat&query=172&hit=1&index=&px=1) | ***391.0759*** | ***780.1373*** |  |  |  |  |  |  |  |
|  | [173](http://df3/mascot/cgi/peptide_view.pl?file=../data/20121119/F002569.dat&query=173&hit=1&index=&px=1) | ***391.0807*** | ***780.1469*** |  |  |  |  |  |  |  |
|  | [174](http://df3/mascot/cgi/peptide_view.pl?file=../data/20121119/F002569.dat&query=174&hit=1&index=&px=1) | ***391.0874*** | ***780.1603*** |  |  |  |  |  |  |  |
|  | [175](http://df3/mascot/cgi/peptide_view.pl?file=../data/20121119/F002569.dat&query=175&hit=1&index=&px=1) | ***391.0887*** | ***780.1628*** |  |  |  |  |  |  |  |
|  | [176](http://df3/mascot/cgi/peptide_view.pl?file=../data/20121119/F002569.dat&query=176&hit=1&index=&px=1) | ***391.1052*** | ***780.1959*** |  |  |  |  |  |  |  |
|  | [177](http://df3/mascot/cgi/peptide_view.pl?file=../data/20121119/F002569.dat&query=177&hit=1&index=&px=1) | ***391.1064*** | ***780.1983*** |  |  |  |  |  |  |  |
|  | [178](http://df3/mascot/cgi/peptide_view.pl?file=../data/20121119/F002569.dat&query=178&hit=1&index=&px=1) | ***391.1107*** | ***780.2069*** |  |  |  |  |  |  |  |
|  | [179](http://df3/mascot/cgi/peptide_view.pl?file=../data/20121119/F002569.dat&query=179&hit=1&index=&px=1) | ***391.1133*** | ***780.2120*** |  |  |  |  |  |  |  |
|  | [180](http://df3/mascot/cgi/peptide_view.pl?file=../data/20121119/F002569.dat&query=180&hit=1&index=&px=1) | ***391.1158*** | ***780.2170*** |  |  |  |  |  |  |  |
|  | [181](http://df3/mascot/cgi/peptide_view.pl?file=../data/20121119/F002569.dat&query=181&hit=1&index=&px=1) | ***391.1218*** | ***780.2290*** |  |  |  |  |  |  |  |
|  | [182](http://df3/mascot/cgi/peptide_view.pl?file=../data/20121119/F002569.dat&query=182&hit=1&index=&px=1) | ***391.1250*** | ***780.2355*** |  |  |  |  |  |  |  |
|  | [183](http://df3/mascot/cgi/peptide_view.pl?file=../data/20121119/F002569.dat&query=183&hit=1&index=&px=1) | ***391.1255*** | ***780.2365*** |  |  |  |  |  |  |  |
|  | [184](http://df3/mascot/cgi/peptide_view.pl?file=../data/20121119/F002569.dat&query=184&hit=1&index=&px=1) | ***391.1316*** | ***780.2486*** |  |  |  |  |  |  |  |
|  | [185](http://df3/mascot/cgi/peptide_view.pl?file=../data/20121119/F002569.dat&query=185&hit=1&index=&px=1) | ***391.1323*** | ***780.2501*** |  |  |  |  |  |  |  |
|  | [186](http://df3/mascot/cgi/peptide_view.pl?file=../data/20121119/F002569.dat&query=186&hit=1&index=&px=1) | ***391.1356*** | ***780.2566*** |  |  |  |  |  |  |  |
|  | [187](http://df3/mascot/cgi/peptide_view.pl?file=../data/20121119/F002569.dat&query=187&hit=1&index=&px=1) | ***391.1382*** | ***780.2619*** |  |  |  |  |  |  |  |
|  | [188](http://df3/mascot/cgi/peptide_view.pl?file=../data/20121119/F002569.dat&query=188&hit=1&index=&px=1) | ***391.1431*** | ***780.2717*** |  |  |  |  |  |  |  |
|  | [189](http://df3/mascot/cgi/peptide_view.pl?file=../data/20121119/F002569.dat&query=189&hit=1&index=&px=1) | ***391.1679*** | ***780.3213*** |  |  |  |  |  |  |  |
|  | [190](http://df3/mascot/cgi/peptide_view.pl?file=../data/20121119/F002569.dat&query=190&hit=1&index=&px=1) | ***391.1706*** | ***780.3267*** |  |  |  |  |  |  |  |
|  | [191](http://df3/mascot/cgi/peptide_view.pl?file=../data/20121119/F002569.dat&query=191&hit=1&index=&px=1) | ***391.1714*** | ***780.3283*** |  |  |  |  |  |  |  |
|  | [192](http://df3/mascot/cgi/peptide_view.pl?file=../data/20121119/F002569.dat&query=192&hit=1&index=&px=1) | ***391.1730*** | ***780.3314*** |  |  |  |  |  |  |  |
|  | [193](http://df3/mascot/cgi/peptide_view.pl?file=../data/20121119/F002569.dat&query=193&hit=1&index=&px=1) | ***391.1747*** | ***780.3348*** |  |  |  |  |  |  |  |
|  | [194](http://df3/mascot/cgi/peptide_view.pl?file=../data/20121119/F002569.dat&query=194&hit=1&index=&px=1) | ***391.1811*** | ***780.3477*** |  |  |  |  |  |  |  |
|  | [195](http://df3/mascot/cgi/peptide_view.pl?file=../data/20121119/F002569.dat&query=195&hit=1&index=&px=1) | ***391.1820*** | ***780.3495*** |  |  |  |  |  |  |  |
|  | [196](http://df3/mascot/cgi/peptide_view.pl?file=../data/20121119/F002569.dat&query=196&hit=1&index=&px=1) | ***391.1872*** | ***780.3598*** |  |  |  |  |  |  |  |
|  | [197](http://df3/mascot/cgi/peptide_view.pl?file=../data/20121119/F002569.dat&query=197&hit=1&index=&px=1) | ***391.1960*** | ***780.3774*** |  |  |  |  |  |  |  |
|  | [198](http://df3/mascot/cgi/peptide_view.pl?file=../data/20121119/F002569.dat&query=198&hit=1&index=&px=1) | ***391.1994*** | ***780.3843*** |  |  |  |  |  |  |  |
|  | [199](http://df3/mascot/cgi/peptide_view.pl?file=../data/20121119/F002569.dat&query=199&hit=1&index=&px=1) | ***391.2021*** | ***780.3897*** |  |  |  |  |  |  |  |
|  | [200](http://df3/mascot/cgi/peptide_view.pl?file=../data/20121119/F002569.dat&query=200&hit=1&index=&px=1) | ***391.2030*** | ***780.3915*** |  |  |  |  |  |  |  |
|  | [201](http://df3/mascot/cgi/peptide_view.pl?file=../data/20121119/F002569.dat&query=201&hit=1&index=&px=1) | ***391.2088*** | ***780.4030*** |  |  |  |  |  |  |  |
|  | [202](http://df3/mascot/cgi/peptide_view.pl?file=../data/20121119/F002569.dat&query=202&hit=1&index=&px=1) | ***391.2215*** | ***780.4285*** |  |  |  |  |  |  |  |
|  | [203](http://df3/mascot/cgi/peptide_view.pl?file=../data/20121119/F002569.dat&query=203&hit=1&index=&px=1) | ***391.2227*** | ***780.4308*** |  |  |  |  |  |  |  |
|  | [204](http://df3/mascot/cgi/peptide_view.pl?file=../data/20121119/F002569.dat&query=204&hit=1&index=&px=1) | ***391.2236*** | ***780.4327*** |  |  |  |  |  |  |  |
|  | [205](http://df3/mascot/cgi/peptide_view.pl?file=../data/20121119/F002569.dat&query=205&hit=1&index=&px=1) | ***391.2333*** | ***780.4521*** |  |  |  |  |  |  |  |
|  | [206](http://df3/mascot/cgi/peptide_view.pl?file=../data/20121119/F002569.dat&query=206&hit=1&index=&px=1) | ***391.2443*** | ***780.4741*** |  |  |  |  |  |  |  |
|  | [207](http://df3/mascot/cgi/peptide_view.pl?file=../data/20121119/F002569.dat&query=207&hit=1&index=&px=1) | ***391.2445*** | ***780.4745*** |  |  |  |  |  |  |  |
|  | [208](http://df3/mascot/cgi/peptide_view.pl?file=../data/20121119/F002569.dat&query=208&hit=1&index=&px=1) | ***391.2455*** | ***780.4765*** |  |  |  |  |  |  |  |
|  | [209](http://df3/mascot/cgi/peptide_view.pl?file=../data/20121119/F002569.dat&query=209&hit=1&index=&px=1) | ***391.2479*** | ***780.4812*** |  |  |  |  |  |  |  |
|  | [210](http://df3/mascot/cgi/peptide_view.pl?file=../data/20121119/F002569.dat&query=210&hit=1&index=&px=1) | ***391.2499*** | ***780.4853*** |  |  |  |  |  |  |  |
|  | [211](http://df3/mascot/cgi/peptide_view.pl?file=../data/20121119/F002569.dat&query=211&hit=1&index=&px=1) | ***391.2596*** | ***780.5046*** |  |  |  |  |  |  |  |
|  | [212](http://df3/mascot/cgi/peptide_view.pl?file=../data/20121119/F002569.dat&query=212&hit=1&index=&px=1) | ***391.2634*** | ***780.5122*** |  |  |  |  |  |  |  |
|  | [213](http://df3/mascot/cgi/peptide_view.pl?file=../data/20121119/F002569.dat&query=213&hit=1&index=&px=1) | ***391.2643*** | ***780.5141*** |  |  |  |  |  |  |  |
|  | [214](http://df3/mascot/cgi/peptide_view.pl?file=../data/20121119/F002569.dat&query=214&hit=1&index=&px=1) | ***391.2727*** | ***780.5309*** |  |  |  |  |  |  |  |
|  | [215](http://df3/mascot/cgi/peptide_view.pl?file=../data/20121119/F002569.dat&query=215&hit=1&index=&px=1) | ***391.3079*** | ***780.6013*** |  |  |  |  |  |  |  |
|  | [216](http://df3/mascot/cgi/peptide_view.pl?file=../data/20121119/F002569.dat&query=216&hit=1&index=&px=1) | ***391.3466*** | ***780.6786*** |  |  |  |  |  |  |  |
|  | [217](http://df3/mascot/cgi/peptide_view.pl?file=../data/20121119/F002569.dat&query=217&hit=1&index=&px=1) | ***391.4207*** | ***780.8269*** |  |  |  |  |  |  |  |
|  | [218](http://df3/mascot/cgi/peptide_view.pl?file=../data/20121119/F002569.dat&query=218&hit=1&index=&px=1) | ***392.1603*** | ***782.3061*** |  |  |  |  |  |  |  |
|  | [219](http://df3/mascot/cgi/peptide_view.pl?file=../data/20121119/F002569.dat&query=219&hit=1&index=&px=1) | ***392.1609*** | ***782.3072*** |  |  |  |  |  |  |  |
|  | [220](http://df3/mascot/cgi/peptide_view.pl?file=../data/20121119/F002569.dat&query=220&hit=1&index=&px=1) | ***392.2066*** | ***782.3987*** |  |  |  |  |  |  |  |
|  | [221](http://df3/mascot/cgi/peptide_view.pl?file=../data/20121119/F002569.dat&query=221&hit=1&index=&px=1) | ***392.2115*** | ***782.4085*** |  |  |  |  |  |  |  |
|  | [222](http://df3/mascot/cgi/peptide_view.pl?file=../data/20121119/F002569.dat&query=222&hit=1&index=&px=1) | ***392.2190*** | ***782.4235*** |  |  |  |  |  |  |  |
|  | [223](http://df3/mascot/cgi/peptide_view.pl?file=../data/20121119/F002569.dat&query=223&hit=1&index=&px=1) | ***392.2241*** | ***782.4336*** |  |  |  |  |  |  |  |
|  | [224](http://df3/mascot/cgi/peptide_view.pl?file=../data/20121119/F002569.dat&query=224&hit=1&index=&px=1) | ***392.2350*** | ***782.4555*** |  |  |  |  |  |  |  |
|  | [225](http://df3/mascot/cgi/peptide_view.pl?file=../data/20121119/F002569.dat&query=225&hit=1&index=&px=1) | ***392.2444*** | ***782.4742*** |  |  |  |  |  |  |  |
|  | [226](http://df3/mascot/cgi/peptide_view.pl?file=../data/20121119/F002569.dat&query=226&hit=1&index=&px=1) | ***392.2505*** | ***782.4865*** |  |  |  |  |  |  |  |
|  | [227](http://df3/mascot/cgi/peptide_view.pl?file=../data/20121119/F002569.dat&query=227&hit=1&index=&px=1) | ***392.2669*** | ***782.5193*** |  |  |  |  |  |  |  |
|  | [228](http://df3/mascot/cgi/peptide_view.pl?file=../data/20121119/F002569.dat&query=228&hit=1&index=&px=1) | ***392.2725*** | ***782.5305*** |  |  |  |  |  |  |  |
|  | [229](http://df3/mascot/cgi/peptide_view.pl?file=../data/20121119/F002569.dat&query=229&hit=1&index=&px=1) | ***392.2726*** | ***782.5306*** |  |  |  |  |  |  |  |
|  | [230](http://df3/mascot/cgi/peptide_view.pl?file=../data/20121119/F002569.dat&query=230&hit=1&index=&px=1) | ***392.2733*** | ***782.5320*** |  |  |  |  |  |  |  |
|  | [231](http://df3/mascot/cgi/peptide_view.pl?file=../data/20121119/F002569.dat&query=231&hit=1&index=&px=1) | ***392.2745*** | ***782.5345*** |  |  |  |  |  |  |  |
|  | [232](http://df3/mascot/cgi/peptide_view.pl?file=../data/20121119/F002569.dat&query=232&hit=1&index=&px=1) | ***392.2761*** | ***782.5376*** |  |  |  |  |  |  |  |
|  | [233](http://df3/mascot/cgi/peptide_view.pl?file=../data/20121119/F002569.dat&query=233&hit=1&index=&px=1) | ***392.2854*** | ***782.5563*** |  |  |  |  |  |  |  |
|  | [234](http://df3/mascot/cgi/peptide_view.pl?file=../data/20121119/F002569.dat&query=234&hit=1&index=&px=1) | ***392.2856*** | ***782.5566*** |  |  |  |  |  |  |  |
|  | [235](http://df3/mascot/cgi/peptide_view.pl?file=../data/20121119/F002569.dat&query=235&hit=1&index=&px=1) | ***392.2904*** | ***782.5663*** |  |  |  |  |  |  |  |
|  | [236](http://df3/mascot/cgi/peptide_view.pl?file=../data/20121119/F002569.dat&query=236&hit=1&index=&px=1) | ***392.2961*** | ***782.5775*** |  |  |  |  |  |  |  |
|  | [237](http://df3/mascot/cgi/peptide_view.pl?file=../data/20121119/F002569.dat&query=237&hit=1&index=&px=1) | ***392.2995*** | ***782.5844*** |  |  |  |  |  |  |  |
|  | [238](http://df3/mascot/cgi/peptide_view.pl?file=../data/20121119/F002569.dat&query=238&hit=1&index=&px=1) | ***392.3065*** | ***782.5984*** |  |  |  |  |  |  |  |
|  | [239](http://df3/mascot/cgi/peptide_view.pl?file=../data/20121119/F002569.dat&query=239&hit=1&index=&px=1) | ***392.3102*** | ***782.6059*** |  |  |  |  |  |  |  |
|  | [240](http://df3/mascot/cgi/peptide_view.pl?file=../data/20121119/F002569.dat&query=240&hit=1&index=&px=1) | ***392.3105*** | ***782.6064*** |  |  |  |  |  |  |  |
|  | [241](http://df3/mascot/cgi/peptide_view.pl?file=../data/20121119/F002569.dat&query=241&hit=1&index=&px=1) | ***392.3127*** | ***782.6108*** |  |  |  |  |  |  |  |
|  | [242](http://df3/mascot/cgi/peptide_view.pl?file=../data/20121119/F002569.dat&query=242&hit=1&index=&px=1) | ***392.3165*** | ***782.6184*** |  |  |  |  |  |  |  |
|  | [243](http://df3/mascot/cgi/peptide_view.pl?file=../data/20121119/F002569.dat&query=243&hit=1&index=&px=1) | ***392.3178*** | ***782.6210*** |  |  |  |  |  |  |  |
|  | [244](http://df3/mascot/cgi/peptide_view.pl?file=../data/20121119/F002569.dat&query=244&hit=1&index=&px=1) | ***392.3194*** | ***782.6242*** |  |  |  |  |  |  |  |
|  | [245](http://df3/mascot/cgi/peptide_view.pl?file=../data/20121119/F002569.dat&query=245&hit=1&index=&px=1) | ***392.3267*** | ***782.6389*** |  |  |  |  |  |  |  |
|  | [246](http://df3/mascot/cgi/peptide_view.pl?file=../data/20121119/F002569.dat&query=246&hit=1&index=&px=1) | ***392.3411*** | ***782.6676*** |  |  |  |  |  |  |  |
|  | [247](http://df3/mascot/cgi/peptide_view.pl?file=../data/20121119/F002569.dat&query=247&hit=1&index=&px=1) | ***392.3480*** | ***782.6814*** |  |  |  |  |  |  |  |
|  | [248](http://df3/mascot/cgi/peptide_view.pl?file=../data/20121119/F002569.dat&query=248&hit=1&index=&px=1) | ***392.3484*** | ***782.6822*** |  |  |  |  |  |  |  |
|  | [249](http://df3/mascot/cgi/peptide_view.pl?file=../data/20121119/F002569.dat&query=249&hit=1&index=&px=1) | ***392.3657*** | ***782.7168*** |  |  |  |  |  |  |  |
|  | [250](http://df3/mascot/cgi/peptide_view.pl?file=../data/20121119/F002569.dat&query=250&hit=1&index=&px=1) | ***392.3734*** | ***782.7322*** |  |  |  |  |  |  |  |
|  | [251](http://df3/mascot/cgi/peptide_view.pl?file=../data/20121119/F002569.dat&query=251&hit=1&index=&px=1) | ***392.3985*** | ***782.7824*** |  |  |  |  |  |  |  |
|  | [252](http://df3/mascot/cgi/peptide_view.pl?file=../data/20121119/F002569.dat&query=252&hit=1&index=&px=1) | ***392.4149*** | ***782.8152*** |  |  |  |  |  |  |  |
|  | [253](http://df3/mascot/cgi/peptide_view.pl?file=../data/20121119/F002569.dat&query=253&hit=1&index=&px=1) | ***392.4421*** | ***782.8697*** |  |  |  |  |  |  |  |
|  | [254](http://df3/mascot/cgi/peptide_view.pl?file=../data/20121119/F002569.dat&query=254&hit=1&index=&px=1) | ***392.6055*** | ***783.1964*** |  |  |  |  |  |  |  |
|  | [255](http://df3/mascot/cgi/peptide_view.pl?file=../data/20121119/F002569.dat&query=255&hit=1&index=&px=1) | ***393.4277*** | ***784.8409*** |  |  |  |  |  |  |  |
|  | [256](http://df3/mascot/cgi/peptide_view.pl?file=../data/20121119/F002569.dat&query=256&hit=1&index=&px=1) | ***395.2553*** | ***788.4960*** |  |  |  |  |  |  |  |
|  | [257](http://df3/mascot/cgi/peptide_view.pl?file=../data/20121119/F002569.dat&query=257&hit=1&index=&px=1) | ***395.2764*** | ***788.5382*** |  |  |  |  |  |  |  |
|  | [258](http://df3/mascot/cgi/peptide_view.pl?file=../data/20121119/F002569.dat&query=258&hit=1&index=&px=1) | ***395.4505*** | ***788.8864*** |  |  |  |  |  |  |  |
|  | [259](http://df3/mascot/cgi/peptide_view.pl?file=../data/20121119/F002569.dat&query=259&hit=1&index=&px=1) | ***395.4807*** | ***788.9468*** |  |  |  |  |  |  |  |
|  | [260](http://df3/mascot/cgi/peptide_view.pl?file=../data/20121119/F002569.dat&query=260&hit=1&index=&px=1) | ***395.6323*** | ***789.2500*** |  |  |  |  |  |  |  |
|  | [261](http://df3/mascot/cgi/peptide_view.pl?file=../data/20121119/F002569.dat&query=261&hit=1&index=&px=1) | ***397.8568*** | ***793.6990*** |  |  |  |  |  |  |  |
|  | [262](http://df3/mascot/cgi/peptide_view.pl?file=../data/20121119/F002569.dat&query=262&hit=1&index=&px=1) | ***397.9457*** | ***793.8768*** |  |  |  |  |  |  |  |
|  | [263](http://df3/mascot/cgi/peptide_view.pl?file=../data/20121119/F002569.dat&query=263&hit=1&index=&px=1) | ***399.1247*** | ***796.2348*** |  |  |  |  |  |  |  |
|  | [264](http://df3/mascot/cgi/peptide_view.pl?file=../data/20121119/F002569.dat&query=264&hit=1&index=&px=1) | ***399.4828*** | ***796.9510*** |  |  |  |  |  |  |  |
|  | [265](http://df3/mascot/cgi/peptide_view.pl?file=../data/20121119/F002569.dat&query=265&hit=1&index=&px=1) | ***400.0175*** | ***798.0204*** |  |  |  |  |  |  |  |
|  | [266](http://df3/mascot/cgi/peptide_view.pl?file=../data/20121119/F002569.dat&query=266&hit=1&index=&px=1) | ***400.2280*** | ***798.4415*** |  |  |  |  |  |  |  |
|  | [267](http://df3/mascot/cgi/peptide_view.pl?file=../data/20121119/F002569.dat&query=267&hit=1&index=&px=1) | ***400.2849*** | ***798.5553*** |  |  |  |  |  |  |  |
|  | [268](http://df3/mascot/cgi/peptide_view.pl?file=../data/20121119/F002569.dat&query=268&hit=1&index=&px=1) | ***400.2952*** | ***798.5758*** |  |  |  |  |  |  |  |
|  | [269](http://df3/mascot/cgi/peptide_view.pl?file=../data/20121119/F002569.dat&query=269&hit=1&index=&px=1) | ***401.3780*** | ***800.7415*** |  |  |  |  |  |  |  |
|  | [270](http://df3/mascot/cgi/peptide_view.pl?file=../data/20121119/F002569.dat&query=270&hit=1&index=&px=1) | ***401.5057*** | ***800.9968*** |  |  |  |  |  |  |  |
|  | [271](http://df3/mascot/cgi/peptide_view.pl?file=../data/20121119/F002569.dat&query=271&hit=1&index=&px=1) | ***402.1885*** | ***802.3624*** |  |  |  |  |  |  |  |
|  | [272](http://df3/mascot/cgi/peptide_view.pl?file=../data/20121119/F002569.dat&query=272&hit=1&index=&px=1) | ***402.6550*** | ***803.2954*** |  |  |  |  |  |  |  |
|  | [273](http://df3/mascot/cgi/peptide_view.pl?file=../data/20121119/F002569.dat&query=273&hit=1&index=&px=1) | ***403.9877*** | ***805.9609*** |  |  |  |  |  |  |  |
|  | [274](http://df3/mascot/cgi/peptide_view.pl?file=../data/20121119/F002569.dat&query=274&hit=1&index=&px=1) | ***405.5253*** | ***809.0360*** |  |  |  |  |  |  |  |
|  | [275](http://df3/mascot/cgi/peptide_view.pl?file=../data/20121119/F002569.dat&query=275&hit=1&index=&px=1) | ***405.6557*** | ***809.2969*** |  |  |  |  |  |  |  |
|  | [276](http://df3/mascot/cgi/peptide_view.pl?file=../data/20121119/F002569.dat&query=276&hit=1&index=&px=1) | ***405.8868*** | ***809.7591*** |  |  |  |  |  |  |  |
|  | [278](http://df3/mascot/cgi/peptide_view.pl?file=../data/20121119/F002569.dat&query=278&hit=1&index=&px=1) | ***406.1102*** | ***810.2059*** |  |  |  |  |  |  |  |
|  | [279](http://df3/mascot/cgi/peptide_view.pl?file=../data/20121119/F002569.dat&query=279&hit=1&index=&px=1) | ***406.1336*** | ***810.2527*** |  |  |  |  |  |  |  |
|  | [280](http://df3/mascot/cgi/peptide_view.pl?file=../data/20121119/F002569.dat&query=280&hit=1&index=&px=1) | ***406.1667*** | ***810.3188*** |  |  |  |  |  |  |  |
|  | [281](http://df3/mascot/cgi/peptide_view.pl?file=../data/20121119/F002569.dat&query=281&hit=1&index=&px=1) | ***406.1768*** | ***810.3390*** |  |  |  |  |  |  |  |
|  | [282](http://df3/mascot/cgi/peptide_view.pl?file=../data/20121119/F002569.dat&query=282&hit=1&index=&px=1) | ***406.2182*** | ***810.4219*** |  |  |  |  |  |  |  |
|  | [283](http://df3/mascot/cgi/peptide_view.pl?file=../data/20121119/F002569.dat&query=283&hit=1&index=&px=1) | ***406.9893*** | ***811.9641*** |  |  |  |  |  |  |  |
|  | [284](http://df3/mascot/cgi/peptide_view.pl?file=../data/20121119/F002569.dat&query=284&hit=1&index=&px=1) | ***407.2542*** | ***812.4939*** |  |  |  |  |  |  |  |
|  | [285](http://df3/mascot/cgi/peptide_view.pl?file=../data/20121119/F002569.dat&query=285&hit=1&index=&px=1) | ***407.2721*** | ***812.5296*** |  |  |  |  |  |  |  |
|  | [286](http://df3/mascot/cgi/peptide_view.pl?file=../data/20121119/F002569.dat&query=286&hit=1&index=&px=1) | ***407.4270*** | ***812.8395*** |  |  |  |  |  |  |  |
|  | [287](http://df3/mascot/cgi/peptide_view.pl?file=../data/20121119/F002569.dat&query=287&hit=1&index=&px=1) | ***407.4772*** | ***812.9399*** |  |  |  |  |  |  |  |
|  | [288](http://df3/mascot/cgi/peptide_view.pl?file=../data/20121119/F002569.dat&query=288&hit=1&index=&px=1) | ***408.0507*** | ***814.0869*** |  |  |  |  |  |  |  |
|  | [289](http://df3/mascot/cgi/peptide_view.pl?file=../data/20121119/F002569.dat&query=289&hit=1&index=&px=1) | ***409.1576*** | ***816.3007*** |  |  |  |  |  |  |  |
|  | [290](http://df3/mascot/cgi/peptide_view.pl?file=../data/20121119/F002569.dat&query=290&hit=1&index=&px=1) | ***409.8481*** | ***817.6816*** |  |  |  |  |  |  |  |
|  | [291](http://df3/mascot/cgi/peptide_view.pl?file=../data/20121119/F002569.dat&query=291&hit=1&index=&px=1) | ***410.2175*** | ***818.4205*** |  |  |  |  |  |  |  |
|  | [292](http://df3/mascot/cgi/peptide_view.pl?file=../data/20121119/F002569.dat&query=292&hit=1&index=&px=1) | ***412.4295*** | ***822.8445*** |  |  |  |  |  |  |  |
|  | [294](http://df3/mascot/cgi/peptide_view.pl?file=../data/20121119/F002569.dat&query=294&hit=1&index=&px=1) | ***413.7672*** | ***825.5198*** |  |  |  |  |  |  |  |
|  | [295](http://df3/mascot/cgi/peptide_view.pl?file=../data/20121119/F002569.dat&query=295&hit=1&index=&px=1) | ***414.8416*** | ***827.6686*** |  |  |  |  |  |  |  |
|  | [296](http://df3/mascot/cgi/peptide_view.pl?file=../data/20121119/F002569.dat&query=296&hit=1&index=&px=1) | ***414.9301*** | ***827.8456*** |  |  |  |  |  |  |  |
|  | [297](http://df3/mascot/cgi/peptide_view.pl?file=../data/20121119/F002569.dat&query=297&hit=1&index=&px=1) | ***415.2505*** | ***828.4864*** |  |  |  |  |  |  |  |
|  | [298](http://df3/mascot/cgi/peptide_view.pl?file=../data/20121119/F002569.dat&query=298&hit=1&index=&px=1) | ***416.0879*** | ***830.1612*** |  |  |  |  |  |  |  |
|  | [302](http://df3/mascot/cgi/peptide_view.pl?file=../data/20121119/F002569.dat&query=302&hit=1&index=&px=1) | ***416.3562*** | ***830.6979*** |  |  |  |  |  |  |  |
|  | [303](http://df3/mascot/cgi/peptide_view.pl?file=../data/20121119/F002569.dat&query=303&hit=1&index=&px=1) | ***417.2857*** | ***832.5568*** |  |  |  |  |  |  |  |
|  | [304](http://df3/mascot/cgi/peptide_view.pl?file=../data/20121119/F002569.dat&query=304&hit=1&index=&px=1) | ***417.7104*** | ***833.4063*** |  |  |  |  |  |  |  |
|  | [305](http://df3/mascot/cgi/peptide_view.pl?file=../data/20121119/F002569.dat&query=305&hit=1&index=&px=1) | ***418.5856*** | ***835.1567*** |  |  |  |  |  |  |  |
|  | [306](http://df3/mascot/cgi/peptide_view.pl?file=../data/20121119/F002569.dat&query=306&hit=1&index=&px=1) | ***418.6716*** | ***835.3287*** |  |  |  |  |  |  |  |
|  | [307](http://df3/mascot/cgi/peptide_view.pl?file=../data/20121119/F002569.dat&query=307&hit=1&index=&px=1) | ***418.6855*** | ***835.3565*** |  |  |  |  |  |  |  |
|  | [308](http://df3/mascot/cgi/peptide_view.pl?file=../data/20121119/F002569.dat&query=308&hit=1&index=&px=1) | ***418.7443*** | ***835.4741*** |  |  |  |  |  |  |  |
|  | [309](http://df3/mascot/cgi/peptide_view.pl?file=../data/20121119/F002569.dat&query=309&hit=1&index=&px=1) | ***418.7910*** | ***835.5675*** |  |  |  |  |  |  |  |
|  | [310](http://df3/mascot/cgi/peptide_view.pl?file=../data/20121119/F002569.dat&query=310&hit=1&index=&px=1) | ***418.8629*** | ***835.7112*** |  |  |  |  |  |  |  |
|  | [311](http://df3/mascot/cgi/peptide_view.pl?file=../data/20121119/F002569.dat&query=311&hit=1&index=&px=1) | ***418.9241*** | ***835.8336*** |  |  |  |  |  |  |  |
|  | [312](http://df3/mascot/cgi/peptide_view.pl?file=../data/20121119/F002569.dat&query=312&hit=1&index=&px=1) | ***418.9597*** | ***835.9049*** |  |  |  |  |  |  |  |
|  | [313](http://df3/mascot/cgi/peptide_view.pl?file=../data/20121119/F002569.dat&query=313&hit=1&index=&px=1) | ***420.0103*** | ***838.0060*** |  |  |  |  |  |  |  |
|  | [314](http://df3/mascot/cgi/peptide_view.pl?file=../data/20121119/F002569.dat&query=314&hit=1&index=&px=1) | ***420.0174*** | ***838.0203*** |  |  |  |  |  |  |  |
|  | [316](http://df3/mascot/cgi/peptide_view.pl?file=../data/20121119/F002569.dat&query=316&hit=1&index=&px=1) | ***420.0822*** | ***838.1498*** |  |  |  |  |  |  |  |
|  | [318](http://df3/mascot/cgi/peptide_view.pl?file=../data/20121119/F002569.dat&query=318&hit=1&index=&px=1) | ***420.1386*** | ***838.2625*** |  |  |  |  |  |  |  |
|  | [319](http://df3/mascot/cgi/peptide_view.pl?file=../data/20121119/F002569.dat&query=319&hit=1&index=&px=1) | ***420.2698*** | ***838.5251*** |  |  |  |  |  |  |  |
|  | [320](http://df3/mascot/cgi/peptide_view.pl?file=../data/20121119/F002569.dat&query=320&hit=1&index=&px=1) | ***420.6325*** | ***839.2504*** |  |  |  |  |  |  |  |
|  | [324](http://df3/mascot/cgi/peptide_view.pl?file=../data/20121119/F002569.dat&query=324&hit=1&index=&px=1) | ***421.6151*** | ***841.2156*** |  |  |  |  |  |  |  |
|  | [327](http://df3/mascot/cgi/peptide_view.pl?file=../data/20121119/F002569.dat&query=327&hit=1&index=&px=1) | ***422.1120*** | ***842.2095*** |  |  |  |  |  |  |  |
|  | [328](http://df3/mascot/cgi/peptide_view.pl?file=../data/20121119/F002569.dat&query=328&hit=1&index=&px=1) | ***422.1475*** | ***842.2804*** |  |  |  |  |  |  |  |
|  | [329](http://df3/mascot/cgi/peptide_view.pl?file=../data/20121119/F002569.dat&query=329&hit=1&index=&px=1) | ***423.3176*** | ***844.6206*** |  |  |  |  |  |  |  |
|  | [330](http://df3/mascot/cgi/peptide_view.pl?file=../data/20121119/F002569.dat&query=330&hit=1&index=&px=1) | ***423.3684*** | ***844.7223*** |  |  |  |  |  |  |  |
|  | [331](http://df3/mascot/cgi/peptide_view.pl?file=../data/20121119/F002569.dat&query=331&hit=1&index=&px=1) | ***423.4164*** | ***844.8182*** |  |  |  |  |  |  |  |
|  | [332](http://df3/mascot/cgi/peptide_view.pl?file=../data/20121119/F002569.dat&query=332&hit=1&index=&px=1) | ***423.8357*** | ***845.6568*** |  |  |  |  |  |  |  |
|  | [333](http://df3/mascot/cgi/peptide_view.pl?file=../data/20121119/F002569.dat&query=333&hit=1&index=&px=1) | ***424.1064*** | ***846.1983*** |  |  |  |  |  |  |  |
|  | [336](http://df3/mascot/cgi/peptide_view.pl?file=../data/20121119/F002569.dat&query=336&hit=1&index=&px=1) | ***425.5248*** | ***849.0351*** |  |  |  |  |  |  |  |
|  | [337](http://df3/mascot/cgi/peptide_view.pl?file=../data/20121119/F002569.dat&query=337&hit=1&index=&px=1) | ***426.7794*** | ***851.5442*** |  |  |  |  |  |  |  |
|  | [338](http://df3/mascot/cgi/peptide_view.pl?file=../data/20121119/F002569.dat&query=338&hit=1&index=&px=1) | ***426.7936*** | ***851.5726*** |  |  |  |  |  |  |  |
|  | [339](http://df3/mascot/cgi/peptide_view.pl?file=../data/20121119/F002569.dat&query=339&hit=1&index=&px=1) | ***426.8973*** | ***851.7801*** |  |  |  |  |  |  |  |
|  | [340](http://df3/mascot/cgi/peptide_view.pl?file=../data/20121119/F002569.dat&query=340&hit=1&index=&px=1) | ***427.2065*** | ***852.3984*** |  |  |  |  |  |  |  |
|  | [341](http://df3/mascot/cgi/peptide_view.pl?file=../data/20121119/F002569.dat&query=341&hit=1&index=&px=1) | ***429.2530*** | ***856.4914*** |  |  |  |  |  |  |  |
|  | [342](http://df3/mascot/cgi/peptide_view.pl?file=../data/20121119/F002569.dat&query=342&hit=1&index=&px=1) | ***429.3167*** | ***856.6189*** |  |  |  |  |  |  |  |
|  | [343](http://df3/mascot/cgi/peptide_view.pl?file=../data/20121119/F002569.dat&query=343&hit=1&index=&px=1) | ***429.3909*** | ***856.7673*** |  |  |  |  |  |  |  |
|  | [344](http://df3/mascot/cgi/peptide_view.pl?file=../data/20121119/F002569.dat&query=344&hit=1&index=&px=1) | ***429.4146*** | ***856.8146*** |  |  |  |  |  |  |  |
|  | [345](http://df3/mascot/cgi/peptide_view.pl?file=../data/20121119/F002569.dat&query=345&hit=1&index=&px=1) | ***429.4489*** | ***856.8832*** |  |  |  |  |  |  |  |
|  | [346](http://df3/mascot/cgi/peptide_view.pl?file=../data/20121119/F002569.dat&query=346&hit=1&index=&px=1) | ***429.5441*** | ***857.0736*** |  |  |  |  |  |  |  |
|  | [347](http://df3/mascot/cgi/peptide_view.pl?file=../data/20121119/F002569.dat&query=347&hit=1&index=&px=1) | ***429.6416*** | ***857.2687*** |  |  |  |  |  |  |  |
|  | [348](http://df3/mascot/cgi/peptide_view.pl?file=../data/20121119/F002569.dat&query=348&hit=1&index=&px=1) | ***429.7788*** | ***857.5430*** |  |  |  |  |  |  |  |
|  | [349](http://df3/mascot/cgi/peptide_view.pl?file=../data/20121119/F002569.dat&query=349&hit=1&index=&px=1) | ***429.8798*** | ***857.7451*** |  |  |  |  |  |  |  |
|  | [350](http://df3/mascot/cgi/peptide_view.pl?file=../data/20121119/F002569.dat&query=350&hit=1&index=&px=1) | ***429.9331*** | ***857.8517*** |  |  |  |  |  |  |  |
|  | [351](http://df3/mascot/cgi/peptide_view.pl?file=../data/20121119/F002569.dat&query=351&hit=1&index=&px=1) | ***431.9734*** | ***861.9322*** |  |  |  |  |  |  |  |
|  | [352](http://df3/mascot/cgi/peptide_view.pl?file=../data/20121119/F002569.dat&query=352&hit=1&index=&px=1) | ***432.7985*** | ***863.5825*** |  |  |  |  |  |  |  |
|  | [353](http://df3/mascot/cgi/peptide_view.pl?file=../data/20121119/F002569.dat&query=353&hit=1&index=&px=1) | ***433.8279*** | ***865.6411*** |  |  |  |  |  |  |  |
|  | [354](http://df3/mascot/cgi/peptide_view.pl?file=../data/20121119/F002569.dat&query=354&hit=1&index=&px=1) | ***433.9408*** | ***865.8671*** |  |  |  |  |  |  |  |
|  | [355](http://df3/mascot/cgi/peptide_view.pl?file=../data/20121119/F002569.dat&query=355&hit=1&index=&px=1) | ***433.9730*** | ***865.9314*** |  |  |  |  |  |  |  |
|  | [356](http://df3/mascot/cgi/peptide_view.pl?file=../data/20121119/F002569.dat&query=356&hit=1&index=&px=1) | ***434.2704*** | ***866.5262*** |  |  |  |  |  |  |  |
|  | [357](http://df3/mascot/cgi/peptide_view.pl?file=../data/20121119/F002569.dat&query=357&hit=1&index=&px=1) | ***434.3139*** | ***866.6133*** |  |  |  |  |  |  |  |
|  | [358](http://df3/mascot/cgi/peptide_view.pl?file=../data/20121119/F002569.dat&query=358&hit=1&index=&px=1) | ***434.3409*** | ***866.6673*** |  |  |  |  |  |  |  |
|  | [359](http://df3/mascot/cgi/peptide_view.pl?file=../data/20121119/F002569.dat&query=359&hit=1&index=&px=1) | ***434.4007*** | ***866.7868*** |  |  |  |  |  |  |  |
|  | [360](http://df3/mascot/cgi/peptide_view.pl?file=../data/20121119/F002569.dat&query=360&hit=1&index=&px=1) | ***434.4892*** | ***866.9638*** |  |  |  |  |  |  |  |
|  | [361](http://df3/mascot/cgi/peptide_view.pl?file=../data/20121119/F002569.dat&query=361&hit=1&index=&px=1) | ***435.0342*** | ***868.0539*** |  |  |  |  |  |  |  |
|  | [362](http://df3/mascot/cgi/peptide_view.pl?file=../data/20121119/F002569.dat&query=362&hit=1&index=&px=1) | ***435.3889*** | ***868.7632*** |  |  |  |  |  |  |  |
|  | [363](http://df3/mascot/cgi/peptide_view.pl?file=../data/20121119/F002569.dat&query=363&hit=1&index=&px=1) | ***435.4037*** | ***868.7929*** |  |  |  |  |  |  |  |
|  | [364](http://df3/mascot/cgi/peptide_view.pl?file=../data/20121119/F002569.dat&query=364&hit=1&index=&px=1) | ***435.4678*** | ***868.9211*** |  |  |  |  |  |  |  |
|  | [365](http://df3/mascot/cgi/peptide_view.pl?file=../data/20121119/F002569.dat&query=365&hit=1&index=&px=1) | ***435.5141*** | ***869.0136*** |  |  |  |  |  |  |  |
|  | [366](http://df3/mascot/cgi/peptide_view.pl?file=../data/20121119/F002569.dat&query=366&hit=1&index=&px=1) | ***435.5394*** | ***869.0643*** |  |  |  |  |  |  |  |
|  | [367](http://df3/mascot/cgi/peptide_view.pl?file=../data/20121119/F002569.dat&query=367&hit=1&index=&px=1) | ***435.7209*** | ***869.4273*** |  |  |  |  |  |  |  |
|  | [368](http://df3/mascot/cgi/peptide_view.pl?file=../data/20121119/F002569.dat&query=368&hit=1&index=&px=1) | ***435.7838*** | ***869.5531*** |  |  |  |  |  |  |  |
|  | [369](http://df3/mascot/cgi/peptide_view.pl?file=../data/20121119/F002569.dat&query=369&hit=1&index=&px=1) | ***435.7902*** | ***869.5659*** |  |  |  |  |  |  |  |
|  | [370](http://df3/mascot/cgi/peptide_view.pl?file=../data/20121119/F002569.dat&query=370&hit=1&index=&px=1) | ***435.8725*** | ***869.7304*** |  |  |  |  |  |  |  |
|  | [371](http://df3/mascot/cgi/peptide_view.pl?file=../data/20121119/F002569.dat&query=371&hit=1&index=&px=1) | ***435.8844*** | ***869.7542*** |  |  |  |  |  |  |  |
|  | [372](http://df3/mascot/cgi/peptide_view.pl?file=../data/20121119/F002569.dat&query=372&hit=1&index=&px=1) | ***435.9081*** | ***869.8016*** |  |  |  |  |  |  |  |
|  | [373](http://df3/mascot/cgi/peptide_view.pl?file=../data/20121119/F002569.dat&query=373&hit=1&index=&px=1) | ***435.9745*** | ***869.9345*** |  |  |  |  |  |  |  |
|  | [374](http://df3/mascot/cgi/peptide_view.pl?file=../data/20121119/F002569.dat&query=374&hit=1&index=&px=1) | ***436.0425*** | ***870.0704*** |  |  |  |  |  |  |  |
|  | [375](http://df3/mascot/cgi/peptide_view.pl?file=../data/20121119/F002569.dat&query=375&hit=1&index=&px=1) | ***436.0656*** | ***870.1166*** |  |  |  |  |  |  |  |
|  | [376](http://df3/mascot/cgi/peptide_view.pl?file=../data/20121119/F002569.dat&query=376&hit=1&index=&px=1) | ***436.0734*** | ***870.1323*** |  |  |  |  |  |  |  |
|  | [377](http://df3/mascot/cgi/peptide_view.pl?file=../data/20121119/F002569.dat&query=377&hit=1&index=&px=1) | ***436.1888*** | ***870.3631*** |  |  |  |  |  |  |  |
|  | [378](http://df3/mascot/cgi/peptide_view.pl?file=../data/20121119/F002569.dat&query=378&hit=1&index=&px=1) | ***436.8236*** | ***871.6326*** |  |  |  |  |  |  |  |
|  | [379](http://df3/mascot/cgi/peptide_view.pl?file=../data/20121119/F002569.dat&query=379&hit=1&index=&px=1) | ***436.9567*** | ***871.8989*** |  |  |  |  |  |  |  |
|  | [380](http://df3/mascot/cgi/peptide_view.pl?file=../data/20121119/F002569.dat&query=380&hit=1&index=&px=1) | ***436.9628*** | ***871.9110*** |  |  |  |  |  |  |  |
|  | [381](http://df3/mascot/cgi/peptide_view.pl?file=../data/20121119/F002569.dat&query=381&hit=1&index=&px=1) | ***436.9764*** | ***871.9382*** |  |  |  |  |  |  |  |
|  | [382](http://df3/mascot/cgi/peptide_view.pl?file=../data/20121119/F002569.dat&query=382&hit=1&index=&px=1) | ***436.9922*** | ***871.9699*** |  |  |  |  |  |  |  |
|  | [383](http://df3/mascot/cgi/peptide_view.pl?file=../data/20121119/F002569.dat&query=383&hit=1&index=&px=1) | ***437.0244*** | ***872.0343*** |  |  |  |  |  |  |  |
|  | [384](http://df3/mascot/cgi/peptide_view.pl?file=../data/20121119/F002569.dat&query=384&hit=1&index=&px=1) | ***437.2910*** | ***872.5675*** |  |  |  |  |  |  |  |
|  | [385](http://df3/mascot/cgi/peptide_view.pl?file=../data/20121119/F002569.dat&query=385&hit=1&index=&px=1) | ***437.9452*** | ***873.8758*** |  |  |  |  |  |  |  |
|  | [386](http://df3/mascot/cgi/peptide_view.pl?file=../data/20121119/F002569.dat&query=386&hit=1&index=&px=1) | ***437.9561*** | ***873.8977*** |  |  |  |  |  |  |  |
|  | [387](http://df3/mascot/cgi/peptide_view.pl?file=../data/20121119/F002569.dat&query=387&hit=1&index=&px=1) | ***437.9842*** | ***873.9538*** |  |  |  |  |  |  |  |
|  | [388](http://df3/mascot/cgi/peptide_view.pl?file=../data/20121119/F002569.dat&query=388&hit=1&index=&px=1) | ***438.0482*** | ***874.0819*** |  |  |  |  |  |  |  |
|  | [389](http://df3/mascot/cgi/peptide_view.pl?file=../data/20121119/F002569.dat&query=389&hit=1&index=&px=1) | ***438.4398*** | ***874.8650*** |  |  |  |  |  |  |  |
|  | [390](http://df3/mascot/cgi/peptide_view.pl?file=../data/20121119/F002569.dat&query=390&hit=1&index=&px=1) | ***439.7156*** | ***877.4167*** |  |  |  |  |  |  |  |
|  | [391](http://df3/mascot/cgi/peptide_view.pl?file=../data/20121119/F002569.dat&query=391&hit=1&index=&px=1) | ***441.1380*** | ***880.2614*** |  |  |  |  |  |  |  |
|  | [392](http://df3/mascot/cgi/peptide_view.pl?file=../data/20121119/F002569.dat&query=392&hit=1&index=&px=1) | ***441.8363*** | ***881.6581*** |  |  |  |  |  |  |  |
|  | [393](http://df3/mascot/cgi/peptide_view.pl?file=../data/20121119/F002569.dat&query=393&hit=1&index=&px=1) | ***441.8598*** | ***881.7050*** |  |  |  |  |  |  |  |
|  | [394](http://df3/mascot/cgi/peptide_view.pl?file=../data/20121119/F002569.dat&query=394&hit=1&index=&px=1) | ***442.0923*** | ***882.1701*** |  |  |  |  |  |  |  |
|  | [395](http://df3/mascot/cgi/peptide_view.pl?file=../data/20121119/F002569.dat&query=395&hit=1&index=&px=1) | ***442.2932*** | ***882.5719*** |  |  |  |  |  |  |  |
|  | [396](http://df3/mascot/cgi/peptide_view.pl?file=../data/20121119/F002569.dat&query=396&hit=1&index=&px=1) | ***442.6044*** | ***883.1943*** |  |  |  |  |  |  |  |
|  | [397](http://df3/mascot/cgi/peptide_view.pl?file=../data/20121119/F002569.dat&query=397&hit=1&index=&px=1) | ***442.8777*** | ***883.7409*** |  |  |  |  |  |  |  |
|  | [398](http://df3/mascot/cgi/peptide_view.pl?file=../data/20121119/F002569.dat&query=398&hit=1&index=&px=1) | ***443.0196*** | ***884.0246*** |  |  |  |  |  |  |  |
|  | [402](http://df3/mascot/cgi/peptide_view.pl?file=../data/20121119/F002569.dat&query=402&hit=1&index=&px=1) | ***443.7538*** | ***885.4930*** |  |  |  |  |  |  |  |
|  | [404](http://df3/mascot/cgi/peptide_view.pl?file=../data/20121119/F002569.dat&query=404&hit=1&index=&px=1) | ***444.0424*** | ***886.0703*** |  |  |  |  |  |  |  |
|  | [407](http://df3/mascot/cgi/peptide_view.pl?file=../data/20121119/F002569.dat&query=407&hit=1&index=&px=1) | ***444.8951*** | ***887.7755*** |  |  |  |  |  |  |  |
|  | [408](http://df3/mascot/cgi/peptide_view.pl?file=../data/20121119/F002569.dat&query=408&hit=1&index=&px=1) | ***444.9422*** | ***887.8699*** |  |  |  |  |  |  |  |
|  | [409](http://df3/mascot/cgi/peptide_view.pl?file=../data/20121119/F002569.dat&query=409&hit=1&index=&px=1) | ***444.9609*** | ***887.9073*** |  |  |  |  |  |  |  |
|  | [410](http://df3/mascot/cgi/peptide_view.pl?file=../data/20121119/F002569.dat&query=410&hit=1&index=&px=1) | ***444.9714*** | ***887.9283*** |  |  |  |  |  |  |  |
|  | [411](http://df3/mascot/cgi/peptide_view.pl?file=../data/20121119/F002569.dat&query=411&hit=1&index=&px=1) | ***445.0604*** | ***888.1063*** |  |  |  |  |  |  |  |
|  | [413](http://df3/mascot/cgi/peptide_view.pl?file=../data/20121119/F002569.dat&query=413&hit=1&index=&px=1) | ***445.1792*** | ***888.3438*** |  |  |  |  |  |  |  |
|  | [414](http://df3/mascot/cgi/peptide_view.pl?file=../data/20121119/F002569.dat&query=414&hit=1&index=&px=1) | ***445.1962*** | ***888.3779*** |  |  |  |  |  |  |  |
|  | [415](http://df3/mascot/cgi/peptide_view.pl?file=../data/20121119/F002569.dat&query=415&hit=1&index=&px=1) | ***445.2902*** | ***888.5659*** |  |  |  |  |  |  |  |
|  | [417](http://df3/mascot/cgi/peptide_view.pl?file=../data/20121119/F002569.dat&query=417&hit=1&index=&px=1) | ***446.7298*** | ***891.4451*** |  |  |  |  |  |  |  |
|  | [421](http://df3/mascot/cgi/peptide_view.pl?file=../data/20121119/F002569.dat&query=421&hit=1&index=&px=1) | ***447.0112*** | ***892.0079*** |  |  |  |  |  |  |  |
|  | [423](http://df3/mascot/cgi/peptide_view.pl?file=../data/20121119/F002569.dat&query=423&hit=1&index=&px=1) | ***447.0374*** | ***892.0603*** |  |  |  |  |  |  |  |
|  | [425](http://df3/mascot/cgi/peptide_view.pl?file=../data/20121119/F002569.dat&query=425&hit=1&index=&px=1) | ***447.0672*** | ***892.1198*** |  |  |  |  |  |  |  |
|  | [426](http://df3/mascot/cgi/peptide_view.pl?file=../data/20121119/F002569.dat&query=426&hit=1&index=&px=1) | ***447.0837*** | ***892.1529*** |  |  |  |  |  |  |  |
|  | [427](http://df3/mascot/cgi/peptide_view.pl?file=../data/20121119/F002569.dat&query=427&hit=1&index=&px=1) | ***447.0843*** | ***892.1540*** |  |  |  |  |  |  |  |
|  | [428](http://df3/mascot/cgi/peptide_view.pl?file=../data/20121119/F002569.dat&query=428&hit=1&index=&px=1) | ***447.0855*** | ***892.1565*** |  |  |  |  |  |  |  |
|  | [429](http://df3/mascot/cgi/peptide_view.pl?file=../data/20121119/F002569.dat&query=429&hit=1&index=&px=1) | ***447.0865*** | ***892.1584*** |  |  |  |  |  |  |  |
|  | [430](http://df3/mascot/cgi/peptide_view.pl?file=../data/20121119/F002569.dat&query=430&hit=1&index=&px=1) | ***447.0896*** | ***892.1647*** |  |  |  |  |  |  |  |
|  | [431](http://df3/mascot/cgi/peptide_view.pl?file=../data/20121119/F002569.dat&query=431&hit=1&index=&px=1) | ***447.0911*** | ***892.1676*** |  |  |  |  |  |  |  |
|  | [432](http://df3/mascot/cgi/peptide_view.pl?file=../data/20121119/F002569.dat&query=432&hit=1&index=&px=1) | ***447.0926*** | ***892.1706*** |  |  |  |  |  |  |  |
|  | [433](http://df3/mascot/cgi/peptide_view.pl?file=../data/20121119/F002569.dat&query=433&hit=1&index=&px=1) | ***447.0927*** | ***892.1709*** |  |  |  |  |  |  |  |
|  | [434](http://df3/mascot/cgi/peptide_view.pl?file=../data/20121119/F002569.dat&query=434&hit=1&index=&px=1) | ***447.0956*** | ***892.1766*** |  |  |  |  |  |  |  |
|  | [435](http://df3/mascot/cgi/peptide_view.pl?file=../data/20121119/F002569.dat&query=435&hit=1&index=&px=1) | ***447.1026*** | ***892.1907*** |  |  |  |  |  |  |  |
|  | [437](http://df3/mascot/cgi/peptide_view.pl?file=../data/20121119/F002569.dat&query=437&hit=1&index=&px=1) | ***447.1105*** | ***892.2065*** |  |  |  |  |  |  |  |
|  | [438](http://df3/mascot/cgi/peptide_view.pl?file=../data/20121119/F002569.dat&query=438&hit=1&index=&px=1) | ***447.1172*** | ***892.2199*** |  |  |  |  |  |  |  |
|  | [439](http://df3/mascot/cgi/peptide_view.pl?file=../data/20121119/F002569.dat&query=439&hit=1&index=&px=1) | ***447.1334*** | ***892.2522*** |  |  |  |  |  |  |  |
|  | [440](http://df3/mascot/cgi/peptide_view.pl?file=../data/20121119/F002569.dat&query=440&hit=1&index=&px=1) | ***447.1356*** | ***892.2567*** |  |  |  |  |  |  |  |
|  | [441](http://df3/mascot/cgi/peptide_view.pl?file=../data/20121119/F002569.dat&query=441&hit=1&index=&px=1) | ***447.1378*** | ***892.2611*** |  |  |  |  |  |  |  |
|  | [442](http://df3/mascot/cgi/peptide_view.pl?file=../data/20121119/F002569.dat&query=442&hit=1&index=&px=1) | ***447.1792*** | ***892.3438*** |  |  |  |  |  |  |  |
|  | [443](http://df3/mascot/cgi/peptide_view.pl?file=../data/20121119/F002569.dat&query=443&hit=1&index=&px=1) | ***447.2012*** | ***892.3878*** |  |  |  |  |  |  |  |
|  | [444](http://df3/mascot/cgi/peptide_view.pl?file=../data/20121119/F002569.dat&query=444&hit=1&index=&px=1) | ***447.8175*** | ***893.6205*** |  |  |  |  |  |  |  |
|  | [445](http://df3/mascot/cgi/peptide_view.pl?file=../data/20121119/F002569.dat&query=445&hit=1&index=&px=1) | ***448.1395*** | ***894.2645*** |  |  |  |  |  |  |  |
|  | [446](http://df3/mascot/cgi/peptide_view.pl?file=../data/20121119/F002569.dat&query=446&hit=1&index=&px=1) | ***448.1649*** | ***894.3152*** |  |  |  |  |  |  |  |
|  | [447](http://df3/mascot/cgi/peptide_view.pl?file=../data/20121119/F002569.dat&query=447&hit=1&index=&px=1) | ***448.2264*** | ***894.4383*** |  |  |  |  |  |  |  |
|  | [449](http://df3/mascot/cgi/peptide_view.pl?file=../data/20121119/F002569.dat&query=449&hit=1&index=&px=1) | ***448.2605*** | ***894.5064*** |  |  |  |  |  |  |  |
|  | [450](http://df3/mascot/cgi/peptide_view.pl?file=../data/20121119/F002569.dat&query=450&hit=1&index=&px=1) | ***448.3212*** | ***894.6278*** |  |  |  |  |  |  |  |
|  | [453](http://df3/mascot/cgi/peptide_view.pl?file=../data/20121119/F002569.dat&query=453&hit=1&index=&px=1) | ***449.0361*** | ***896.0576*** |  |  |  |  |  |  |  |
|  | [456](http://df3/mascot/cgi/peptide_view.pl?file=../data/20121119/F002569.dat&query=456&hit=1&index=&px=1) | ***449.1228*** | ***896.2311*** |  |  |  |  |  |  |  |
|  | [458](http://df3/mascot/cgi/peptide_view.pl?file=../data/20121119/F002569.dat&query=458&hit=1&index=&px=1) | ***449.2870*** | ***896.5595*** |  |  |  |  |  |  |  |
|  | [460](http://df3/mascot/cgi/peptide_view.pl?file=../data/20121119/F002569.dat&query=460&hit=1&index=&px=1) | ***449.9015*** | ***897.7885*** |  |  |  |  |  |  |  |
|  | [461](http://df3/mascot/cgi/peptide_view.pl?file=../data/20121119/F002569.dat&query=461&hit=1&index=&px=1) | ***450.1836*** | ***898.3526*** |  |  |  |  |  |  |  |
|  | [462](http://df3/mascot/cgi/peptide_view.pl?file=../data/20121119/F002569.dat&query=462&hit=1&index=&px=1) | ***450.2777*** | ***898.5408*** |  |  |  |  |  |  |  |
|  | [463](http://df3/mascot/cgi/peptide_view.pl?file=../data/20121119/F002569.dat&query=463&hit=1&index=&px=1) | ***450.3060*** | ***898.5974*** |  |  |  |  |  |  |  |
|  | [464](http://df3/mascot/cgi/peptide_view.pl?file=../data/20121119/F002569.dat&query=464&hit=1&index=&px=1) | ***450.3127*** | ***898.6109*** |  |  |  |  |  |  |  |
|  | [465](http://df3/mascot/cgi/peptide_view.pl?file=../data/20121119/F002569.dat&query=465&hit=1&index=&px=1) | ***450.4705*** | ***898.9265*** |  |  |  |  |  |  |  |
|  | [466](http://df3/mascot/cgi/peptide_view.pl?file=../data/20121119/F002569.dat&query=466&hit=1&index=&px=1) | ***451.4024*** | ***900.7902*** |  |  |  |  |  |  |  |
|  | [467](http://df3/mascot/cgi/peptide_view.pl?file=../data/20121119/F002569.dat&query=467&hit=1&index=&px=1) | ***451.4063*** | ***900.7979*** |  |  |  |  |  |  |  |
|  | [468](http://df3/mascot/cgi/peptide_view.pl?file=../data/20121119/F002569.dat&query=468&hit=1&index=&px=1) | ***451.4102*** | ***900.8058*** |  |  |  |  |  |  |  |
|  | [469](http://df3/mascot/cgi/peptide_view.pl?file=../data/20121119/F002569.dat&query=469&hit=1&index=&px=1) | ***451.4155*** | ***900.8164*** |  |  |  |  |  |  |  |
|  | [470](http://df3/mascot/cgi/peptide_view.pl?file=../data/20121119/F002569.dat&query=470&hit=1&index=&px=1) | ***451.4934*** | ***900.9722*** |  |  |  |  |  |  |  |
|  | [471](http://df3/mascot/cgi/peptide_view.pl?file=../data/20121119/F002569.dat&query=471&hit=1&index=&px=1) | ***451.8468*** | ***901.6791*** |  |  |  |  |  |  |  |
|  | [472](http://df3/mascot/cgi/peptide_view.pl?file=../data/20121119/F002569.dat&query=472&hit=1&index=&px=1) | ***453.3179*** | ***904.6212*** |  |  |  |  |  |  |  |
|  | [473](http://df3/mascot/cgi/peptide_view.pl?file=../data/20121119/F002569.dat&query=473&hit=1&index=&px=1) | ***453.5339*** | ***905.0533*** |  |  |  |  |  |  |  |
|  | [474](http://df3/mascot/cgi/peptide_view.pl?file=../data/20121119/F002569.dat&query=474&hit=1&index=&px=1) | ***454.0091*** | ***906.0036*** |  |  |  |  |  |  |  |
|  | [475](http://df3/mascot/cgi/peptide_view.pl?file=../data/20121119/F002569.dat&query=475&hit=1&index=&px=1) | ***454.5895*** | ***907.1645*** |  |  |  |  |  |  |  |
|  | [476](http://df3/mascot/cgi/peptide_view.pl?file=../data/20121119/F002569.dat&query=476&hit=1&index=&px=1) | ***454.6750*** | ***907.3355*** |  |  |  |  |  |  |  |
|  | [481](http://df3/mascot/cgi/peptide_view.pl?file=../data/20121119/F002569.dat&query=481&hit=1&index=&px=1) | ***456.4578*** | ***910.9011*** |  |  |  |  |  |  |  |
|  | [482](http://df3/mascot/cgi/peptide_view.pl?file=../data/20121119/F002569.dat&query=482&hit=1&index=&px=1) | ***456.4960*** | ***910.9775*** |  |  |  |  |  |  |  |
|  | [483](http://df3/mascot/cgi/peptide_view.pl?file=../data/20121119/F002569.dat&query=483&hit=1&index=&px=1) | ***456.9985*** | ***911.9824*** |  |  |  |  |  |  |  |
|  | [484](http://df3/mascot/cgi/peptide_view.pl?file=../data/20121119/F002569.dat&query=484&hit=1&index=&px=1) | ***457.3908*** | ***912.7670*** |  |  |  |  |  |  |  |
|  | [486](http://df3/mascot/cgi/peptide_view.pl?file=../data/20121119/F002569.dat&query=486&hit=1&index=&px=1) | ***458.1809*** | ***914.3473*** |  |  |  |  |  |  |  |
|  | [487](http://df3/mascot/cgi/peptide_view.pl?file=../data/20121119/F002569.dat&query=487&hit=1&index=&px=1) | ***458.2980*** | ***914.5815*** |  |  |  |  |  |  |  |
|  | [492](http://df3/mascot/cgi/peptide_view.pl?file=../data/20121119/F002569.dat&query=492&hit=1&index=&px=1) | ***458.7518*** | ***915.4891*** |  |  |  |  |  |  |  |
|  | [493](http://df3/mascot/cgi/peptide_view.pl?file=../data/20121119/F002569.dat&query=493&hit=1&index=&px=1) | ***458.7752*** | ***915.5358*** |  |  |  |  |  |  |  |
|  | [494](http://df3/mascot/cgi/peptide_view.pl?file=../data/20121119/F002569.dat&query=494&hit=1&index=&px=1) | ***458.8998*** | ***915.7851*** |  |  |  |  |  |  |  |
|  | [495](http://df3/mascot/cgi/peptide_view.pl?file=../data/20121119/F002569.dat&query=495&hit=1&index=&px=1) | ***458.9178*** | ***915.8210*** |  |  |  |  |  |  |  |
|  | [496](http://df3/mascot/cgi/peptide_view.pl?file=../data/20121119/F002569.dat&query=496&hit=1&index=&px=1) | ***458.9342*** | ***915.8539*** |  |  |  |  |  |  |  |
|  | [497](http://df3/mascot/cgi/peptide_view.pl?file=../data/20121119/F002569.dat&query=497&hit=1&index=&px=1) | ***459.2349*** | ***916.4553*** |  |  |  |  |  |  |  |
|  | [498](http://df3/mascot/cgi/peptide_view.pl?file=../data/20121119/F002569.dat&query=498&hit=1&index=&px=1) | ***459.2760*** | ***916.5375*** |  |  |  |  |  |  |  |
|  | [499](http://df3/mascot/cgi/peptide_view.pl?file=../data/20121119/F002569.dat&query=499&hit=1&index=&px=1) | ***459.5745*** | ***917.1345*** |  |  |  |  |  |  |  |
|  | [500](http://df3/mascot/cgi/peptide_view.pl?file=../data/20121119/F002569.dat&query=500&hit=1&index=&px=1) | ***459.7205*** | ***917.4265*** |  |  |  |  |  |  |  |
|  | [501](http://df3/mascot/cgi/peptide_view.pl?file=../data/20121119/F002569.dat&query=501&hit=1&index=&px=1) | ***459.9665*** | ***917.9184*** |  |  |  |  |  |  |  |
|  | [502](http://df3/mascot/cgi/peptide_view.pl?file=../data/20121119/F002569.dat&query=502&hit=1&index=&px=1) | ***460.0012*** | ***917.9879*** |  |  |  |  |  |  |  |
|  | [503](http://df3/mascot/cgi/peptide_view.pl?file=../data/20121119/F002569.dat&query=503&hit=1&index=&px=1) | ***460.2068*** | ***918.3990*** |  |  |  |  |  |  |  |
|  | [504](http://df3/mascot/cgi/peptide_view.pl?file=../data/20121119/F002569.dat&query=504&hit=1&index=&px=1) | ***460.2188*** | ***918.4231*** |  |  |  |  |  |  |  |
|  | [505](http://df3/mascot/cgi/peptide_view.pl?file=../data/20121119/F002569.dat&query=505&hit=1&index=&px=1) | ***460.5865*** | ***919.1585*** |  |  |  |  |  |  |  |
|  | [506](http://df3/mascot/cgi/peptide_view.pl?file=../data/20121119/F002569.dat&query=506&hit=1&index=&px=1) | ***460.8274*** | ***919.6403*** |  |  |  |  |  |  |  |
|  | [507](http://df3/mascot/cgi/peptide_view.pl?file=../data/20121119/F002569.dat&query=507&hit=1&index=&px=1) | ***460.9024*** | ***919.7903*** |  |  |  |  |  |  |  |
|  | [508](http://df3/mascot/cgi/peptide_view.pl?file=../data/20121119/F002569.dat&query=508&hit=1&index=&px=1) | ***461.5439*** | ***921.0733*** |  |  |  |  |  |  |  |
|  | [509](http://df3/mascot/cgi/peptide_view.pl?file=../data/20121119/F002569.dat&query=509&hit=1&index=&px=1) | ***461.5998*** | ***921.1850*** |  |  |  |  |  |  |  |
|  | [510](http://df3/mascot/cgi/peptide_view.pl?file=../data/20121119/F002569.dat&query=510&hit=1&index=&px=1) | ***461.7128*** | ***921.4110*** |  |  |  |  |  |  |  |
|  | [511](http://df3/mascot/cgi/peptide_view.pl?file=../data/20121119/F002569.dat&query=511&hit=1&index=&px=1) | ***461.7341*** | ***921.4536*** |  |  |  |  |  |  |  |
|  | [512](http://df3/mascot/cgi/peptide_view.pl?file=../data/20121119/F002569.dat&query=512&hit=1&index=&px=1) | ***461.7538*** | ***921.4931*** |  |  |  |  |  |  |  |
|  | [513](http://df3/mascot/cgi/peptide_view.pl?file=../data/20121119/F002569.dat&query=513&hit=1&index=&px=1) | ***461.7764*** | ***921.5382*** |  |  |  |  |  |  |  |
|  | [515](http://df3/mascot/cgi/peptide_view.pl?file=../data/20121119/F002569.dat&query=515&hit=1&index=&px=1) | ***461.8053*** | ***921.5960*** |  |  |  |  |  |  |  |
|  | [516](http://df3/mascot/cgi/peptide_view.pl?file=../data/20121119/F002569.dat&query=516&hit=1&index=&px=1) | ***461.8116*** | ***921.6086*** |  |  |  |  |  |  |  |
|  | [517](http://df3/mascot/cgi/peptide_view.pl?file=../data/20121119/F002569.dat&query=517&hit=1&index=&px=1) | ***461.8234*** | ***921.6322*** |  |  |  |  |  |  |  |
|  | [518](http://df3/mascot/cgi/peptide_view.pl?file=../data/20121119/F002569.dat&query=518&hit=1&index=&px=1) | ***461.8748*** | ***921.7350*** |  |  |  |  |  |  |  |
|  | [519](http://df3/mascot/cgi/peptide_view.pl?file=../data/20121119/F002569.dat&query=519&hit=1&index=&px=1) | ***461.8824*** | ***921.7503*** |  |  |  |  |  |  |  |
|  | [520](http://df3/mascot/cgi/peptide_view.pl?file=../data/20121119/F002569.dat&query=520&hit=1&index=&px=1) | ***461.8876*** | ***921.7606*** |  |  |  |  |  |  |  |
|  | [521](http://df3/mascot/cgi/peptide_view.pl?file=../data/20121119/F002569.dat&query=521&hit=1&index=&px=1) | ***461.9269*** | ***921.8393*** |  |  |  |  |  |  |  |
|  | [522](http://df3/mascot/cgi/peptide_view.pl?file=../data/20121119/F002569.dat&query=522&hit=1&index=&px=1) | ***461.9396*** | ***921.8647*** |  |  |  |  |  |  |  |
|  | [523](http://df3/mascot/cgi/peptide_view.pl?file=../data/20121119/F002569.dat&query=523&hit=1&index=&px=1) | ***461.9437*** | ***921.8729*** |  |  |  |  |  |  |  |
|  | [524](http://df3/mascot/cgi/peptide_view.pl?file=../data/20121119/F002569.dat&query=524&hit=1&index=&px=1) | ***461.9956*** | ***921.9767*** |  |  |  |  |  |  |  |
|  | [525](http://df3/mascot/cgi/peptide_view.pl?file=../data/20121119/F002569.dat&query=525&hit=1&index=&px=1) | ***462.0013*** | ***921.9879*** |  |  |  |  |  |  |  |
|  | [526](http://df3/mascot/cgi/peptide_view.pl?file=../data/20121119/F002569.dat&query=526&hit=1&index=&px=1) | ***462.0164*** | ***922.0182*** |  |  |  |  |  |  |  |
|  | [527](http://df3/mascot/cgi/peptide_view.pl?file=../data/20121119/F002569.dat&query=527&hit=1&index=&px=1) | ***463.0228*** | ***924.0311*** |  |  |  |  |  |  |  |
|  | [528](http://df3/mascot/cgi/peptide_view.pl?file=../data/20121119/F002569.dat&query=528&hit=1&index=&px=1) | ***463.0530*** | ***924.0915*** |  |  |  |  |  |  |  |
|  | [529](http://df3/mascot/cgi/peptide_view.pl?file=../data/20121119/F002569.dat&query=529&hit=1&index=&px=1) | ***463.0996*** | ***924.1846*** |  |  |  |  |  |  |  |
|  | [530](http://df3/mascot/cgi/peptide_view.pl?file=../data/20121119/F002569.dat&query=530&hit=1&index=&px=1) | ***463.1256*** | ***924.2367*** |  |  |  |  |  |  |  |
|  | [531](http://df3/mascot/cgi/peptide_view.pl?file=../data/20121119/F002569.dat&query=531&hit=1&index=&px=1) | ***463.1651*** | ***924.3156*** |  |  |  |  |  |  |  |
|  | [532](http://df3/mascot/cgi/peptide_view.pl?file=../data/20121119/F002569.dat&query=532&hit=1&index=&px=1) | ***463.5767*** | ***925.1388*** |  |  |  |  |  |  |  |
|  | [533](http://df3/mascot/cgi/peptide_view.pl?file=../data/20121119/F002569.dat&query=533&hit=1&index=&px=1) | ***463.5779*** | ***925.1413*** |  |  |  |  |  |  |  |
|  | [534](http://df3/mascot/cgi/peptide_view.pl?file=../data/20121119/F002569.dat&query=534&hit=1&index=&px=1) | ***463.5785*** | ***925.1425*** |  |  |  |  |  |  |  |
|  | [535](http://df3/mascot/cgi/peptide_view.pl?file=../data/20121119/F002569.dat&query=535&hit=1&index=&px=1) | ***463.5891*** | ***925.1636*** |  |  |  |  |  |  |  |
|  | [536](http://df3/mascot/cgi/peptide_view.pl?file=../data/20121119/F002569.dat&query=536&hit=1&index=&px=1) | ***463.6545*** | ***925.2945*** |  |  |  |  |  |  |  |
|  | [537](http://df3/mascot/cgi/peptide_view.pl?file=../data/20121119/F002569.dat&query=537&hit=1&index=&px=1) | ***463.6696*** | ***925.3246*** |  |  |  |  |  |  |  |
|  | [538](http://df3/mascot/cgi/peptide_view.pl?file=../data/20121119/F002569.dat&query=538&hit=1&index=&px=1) | ***463.6767*** | ***925.3388*** |  |  |  |  |  |  |  |
|  | [539](http://df3/mascot/cgi/peptide_view.pl?file=../data/20121119/F002569.dat&query=539&hit=1&index=&px=1) | ***463.6768*** | ***925.3390*** |  |  |  |  |  |  |  |
|  | [540](http://df3/mascot/cgi/peptide_view.pl?file=../data/20121119/F002569.dat&query=540&hit=1&index=&px=1) | ***463.6947*** | ***925.3749*** |  |  |  |  |  |  |  |
|  | [541](http://df3/mascot/cgi/peptide_view.pl?file=../data/20121119/F002569.dat&query=541&hit=1&index=&px=1) | ***463.8084*** | ***925.6021*** |  |  |  |  |  |  |  |
|  | [542](http://df3/mascot/cgi/peptide_view.pl?file=../data/20121119/F002569.dat&query=542&hit=1&index=&px=1) | ***463.8175*** | ***925.6204*** |  |  |  |  |  |  |  |
|  | [543](http://df3/mascot/cgi/peptide_view.pl?file=../data/20121119/F002569.dat&query=543&hit=1&index=&px=1) | ***463.9307*** | ***925.8469*** |  |  |  |  |  |  |  |
|  | [544](http://df3/mascot/cgi/peptide_view.pl?file=../data/20121119/F002569.dat&query=544&hit=1&index=&px=1) | ***463.9352*** | ***925.8558*** |  |  |  |  |  |  |  |
|  | [545](http://df3/mascot/cgi/peptide_view.pl?file=../data/20121119/F002569.dat&query=545&hit=1&index=&px=1) | ***463.9426*** | ***925.8707*** |  |  |  |  |  |  |  |
|  | [546](http://df3/mascot/cgi/peptide_view.pl?file=../data/20121119/F002569.dat&query=546&hit=1&index=&px=1) | ***463.9535*** | ***925.8924*** |  |  |  |  |  |  |  |
|  | [547](http://df3/mascot/cgi/peptide_view.pl?file=../data/20121119/F002569.dat&query=547&hit=1&index=&px=1) | ***463.9590*** | ***925.9034*** |  |  |  |  |  |  |  |
|  | [548](http://df3/mascot/cgi/peptide_view.pl?file=../data/20121119/F002569.dat&query=548&hit=1&index=&px=1) | ***463.9670*** | ***925.9194*** |  |  |  |  |  |  |  |
|  | [549](http://df3/mascot/cgi/peptide_view.pl?file=../data/20121119/F002569.dat&query=549&hit=1&index=&px=1) | ***463.9805*** | ***925.9464*** |  |  |  |  |  |  |  |
|  | [550](http://df3/mascot/cgi/peptide_view.pl?file=../data/20121119/F002569.dat&query=550&hit=1&index=&px=1) | ***463.9894*** | ***925.9643*** |  |  |  |  |  |  |  |
|  | [551](http://df3/mascot/cgi/peptide_view.pl?file=../data/20121119/F002569.dat&query=551&hit=1&index=&px=1) | ***464.0013*** | ***925.9879*** |  |  |  |  |  |  |  |
|  | [552](http://df3/mascot/cgi/peptide_view.pl?file=../data/20121119/F002569.dat&query=552&hit=1&index=&px=1) | ***464.0026*** | ***925.9906*** |  |  |  |  |  |  |  |
|  | [553](http://df3/mascot/cgi/peptide_view.pl?file=../data/20121119/F002569.dat&query=553&hit=1&index=&px=1) | ***464.0225*** | ***926.0304*** |  |  |  |  |  |  |  |
|  | [554](http://df3/mascot/cgi/peptide_view.pl?file=../data/20121119/F002569.dat&query=554&hit=1&index=&px=1) | ***464.0342*** | ***926.0539*** |  |  |  |  |  |  |  |
|  | [555](http://df3/mascot/cgi/peptide_view.pl?file=../data/20121119/F002569.dat&query=555&hit=1&index=&px=1) | ***464.0643*** | ***926.1140*** |  |  |  |  |  |  |  |
|  | [556](http://df3/mascot/cgi/peptide_view.pl?file=../data/20121119/F002569.dat&query=556&hit=1&index=&px=1) | ***464.1296*** | ***926.2447*** |  |  |  |  |  |  |  |
|  | [557](http://df3/mascot/cgi/peptide_view.pl?file=../data/20121119/F002569.dat&query=557&hit=1&index=&px=1) | ***464.9548*** | ***927.8951*** |  |  |  |  |  |  |  |
|  | [558](http://df3/mascot/cgi/peptide_view.pl?file=../data/20121119/F002569.dat&query=558&hit=1&index=&px=1) | ***464.9873*** | ***927.9601*** |  |  |  |  |  |  |  |
|  | [559](http://df3/mascot/cgi/peptide_view.pl?file=../data/20121119/F002569.dat&query=559&hit=1&index=&px=1) | ***464.9894*** | ***927.9643*** |  |  |  |  |  |  |  |
|  | [560](http://df3/mascot/cgi/peptide_view.pl?file=../data/20121119/F002569.dat&query=560&hit=1&index=&px=1) | ***464.9978*** | ***927.9811*** |  |  |  |  |  |  |  |
|  | [561](http://df3/mascot/cgi/peptide_view.pl?file=../data/20121119/F002569.dat&query=561&hit=1&index=&px=1) | ***465.0039*** | ***927.9933*** |  |  |  |  |  |  |  |
|  | [562](http://df3/mascot/cgi/peptide_view.pl?file=../data/20121119/F002569.dat&query=562&hit=1&index=&px=1) | ***465.0045*** | ***927.9944*** |  |  |  |  |  |  |  |
|  | [563](http://df3/mascot/cgi/peptide_view.pl?file=../data/20121119/F002569.dat&query=563&hit=1&index=&px=1) | ***465.0109*** | ***928.0072*** |  |  |  |  |  |  |  |
|  | [564](http://df3/mascot/cgi/peptide_view.pl?file=../data/20121119/F002569.dat&query=564&hit=1&index=&px=1) | ***465.0138*** | ***928.0130*** |  |  |  |  |  |  |  |
|  | [565](http://df3/mascot/cgi/peptide_view.pl?file=../data/20121119/F002569.dat&query=565&hit=1&index=&px=1) | ***465.0145*** | ***928.0145*** |  |  |  |  |  |  |  |
|  | [566](http://df3/mascot/cgi/peptide_view.pl?file=../data/20121119/F002569.dat&query=566&hit=1&index=&px=1) | ***465.0208*** | ***928.0270*** |  |  |  |  |  |  |  |
|  | [567](http://df3/mascot/cgi/peptide_view.pl?file=../data/20121119/F002569.dat&query=567&hit=1&index=&px=1) | ***465.0269*** | ***928.0392*** |  |  |  |  |  |  |  |
|  | [568](http://df3/mascot/cgi/peptide_view.pl?file=../data/20121119/F002569.dat&query=568&hit=1&index=&px=1) | ***465.1067*** | ***928.1988*** |  |  |  |  |  |  |  |
|  | [569](http://df3/mascot/cgi/peptide_view.pl?file=../data/20121119/F002569.dat&query=569&hit=1&index=&px=1) | ***465.1214*** | ***928.2282*** |  |  |  |  |  |  |  |
|  | [570](http://df3/mascot/cgi/peptide_view.pl?file=../data/20121119/F002569.dat&query=570&hit=1&index=&px=1) | ***465.1252*** | ***928.2359*** |  |  |  |  |  |  |  |
|  | [571](http://df3/mascot/cgi/peptide_view.pl?file=../data/20121119/F002569.dat&query=571&hit=1&index=&px=1) | ***465.1375*** | ***928.2604*** |  |  |  |  |  |  |  |
|  | [572](http://df3/mascot/cgi/peptide_view.pl?file=../data/20121119/F002569.dat&query=572&hit=1&index=&px=1) | ***465.1919*** | ***928.3692*** |  |  |  |  |  |  |  |
|  | [573](http://df3/mascot/cgi/peptide_view.pl?file=../data/20121119/F002569.dat&query=573&hit=1&index=&px=1) | ***465.6596*** | ***929.3047*** |  |  |  |  |  |  |  |
|  | [574](http://df3/mascot/cgi/peptide_view.pl?file=../data/20121119/F002569.dat&query=574&hit=1&index=&px=1) | ***465.8108*** | ***929.6070*** |  |  |  |  |  |  |  |
|  | [575](http://df3/mascot/cgi/peptide_view.pl?file=../data/20121119/F002569.dat&query=575&hit=1&index=&px=1) | ***465.8127*** | ***929.6108*** |  |  |  |  |  |  |  |
|  | [576](http://df3/mascot/cgi/peptide_view.pl?file=../data/20121119/F002569.dat&query=576&hit=1&index=&px=1) | ***465.8190*** | ***929.6234*** |  |  |  |  |  |  |  |
|  | [577](http://df3/mascot/cgi/peptide_view.pl?file=../data/20121119/F002569.dat&query=577&hit=1&index=&px=1) | ***465.8681*** | ***929.7216*** |  |  |  |  |  |  |  |
|  | [578](http://df3/mascot/cgi/peptide_view.pl?file=../data/20121119/F002569.dat&query=578&hit=1&index=&px=1) | ***465.9297*** | ***929.8448*** |  |  |  |  |  |  |  |
|  | [579](http://df3/mascot/cgi/peptide_view.pl?file=../data/20121119/F002569.dat&query=579&hit=1&index=&px=1) | ***465.9964*** | ***929.9782*** |  |  |  |  |  |  |  |
|  | [580](http://df3/mascot/cgi/peptide_view.pl?file=../data/20121119/F002569.dat&query=580&hit=1&index=&px=1) | ***466.5282*** | ***931.0418*** |  |  |  |  |  |  |  |
|  | [581](http://df3/mascot/cgi/peptide_view.pl?file=../data/20121119/F002569.dat&query=581&hit=1&index=&px=1) | ***467.2186*** | ***932.4227*** |  |  |  |  |  |  |  |
|  | [582](http://df3/mascot/cgi/peptide_view.pl?file=../data/20121119/F002569.dat&query=582&hit=1&index=&px=1) | ***467.2627*** | ***932.5108*** |  |  |  |  |  |  |  |
|  | [583](http://df3/mascot/cgi/peptide_view.pl?file=../data/20121119/F002569.dat&query=583&hit=1&index=&px=1) | ***467.3490*** | ***932.6834*** |  |  |  |  |  |  |  |
|  | [586](http://df3/mascot/cgi/peptide_view.pl?file=../data/20121119/F002569.dat&query=586&hit=1&index=&px=1) | ***467.4194*** | ***932.8243*** |  |  |  |  |  |  |  |
|  | [587](http://df3/mascot/cgi/peptide_view.pl?file=../data/20121119/F002569.dat&query=587&hit=1&index=&px=1) | ***467.4258*** | ***932.8370*** |  |  |  |  |  |  |  |
|  | [588](http://df3/mascot/cgi/peptide_view.pl?file=../data/20121119/F002569.dat&query=588&hit=1&index=&px=1) | ***467.5113*** | ***933.0080*** |  |  |  |  |  |  |  |
|  | [590](http://df3/mascot/cgi/peptide_view.pl?file=../data/20121119/F002569.dat&query=590&hit=1&index=&px=1) | ***467.8993*** | ***933.7840*** |  |  |  |  |  |  |  |
|  | [591](http://df3/mascot/cgi/peptide_view.pl?file=../data/20121119/F002569.dat&query=591&hit=1&index=&px=1) | ***467.9144*** | ***933.8143*** |  |  |  |  |  |  |  |
|  | [592](http://df3/mascot/cgi/peptide_view.pl?file=../data/20121119/F002569.dat&query=592&hit=1&index=&px=1) | ***467.9160*** | ***933.8174*** |  |  |  |  |  |  |  |
|  | [594](http://df3/mascot/cgi/peptide_view.pl?file=../data/20121119/F002569.dat&query=594&hit=1&index=&px=1) | ***467.9336*** | ***933.8526*** |  |  |  |  |  |  |  |
|  | [596](http://df3/mascot/cgi/peptide_view.pl?file=../data/20121119/F002569.dat&query=596&hit=1&index=&px=1) | ***467.9803*** | ***933.9460*** |  |  |  |  |  |  |  |
|  | [597](http://df3/mascot/cgi/peptide_view.pl?file=../data/20121119/F002569.dat&query=597&hit=1&index=&px=1) | ***468.0386*** | ***934.0626*** |  |  |  |  |  |  |  |
|  | [598](http://df3/mascot/cgi/peptide_view.pl?file=../data/20121119/F002569.dat&query=598&hit=1&index=&px=1) | ***468.9406*** | ***935.8666*** |  |  |  |  |  |  |  |
|  | [599](http://df3/mascot/cgi/peptide_view.pl?file=../data/20121119/F002569.dat&query=599&hit=1&index=&px=1) | ***469.0518*** | ***936.0890*** |  |  |  |  |  |  |  |
|  | [600](http://df3/mascot/cgi/peptide_view.pl?file=../data/20121119/F002569.dat&query=600&hit=1&index=&px=1) | ***469.0548*** | ***936.0951*** |  |  |  |  |  |  |  |
|  | [601](http://df3/mascot/cgi/peptide_view.pl?file=../data/20121119/F002569.dat&query=601&hit=1&index=&px=1) | ***469.1097*** | ***936.2049*** |  |  |  |  |  |  |  |
|  | [602](http://df3/mascot/cgi/peptide_view.pl?file=../data/20121119/F002569.dat&query=602&hit=1&index=&px=1) | ***469.1300*** | ***936.2455*** |  |  |  |  |  |  |  |
|  | [603](http://df3/mascot/cgi/peptide_view.pl?file=../data/20121119/F002569.dat&query=603&hit=1&index=&px=1) | ***469.6205*** | ***937.2265*** |  |  |  |  |  |  |  |
|  | [604](http://df3/mascot/cgi/peptide_view.pl?file=../data/20121119/F002569.dat&query=604&hit=1&index=&px=1) | ***470.0677*** | ***938.1208*** |  |  |  |  |  |  |  |
|  | [605](http://df3/mascot/cgi/peptide_view.pl?file=../data/20121119/F002569.dat&query=605&hit=1&index=&px=1) | ***470.1954*** | ***938.3763*** |  |  |  |  |  |  |  |
|  | [606](http://df3/mascot/cgi/peptide_view.pl?file=../data/20121119/F002569.dat&query=606&hit=1&index=&px=1) | ***470.6642*** | ***939.3138*** |  |  |  |  |  |  |  |
|  | [607](http://df3/mascot/cgi/peptide_view.pl?file=../data/20121119/F002569.dat&query=607&hit=1&index=&px=1) | ***471.4745*** | ***940.9344*** |  |  |  |  |  |  |  |
|  | [608](http://df3/mascot/cgi/peptide_view.pl?file=../data/20121119/F002569.dat&query=608&hit=1&index=&px=1) | ***471.9685*** | ***941.9225*** |  |  |  |  |  |  |  |
|  | [609](http://df3/mascot/cgi/peptide_view.pl?file=../data/20121119/F002569.dat&query=609&hit=1&index=&px=1) | ***472.0023*** | ***941.9901*** |  |  |  |  |  |  |  |
|  | [610](http://df3/mascot/cgi/peptide_view.pl?file=../data/20121119/F002569.dat&query=610&hit=1&index=&px=1) | ***472.1630*** | ***942.3115*** |  |  |  |  |  |  |  |
|  | [611](http://df3/mascot/cgi/peptide_view.pl?file=../data/20121119/F002569.dat&query=611&hit=1&index=&px=1) | ***472.4526*** | ***942.8907*** |  |  |  |  |  |  |  |
|  | [612](http://df3/mascot/cgi/peptide_view.pl?file=../data/20121119/F002569.dat&query=612&hit=1&index=&px=1) | ***472.9332*** | ***943.8518*** |  |  |  |  |  |  |  |
|  | [613](http://df3/mascot/cgi/peptide_view.pl?file=../data/20121119/F002569.dat&query=613&hit=1&index=&px=1) | ***473.0577*** | ***944.1008*** |  |  |  |  |  |  |  |
|  | [614](http://df3/mascot/cgi/peptide_view.pl?file=../data/20121119/F002569.dat&query=614&hit=1&index=&px=1) | ***473.2029*** | ***944.3912*** |  |  |  |  |  |  |  |
|  | [615](http://df3/mascot/cgi/peptide_view.pl?file=../data/20121119/F002569.dat&query=615&hit=1&index=&px=1) | ***473.2066*** | ***944.3987*** |  |  |  |  |  |  |  |
|  | [616](http://df3/mascot/cgi/peptide_view.pl?file=../data/20121119/F002569.dat&query=616&hit=1&index=&px=1) | ***473.2126*** | ***944.4107*** |  |  |  |  |  |  |  |
|  | [617](http://df3/mascot/cgi/peptide_view.pl?file=../data/20121119/F002569.dat&query=617&hit=1&index=&px=1) | ***473.3660*** | ***944.7174*** |  |  |  |  |  |  |  |
|  | [618](http://df3/mascot/cgi/peptide_view.pl?file=../data/20121119/F002569.dat&query=618&hit=1&index=&px=1) | ***473.3810*** | ***944.7474*** |  |  |  |  |  |  |  |
|  | [619](http://df3/mascot/cgi/peptide_view.pl?file=../data/20121119/F002569.dat&query=619&hit=1&index=&px=1) | ***473.4154*** | ***944.8163*** |  |  |  |  |  |  |  |
|  | [620](http://df3/mascot/cgi/peptide_view.pl?file=../data/20121119/F002569.dat&query=620&hit=1&index=&px=1) | ***473.4606*** | ***944.9066*** |  |  |  |  |  |  |  |
|  | [621](http://df3/mascot/cgi/peptide_view.pl?file=../data/20121119/F002569.dat&query=621&hit=1&index=&px=1) | ***473.4716*** | ***944.9287*** |  |  |  |  |  |  |  |
|  | [622](http://df3/mascot/cgi/peptide_view.pl?file=../data/20121119/F002569.dat&query=622&hit=1&index=&px=1) | ***473.5812*** | ***945.1478*** |  |  |  |  |  |  |  |
|  | [623](http://df3/mascot/cgi/peptide_view.pl?file=../data/20121119/F002569.dat&query=623&hit=1&index=&px=1) | ***473.5903*** | ***945.1661*** |  |  |  |  |  |  |  |
|  | [624](http://df3/mascot/cgi/peptide_view.pl?file=../data/20121119/F002569.dat&query=624&hit=1&index=&px=1) | ***473.6001*** | ***945.1856*** |  |  |  |  |  |  |  |
|  | [625](http://df3/mascot/cgi/peptide_view.pl?file=../data/20121119/F002569.dat&query=625&hit=1&index=&px=1) | ***473.6492*** | ***945.2838*** |  |  |  |  |  |  |  |
|  | [626](http://df3/mascot/cgi/peptide_view.pl?file=../data/20121119/F002569.dat&query=626&hit=1&index=&px=1) | ***473.7563*** | ***945.4980*** |  |  |  |  |  |  |  |
|  | [627](http://df3/mascot/cgi/peptide_view.pl?file=../data/20121119/F002569.dat&query=627&hit=1&index=&px=1) | ***474.5662*** | ***947.1178*** |  |  |  |  |  |  |  |
|  | [628](http://df3/mascot/cgi/peptide_view.pl?file=../data/20121119/F002569.dat&query=628&hit=1&index=&px=1) | ***474.6505*** | ***947.2865*** |  |  |  |  |  |  |  |
|  | [629](http://df3/mascot/cgi/peptide_view.pl?file=../data/20121119/F002569.dat&query=629&hit=1&index=&px=1) | ***475.2447*** | ***948.4748*** |  |  |  |  |  |  |  |
|  | [630](http://df3/mascot/cgi/peptide_view.pl?file=../data/20121119/F002569.dat&query=630&hit=1&index=&px=1) | ***476.5562*** | ***951.0978*** |  |  |  |  |  |  |  |
|  | [631](http://df3/mascot/cgi/peptide_view.pl?file=../data/20121119/F002569.dat&query=631&hit=1&index=&px=1) | ***476.8702*** | ***951.7259*** |  |  |  |  |  |  |  |
|  | [632](http://df3/mascot/cgi/peptide_view.pl?file=../data/20121119/F002569.dat&query=632&hit=1&index=&px=1) | ***477.4228*** | ***952.8311*** |  |  |  |  |  |  |  |
|  | [633](http://df3/mascot/cgi/peptide_view.pl?file=../data/20121119/F002569.dat&query=633&hit=1&index=&px=1) | ***477.9321*** | ***953.8496*** |  |  |  |  |  |  |  |
|  | [634](http://df3/mascot/cgi/peptide_view.pl?file=../data/20121119/F002569.dat&query=634&hit=1&index=&px=1) | ***477.9819*** | ***953.9493*** |  |  |  |  |  |  |  |
|  | [635](http://df3/mascot/cgi/peptide_view.pl?file=../data/20121119/F002569.dat&query=635&hit=1&index=&px=1) | ***478.0070*** | ***953.9994*** |  |  |  |  |  |  |  |
|  | [636](http://df3/mascot/cgi/peptide_view.pl?file=../data/20121119/F002569.dat&query=636&hit=1&index=&px=1) | ***478.0226*** | ***954.0306*** |  |  |  |  |  |  |  |
|  | [637](http://df3/mascot/cgi/peptide_view.pl?file=../data/20121119/F002569.dat&query=637&hit=1&index=&px=1) | ***478.0546*** | ***954.0946*** |  |  |  |  |  |  |  |
|  | [638](http://df3/mascot/cgi/peptide_view.pl?file=../data/20121119/F002569.dat&query=638&hit=1&index=&px=1) | ***478.0570*** | ***954.0995*** |  |  |  |  |  |  |  |
|  | [639](http://df3/mascot/cgi/peptide_view.pl?file=../data/20121119/F002569.dat&query=639&hit=1&index=&px=1) | ***478.0712*** | ***954.1278*** |  |  |  |  |  |  |  |
|  | [640](http://df3/mascot/cgi/peptide_view.pl?file=../data/20121119/F002569.dat&query=640&hit=1&index=&px=1) | ***478.1006*** | ***954.1866*** |  |  |  |  |  |  |  |
|  | [641](http://df3/mascot/cgi/peptide_view.pl?file=../data/20121119/F002569.dat&query=641&hit=1&index=&px=1) | ***478.2900*** | ***954.5654*** |  |  |  |  |  |  |  |
|  | [642](http://df3/mascot/cgi/peptide_view.pl?file=../data/20121119/F002569.dat&query=642&hit=1&index=&px=1) | ***478.4579*** | ***954.9012*** |  |  |  |  |  |  |  |
|  | [643](http://df3/mascot/cgi/peptide_view.pl?file=../data/20121119/F002569.dat&query=643&hit=1&index=&px=1) | ***478.7934*** | ***955.5722*** |  |  |  |  |  |  |  |
|  | [644](http://df3/mascot/cgi/peptide_view.pl?file=../data/20121119/F002569.dat&query=644&hit=1&index=&px=1) | ***478.9797*** | ***955.9448*** |  |  |  |  |  |  |  |
|  | [645](http://df3/mascot/cgi/peptide_view.pl?file=../data/20121119/F002569.dat&query=645&hit=1&index=&px=1) | ***479.2479*** | ***956.4812*** |  |  |  |  |  |  |  |
|  | [646](http://df3/mascot/cgi/peptide_view.pl?file=../data/20121119/F002569.dat&query=646&hit=1&index=&px=1) | ***479.6557*** | ***957.2968*** |  |  |  |  |  |  |  |
|  | [647](http://df3/mascot/cgi/peptide_view.pl?file=../data/20121119/F002569.dat&query=647&hit=1&index=&px=1) | ***479.7346*** | ***957.4546*** |  |  |  |  |  |  |  |
|  | [648](http://df3/mascot/cgi/peptide_view.pl?file=../data/20121119/F002569.dat&query=648&hit=1&index=&px=1) | ***479.7463*** | ***957.4780*** |  |  |  |  |  |  |  |
|  | [649](http://df3/mascot/cgi/peptide_view.pl?file=../data/20121119/F002569.dat&query=649&hit=1&index=&px=1) | ***480.3076*** | ***958.6007*** |  |  |  |  |  |  |  |
|  | [650](http://df3/mascot/cgi/peptide_view.pl?file=../data/20121119/F002569.dat&query=650&hit=1&index=&px=1) | ***480.5078*** | ***959.0011*** |  |  |  |  |  |  |  |
|  | [652](http://df3/mascot/cgi/peptide_view.pl?file=../data/20121119/F002569.dat&query=652&hit=1&index=&px=1) | ***481.0321*** | ***960.0497*** |  |  |  |  |  |  |  |
|  | [653](http://df3/mascot/cgi/peptide_view.pl?file=../data/20121119/F002569.dat&query=653&hit=1&index=&px=1) | ***481.2407*** | ***960.4669*** |  |  |  |  |  |  |  |
|  | [654](http://df3/mascot/cgi/peptide_view.pl?file=../data/20121119/F002569.dat&query=654&hit=1&index=&px=1) | ***481.3566*** | ***960.6986*** |  |  |  |  |  |  |  |
|  | [655](http://df3/mascot/cgi/peptide_view.pl?file=../data/20121119/F002569.dat&query=655&hit=1&index=&px=1) | ***481.4087*** | ***960.8028*** |  |  |  |  |  |  |  |
|  | [656](http://df3/mascot/cgi/peptide_view.pl?file=../data/20121119/F002569.dat&query=656&hit=1&index=&px=1) | ***481.5317*** | ***961.0489*** |  |  |  |  |  |  |  |
|  | [657](http://df3/mascot/cgi/peptide_view.pl?file=../data/20121119/F002569.dat&query=657&hit=1&index=&px=1) | ***481.6912*** | ***961.3678*** |  |  |  |  |  |  |  |
|  | [658](http://df3/mascot/cgi/peptide_view.pl?file=../data/20121119/F002569.dat&query=658&hit=1&index=&px=1) | ***481.9406*** | ***961.8667*** |  |  |  |  |  |  |  |
|  | [659](http://df3/mascot/cgi/peptide_view.pl?file=../data/20121119/F002569.dat&query=659&hit=1&index=&px=1) | ***482.3032*** | ***962.5919*** |  |  |  |  |  |  |  |
|  | [660](http://df3/mascot/cgi/peptide_view.pl?file=../data/20121119/F002569.dat&query=660&hit=1&index=&px=1) | ***482.4657*** | ***962.9168*** |  |  |  |  |  |  |  |
|  | [661](http://df3/mascot/cgi/peptide_view.pl?file=../data/20121119/F002569.dat&query=661&hit=1&index=&px=1) | ***482.4903*** | ***962.9660*** |  |  |  |  |  |  |  |
|  | [662](http://df3/mascot/cgi/peptide_view.pl?file=../data/20121119/F002569.dat&query=662&hit=1&index=&px=1) | ***482.5223*** | ***963.0301*** |  |  |  |  |  |  |  |
|  | [663](http://df3/mascot/cgi/peptide_view.pl?file=../data/20121119/F002569.dat&query=663&hit=1&index=&px=1) | ***482.5671*** | ***963.1197*** |  |  |  |  |  |  |  |
|  | [664](http://df3/mascot/cgi/peptide_view.pl?file=../data/20121119/F002569.dat&query=664&hit=1&index=&px=1) | ***482.6079*** | ***963.2013*** |  |  |  |  |  |  |  |
|  | [665](http://df3/mascot/cgi/peptide_view.pl?file=../data/20121119/F002569.dat&query=665&hit=1&index=&px=1) | ***483.3973*** | ***964.7800*** |  |  |  |  |  |  |  |
|  | [666](http://df3/mascot/cgi/peptide_view.pl?file=../data/20121119/F002569.dat&query=666&hit=1&index=&px=1) | ***483.6233*** | ***965.2320*** |  |  |  |  |  |  |  |
|  | [667](http://df3/mascot/cgi/peptide_view.pl?file=../data/20121119/F002569.dat&query=667&hit=1&index=&px=1) | ***483.7418*** | ***965.4690*** |  |  |  |  |  |  |  |
|  | [668](http://df3/mascot/cgi/peptide_view.pl?file=../data/20121119/F002569.dat&query=668&hit=1&index=&px=1) | ***483.7603*** | ***965.5061*** |  |  |  |  |  |  |  |
|  | [669](http://df3/mascot/cgi/peptide_view.pl?file=../data/20121119/F002569.dat&query=669&hit=1&index=&px=1) | ***483.8322*** | ***965.6499*** |  |  |  |  |  |  |  |
|  | [670](http://df3/mascot/cgi/peptide_view.pl?file=../data/20121119/F002569.dat&query=670&hit=1&index=&px=1) | ***483.8953*** | ***965.7761*** |  |  |  |  |  |  |  |
|  | [671](http://df3/mascot/cgi/peptide_view.pl?file=../data/20121119/F002569.dat&query=671&hit=1&index=&px=1) | ***483.8992*** | ***965.7838*** |  |  |  |  |  |  |  |
|  | [672](http://df3/mascot/cgi/peptide_view.pl?file=../data/20121119/F002569.dat&query=672&hit=1&index=&px=1) | ***483.9749*** | ***965.9353*** |  |  |  |  |  |  |  |
|  | [673](http://df3/mascot/cgi/peptide_view.pl?file=../data/20121119/F002569.dat&query=673&hit=1&index=&px=1) | ***484.0041*** | ***965.9937*** |  |  |  |  |  |  |  |
|  | [674](http://df3/mascot/cgi/peptide_view.pl?file=../data/20121119/F002569.dat&query=674&hit=1&index=&px=1) | ***485.3560*** | ***968.6974*** |  |  |  |  |  |  |  |
|  | [675](http://df3/mascot/cgi/peptide_view.pl?file=../data/20121119/F002569.dat&query=675&hit=1&index=&px=1) | ***485.5389*** | ***969.0633*** |  |  |  |  |  |  |  |
|  | [676](http://df3/mascot/cgi/peptide_view.pl?file=../data/20121119/F002569.dat&query=676&hit=1&index=&px=1) | ***485.5616*** | ***969.1087*** |  |  |  |  |  |  |  |
|  | [677](http://df3/mascot/cgi/peptide_view.pl?file=../data/20121119/F002569.dat&query=677&hit=1&index=&px=1) | ***485.5962*** | ***969.1779*** |  |  |  |  |  |  |  |
|  | [678](http://df3/mascot/cgi/peptide_view.pl?file=../data/20121119/F002569.dat&query=678&hit=1&index=&px=1) | ***485.6190*** | ***969.2234*** |  |  |  |  |  |  |  |
|  | [679](http://df3/mascot/cgi/peptide_view.pl?file=../data/20121119/F002569.dat&query=679&hit=1&index=&px=1) | ***485.7474*** | ***969.4803*** |  |  |  |  |  |  |  |
|  | [680](http://df3/mascot/cgi/peptide_view.pl?file=../data/20121119/F002569.dat&query=680&hit=1&index=&px=1) | ***485.7479*** | ***969.4813*** |  |  |  |  |  |  |  |
|  | [681](http://df3/mascot/cgi/peptide_view.pl?file=../data/20121119/F002569.dat&query=681&hit=1&index=&px=1) | ***486.1049*** | ***970.1952*** |  |  |  |  |  |  |  |
|  | [682](http://df3/mascot/cgi/peptide_view.pl?file=../data/20121119/F002569.dat&query=682&hit=1&index=&px=1) | ***486.5264*** | ***971.0382*** |  |  |  |  |  |  |  |
|  | [683](http://df3/mascot/cgi/peptide_view.pl?file=../data/20121119/F002569.dat&query=683&hit=1&index=&px=1) | ***486.8059*** | ***971.5973*** |  |  |  |  |  |  |  |
|  | [684](http://df3/mascot/cgi/peptide_view.pl?file=../data/20121119/F002569.dat&query=684&hit=1&index=&px=1) | ***486.9745*** | ***971.9344*** |  |  |  |  |  |  |  |
|  | [685](http://df3/mascot/cgi/peptide_view.pl?file=../data/20121119/F002569.dat&query=685&hit=1&index=&px=1) | ***487.0002*** | ***971.9859*** |  |  |  |  |  |  |  |
|  | [686](http://df3/mascot/cgi/peptide_view.pl?file=../data/20121119/F002569.dat&query=686&hit=1&index=&px=1) | ***488.0852*** | ***974.1558*** |  |  |  |  |  |  |  |
|  | [687](http://df3/mascot/cgi/peptide_view.pl?file=../data/20121119/F002569.dat&query=687&hit=1&index=&px=1) | ***488.0861*** | ***974.1576*** |  |  |  |  |  |  |  |
|  | [688](http://df3/mascot/cgi/peptide_view.pl?file=../data/20121119/F002569.dat&query=688&hit=1&index=&px=1) | ***488.1487*** | ***974.2829*** |  |  |  |  |  |  |  |
|  | [689](http://df3/mascot/cgi/peptide_view.pl?file=../data/20121119/F002569.dat&query=689&hit=1&index=&px=1) | ***488.1496*** | ***974.2846*** |  |  |  |  |  |  |  |
|  | [690](http://df3/mascot/cgi/peptide_view.pl?file=../data/20121119/F002569.dat&query=690&hit=1&index=&px=1) | ***488.1557*** | ***974.2968*** |  |  |  |  |  |  |  |
|  | [691](http://df3/mascot/cgi/peptide_view.pl?file=../data/20121119/F002569.dat&query=691&hit=1&index=&px=1) | ***488.2295*** | ***974.4444*** |  |  |  |  |  |  |  |
|  | [692](http://df3/mascot/cgi/peptide_view.pl?file=../data/20121119/F002569.dat&query=692&hit=1&index=&px=1) | ***488.2816*** | ***974.5486*** |  |  |  |  |  |  |  |
|  | [693](http://df3/mascot/cgi/peptide_view.pl?file=../data/20121119/F002569.dat&query=693&hit=1&index=&px=1) | ***488.9406*** | ***975.8666*** |  |  |  |  |  |  |  |
|  | [694](http://df3/mascot/cgi/peptide_view.pl?file=../data/20121119/F002569.dat&query=694&hit=1&index=&px=1) | ***489.2533*** | ***976.4921*** |  |  |  |  |  |  |  |
|  | [695](http://df3/mascot/cgi/peptide_view.pl?file=../data/20121119/F002569.dat&query=695&hit=1&index=&px=1) | ***489.2628*** | ***976.5111*** |  |  |  |  |  |  |  |
|  | [696](http://df3/mascot/cgi/peptide_view.pl?file=../data/20121119/F002569.dat&query=696&hit=1&index=&px=1) | ***489.4243*** | ***976.8340*** |  |  |  |  |  |  |  |
|  | [697](http://df3/mascot/cgi/peptide_view.pl?file=../data/20121119/F002569.dat&query=697&hit=1&index=&px=1) | ***489.5338*** | ***977.0531*** |  |  |  |  |  |  |  |
|  | [698](http://df3/mascot/cgi/peptide_view.pl?file=../data/20121119/F002569.dat&query=698&hit=1&index=&px=1) | ***489.9258*** | ***977.8370*** |  |  |  |  |  |  |  |
|  | [699](http://df3/mascot/cgi/peptide_view.pl?file=../data/20121119/F002569.dat&query=699&hit=1&index=&px=1) | ***490.5465*** | ***979.0784*** |  |  |  |  |  |  |  |
|  | [700](http://df3/mascot/cgi/peptide_view.pl?file=../data/20121119/F002569.dat&query=700&hit=1&index=&px=1) | ***490.5490*** | ***979.0834*** |  |  |  |  |  |  |  |
|  | [701](http://df3/mascot/cgi/peptide_view.pl?file=../data/20121119/F002569.dat&query=701&hit=1&index=&px=1) | ***491.0287*** | ***980.0428*** |  |  |  |  |  |  |  |
|  | [702](http://df3/mascot/cgi/peptide_view.pl?file=../data/20121119/F002569.dat&query=702&hit=1&index=&px=1) | ***491.1296*** | ***980.2446*** |  |  |  |  |  |  |  |
|  | [703](http://df3/mascot/cgi/peptide_view.pl?file=../data/20121119/F002569.dat&query=703&hit=1&index=&px=1) | ***491.4669*** | ***980.9192*** |  |  |  |  |  |  |  |
|  | [704](http://df3/mascot/cgi/peptide_view.pl?file=../data/20121119/F002569.dat&query=704&hit=1&index=&px=1) | ***491.4860*** | ***980.9574*** |  |  |  |  |  |  |  |
|  | [705](http://df3/mascot/cgi/peptide_view.pl?file=../data/20121119/F002569.dat&query=705&hit=1&index=&px=1) | ***492.0172*** | ***982.0199*** |  |  |  |  |  |  |  |
|  | [706](http://df3/mascot/cgi/peptide_view.pl?file=../data/20121119/F002569.dat&query=706&hit=1&index=&px=1) | ***492.1996*** | ***982.3846*** |  |  |  |  |  |  |  |
|  | [707](http://df3/mascot/cgi/peptide_view.pl?file=../data/20121119/F002569.dat&query=707&hit=1&index=&px=1) | ***493.8096*** | ***985.6046*** |  |  |  |  |  |  |  |
|  | [708](http://df3/mascot/cgi/peptide_view.pl?file=../data/20121119/F002569.dat&query=708&hit=1&index=&px=1) | ***493.9063*** | ***985.7981*** |  |  |  |  |  |  |  |
|  | [709](http://df3/mascot/cgi/peptide_view.pl?file=../data/20121119/F002569.dat&query=709&hit=1&index=&px=1) | ***494.6313*** | ***987.2481*** |  |  |  |  |  |  |  |
|  | [711](http://df3/mascot/cgi/peptide_view.pl?file=../data/20121119/F002569.dat&query=711&hit=1&index=&px=1) | ***495.7702*** | ***989.5259*** |  |  |  |  |  |  |  |
|  | [712](http://df3/mascot/cgi/peptide_view.pl?file=../data/20121119/F002569.dat&query=712&hit=1&index=&px=1) | ***495.8923*** | ***989.7701*** |  |  |  |  |  |  |  |
|  | [713](http://df3/mascot/cgi/peptide_view.pl?file=../data/20121119/F002569.dat&query=713&hit=1&index=&px=1) | ***495.9868*** | ***989.9591*** |  |  |  |  |  |  |  |
|  | [714](http://df3/mascot/cgi/peptide_view.pl?file=../data/20121119/F002569.dat&query=714&hit=1&index=&px=1) | ***496.9565*** | ***991.8985*** |  |  |  |  |  |  |  |
|  | [715](http://df3/mascot/cgi/peptide_view.pl?file=../data/20121119/F002569.dat&query=715&hit=1&index=&px=1) | ***497.2473*** | ***992.4801*** |  |  |  |  |  |  |  |
|  | [716](http://df3/mascot/cgi/peptide_view.pl?file=../data/20121119/F002569.dat&query=716&hit=1&index=&px=1) | ***497.4368*** | ***992.8591*** |  |  |  |  |  |  |  |
|  | [717](http://df3/mascot/cgi/peptide_view.pl?file=../data/20121119/F002569.dat&query=717&hit=1&index=&px=1) | ***498.1116*** | ***994.2087*** |  |  |  |  |  |  |  |
|  | [718](http://df3/mascot/cgi/peptide_view.pl?file=../data/20121119/F002569.dat&query=718&hit=1&index=&px=1) | ***498.6096*** | ***995.2047*** |  |  |  |  |  |  |  |
|  | [719](http://df3/mascot/cgi/peptide_view.pl?file=../data/20121119/F002569.dat&query=719&hit=1&index=&px=1) | ***498.7751*** | ***995.5357*** |  |  |  |  |  |  |  |
|  | [720](http://df3/mascot/cgi/peptide_view.pl?file=../data/20121119/F002569.dat&query=720&hit=1&index=&px=1) | ***498.9847*** | ***995.9548*** |  |  |  |  |  |  |  |
|  | [721](http://df3/mascot/cgi/peptide_view.pl?file=../data/20121119/F002569.dat&query=721&hit=1&index=&px=1) | ***499.0386*** | ***996.0626*** |  |  |  |  |  |  |  |
|  | [722](http://df3/mascot/cgi/peptide_view.pl?file=../data/20121119/F002569.dat&query=722&hit=1&index=&px=1) | ***500.0985*** | ***998.1825*** |  |  |  |  |  |  |  |
|  | [723](http://df3/mascot/cgi/peptide_view.pl?file=../data/20121119/F002569.dat&query=723&hit=1&index=&px=1) | ***500.5003*** | ***998.9860*** |  |  |  |  |  |  |  |
|  | [724](http://df3/mascot/cgi/peptide_view.pl?file=../data/20121119/F002569.dat&query=724&hit=1&index=&px=1) | ***500.5376*** | ***999.0606*** |  |  |  |  |  |  |  |
|  | [725](http://df3/mascot/cgi/peptide_view.pl?file=../data/20121119/F002569.dat&query=725&hit=1&index=&px=1) | ***500.6252*** | ***999.2358*** |  |  |  |  |  |  |  |
|  | [726](http://df3/mascot/cgi/peptide_view.pl?file=../data/20121119/F002569.dat&query=726&hit=1&index=&px=1) | ***500.8992*** | ***999.7838*** |  |  |  |  |  |  |  |
|  | [727](http://df3/mascot/cgi/peptide_view.pl?file=../data/20121119/F002569.dat&query=727&hit=1&index=&px=1) | ***502.2226*** | ***1002.4306*** |  |  |  |  |  |  |  |
|  | [728](http://df3/mascot/cgi/peptide_view.pl?file=../data/20121119/F002569.dat&query=728&hit=1&index=&px=1) | ***502.2384*** | ***1002.4622*** |  |  |  |  |  |  |  |
|  | [729](http://df3/mascot/cgi/peptide_view.pl?file=../data/20121119/F002569.dat&query=729&hit=1&index=&px=1) | ***502.6038*** | ***1003.1931*** |  |  |  |  |  |  |  |
|  | [730](http://df3/mascot/cgi/peptide_view.pl?file=../data/20121119/F002569.dat&query=730&hit=1&index=&px=1) | ***502.9952*** | ***1003.9759*** |  |  |  |  |  |  |  |
|  | [731](http://df3/mascot/cgi/peptide_view.pl?file=../data/20121119/F002569.dat&query=731&hit=1&index=&px=1) | ***503.4716*** | ***1004.9286*** |  |  |  |  |  |  |  |
|  | [732](http://df3/mascot/cgi/peptide_view.pl?file=../data/20121119/F002569.dat&query=732&hit=1&index=&px=1) | ***504.0588*** | ***1006.1030*** |  |  |  |  |  |  |  |
|  | [733](http://df3/mascot/cgi/peptide_view.pl?file=../data/20121119/F002569.dat&query=733&hit=1&index=&px=1) | ***504.4126*** | ***1006.8106*** |  |  |  |  |  |  |  |
|  | [734](http://df3/mascot/cgi/peptide_view.pl?file=../data/20121119/F002569.dat&query=734&hit=1&index=&px=1) | ***505.4509*** | ***1008.8872*** |  |  |  |  |  |  |  |
|  | [735](http://df3/mascot/cgi/peptide_view.pl?file=../data/20121119/F002569.dat&query=735&hit=1&index=&px=1) | ***505.8083*** | ***1009.6020*** |  |  |  |  |  |  |  |
|  | [736](http://df3/mascot/cgi/peptide_view.pl?file=../data/20121119/F002569.dat&query=736&hit=1&index=&px=1) | ***505.8574*** | ***1009.7003*** |  |  |  |  |  |  |  |
|  | [737](http://df3/mascot/cgi/peptide_view.pl?file=../data/20121119/F002569.dat&query=737&hit=1&index=&px=1) | ***506.2373*** | ***1010.4600*** |  |  |  |  |  |  |  |
|  | [738](http://df3/mascot/cgi/peptide_view.pl?file=../data/20121119/F002569.dat&query=738&hit=1&index=&px=1) | ***506.4972*** | ***1010.9798*** |  |  |  |  |  |  |  |
|  | [739](http://df3/mascot/cgi/peptide_view.pl?file=../data/20121119/F002569.dat&query=739&hit=1&index=&px=1) | ***506.5215*** | ***1011.0284*** |  |  |  |  |  |  |  |
|  | [740](http://df3/mascot/cgi/peptide_view.pl?file=../data/20121119/F002569.dat&query=740&hit=1&index=&px=1) | ***507.4614*** | ***1012.9083*** |  |  |  |  |  |  |  |
|  | [741](http://df3/mascot/cgi/peptide_view.pl?file=../data/20121119/F002569.dat&query=741&hit=1&index=&px=1) | ***508.6469*** | ***1015.2793*** |  |  |  |  |  |  |  |
|  | [742](http://df3/mascot/cgi/peptide_view.pl?file=../data/20121119/F002569.dat&query=742&hit=1&index=&px=1) | ***508.6630*** | ***1015.3115*** |  |  |  |  |  |  |  |
|  | [743](http://df3/mascot/cgi/peptide_view.pl?file=../data/20121119/F002569.dat&query=743&hit=1&index=&px=1) | ***508.6992*** | ***1015.3839*** |  |  |  |  |  |  |  |
|  | [744](http://df3/mascot/cgi/peptide_view.pl?file=../data/20121119/F002569.dat&query=744&hit=1&index=&px=1) | ***508.7016*** | ***1015.3887*** |  |  |  |  |  |  |  |
|  | [745](http://df3/mascot/cgi/peptide_view.pl?file=../data/20121119/F002569.dat&query=745&hit=1&index=&px=1) | ***508.7572*** | ***1015.4999*** |  |  |  |  |  |  |  |
|  | [746](http://df3/mascot/cgi/peptide_view.pl?file=../data/20121119/F002569.dat&query=746&hit=1&index=&px=1) | ***508.8445*** | ***1015.6745*** |  |  |  |  |  |  |  |
|  | [747](http://df3/mascot/cgi/peptide_view.pl?file=../data/20121119/F002569.dat&query=747&hit=1&index=&px=1) | ***508.9255*** | ***1015.8364*** |  |  |  |  |  |  |  |
|  | [748](http://df3/mascot/cgi/peptide_view.pl?file=../data/20121119/F002569.dat&query=748&hit=1&index=&px=1) | ***508.9420*** | ***1015.8694*** |  |  |  |  |  |  |  |
|  | [749](http://df3/mascot/cgi/peptide_view.pl?file=../data/20121119/F002569.dat&query=749&hit=1&index=&px=1) | ***508.9566*** | ***1015.8987*** |  |  |  |  |  |  |  |
|  | [750](http://df3/mascot/cgi/peptide_view.pl?file=../data/20121119/F002569.dat&query=750&hit=1&index=&px=1) | ***509.5878*** | ***1017.1610*** |  |  |  |  |  |  |  |
|  | [751](http://df3/mascot/cgi/peptide_view.pl?file=../data/20121119/F002569.dat&query=751&hit=1&index=&px=1) | ***510.8853*** | ***1019.7561*** |  |  |  |  |  |  |  |
|  | [752](http://df3/mascot/cgi/peptide_view.pl?file=../data/20121119/F002569.dat&query=752&hit=1&index=&px=1) | ***511.5537*** | ***1021.0927*** |  |  |  |  |  |  |  |
|  | [753](http://df3/mascot/cgi/peptide_view.pl?file=../data/20121119/F002569.dat&query=753&hit=1&index=&px=1) | ***511.6442*** | ***1021.2738*** |  |  |  |  |  |  |  |
|  | [754](http://df3/mascot/cgi/peptide_view.pl?file=../data/20121119/F002569.dat&query=754&hit=1&index=&px=1) | ***511.8117*** | ***1021.6089*** |  |  |  |  |  |  |  |
|  | [755](http://df3/mascot/cgi/peptide_view.pl?file=../data/20121119/F002569.dat&query=755&hit=1&index=&px=1) | ***511.9536*** | ***1021.8926*** |  |  |  |  |  |  |  |
|  | [756](http://df3/mascot/cgi/peptide_view.pl?file=../data/20121119/F002569.dat&query=756&hit=1&index=&px=1) | ***511.9755*** | ***1021.9364*** |  |  |  |  |  |  |  |
|  | [757](http://df3/mascot/cgi/peptide_view.pl?file=../data/20121119/F002569.dat&query=757&hit=1&index=&px=1) | ***512.0048*** | ***1021.9951*** |  |  |  |  |  |  |  |
|  | [758](http://df3/mascot/cgi/peptide_view.pl?file=../data/20121119/F002569.dat&query=758&hit=1&index=&px=1) | ***512.0223*** | ***1022.0301*** |  |  |  |  |  |  |  |
|  | [759](http://df3/mascot/cgi/peptide_view.pl?file=../data/20121119/F002569.dat&query=759&hit=1&index=&px=1) | ***512.1931*** | ***1022.3717*** |  |  |  |  |  |  |  |
|  | [760](http://df3/mascot/cgi/peptide_view.pl?file=../data/20121119/F002569.dat&query=760&hit=1&index=&px=1) | ***513.7760*** | ***1025.5375*** |  |  |  |  |  |  |  |
|  | [761](http://df3/mascot/cgi/peptide_view.pl?file=../data/20121119/F002569.dat&query=761&hit=1&index=&px=1) | ***513.8651*** | ***1025.7157*** |  |  |  |  |  |  |  |
|  | [762](http://df3/mascot/cgi/peptide_view.pl?file=../data/20121119/F002569.dat&query=762&hit=1&index=&px=1) | ***514.3660*** | ***1026.7174*** |  |  |  |  |  |  |  |
|  | [764](http://df3/mascot/cgi/peptide_view.pl?file=../data/20121119/F002569.dat&query=764&hit=1&index=&px=1) | ***514.5790*** | ***1027.1434*** |  |  |  |  |  |  |  |
|  | [766](http://df3/mascot/cgi/peptide_view.pl?file=../data/20121119/F002569.dat&query=766&hit=1&index=&px=1) | ***515.3058*** | ***1028.5970*** |  |  |  |  |  |  |  |
|  | [767](http://df3/mascot/cgi/peptide_view.pl?file=../data/20121119/F002569.dat&query=767&hit=1&index=&px=1) | ***516.2855*** | ***1030.5564*** |  |  |  |  |  |  |  |
|  | [768](http://df3/mascot/cgi/peptide_view.pl?file=../data/20121119/F002569.dat&query=768&hit=1&index=&px=1) | ***516.4234*** | ***1030.8322*** |  |  |  |  |  |  |  |
|  | [769](http://df3/mascot/cgi/peptide_view.pl?file=../data/20121119/F002569.dat&query=769&hit=1&index=&px=1) | ***516.4858*** | ***1030.9570*** |  |  |  |  |  |  |  |
|  | [770](http://df3/mascot/cgi/peptide_view.pl?file=../data/20121119/F002569.dat&query=770&hit=1&index=&px=1) | ***516.6290*** | ***1031.2435*** |  |  |  |  |  |  |  |
|  | [773](http://df3/mascot/cgi/peptide_view.pl?file=../data/20121119/F002569.dat&query=773&hit=1&index=&px=1) | ***517.4426*** | ***1032.8707*** |  |  |  |  |  |  |  |
|  | [774](http://df3/mascot/cgi/peptide_view.pl?file=../data/20121119/F002569.dat&query=774&hit=1&index=&px=1) | ***517.5081*** | ***1033.0017*** |  |  |  |  |  |  |  |
|  | [777](http://df3/mascot/cgi/peptide_view.pl?file=../data/20121119/F002569.dat&query=777&hit=1&index=&px=1) | ***517.9739*** | ***1033.9333*** |  |  |  |  |  |  |  |
|  | [778](http://df3/mascot/cgi/peptide_view.pl?file=../data/20121119/F002569.dat&query=778&hit=1&index=&px=1) | ***518.0319*** | ***1034.0492*** |  |  |  |  |  |  |  |
|  | [779](http://df3/mascot/cgi/peptide_view.pl?file=../data/20121119/F002569.dat&query=779&hit=1&index=&px=1) | ***518.5138*** | ***1035.0130*** |  |  |  |  |  |  |  |
|  | [780](http://df3/mascot/cgi/peptide_view.pl?file=../data/20121119/F002569.dat&query=780&hit=1&index=&px=1) | ***518.5901*** | ***1035.1656*** |  |  |  |  |  |  |  |
|  | [781](http://df3/mascot/cgi/peptide_view.pl?file=../data/20121119/F002569.dat&query=781&hit=1&index=&px=1) | ***518.6040*** | ***1035.1935*** |  |  |  |  |  |  |  |
|  | [782](http://df3/mascot/cgi/peptide_view.pl?file=../data/20121119/F002569.dat&query=782&hit=1&index=&px=1) | ***518.6051*** | ***1035.1957*** |  |  |  |  |  |  |  |
|  | [783](http://df3/mascot/cgi/peptide_view.pl?file=../data/20121119/F002569.dat&query=783&hit=1&index=&px=1) | ***518.6377*** | ***1035.2608*** |  |  |  |  |  |  |  |
|  | [784](http://df3/mascot/cgi/peptide_view.pl?file=../data/20121119/F002569.dat&query=784&hit=1&index=&px=1) | ***518.6734*** | ***1035.3322*** |  |  |  |  |  |  |  |
|  | [785](http://df3/mascot/cgi/peptide_view.pl?file=../data/20121119/F002569.dat&query=785&hit=1&index=&px=1) | ***518.6825*** | ***1035.3504*** |  |  |  |  |  |  |  |
|  | [786](http://df3/mascot/cgi/peptide_view.pl?file=../data/20121119/F002569.dat&query=786&hit=1&index=&px=1) | ***518.6856*** | ***1035.3567*** |  |  |  |  |  |  |  |
|  | [787](http://df3/mascot/cgi/peptide_view.pl?file=../data/20121119/F002569.dat&query=787&hit=1&index=&px=1) | ***518.6859*** | ***1035.3573*** |  |  |  |  |  |  |  |
|  | [788](http://df3/mascot/cgi/peptide_view.pl?file=../data/20121119/F002569.dat&query=788&hit=1&index=&px=1) | ***518.7029*** | ***1035.3913*** |  |  |  |  |  |  |  |
|  | [789](http://df3/mascot/cgi/peptide_view.pl?file=../data/20121119/F002569.dat&query=789&hit=1&index=&px=1) | ***518.7036*** | ***1035.3927*** |  |  |  |  |  |  |  |
|  | [790](http://df3/mascot/cgi/peptide_view.pl?file=../data/20121119/F002569.dat&query=790&hit=1&index=&px=1) | ***518.7324*** | ***1035.4502*** |  |  |  |  |  |  |  |
|  | [791](http://df3/mascot/cgi/peptide_view.pl?file=../data/20121119/F002569.dat&query=791&hit=1&index=&px=1) | ***518.7386*** | ***1035.4627*** |  |  |  |  |  |  |  |
|  | [792](http://df3/mascot/cgi/peptide_view.pl?file=../data/20121119/F002569.dat&query=792&hit=1&index=&px=1) | ***518.7468*** | ***1035.4790*** |  |  |  |  |  |  |  |
|  | [793](http://df3/mascot/cgi/peptide_view.pl?file=../data/20121119/F002569.dat&query=793&hit=1&index=&px=1) | ***518.7540*** | ***1035.4935*** |  |  |  |  |  |  |  |
|  | [794](http://df3/mascot/cgi/peptide_view.pl?file=../data/20121119/F002569.dat&query=794&hit=1&index=&px=1) | ***518.7554*** | ***1035.4962*** |  |  |  |  |  |  |  |
|  | [795](http://df3/mascot/cgi/peptide_view.pl?file=../data/20121119/F002569.dat&query=795&hit=1&index=&px=1) | ***518.7559*** | ***1035.4972*** |  |  |  |  |  |  |  |
|  | [796](http://df3/mascot/cgi/peptide_view.pl?file=../data/20121119/F002569.dat&query=796&hit=1&index=&px=1) | ***518.7662*** | ***1035.5178*** |  |  |  |  |  |  |  |
|  | [797](http://df3/mascot/cgi/peptide_view.pl?file=../data/20121119/F002569.dat&query=797&hit=1&index=&px=1) | ***518.8398*** | ***1035.6650*** |  |  |  |  |  |  |  |
|  | [798](http://df3/mascot/cgi/peptide_view.pl?file=../data/20121119/F002569.dat&query=798&hit=1&index=&px=1) | ***518.8418*** | ***1035.6690*** |  |  |  |  |  |  |  |
|  | [799](http://df3/mascot/cgi/peptide_view.pl?file=../data/20121119/F002569.dat&query=799&hit=1&index=&px=1) | ***518.8458*** | ***1035.6770*** |  |  |  |  |  |  |  |
|  | [800](http://df3/mascot/cgi/peptide_view.pl?file=../data/20121119/F002569.dat&query=800&hit=1&index=&px=1) | ***518.8488*** | ***1035.6830*** |  |  |  |  |  |  |  |
|  | [801](http://df3/mascot/cgi/peptide_view.pl?file=../data/20121119/F002569.dat&query=801&hit=1&index=&px=1) | ***518.9241*** | ***1035.8336*** |  |  |  |  |  |  |  |
|  | [802](http://df3/mascot/cgi/peptide_view.pl?file=../data/20121119/F002569.dat&query=802&hit=1&index=&px=1) | ***519.8497*** | ***1037.6848*** |  |  |  |  |  |  |  |
|  | [806](http://df3/mascot/cgi/peptide_view.pl?file=../data/20121119/F002569.dat&query=806&hit=1&index=&px=1) | ***520.1240*** | ***1038.2334*** |  |  |  |  |  |  |  |
|  | [816](http://df3/mascot/cgi/peptide_view.pl?file=../data/20121119/F002569.dat&query=816&hit=1&index=&px=1) | ***520.6990*** | ***1039.3834*** |  |  |  |  |  |  |  |
|  | [817](http://df3/mascot/cgi/peptide_view.pl?file=../data/20121119/F002569.dat&query=817&hit=1&index=&px=1) | ***520.7473*** | ***1039.4800*** |  |  |  |  |  |  |  |
|  | [818](http://df3/mascot/cgi/peptide_view.pl?file=../data/20121119/F002569.dat&query=818&hit=1&index=&px=1) | ***520.7653*** | ***1039.5160*** |  |  |  |  |  |  |  |
|  | [823](http://df3/mascot/cgi/peptide_view.pl?file=../data/20121119/F002569.dat&query=823&hit=1&index=&px=1) | ***520.8488*** | ***1039.6830*** |  |  |  |  |  |  |  |
|  | [825](http://df3/mascot/cgi/peptide_view.pl?file=../data/20121119/F002569.dat&query=825&hit=1&index=&px=1) | ***520.9215*** | ***1039.8285*** |  |  |  |  |  |  |  |
|  | [826](http://df3/mascot/cgi/peptide_view.pl?file=../data/20121119/F002569.dat&query=826&hit=1&index=&px=1) | ***520.9301*** | ***1039.8457*** |  |  |  |  |  |  |  |
|  | [827](http://df3/mascot/cgi/peptide_view.pl?file=../data/20121119/F002569.dat&query=827&hit=1&index=&px=1) | ***520.9851*** | ***1039.9557*** |  |  |  |  |  |  |  |
|  | [836](http://df3/mascot/cgi/peptide_view.pl?file=../data/20121119/F002569.dat&query=836&hit=1&index=&px=1) | ***522.1008*** | ***1042.1871*** |  |  |  |  |  |  |  |
|  | [837](http://df3/mascot/cgi/peptide_view.pl?file=../data/20121119/F002569.dat&query=837&hit=1&index=&px=1) | ***522.5356*** | ***1043.0567*** |  |  |  |  |  |  |  |
|  | [838](http://df3/mascot/cgi/peptide_view.pl?file=../data/20121119/F002569.dat&query=838&hit=1&index=&px=1) | ***522.9896*** | ***1043.9647*** |  |  |  |  |  |  |  |
|  | [839](http://df3/mascot/cgi/peptide_view.pl?file=../data/20121119/F002569.dat&query=839&hit=1&index=&px=1) | ***523.0898*** | ***1044.1650*** |  |  |  |  |  |  |  |
|  | [840](http://df3/mascot/cgi/peptide_view.pl?file=../data/20121119/F002569.dat&query=840&hit=1&index=&px=1) | ***524.8423*** | ***1047.6700*** |  |  |  |  |  |  |  |
|  | [844](http://df3/mascot/cgi/peptide_view.pl?file=../data/20121119/F002569.dat&query=844&hit=1&index=&px=1) | ***525.0543*** | ***1048.0941*** |  |  |  |  |  |  |  |
|  | [845](http://df3/mascot/cgi/peptide_view.pl?file=../data/20121119/F002569.dat&query=845&hit=1&index=&px=1) | ***525.3683*** | ***1048.7220*** |  |  |  |  |  |  |  |
|  | [846](http://df3/mascot/cgi/peptide_view.pl?file=../data/20121119/F002569.dat&query=846&hit=1&index=&px=1) | ***525.5573*** | ***1049.1000*** |  |  |  |  |  |  |  |
|  | [847](http://df3/mascot/cgi/peptide_view.pl?file=../data/20121119/F002569.dat&query=847&hit=1&index=&px=1) | ***526.0769*** | ***1050.1393*** |  |  |  |  |  |  |  |
|  | [849](http://df3/mascot/cgi/peptide_view.pl?file=../data/20121119/F002569.dat&query=849&hit=1&index=&px=1) | ***526.4187*** | ***1050.8229*** |  |  |  |  |  |  |  |
|  | [850](http://df3/mascot/cgi/peptide_view.pl?file=../data/20121119/F002569.dat&query=850&hit=1&index=&px=1) | ***526.5084*** | ***1051.0023*** |  |  |  |  |  |  |  |
|  | [851](http://df3/mascot/cgi/peptide_view.pl?file=../data/20121119/F002569.dat&query=851&hit=1&index=&px=1) | ***526.5750*** | ***1051.1354*** |  |  |  |  |  |  |  |
|  | [852](http://df3/mascot/cgi/peptide_view.pl?file=../data/20121119/F002569.dat&query=852&hit=1&index=&px=1) | ***526.9208*** | ***1051.8270*** |  |  |  |  |  |  |  |
|  | [853](http://df3/mascot/cgi/peptide_view.pl?file=../data/20121119/F002569.dat&query=853&hit=1&index=&px=1) | ***527.1559*** | ***1052.2972*** |  |  |  |  |  |  |  |
|  | [854](http://df3/mascot/cgi/peptide_view.pl?file=../data/20121119/F002569.dat&query=854&hit=1&index=&px=1) | ***527.2810*** | ***1052.5475*** |  |  |  |  |  |  |  |
|  | [855](http://df3/mascot/cgi/peptide_view.pl?file=../data/20121119/F002569.dat&query=855&hit=1&index=&px=1) | ***527.2982*** | ***1052.5819*** |  |  |  |  |  |  |  |
|  | [856](http://df3/mascot/cgi/peptide_view.pl?file=../data/20121119/F002569.dat&query=856&hit=1&index=&px=1) | ***527.3262*** | ***1052.6378*** |  |  |  |  |  |  |  |
|  | [857](http://df3/mascot/cgi/peptide_view.pl?file=../data/20121119/F002569.dat&query=857&hit=1&index=&px=1) | ***527.7639*** | ***1053.5133*** |  |  |  |  |  |  |  |
|  | [858](http://df3/mascot/cgi/peptide_view.pl?file=../data/20121119/F002569.dat&query=858&hit=1&index=&px=1) | ***527.9711*** | ***1053.9276*** |  |  |  |  |  |  |  |
|  | [859](http://df3/mascot/cgi/peptide_view.pl?file=../data/20121119/F002569.dat&query=859&hit=1&index=&px=1) | ***529.5104*** | ***1057.0062*** |  |  |  |  |  |  |  |
|  | [860](http://df3/mascot/cgi/peptide_view.pl?file=../data/20121119/F002569.dat&query=860&hit=1&index=&px=1) | ***529.6481*** | ***1057.2817*** |  |  |  |  |  |  |  |
|  | [861](http://df3/mascot/cgi/peptide_view.pl?file=../data/20121119/F002569.dat&query=861&hit=1&index=&px=1) | ***529.9862*** | ***1057.9579*** |  |  |  |  |  |  |  |
|  | [862](http://df3/mascot/cgi/peptide_view.pl?file=../data/20121119/F002569.dat&query=862&hit=1&index=&px=1) | ***530.0653*** | ***1058.1161*** |  |  |  |  |  |  |  |
|  | [863](http://df3/mascot/cgi/peptide_view.pl?file=../data/20121119/F002569.dat&query=863&hit=1&index=&px=1) | ***530.0678*** | ***1058.1211*** |  |  |  |  |  |  |  |
|  | [864](http://df3/mascot/cgi/peptide_view.pl?file=../data/20121119/F002569.dat&query=864&hit=1&index=&px=1) | ***530.1010*** | ***1058.1875*** |  |  |  |  |  |  |  |
|  | [865](http://df3/mascot/cgi/peptide_view.pl?file=../data/20121119/F002569.dat&query=865&hit=1&index=&px=1) | ***530.1885*** | ***1058.3624*** |  |  |  |  |  |  |  |
|  | [866](http://df3/mascot/cgi/peptide_view.pl?file=../data/20121119/F002569.dat&query=866&hit=1&index=&px=1) | ***530.2762*** | ***1058.5379*** |  |  |  |  |  |  |  |
|  | [867](http://df3/mascot/cgi/peptide_view.pl?file=../data/20121119/F002569.dat&query=867&hit=1&index=&px=1) | ***530.4308*** | ***1058.8471*** |  |  |  |  |  |  |  |
|  | [868](http://df3/mascot/cgi/peptide_view.pl?file=../data/20121119/F002569.dat&query=868&hit=1&index=&px=1) | ***530.7519*** | ***1059.4892*** |  |  |  |  |  |  |  |
|  | [869](http://df3/mascot/cgi/peptide_view.pl?file=../data/20121119/F002569.dat&query=869&hit=1&index=&px=1) | ***530.8433*** | ***1059.6720*** |  |  |  |  |  |  |  |
|  | [870](http://df3/mascot/cgi/peptide_view.pl?file=../data/20121119/F002569.dat&query=870&hit=1&index=&px=1) | ***531.0128*** | ***1060.0111*** |  |  |  |  |  |  |  |
|  | [871](http://df3/mascot/cgi/peptide_view.pl?file=../data/20121119/F002569.dat&query=871&hit=1&index=&px=1) | ***531.3243*** | ***1060.6341*** |  |  |  |  |  |  |  |
|  | [872](http://df3/mascot/cgi/peptide_view.pl?file=../data/20121119/F002569.dat&query=872&hit=1&index=&px=1) | ***531.6469*** | ***1061.2793*** |  |  |  |  |  |  |  |
|  | [873](http://df3/mascot/cgi/peptide_view.pl?file=../data/20121119/F002569.dat&query=873&hit=1&index=&px=1) | ***531.9320*** | ***1061.8495*** |  |  |  |  |  |  |  |
|  | [874](http://df3/mascot/cgi/peptide_view.pl?file=../data/20121119/F002569.dat&query=874&hit=1&index=&px=1) | ***532.0804*** | ***1062.1463*** |  |  |  |  |  |  |  |
|  | [875](http://df3/mascot/cgi/peptide_view.pl?file=../data/20121119/F002569.dat&query=875&hit=1&index=&px=1) | ***532.1290*** | ***1062.2435*** |  |  |  |  |  |  |  |
|  | [876](http://df3/mascot/cgi/peptide_view.pl?file=../data/20121119/F002569.dat&query=876&hit=1&index=&px=1) | ***532.2045*** | ***1062.3944*** |  |  |  |  |  |  |  |
|  | [877](http://df3/mascot/cgi/peptide_view.pl?file=../data/20121119/F002569.dat&query=877&hit=1&index=&px=1) | ***532.2527*** | ***1062.4908*** |  |  |  |  |  |  |  |
|  | [878](http://df3/mascot/cgi/peptide_view.pl?file=../data/20121119/F002569.dat&query=878&hit=1&index=&px=1) | ***532.3205*** | ***1062.6264*** |  |  |  |  |  |  |  |
|  | [879](http://df3/mascot/cgi/peptide_view.pl?file=../data/20121119/F002569.dat&query=879&hit=1&index=&px=1) | ***532.7191*** | ***1063.4237*** |  |  |  |  |  |  |  |
|  | [880](http://df3/mascot/cgi/peptide_view.pl?file=../data/20121119/F002569.dat&query=880&hit=1&index=&px=1) | ***533.1898*** | ***1064.3651*** |  |  |  |  |  |  |  |
|  | [881](http://df3/mascot/cgi/peptide_view.pl?file=../data/20121119/F002569.dat&query=881&hit=1&index=&px=1) | ***533.9166*** | ***1065.8187*** |  |  |  |  |  |  |  |
|  | [882](http://df3/mascot/cgi/peptide_view.pl?file=../data/20121119/F002569.dat&query=882&hit=1&index=&px=1) | ***534.1552*** | ***1066.2959*** |  |  |  |  |  |  |  |
|  | [883](http://df3/mascot/cgi/peptide_view.pl?file=../data/20121119/F002569.dat&query=883&hit=1&index=&px=1) | ***535.2223*** | ***1068.4300*** |  |  |  |  |  |  |  |
|  | [884](http://df3/mascot/cgi/peptide_view.pl?file=../data/20121119/F002569.dat&query=884&hit=1&index=&px=1) | ***535.3555*** | ***1068.6964*** |  |  |  |  |  |  |  |
|  | [885](http://df3/mascot/cgi/peptide_view.pl?file=../data/20121119/F002569.dat&query=885&hit=1&index=&px=1) | ***535.5115*** | ***1069.0084*** |  |  |  |  |  |  |  |
|  | [886](http://df3/mascot/cgi/peptide_view.pl?file=../data/20121119/F002569.dat&query=886&hit=1&index=&px=1) | ***535.5255*** | ***1069.0364*** |  |  |  |  |  |  |  |
|  | [887](http://df3/mascot/cgi/peptide_view.pl?file=../data/20121119/F002569.dat&query=887&hit=1&index=&px=1) | ***535.7950*** | ***1069.5755*** |  |  |  |  |  |  |  |
|  | [888](http://df3/mascot/cgi/peptide_view.pl?file=../data/20121119/F002569.dat&query=888&hit=1&index=&px=1) | ***535.8543*** | ***1069.6941*** |  |  |  |  |  |  |  |
|  | [889](http://df3/mascot/cgi/peptide_view.pl?file=../data/20121119/F002569.dat&query=889&hit=1&index=&px=1) | ***536.5436*** | ***1071.0727*** |  |  |  |  |  |  |  |
|  | [890](http://df3/mascot/cgi/peptide_view.pl?file=../data/20121119/F002569.dat&query=890&hit=1&index=&px=1) | ***536.7166*** | ***1071.4187*** |  |  |  |  |  |  |  |
|  | [891](http://df3/mascot/cgi/peptide_view.pl?file=../data/20121119/F002569.dat&query=891&hit=1&index=&px=1) | ***536.9437*** | ***1071.8729*** |  |  |  |  |  |  |  |
|  | [893](http://df3/mascot/cgi/peptide_view.pl?file=../data/20121119/F002569.dat&query=893&hit=1&index=&px=1) | ***537.7478*** | ***1073.4811*** |  |  |  |  |  |  |  |
|  | [894](http://df3/mascot/cgi/peptide_view.pl?file=../data/20121119/F002569.dat&query=894&hit=1&index=&px=1) | ***537.8505*** | ***1073.6864*** |  |  |  |  |  |  |  |
|  | [895](http://df3/mascot/cgi/peptide_view.pl?file=../data/20121119/F002569.dat&query=895&hit=1&index=&px=1) | ***537.9393*** | ***1073.8641*** |  |  |  |  |  |  |  |
|  | [896](http://df3/mascot/cgi/peptide_view.pl?file=../data/20121119/F002569.dat&query=896&hit=1&index=&px=1) | ***537.9564*** | ***1073.8983*** |  |  |  |  |  |  |  |
|  | [897](http://df3/mascot/cgi/peptide_view.pl?file=../data/20121119/F002569.dat&query=897&hit=1&index=&px=1) | ***537.9805*** | ***1073.9464*** |  |  |  |  |  |  |  |
|  | [898](http://df3/mascot/cgi/peptide_view.pl?file=../data/20121119/F002569.dat&query=898&hit=1&index=&px=1) | ***537.9898*** | ***1073.9651*** |  |  |  |  |  |  |  |
|  | [899](http://df3/mascot/cgi/peptide_view.pl?file=../data/20121119/F002569.dat&query=899&hit=1&index=&px=1) | ***537.9949*** | ***1073.9752*** |  |  |  |  |  |  |  |
|  | [900](http://df3/mascot/cgi/peptide_view.pl?file=../data/20121119/F002569.dat&query=900&hit=1&index=&px=1) | ***538.0184*** | ***1074.0223*** |  |  |  |  |  |  |  |
|  | [901](http://df3/mascot/cgi/peptide_view.pl?file=../data/20121119/F002569.dat&query=901&hit=1&index=&px=1) | ***538.2411*** | ***1074.4676*** |  |  |  |  |  |  |  |
|  | [902](http://df3/mascot/cgi/peptide_view.pl?file=../data/20121119/F002569.dat&query=902&hit=1&index=&px=1) | ***538.7086*** | ***1075.4026*** |  |  |  |  |  |  |  |
|  | [903](http://df3/mascot/cgi/peptide_view.pl?file=../data/20121119/F002569.dat&query=903&hit=1&index=&px=1) | ***539.0281*** | ***1076.0417*** |  |  |  |  |  |  |  |
|  | [904](http://df3/mascot/cgi/peptide_view.pl?file=../data/20121119/F002569.dat&query=904&hit=1&index=&px=1) | ***540.2757*** | ***1078.5368*** |  |  |  |  |  |  |  |
|  | [905](http://df3/mascot/cgi/peptide_view.pl?file=../data/20121119/F002569.dat&query=905&hit=1&index=&px=1) | ***540.4398*** | ***1078.8650*** |  |  |  |  |  |  |  |
|  | [906](http://df3/mascot/cgi/peptide_view.pl?file=../data/20121119/F002569.dat&query=906&hit=1&index=&px=1) | ***540.6568*** | ***1079.2990*** |  |  |  |  |  |  |  |
|  | [907](http://df3/mascot/cgi/peptide_view.pl?file=../data/20121119/F002569.dat&query=907&hit=1&index=&px=1) | ***540.6832*** | ***1079.3518*** |  |  |  |  |  |  |  |
|  | [908](http://df3/mascot/cgi/peptide_view.pl?file=../data/20121119/F002569.dat&query=908&hit=1&index=&px=1) | ***540.7379*** | ***1079.4613*** |  |  |  |  |  |  |  |
|  | [909](http://df3/mascot/cgi/peptide_view.pl?file=../data/20121119/F002569.dat&query=909&hit=1&index=&px=1) | ***540.9594*** | ***1079.9042*** |  |  |  |  |  |  |  |
|  | [910](http://df3/mascot/cgi/peptide_view.pl?file=../data/20121119/F002569.dat&query=910&hit=1&index=&px=1) | ***541.1052*** | ***1080.1958*** |  |  |  |  |  |  |  |
|  | [911](http://df3/mascot/cgi/peptide_view.pl?file=../data/20121119/F002569.dat&query=911&hit=1&index=&px=1) | ***541.2416*** | ***1080.4686*** |  |  |  |  |  |  |  |
|  | [912](http://df3/mascot/cgi/peptide_view.pl?file=../data/20121119/F002569.dat&query=912&hit=1&index=&px=1) | ***541.2946*** | ***1080.5746*** |  |  |  |  |  |  |  |
|  | [913](http://df3/mascot/cgi/peptide_view.pl?file=../data/20121119/F002569.dat&query=913&hit=1&index=&px=1) | ***541.3948*** | ***1080.7750*** |  |  |  |  |  |  |  |
|  | [914](http://df3/mascot/cgi/peptide_view.pl?file=../data/20121119/F002569.dat&query=914&hit=1&index=&px=1) | ***541.6901*** | ***1081.3656*** |  |  |  |  |  |  |  |
|  | [915](http://df3/mascot/cgi/peptide_view.pl?file=../data/20121119/F002569.dat&query=915&hit=1&index=&px=1) | ***542.1007*** | ***1082.1869*** |  |  |  |  |  |  |  |
|  | [916](http://df3/mascot/cgi/peptide_view.pl?file=../data/20121119/F002569.dat&query=916&hit=1&index=&px=1) | ***542.1033*** | ***1082.1920*** |  |  |  |  |  |  |  |
|  | [917](http://df3/mascot/cgi/peptide_view.pl?file=../data/20121119/F002569.dat&query=917&hit=1&index=&px=1) | ***542.9156*** | ***1083.8167*** |  |  |  |  |  |  |  |
|  | [918](http://df3/mascot/cgi/peptide_view.pl?file=../data/20121119/F002569.dat&query=918&hit=1&index=&px=1) | ***544.0648*** | ***1086.1151*** |  |  |  |  |  |  |  |
|  | [919](http://df3/mascot/cgi/peptide_view.pl?file=../data/20121119/F002569.dat&query=919&hit=1&index=&px=1) | ***544.1127*** | ***1086.2108*** |  |  |  |  |  |  |  |
|  | [920](http://df3/mascot/cgi/peptide_view.pl?file=../data/20121119/F002569.dat&query=920&hit=1&index=&px=1) | ***544.3551*** | ***1086.6957*** |  |  |  |  |  |  |  |
|  | [921](http://df3/mascot/cgi/peptide_view.pl?file=../data/20121119/F002569.dat&query=921&hit=1&index=&px=1) | ***545.0078*** | ***1088.0010*** |  |  |  |  |  |  |  |
|  | [922](http://df3/mascot/cgi/peptide_view.pl?file=../data/20121119/F002569.dat&query=922&hit=1&index=&px=1) | ***545.1213*** | ***1088.2281*** |  |  |  |  |  |  |  |
|  | [923](http://df3/mascot/cgi/peptide_view.pl?file=../data/20121119/F002569.dat&query=923&hit=1&index=&px=1) | ***545.4054*** | ***1088.7962*** |  |  |  |  |  |  |  |
|  | [924](http://df3/mascot/cgi/peptide_view.pl?file=../data/20121119/F002569.dat&query=924&hit=1&index=&px=1) | ***545.9325*** | ***1089.8504*** |  |  |  |  |  |  |  |
|  | [925](http://df3/mascot/cgi/peptide_view.pl?file=../data/20121119/F002569.dat&query=925&hit=1&index=&px=1) | ***545.9722*** | ***1089.9298*** |  |  |  |  |  |  |  |
|  | [926](http://df3/mascot/cgi/peptide_view.pl?file=../data/20121119/F002569.dat&query=926&hit=1&index=&px=1) | ***546.0321*** | ***1090.0497*** |  |  |  |  |  |  |  |
|  | [927](http://df3/mascot/cgi/peptide_view.pl?file=../data/20121119/F002569.dat&query=927&hit=1&index=&px=1) | ***546.4327*** | ***1090.8508*** |  |  |  |  |  |  |  |
|  | [928](http://df3/mascot/cgi/peptide_view.pl?file=../data/20121119/F002569.dat&query=928&hit=1&index=&px=1) | ***546.6055*** | ***1091.1965*** |  |  |  |  |  |  |  |
|  | [929](http://df3/mascot/cgi/peptide_view.pl?file=../data/20121119/F002569.dat&query=929&hit=1&index=&px=1) | ***546.7507*** | ***1091.4869*** |  |  |  |  |  |  |  |
|  | [930](http://df3/mascot/cgi/peptide_view.pl?file=../data/20121119/F002569.dat&query=930&hit=1&index=&px=1) | ***546.8644*** | ***1091.7143*** |  |  |  |  |  |  |  |
|  | [931](http://df3/mascot/cgi/peptide_view.pl?file=../data/20121119/F002569.dat&query=931&hit=1&index=&px=1) | ***547.0283*** | ***1092.0421*** |  |  |  |  |  |  |  |
|  | [932](http://df3/mascot/cgi/peptide_view.pl?file=../data/20121119/F002569.dat&query=932&hit=1&index=&px=1) | ***547.3243*** | ***1092.6341*** |  |  |  |  |  |  |  |
|  | [933](http://df3/mascot/cgi/peptide_view.pl?file=../data/20121119/F002569.dat&query=933&hit=1&index=&px=1) | ***547.5044*** | ***1092.9942*** |  |  |  |  |  |  |  |
|  | [934](http://df3/mascot/cgi/peptide_view.pl?file=../data/20121119/F002569.dat&query=934&hit=1&index=&px=1) | ***547.5625*** | ***1093.1104*** |  |  |  |  |  |  |  |
|  | [935](http://df3/mascot/cgi/peptide_view.pl?file=../data/20121119/F002569.dat&query=935&hit=1&index=&px=1) | ***547.5701*** | ***1093.1256*** |  |  |  |  |  |  |  |
|  | [936](http://df3/mascot/cgi/peptide_view.pl?file=../data/20121119/F002569.dat&query=936&hit=1&index=&px=1) | ***547.6195*** | ***1093.2245*** |  |  |  |  |  |  |  |
|  | [937](http://df3/mascot/cgi/peptide_view.pl?file=../data/20121119/F002569.dat&query=937&hit=1&index=&px=1) | ***547.8911*** | ***1093.7677*** |  |  |  |  |  |  |  |
|  | [938](http://df3/mascot/cgi/peptide_view.pl?file=../data/20121119/F002569.dat&query=938&hit=1&index=&px=1) | ***548.1682*** | ***1094.3218*** |  |  |  |  |  |  |  |
|  | [939](http://df3/mascot/cgi/peptide_view.pl?file=../data/20121119/F002569.dat&query=939&hit=1&index=&px=1) | ***550.0906*** | ***1098.1666*** |  |  |  |  |  |  |  |
|  | [940](http://df3/mascot/cgi/peptide_view.pl?file=../data/20121119/F002569.dat&query=940&hit=1&index=&px=1) | ***550.1832*** | ***1098.3518*** |  |  |  |  |  |  |  |
|  | [941](http://df3/mascot/cgi/peptide_view.pl?file=../data/20121119/F002569.dat&query=941&hit=1&index=&px=1) | ***550.2294*** | ***1098.4442*** |  |  |  |  |  |  |  |
|  | [942](http://df3/mascot/cgi/peptide_view.pl?file=../data/20121119/F002569.dat&query=942&hit=1&index=&px=1) | ***550.7452*** | ***1099.4759*** |  |  |  |  |  |  |  |
|  | [943](http://df3/mascot/cgi/peptide_view.pl?file=../data/20121119/F002569.dat&query=943&hit=1&index=&px=1) | ***551.0369*** | ***1100.0593*** |  |  |  |  |  |  |  |
|  | [944](http://df3/mascot/cgi/peptide_view.pl?file=../data/20121119/F002569.dat&query=944&hit=1&index=&px=1) | ***551.5267*** | ***1101.0388*** |  |  |  |  |  |  |  |
|  | [945](http://df3/mascot/cgi/peptide_view.pl?file=../data/20121119/F002569.dat&query=945&hit=1&index=&px=1) | ***551.5862*** | ***1101.1579*** |  |  |  |  |  |  |  |
|  | [946](http://df3/mascot/cgi/peptide_view.pl?file=../data/20121119/F002569.dat&query=946&hit=1&index=&px=1) | ***551.9916*** | ***1101.9687*** |  |  |  |  |  |  |  |
|  | [947](http://df3/mascot/cgi/peptide_view.pl?file=../data/20121119/F002569.dat&query=947&hit=1&index=&px=1) | ***552.0184*** | ***1102.0223*** |  |  |  |  |  |  |  |
|  | [948](http://df3/mascot/cgi/peptide_view.pl?file=../data/20121119/F002569.dat&query=948&hit=1&index=&px=1) | ***552.0591*** | ***1102.1036*** |  |  |  |  |  |  |  |
|  | [949](http://df3/mascot/cgi/peptide_view.pl?file=../data/20121119/F002569.dat&query=949&hit=1&index=&px=1) | ***553.4948*** | ***1104.9750*** |  |  |  |  |  |  |  |
|  | [950](http://df3/mascot/cgi/peptide_view.pl?file=../data/20121119/F002569.dat&query=950&hit=1&index=&px=1) | ***553.5243*** | ***1105.0340*** |  |  |  |  |  |  |  |
|  | [951](http://df3/mascot/cgi/peptide_view.pl?file=../data/20121119/F002569.dat&query=951&hit=1&index=&px=1) | ***553.6835*** | ***1105.3525*** |  |  |  |  |  |  |  |
|  | [952](http://df3/mascot/cgi/peptide_view.pl?file=../data/20121119/F002569.dat&query=952&hit=1&index=&px=1) | ***553.9301*** | ***1105.8457*** |  |  |  |  |  |  |  |
|  | [953](http://df3/mascot/cgi/peptide_view.pl?file=../data/20121119/F002569.dat&query=953&hit=1&index=&px=1) | ***554.8616*** | ***1107.7086*** |  |  |  |  |  |  |  |
|  | [954](http://df3/mascot/cgi/peptide_view.pl?file=../data/20121119/F002569.dat&query=954&hit=1&index=&px=1) | ***555.3801*** | ***1108.7457*** |  |  |  |  |  |  |  |
|  | [955](http://df3/mascot/cgi/peptide_view.pl?file=../data/20121119/F002569.dat&query=955&hit=1&index=&px=1) | ***555.6381*** | ***1109.2616*** |  |  |  |  |  |  |  |
|  | [956](http://df3/mascot/cgi/peptide_view.pl?file=../data/20121119/F002569.dat&query=956&hit=1&index=&px=1) | ***555.6438*** | ***1109.2730*** |  |  |  |  |  |  |  |
|  | [957](http://df3/mascot/cgi/peptide_view.pl?file=../data/20121119/F002569.dat&query=957&hit=1&index=&px=1) | ***556.2287*** | ***1110.4428*** |  |  |  |  |  |  |  |
|  | [958](http://df3/mascot/cgi/peptide_view.pl?file=../data/20121119/F002569.dat&query=958&hit=1&index=&px=1) | ***556.2333*** | ***1110.4521*** |  |  |  |  |  |  |  |
|  | [959](http://df3/mascot/cgi/peptide_view.pl?file=../data/20121119/F002569.dat&query=959&hit=1&index=&px=1) | ***556.3280*** | ***1110.6415*** |  |  |  |  |  |  |  |
|  | [960](http://df3/mascot/cgi/peptide_view.pl?file=../data/20121119/F002569.dat&query=960&hit=1&index=&px=1) | ***556.7366*** | ***1111.4587*** |  |  |  |  |  |  |  |
|  | [961](http://df3/mascot/cgi/peptide_view.pl?file=../data/20121119/F002569.dat&query=961&hit=1&index=&px=1) | ***556.7958*** | ***1111.5770*** |  |  |  |  |  |  |  |
|  | [962](http://df3/mascot/cgi/peptide_view.pl?file=../data/20121119/F002569.dat&query=962&hit=1&index=&px=1) | ***556.8111*** | ***1111.6076*** |  |  |  |  |  |  |  |
|  | [963](http://df3/mascot/cgi/peptide_view.pl?file=../data/20121119/F002569.dat&query=963&hit=1&index=&px=1) | ***556.8378*** | ***1111.6611*** |  |  |  |  |  |  |  |
|  | [964](http://df3/mascot/cgi/peptide_view.pl?file=../data/20121119/F002569.dat&query=964&hit=1&index=&px=1) | ***556.9206*** | ***1111.8266*** |  |  |  |  |  |  |  |
|  | [965](http://df3/mascot/cgi/peptide_view.pl?file=../data/20121119/F002569.dat&query=965&hit=1&index=&px=1) | ***557.2961*** | ***1112.5777*** |  |  |  |  |  |  |  |
|  | [966](http://df3/mascot/cgi/peptide_view.pl?file=../data/20121119/F002569.dat&query=966&hit=1&index=&px=1) | ***558.7411*** | ***1115.4676*** |  |  |  |  |  |  |  |
|  | [967](http://df3/mascot/cgi/peptide_view.pl?file=../data/20121119/F002569.dat&query=967&hit=1&index=&px=1) | ***558.7528*** | ***1115.4911*** |  |  |  |  |  |  |  |
|  | [968](http://df3/mascot/cgi/peptide_view.pl?file=../data/20121119/F002569.dat&query=968&hit=1&index=&px=1) | ***559.1318*** | ***1116.2490*** |  |  |  |  |  |  |  |
|  | [969](http://df3/mascot/cgi/peptide_view.pl?file=../data/20121119/F002569.dat&query=969&hit=1&index=&px=1) | ***559.4987*** | ***1116.9829*** |  |  |  |  |  |  |  |
|  | [970](http://df3/mascot/cgi/peptide_view.pl?file=../data/20121119/F002569.dat&query=970&hit=1&index=&px=1) | ***560.2963*** | ***1118.5780*** |  |  |  |  |  |  |  |
|  | [971](http://df3/mascot/cgi/peptide_view.pl?file=../data/20121119/F002569.dat&query=971&hit=1&index=&px=1) | ***561.5395*** | ***1121.0644*** |  |  |  |  |  |  |  |
|  | [972](http://df3/mascot/cgi/peptide_view.pl?file=../data/20121119/F002569.dat&query=972&hit=1&index=&px=1) | ***561.6266*** | ***1121.2386*** |  |  |  |  |  |  |  |
|  | [973](http://df3/mascot/cgi/peptide_view.pl?file=../data/20121119/F002569.dat&query=973&hit=1&index=&px=1) | ***562.0997*** | ***1122.1849*** |  |  |  |  |  |  |  |
|  | [974](http://df3/mascot/cgi/peptide_view.pl?file=../data/20121119/F002569.dat&query=974&hit=1&index=&px=1) | ***562.4906*** | ***1122.9667*** |  |  |  |  |  |  |  |
|  | [975](http://df3/mascot/cgi/peptide_view.pl?file=../data/20121119/F002569.dat&query=975&hit=1&index=&px=1) | ***563.1432*** | ***1124.2719*** |  |  |  |  |  |  |  |
|  | [976](http://df3/mascot/cgi/peptide_view.pl?file=../data/20121119/F002569.dat&query=976&hit=1&index=&px=1) | ***564.4999*** | ***1126.9853*** |  |  |  |  |  |  |  |
|  | [977](http://df3/mascot/cgi/peptide_view.pl?file=../data/20121119/F002569.dat&query=977&hit=1&index=&px=1) | ***564.7562*** | ***1127.4979*** |  |  |  |  |  |  |  |
|  | [978](http://df3/mascot/cgi/peptide_view.pl?file=../data/20121119/F002569.dat&query=978&hit=1&index=&px=1) | ***564.7637*** | ***1127.5129*** |  |  |  |  |  |  |  |
|  | [979](http://df3/mascot/cgi/peptide_view.pl?file=../data/20121119/F002569.dat&query=979&hit=1&index=&px=1) | ***564.8715*** | ***1127.7284*** |  |  |  |  |  |  |  |
|  | [980](http://df3/mascot/cgi/peptide_view.pl?file=../data/20121119/F002569.dat&query=980&hit=1&index=&px=1) | ***564.9871*** | ***1127.9596*** |  |  |  |  |  |  |  |
|  | [981](http://df3/mascot/cgi/peptide_view.pl?file=../data/20121119/F002569.dat&query=981&hit=1&index=&px=1) | ***566.6904*** | ***1131.3662*** |  |  |  |  |  |  |  |
|  | [982](http://df3/mascot/cgi/peptide_view.pl?file=../data/20121119/F002569.dat&query=982&hit=1&index=&px=1) | ***566.7355*** | ***1131.4564*** |  |  |  |  |  |  |  |
|  | [983](http://df3/mascot/cgi/peptide_view.pl?file=../data/20121119/F002569.dat&query=983&hit=1&index=&px=1) | ***566.7360*** | ***1131.4574*** |  |  |  |  |  |  |  |
|  | [984](http://df3/mascot/cgi/peptide_view.pl?file=../data/20121119/F002569.dat&query=984&hit=1&index=&px=1) | ***567.0549*** | ***1132.0952*** |  |  |  |  |  |  |  |
|  | [985](http://df3/mascot/cgi/peptide_view.pl?file=../data/20121119/F002569.dat&query=985&hit=1&index=&px=1) | ***568.1636*** | ***1134.3127*** |  |  |  |  |  |  |  |
|  | [986](http://df3/mascot/cgi/peptide_view.pl?file=../data/20121119/F002569.dat&query=986&hit=1&index=&px=1) | ***568.5338*** | ***1135.0531*** |  |  |  |  |  |  |  |
|  | [987](http://df3/mascot/cgi/peptide_view.pl?file=../data/20121119/F002569.dat&query=987&hit=1&index=&px=1) | ***569.3412*** | ***1136.6678*** |  |  |  |  |  |  |  |
|  | [988](http://df3/mascot/cgi/peptide_view.pl?file=../data/20121119/F002569.dat&query=988&hit=1&index=&px=1) | ***569.4247*** | ***1136.8349*** |  |  |  |  |  |  |  |
|  | [989](http://df3/mascot/cgi/peptide_view.pl?file=../data/20121119/F002569.dat&query=989&hit=1&index=&px=1) | ***570.0052*** | ***1137.9959*** |  |  |  |  |  |  |  |
|  | [990](http://df3/mascot/cgi/peptide_view.pl?file=../data/20121119/F002569.dat&query=990&hit=1&index=&px=1) | ***570.5193*** | ***1139.0240*** |  |  |  |  |  |  |  |
|  | [991](http://df3/mascot/cgi/peptide_view.pl?file=../data/20121119/F002569.dat&query=991&hit=1&index=&px=1) | ***570.7020*** | ***1139.3895*** |  |  |  |  |  |  |  |
|  | [992](http://df3/mascot/cgi/peptide_view.pl?file=../data/20121119/F002569.dat&query=992&hit=1&index=&px=1) | ***570.7448*** | ***1139.4750*** |  |  |  |  |  |  |  |
|  | [993](http://df3/mascot/cgi/peptide_view.pl?file=../data/20121119/F002569.dat&query=993&hit=1&index=&px=1) | ***570.7557*** | ***1139.4968*** |  |  |  |  |  |  |  |
|  | [994](http://df3/mascot/cgi/peptide_view.pl?file=../data/20121119/F002569.dat&query=994&hit=1&index=&px=1) | ***570.7672*** | ***1139.5197*** |  |  |  |  |  |  |  |
|  | [995](http://df3/mascot/cgi/peptide_view.pl?file=../data/20121119/F002569.dat&query=995&hit=1&index=&px=1) | ***570.7676*** | ***1139.5207*** |  |  |  |  |  |  |  |
|  | [996](http://df3/mascot/cgi/peptide_view.pl?file=../data/20121119/F002569.dat&query=996&hit=1&index=&px=1) | ***570.7811*** | ***1139.5477*** |  |  |  |  |  |  |  |
|  | [997](http://df3/mascot/cgi/peptide_view.pl?file=../data/20121119/F002569.dat&query=997&hit=1&index=&px=1) | ***570.9553*** | ***1139.8961*** |  |  |  |  |  |  |  |
|  | [1006](http://df3/mascot/cgi/peptide_view.pl?file=../data/20121119/F002569.dat&query=1006&hit=1&index=&px=1) | ***572.3070*** | ***1142.5995*** |  |  |  |  |  |  |  |
|  | [1008](http://df3/mascot/cgi/peptide_view.pl?file=../data/20121119/F002569.dat&query=1008&hit=1&index=&px=1) | ***572.3861*** | ***1142.7577*** |  |  |  |  |  |  |  |
|  | [1009](http://df3/mascot/cgi/peptide_view.pl?file=../data/20121119/F002569.dat&query=1009&hit=1&index=&px=1) | ***572.7789*** | ***1143.5432*** |  |  |  |  |  |  |  |
|  | [1010](http://df3/mascot/cgi/peptide_view.pl?file=../data/20121119/F002569.dat&query=1010&hit=1&index=&px=1) | ***572.8596*** | ***1143.7046*** |  |  |  |  |  |  |  |
|  | [1011](http://df3/mascot/cgi/peptide_view.pl?file=../data/20121119/F002569.dat&query=1011&hit=1&index=&px=1) | ***573.0514*** | ***1144.0882*** |  |  |  |  |  |  |  |
|  | [1012](http://df3/mascot/cgi/peptide_view.pl?file=../data/20121119/F002569.dat&query=1012&hit=1&index=&px=1) | ***573.4120*** | ***1144.8094*** |  |  |  |  |  |  |  |
|  | [1013](http://df3/mascot/cgi/peptide_view.pl?file=../data/20121119/F002569.dat&query=1013&hit=1&index=&px=1) | ***573.7538*** | ***1145.4930*** |  |  |  |  |  |  |  |
|  | [1014](http://df3/mascot/cgi/peptide_view.pl?file=../data/20121119/F002569.dat&query=1014&hit=1&index=&px=1) | ***573.7570*** | ***1145.4994*** |  |  |  |  |  |  |  |
|  | [1020](http://df3/mascot/cgi/peptide_view.pl?file=../data/20121119/F002569.dat&query=1020&hit=1&index=&px=1) | ***575.4607*** | ***1148.9068*** |  |  |  |  |  |  |  |
|  | [1021](http://df3/mascot/cgi/peptide_view.pl?file=../data/20121119/F002569.dat&query=1021&hit=1&index=&px=1) | ***575.8082*** | ***1149.6019*** |  |  |  |  |  |  |  |
|  | [1022](http://df3/mascot/cgi/peptide_view.pl?file=../data/20121119/F002569.dat&query=1022&hit=1&index=&px=1) | ***576.1838*** | ***1150.3531*** |  |  |  |  |  |  |  |
|  | [1023](http://df3/mascot/cgi/peptide_view.pl?file=../data/20121119/F002569.dat&query=1023&hit=1&index=&px=1) | ***576.1868*** | ***1150.3590*** |  |  |  |  |  |  |  |
|  | [1024](http://df3/mascot/cgi/peptide_view.pl?file=../data/20121119/F002569.dat&query=1024&hit=1&index=&px=1) | ***576.2310*** | ***1150.4474*** |  |  |  |  |  |  |  |
|  | [1025](http://df3/mascot/cgi/peptide_view.pl?file=../data/20121119/F002569.dat&query=1025&hit=1&index=&px=1) | ***576.2661*** | ***1150.5177*** |  |  |  |  |  |  |  |
|  | [1026](http://df3/mascot/cgi/peptide_view.pl?file=../data/20121119/F002569.dat&query=1026&hit=1&index=&px=1) | ***576.3384*** | ***1150.6622*** |  |  |  |  |  |  |  |
|  | [1027](http://df3/mascot/cgi/peptide_view.pl?file=../data/20121119/F002569.dat&query=1027&hit=1&index=&px=1) | ***576.8269*** | ***1151.6393*** |  |  |  |  |  |  |  |
|  | [1028](http://df3/mascot/cgi/peptide_view.pl?file=../data/20121119/F002569.dat&query=1028&hit=1&index=&px=1) | ***577.3876*** | ***1152.7606*** |  |  |  |  |  |  |  |
|  | [1029](http://df3/mascot/cgi/peptide_view.pl?file=../data/20121119/F002569.dat&query=1029&hit=1&index=&px=1) | ***577.4245*** | ***1152.8344*** |  |  |  |  |  |  |  |
|  | [1030](http://df3/mascot/cgi/peptide_view.pl?file=../data/20121119/F002569.dat&query=1030&hit=1&index=&px=1) | ***578.7800*** | ***1155.5455*** |  |  |  |  |  |  |  |
|  | [1031](http://df3/mascot/cgi/peptide_view.pl?file=../data/20121119/F002569.dat&query=1031&hit=1&index=&px=1) | ***578.8680*** | ***1155.7215*** |  |  |  |  |  |  |  |
|  | [1032](http://df3/mascot/cgi/peptide_view.pl?file=../data/20121119/F002569.dat&query=1032&hit=1&index=&px=1) | ***579.0826*** | ***1156.1506*** |  |  |  |  |  |  |  |
|  | [1033](http://df3/mascot/cgi/peptide_view.pl?file=../data/20121119/F002569.dat&query=1033&hit=1&index=&px=1) | ***579.1516*** | ***1156.2885*** |  |  |  |  |  |  |  |
|  | [1034](http://df3/mascot/cgi/peptide_view.pl?file=../data/20121119/F002569.dat&query=1034&hit=1&index=&px=1) | ***579.4365*** | ***1156.8585*** |  |  |  |  |  |  |  |
|  | [1035](http://df3/mascot/cgi/peptide_view.pl?file=../data/20121119/F002569.dat&query=1035&hit=1&index=&px=1) | ***579.4814*** | ***1156.9482*** |  |  |  |  |  |  |  |
|  | [1036](http://df3/mascot/cgi/peptide_view.pl?file=../data/20121119/F002569.dat&query=1036&hit=1&index=&px=1) | ***579.5110*** | ***1157.0074*** |  |  |  |  |  |  |  |
|  | [1037](http://df3/mascot/cgi/peptide_view.pl?file=../data/20121119/F002569.dat&query=1037&hit=1&index=&px=1) | ***579.5861*** | ***1157.1577*** |  |  |  |  |  |  |  |
|  | [1038](http://df3/mascot/cgi/peptide_view.pl?file=../data/20121119/F002569.dat&query=1038&hit=1&index=&px=1) | ***579.6029*** | ***1157.1913*** |  |  |  |  |  |  |  |
|  | [1039](http://df3/mascot/cgi/peptide_view.pl?file=../data/20121119/F002569.dat&query=1039&hit=1&index=&px=1) | ***579.7561*** | ***1157.4977*** |  |  |  |  |  |  |  |
|  | [1040](http://df3/mascot/cgi/peptide_view.pl?file=../data/20121119/F002569.dat&query=1040&hit=1&index=&px=1) | ***579.7597*** | ***1157.5049*** |  |  |  |  |  |  |  |
|  | [1041](http://df3/mascot/cgi/peptide_view.pl?file=../data/20121119/F002569.dat&query=1041&hit=1&index=&px=1) | ***579.9363*** | ***1157.8580*** |  |  |  |  |  |  |  |
|  | [1042](http://df3/mascot/cgi/peptide_view.pl?file=../data/20121119/F002569.dat&query=1042&hit=1&index=&px=1) | ***579.9762*** | ***1157.9378*** |  |  |  |  |  |  |  |
|  | [1043](http://df3/mascot/cgi/peptide_view.pl?file=../data/20121119/F002569.dat&query=1043&hit=1&index=&px=1) | ***580.3196*** | ***1158.6246*** |  |  |  |  |  |  |  |
|  | [1044](http://df3/mascot/cgi/peptide_view.pl?file=../data/20121119/F002569.dat&query=1044&hit=1&index=&px=1) | ***580.5890*** | ***1159.1634*** |  |  |  |  |  |  |  |
|  | [1045](http://df3/mascot/cgi/peptide_view.pl?file=../data/20121119/F002569.dat&query=1045&hit=1&index=&px=1) | ***580.7865*** | ***1159.5584*** |  |  |  |  |  |  |  |
|  | [1046](http://df3/mascot/cgi/peptide_view.pl?file=../data/20121119/F002569.dat&query=1046&hit=1&index=&px=1) | ***580.8097*** | ***1159.6048*** |  |  |  |  |  |  |  |
|  | [1047](http://df3/mascot/cgi/peptide_view.pl?file=../data/20121119/F002569.dat&query=1047&hit=1&index=&px=1) | ***581.0537*** | ***1160.0929*** |  |  |  |  |  |  |  |
|  | [1048](http://df3/mascot/cgi/peptide_view.pl?file=../data/20121119/F002569.dat&query=1048&hit=1&index=&px=1) | ***581.6505*** | ***1161.2865*** |  |  |  |  |  |  |  |
|  | [1049](http://df3/mascot/cgi/peptide_view.pl?file=../data/20121119/F002569.dat&query=1049&hit=1&index=&px=1) | ***582.4666*** | ***1162.9187*** |  |  |  |  |  |  |  |
|  | [1050](http://df3/mascot/cgi/peptide_view.pl?file=../data/20121119/F002569.dat&query=1050&hit=1&index=&px=1) | ***582.5038*** | ***1162.9930*** |  |  |  |  |  |  |  |
|  | [1051](http://df3/mascot/cgi/peptide_view.pl?file=../data/20121119/F002569.dat&query=1051&hit=1&index=&px=1) | ***582.6501*** | ***1163.2856*** |  |  |  |  |  |  |  |
|  | [1052](http://df3/mascot/cgi/peptide_view.pl?file=../data/20121119/F002569.dat&query=1052&hit=1&index=&px=1) | ***582.6924*** | ***1163.3703*** |  |  |  |  |  |  |  |
|  | [1053](http://df3/mascot/cgi/peptide_view.pl?file=../data/20121119/F002569.dat&query=1053&hit=1&index=&px=1) | ***583.3149*** | ***1164.6153*** |  |  |  |  |  |  |  |
|  | [1054](http://df3/mascot/cgi/peptide_view.pl?file=../data/20121119/F002569.dat&query=1054&hit=1&index=&px=1) | ***583.4762*** | ***1164.9378*** |  |  |  |  |  |  |  |
|  | [1055](http://df3/mascot/cgi/peptide_view.pl?file=../data/20121119/F002569.dat&query=1055&hit=1&index=&px=1) | ***583.5114*** | ***1165.0082*** |  |  |  |  |  |  |  |
|  | [1056](http://df3/mascot/cgi/peptide_view.pl?file=../data/20121119/F002569.dat&query=1056&hit=1&index=&px=1) | ***583.8552*** | ***1165.6958*** |  |  |  |  |  |  |  |
|  | [1057](http://df3/mascot/cgi/peptide_view.pl?file=../data/20121119/F002569.dat&query=1057&hit=1&index=&px=1) | ***583.9195*** | ***1165.8244*** |  |  |  |  |  |  |  |
|  | [1058](http://df3/mascot/cgi/peptide_view.pl?file=../data/20121119/F002569.dat&query=1058&hit=1&index=&px=1) | ***583.9830*** | ***1165.9515*** |  |  |  |  |  |  |  |
|  | [1059](http://df3/mascot/cgi/peptide_view.pl?file=../data/20121119/F002569.dat&query=1059&hit=1&index=&px=1) | ***584.2892*** | ***1166.5639*** |  |  |  |  |  |  |  |
|  | [1060](http://df3/mascot/cgi/peptide_view.pl?file=../data/20121119/F002569.dat&query=1060&hit=1&index=&px=1) | ***584.3977*** | ***1166.7809*** |  |  |  |  |  |  |  |
|  | [1061](http://df3/mascot/cgi/peptide_view.pl?file=../data/20121119/F002569.dat&query=1061&hit=1&index=&px=1) | ***584.4933*** | ***1166.9720*** |  |  |  |  |  |  |  |
|  | [1062](http://df3/mascot/cgi/peptide_view.pl?file=../data/20121119/F002569.dat&query=1062&hit=1&index=&px=1) | ***584.6364*** | ***1167.2583*** |  |  |  |  |  |  |  |
|  | [1063](http://df3/mascot/cgi/peptide_view.pl?file=../data/20121119/F002569.dat&query=1063&hit=1&index=&px=1) | ***584.7607*** | ***1167.5069*** |  |  |  |  |  |  |  |
|  | [1064](http://df3/mascot/cgi/peptide_view.pl?file=../data/20121119/F002569.dat&query=1064&hit=1&index=&px=1) | ***584.7824*** | ***1167.5503*** |  |  |  |  |  |  |  |
|  | [1065](http://df3/mascot/cgi/peptide_view.pl?file=../data/20121119/F002569.dat&query=1065&hit=1&index=&px=1) | ***584.9329*** | ***1167.8512*** |  |  |  |  |  |  |  |
|  | [1066](http://df3/mascot/cgi/peptide_view.pl?file=../data/20121119/F002569.dat&query=1066&hit=1&index=&px=1) | ***584.9563*** | ***1167.8980*** |  |  |  |  |  |  |  |
|  | [1067](http://df3/mascot/cgi/peptide_view.pl?file=../data/20121119/F002569.dat&query=1067&hit=1&index=&px=1) | ***585.0015*** | ***1167.9885*** |  |  |  |  |  |  |  |
|  | [1068](http://df3/mascot/cgi/peptide_view.pl?file=../data/20121119/F002569.dat&query=1068&hit=1&index=&px=1) | ***585.1930*** | ***1168.3714*** |  |  |  |  |  |  |  |
|  | [1069](http://df3/mascot/cgi/peptide_view.pl?file=../data/20121119/F002569.dat&query=1069&hit=1&index=&px=1) | ***585.2693*** | ***1168.5240*** |  |  |  |  |  |  |  |
|  | [1070](http://df3/mascot/cgi/peptide_view.pl?file=../data/20121119/F002569.dat&query=1070&hit=1&index=&px=1) | ***585.6829*** | ***1169.3512*** |  |  |  |  |  |  |  |
|  | [1071](http://df3/mascot/cgi/peptide_view.pl?file=../data/20121119/F002569.dat&query=1071&hit=1&index=&px=1) | ***586.1049*** | ***1170.1952*** |  |  |  |  |  |  |  |
|  | [1072](http://df3/mascot/cgi/peptide_view.pl?file=../data/20121119/F002569.dat&query=1072&hit=1&index=&px=1) | ***586.2503*** | ***1170.4861*** |  |  |  |  |  |  |  |
|  | [1073](http://df3/mascot/cgi/peptide_view.pl?file=../data/20121119/F002569.dat&query=1073&hit=1&index=&px=1) | ***586.5705*** | ***1171.1264*** |  |  |  |  |  |  |  |
|  | [1074](http://df3/mascot/cgi/peptide_view.pl?file=../data/20121119/F002569.dat&query=1074&hit=1&index=&px=1) | ***586.6472*** | ***1171.2799*** |  |  |  |  |  |  |  |
|  | [1075](http://df3/mascot/cgi/peptide_view.pl?file=../data/20121119/F002569.dat&query=1075&hit=1&index=&px=1) | ***587.3156*** | ***1172.6166*** |  |  |  |  |  |  |  |
|  | [1076](http://df3/mascot/cgi/peptide_view.pl?file=../data/20121119/F002569.dat&query=1076&hit=1&index=&px=1) | ***588.2283*** | ***1174.4420*** |  |  |  |  |  |  |  |
|  | [1077](http://df3/mascot/cgi/peptide_view.pl?file=../data/20121119/F002569.dat&query=1077&hit=1&index=&px=1) | ***588.3141*** | ***1174.6136*** |  |  |  |  |  |  |  |
|  | [1078](http://df3/mascot/cgi/peptide_view.pl?file=../data/20121119/F002569.dat&query=1078&hit=1&index=&px=1) | ***588.3431*** | ***1174.6717*** |  |  |  |  |  |  |  |
|  | [1079](http://df3/mascot/cgi/peptide_view.pl?file=../data/20121119/F002569.dat&query=1079&hit=1&index=&px=1) | ***588.7216*** | ***1175.4287*** |  |  |  |  |  |  |  |
|  | [1080](http://df3/mascot/cgi/peptide_view.pl?file=../data/20121119/F002569.dat&query=1080&hit=1&index=&px=1) | ***588.7322*** | ***1175.4499*** |  |  |  |  |  |  |  |
|  | [1081](http://df3/mascot/cgi/peptide_view.pl?file=../data/20121119/F002569.dat&query=1081&hit=1&index=&px=1) | ***588.7388*** | ***1175.4631*** |  |  |  |  |  |  |  |
|  | [1082](http://df3/mascot/cgi/peptide_view.pl?file=../data/20121119/F002569.dat&query=1082&hit=1&index=&px=1) | ***588.7614*** | ***1175.5082*** |  |  |  |  |  |  |  |
|  | [1083](http://df3/mascot/cgi/peptide_view.pl?file=../data/20121119/F002569.dat&query=1083&hit=1&index=&px=1) | ***588.7620*** | ***1175.5095*** |  |  |  |  |  |  |  |
|  | [1084](http://df3/mascot/cgi/peptide_view.pl?file=../data/20121119/F002569.dat&query=1084&hit=1&index=&px=1) | ***588.8486*** | ***1175.6827*** |  |  |  |  |  |  |  |
|  | [1085](http://df3/mascot/cgi/peptide_view.pl?file=../data/20121119/F002569.dat&query=1085&hit=1&index=&px=1) | ***588.8530*** | ***1175.6915*** |  |  |  |  |  |  |  |
|  | [1086](http://df3/mascot/cgi/peptide_view.pl?file=../data/20121119/F002569.dat&query=1086&hit=1&index=&px=1) | ***588.8613*** | ***1175.7080*** |  |  |  |  |  |  |  |
|  | [1087](http://df3/mascot/cgi/peptide_view.pl?file=../data/20121119/F002569.dat&query=1087&hit=1&index=&px=1) | ***589.5922*** | ***1177.1698*** |  |  |  |  |  |  |  |
|  | [1088](http://df3/mascot/cgi/peptide_view.pl?file=../data/20121119/F002569.dat&query=1088&hit=1&index=&px=1) | ***589.6177*** | ***1177.2208*** |  |  |  |  |  |  |  |
|  | [1089](http://df3/mascot/cgi/peptide_view.pl?file=../data/20121119/F002569.dat&query=1089&hit=1&index=&px=1) | ***589.6490*** | ***1177.2834*** |  |  |  |  |  |  |  |
|  | [1090](http://df3/mascot/cgi/peptide_view.pl?file=../data/20121119/F002569.dat&query=1090&hit=1&index=&px=1) | ***589.6537*** | ***1177.2928*** |  |  |  |  |  |  |  |
|  | [1091](http://df3/mascot/cgi/peptide_view.pl?file=../data/20121119/F002569.dat&query=1091&hit=1&index=&px=1) | ***589.7931*** | ***1177.5716*** |  |  |  |  |  |  |  |
|  | [1092](http://df3/mascot/cgi/peptide_view.pl?file=../data/20121119/F002569.dat&query=1092&hit=1&index=&px=1) | ***590.2455*** | ***1178.4764*** |  |  |  |  |  |  |  |
|  | [1093](http://df3/mascot/cgi/peptide_view.pl?file=../data/20121119/F002569.dat&query=1093&hit=1&index=&px=1) | ***590.7067*** | ***1179.3988*** |  |  |  |  |  |  |  |
|  | [1094](http://df3/mascot/cgi/peptide_view.pl?file=../data/20121119/F002569.dat&query=1094&hit=1&index=&px=1) | ***590.7606*** | ***1179.5067*** |  |  |  |  |  |  |  |
|  | [1095](http://df3/mascot/cgi/peptide_view.pl?file=../data/20121119/F002569.dat&query=1095&hit=1&index=&px=1) | ***590.7728*** | ***1179.5311*** |  |  |  |  |  |  |  |
|  | [1096](http://df3/mascot/cgi/peptide_view.pl?file=../data/20121119/F002569.dat&query=1096&hit=1&index=&px=1) | ***590.8362*** | ***1179.6578*** |  |  |  |  |  |  |  |
|  | [1097](http://df3/mascot/cgi/peptide_view.pl?file=../data/20121119/F002569.dat&query=1097&hit=1&index=&px=1) | ***590.8726*** | ***1179.7306*** |  |  |  |  |  |  |  |
|  | [1099](http://df3/mascot/cgi/peptide_view.pl?file=../data/20121119/F002569.dat&query=1099&hit=1&index=&px=1) | ***591.2491*** | ***1180.4837*** |  |  |  |  |  |  |  |
|  | [1100](http://df3/mascot/cgi/peptide_view.pl?file=../data/20121119/F002569.dat&query=1100&hit=1&index=&px=1) | ***591.5013*** | ***1180.9880*** |  |  |  |  |  |  |  |
|  | [1101](http://df3/mascot/cgi/peptide_view.pl?file=../data/20121119/F002569.dat&query=1101&hit=1&index=&px=1) | ***591.5807*** | ***1181.1468*** |  |  |  |  |  |  |  |
|  | [1102](http://df3/mascot/cgi/peptide_view.pl?file=../data/20121119/F002569.dat&query=1102&hit=1&index=&px=1) | ***591.8912*** | ***1181.7679*** |  |  |  |  |  |  |  |
|  | [1103](http://df3/mascot/cgi/peptide_view.pl?file=../data/20121119/F002569.dat&query=1103&hit=1&index=&px=1) | ***591.8971*** | ***1181.7796*** |  |  |  |  |  |  |  |
|  | [1105](http://df3/mascot/cgi/peptide_view.pl?file=../data/20121119/F002569.dat&query=1105&hit=1&index=&px=1) | ***592.2262*** | ***1182.4378*** |  |  |  |  |  |  |  |
|  | [1106](http://df3/mascot/cgi/peptide_view.pl?file=../data/20121119/F002569.dat&query=1106&hit=1&index=&px=1) | ***592.2567*** | ***1182.4989*** |  |  |  |  |  |  |  |
|  | [1107](http://df3/mascot/cgi/peptide_view.pl?file=../data/20121119/F002569.dat&query=1107&hit=1&index=&px=1) | ***592.3755*** | ***1182.7364*** |  |  |  |  |  |  |  |
|  | [1108](http://df3/mascot/cgi/peptide_view.pl?file=../data/20121119/F002569.dat&query=1108&hit=1&index=&px=1) | ***592.4595*** | ***1182.9044*** |  |  |  |  |  |  |  |
|  | [1109](http://df3/mascot/cgi/peptide_view.pl?file=../data/20121119/F002569.dat&query=1109&hit=1&index=&px=1) | ***592.4610*** | ***1182.9074*** |  |  |  |  |  |  |  |
|  | [1110](http://df3/mascot/cgi/peptide_view.pl?file=../data/20121119/F002569.dat&query=1110&hit=1&index=&px=1) | ***592.4965*** | ***1182.9785*** |  |  |  |  |  |  |  |
|  | [1111](http://df3/mascot/cgi/peptide_view.pl?file=../data/20121119/F002569.dat&query=1111&hit=1&index=&px=1) | ***592.5862*** | ***1183.1579*** |  |  |  |  |  |  |  |
|  | [1112](http://df3/mascot/cgi/peptide_view.pl?file=../data/20121119/F002569.dat&query=1112&hit=1&index=&px=1) | ***592.5933*** | ***1183.1720*** |  |  |  |  |  |  |  |
|  | [1113](http://df3/mascot/cgi/peptide_view.pl?file=../data/20121119/F002569.dat&query=1113&hit=1&index=&px=1) | ***592.6052*** | ***1183.1959*** |  |  |  |  |  |  |  |
|  | [1114](http://df3/mascot/cgi/peptide_view.pl?file=../data/20121119/F002569.dat&query=1114&hit=1&index=&px=1) | ***592.6086*** | ***1183.2026*** |  |  |  |  |  |  |  |
|  | [1116](http://df3/mascot/cgi/peptide_view.pl?file=../data/20121119/F002569.dat&query=1116&hit=1&index=&px=1) | ***592.6151*** | ***1183.2157*** |  |  |  |  |  |  |  |
|  | [1117](http://df3/mascot/cgi/peptide_view.pl?file=../data/20121119/F002569.dat&query=1117&hit=1&index=&px=1) | ***592.6165*** | ***1183.2185*** |  |  |  |  |  |  |  |
|  | [1118](http://df3/mascot/cgi/peptide_view.pl?file=../data/20121119/F002569.dat&query=1118&hit=1&index=&px=1) | ***592.6238*** | ***1183.2330*** |  |  |  |  |  |  |  |
|  | [1119](http://df3/mascot/cgi/peptide_view.pl?file=../data/20121119/F002569.dat&query=1119&hit=1&index=&px=1) | ***592.6249*** | ***1183.2352*** |  |  |  |  |  |  |  |
|  | [1120](http://df3/mascot/cgi/peptide_view.pl?file=../data/20121119/F002569.dat&query=1120&hit=1&index=&px=1) | ***592.6639*** | ***1183.3133*** |  |  |  |  |  |  |  |
|  | [1121](http://df3/mascot/cgi/peptide_view.pl?file=../data/20121119/F002569.dat&query=1121&hit=1&index=&px=1) | ***592.6733*** | ***1183.3321*** |  |  |  |  |  |  |  |
|  | [1122](http://df3/mascot/cgi/peptide_view.pl?file=../data/20121119/F002569.dat&query=1122&hit=1&index=&px=1) | ***592.6851*** | ***1183.3557*** |  |  |  |  |  |  |  |
|  | [1123](http://df3/mascot/cgi/peptide_view.pl?file=../data/20121119/F002569.dat&query=1123&hit=1&index=&px=1) | ***592.6877*** | ***1183.3608*** |  |  |  |  |  |  |  |
|  | [1124](http://df3/mascot/cgi/peptide_view.pl?file=../data/20121119/F002569.dat&query=1124&hit=1&index=&px=1) | ***592.6959*** | ***1183.3773*** |  |  |  |  |  |  |  |
|  | [1125](http://df3/mascot/cgi/peptide_view.pl?file=../data/20121119/F002569.dat&query=1125&hit=1&index=&px=1) | ***592.7026*** | ***1183.3907*** |  |  |  |  |  |  |  |
|  | [1126](http://df3/mascot/cgi/peptide_view.pl?file=../data/20121119/F002569.dat&query=1126&hit=1&index=&px=1) | ***592.7053*** | ***1183.3961*** |  |  |  |  |  |  |  |
|  | [1127](http://df3/mascot/cgi/peptide_view.pl?file=../data/20121119/F002569.dat&query=1127&hit=1&index=&px=1) | ***592.7176*** | ***1183.4206*** |  |  |  |  |  |  |  |
|  | [1128](http://df3/mascot/cgi/peptide_view.pl?file=../data/20121119/F002569.dat&query=1128&hit=1&index=&px=1) | ***592.7240*** | ***1183.4334*** |  |  |  |  |  |  |  |
|  | [1129](http://df3/mascot/cgi/peptide_view.pl?file=../data/20121119/F002569.dat&query=1129&hit=1&index=&px=1) | ***592.7417*** | ***1183.4688*** |  |  |  |  |  |  |  |
|  | [1130](http://df3/mascot/cgi/peptide_view.pl?file=../data/20121119/F002569.dat&query=1130&hit=1&index=&px=1) | ***592.7454*** | ***1183.4763*** |  |  |  |  |  |  |  |
|  | [1131](http://df3/mascot/cgi/peptide_view.pl?file=../data/20121119/F002569.dat&query=1131&hit=1&index=&px=1) | ***592.7617*** | ***1183.5089*** |  |  |  |  |  |  |  |
|  | [1132](http://df3/mascot/cgi/peptide_view.pl?file=../data/20121119/F002569.dat&query=1132&hit=1&index=&px=1) | ***592.7714*** | ***1183.5282*** |  |  |  |  |  |  |  |
|  | [1133](http://df3/mascot/cgi/peptide_view.pl?file=../data/20121119/F002569.dat&query=1133&hit=1&index=&px=1) | ***592.8135*** | ***1183.6124*** |  |  |  |  |  |  |  |
|  | [1134](http://df3/mascot/cgi/peptide_view.pl?file=../data/20121119/F002569.dat&query=1134&hit=1&index=&px=1) | ***592.8157*** | ***1183.6168*** |  |  |  |  |  |  |  |
|  | [1135](http://df3/mascot/cgi/peptide_view.pl?file=../data/20121119/F002569.dat&query=1135&hit=1&index=&px=1) | ***592.8295*** | ***1183.6445*** |  |  |  |  |  |  |  |
|  | [1136](http://df3/mascot/cgi/peptide_view.pl?file=../data/20121119/F002569.dat&query=1136&hit=1&index=&px=1) | ***592.8812*** | ***1183.7479*** |  |  |  |  |  |  |  |
|  | [1137](http://df3/mascot/cgi/peptide_view.pl?file=../data/20121119/F002569.dat&query=1137&hit=1&index=&px=1) | ***592.8929*** | ***1183.7712*** |  |  |  |  |  |  |  |
|  | [1139](http://df3/mascot/cgi/peptide_view.pl?file=../data/20121119/F002569.dat&query=1139&hit=1&index=&px=1) | ***592.9546*** | ***1183.8946*** |  |  |  |  |  |  |  |
|  | [1140](http://df3/mascot/cgi/peptide_view.pl?file=../data/20121119/F002569.dat&query=1140&hit=1&index=&px=1) | ***592.9651*** | ***1183.9157*** |  |  |  |  |  |  |  |
|  | [1141](http://df3/mascot/cgi/peptide_view.pl?file=../data/20121119/F002569.dat&query=1141&hit=1&index=&px=1) | ***593.0496*** | ***1184.0847*** |  |  |  |  |  |  |  |
|  | [1142](http://df3/mascot/cgi/peptide_view.pl?file=../data/20121119/F002569.dat&query=1142&hit=1&index=&px=1) | ***593.0704*** | ***1184.1263*** |  |  |  |  |  |  |  |
|  | [1143](http://df3/mascot/cgi/peptide_view.pl?file=../data/20121119/F002569.dat&query=1143&hit=1&index=&px=1) | ***593.7936*** | ***1185.5726*** |  |  |  |  |  |  |  |
|  | [1144](http://df3/mascot/cgi/peptide_view.pl?file=../data/20121119/F002569.dat&query=1144&hit=1&index=&px=1) | ***594.6051*** | ***1187.1957*** |  |  |  |  |  |  |  |
|  | [1145](http://df3/mascot/cgi/peptide_view.pl?file=../data/20121119/F002569.dat&query=1145&hit=1&index=&px=1) | ***594.6086*** | ***1187.2027*** |  |  |  |  |  |  |  |
|  | [1147](http://df3/mascot/cgi/peptide_view.pl?file=../data/20121119/F002569.dat&query=1147&hit=1&index=&px=1) | ***595.1176*** | ***1188.2206*** |  |  |  |  |  |  |  |
|  | [1148](http://df3/mascot/cgi/peptide_view.pl?file=../data/20121119/F002569.dat&query=1148&hit=1&index=&px=1) | ***595.5205*** | ***1189.0265*** |  |  |  |  |  |  |  |
|  | [1150](http://df3/mascot/cgi/peptide_view.pl?file=../data/20121119/F002569.dat&query=1150&hit=1&index=&px=1) | ***595.9185*** | ***1189.8224*** |  |  |  |  |  |  |  |
|  | [1151](http://df3/mascot/cgi/peptide_view.pl?file=../data/20121119/F002569.dat&query=1151&hit=1&index=&px=1) | ***595.9554*** | ***1189.8963*** |  |  |  |  |  |  |  |
|  | [1152](http://df3/mascot/cgi/peptide_view.pl?file=../data/20121119/F002569.dat&query=1152&hit=1&index=&px=1) | ***596.1346*** | ***1190.2547*** |  |  |  |  |  |  |  |
|  | [1153](http://df3/mascot/cgi/peptide_view.pl?file=../data/20121119/F002569.dat&query=1153&hit=1&index=&px=1) | ***596.4840*** | ***1190.9535*** |  |  |  |  |  |  |  |
|  | [1154](http://df3/mascot/cgi/peptide_view.pl?file=../data/20121119/F002569.dat&query=1154&hit=1&index=&px=1) | ***596.7051*** | ***1191.3957*** |  |  |  |  |  |  |  |
|  | [1155](http://df3/mascot/cgi/peptide_view.pl?file=../data/20121119/F002569.dat&query=1155&hit=1&index=&px=1) | ***596.7972*** | ***1191.5798*** |  |  |  |  |  |  |  |
|  | [1156](http://df3/mascot/cgi/peptide_view.pl?file=../data/20121119/F002569.dat&query=1156&hit=1&index=&px=1) | ***596.8064*** | ***1191.5982*** |  |  |  |  |  |  |  |
|  | [1157](http://df3/mascot/cgi/peptide_view.pl?file=../data/20121119/F002569.dat&query=1157&hit=1&index=&px=1) | ***597.0554*** | ***1192.0963*** |  |  |  |  |  |  |  |
|  | [1158](http://df3/mascot/cgi/peptide_view.pl?file=../data/20121119/F002569.dat&query=1158&hit=1&index=&px=1) | ***597.2391*** | ***1192.4637*** |  |  |  |  |  |  |  |
|  | [1159](http://df3/mascot/cgi/peptide_view.pl?file=../data/20121119/F002569.dat&query=1159&hit=1&index=&px=1) | ***597.2410*** | ***1192.4675*** |  |  |  |  |  |  |  |
|  | [1167](http://df3/mascot/cgi/peptide_view.pl?file=../data/20121119/F002569.dat&query=1167&hit=1&index=&px=1) | ***598.1008*** | ***1194.1870*** |  |  |  |  |  |  |  |
|  | [1169](http://df3/mascot/cgi/peptide_view.pl?file=../data/20121119/F002569.dat&query=1169&hit=1&index=&px=1) | ***598.3403*** | ***1194.6661*** |  |  |  |  |  |  |  |
|  | [1170](http://df3/mascot/cgi/peptide_view.pl?file=../data/20121119/F002569.dat&query=1170&hit=1&index=&px=1) | ***598.4130*** | ***1194.8115*** |  |  |  |  |  |  |  |
|  | [1172](http://df3/mascot/cgi/peptide_view.pl?file=../data/20121119/F002569.dat&query=1172&hit=1&index=&px=1) | ***598.6821*** | ***1195.3497*** |  |  |  |  |  |  |  |
|  | [1173](http://df3/mascot/cgi/peptide_view.pl?file=../data/20121119/F002569.dat&query=1173&hit=1&index=&px=1) | ***599.4841*** | ***1196.9536*** |  |  |  |  |  |  |  |
|  | [1174](http://df3/mascot/cgi/peptide_view.pl?file=../data/20121119/F002569.dat&query=1174&hit=1&index=&px=1) | ***599.7795*** | ***1197.5445*** |  |  |  |  |  |  |  |
|  | [1175](http://df3/mascot/cgi/peptide_view.pl?file=../data/20121119/F002569.dat&query=1175&hit=1&index=&px=1) | ***599.9678*** | ***1197.9210*** |  |  |  |  |  |  |  |
|  | [1176](http://df3/mascot/cgi/peptide_view.pl?file=../data/20121119/F002569.dat&query=1176&hit=1&index=&px=1) | ***600.4890*** | ***1198.9635*** |  |  |  |  |  |  |  |
|  | [1177](http://df3/mascot/cgi/peptide_view.pl?file=../data/20121119/F002569.dat&query=1177&hit=1&index=&px=1) | ***600.7329*** | ***1199.4513*** |  |  |  |  |  |  |  |
|  | [1178](http://df3/mascot/cgi/peptide_view.pl?file=../data/20121119/F002569.dat&query=1178&hit=1&index=&px=1) | ***600.9847*** | ***1199.9548*** |  |  |  |  |  |  |  |
|  | [1179](http://df3/mascot/cgi/peptide_view.pl?file=../data/20121119/F002569.dat&query=1179&hit=1&index=&px=1) | ***601.0223*** | ***1200.0300*** |  |  |  |  |  |  |  |
|  | [1180](http://df3/mascot/cgi/peptide_view.pl?file=../data/20121119/F002569.dat&query=1180&hit=1&index=&px=1) | ***601.7317*** | ***1201.4488*** |  |  |  |  |  |  |  |
|  | [1181](http://df3/mascot/cgi/peptide_view.pl?file=../data/20121119/F002569.dat&query=1181&hit=1&index=&px=1) | ***601.7833*** | ***1201.5521*** |  |  |  |  |  |  |  |
|  | [1182](http://df3/mascot/cgi/peptide_view.pl?file=../data/20121119/F002569.dat&query=1182&hit=1&index=&px=1) | ***601.8472*** | ***1201.6798*** |  |  |  |  |  |  |  |
|  | [1183](http://df3/mascot/cgi/peptide_view.pl?file=../data/20121119/F002569.dat&query=1183&hit=1&index=&px=1) | ***602.0251*** | ***1202.0357*** |  |  |  |  |  |  |  |
|  | [1184](http://df3/mascot/cgi/peptide_view.pl?file=../data/20121119/F002569.dat&query=1184&hit=1&index=&px=1) | ***602.4477*** | ***1202.8808*** |  |  |  |  |  |  |  |
|  | [1185](http://df3/mascot/cgi/peptide_view.pl?file=../data/20121119/F002569.dat&query=1185&hit=1&index=&px=1) | ***602.7684*** | ***1203.5223*** |  |  |  |  |  |  |  |
|  | [1186](http://df3/mascot/cgi/peptide_view.pl?file=../data/20121119/F002569.dat&query=1186&hit=1&index=&px=1) | ***602.7877*** | ***1203.5608*** |  |  |  |  |  |  |  |
|  | [1187](http://df3/mascot/cgi/peptide_view.pl?file=../data/20121119/F002569.dat&query=1187&hit=1&index=&px=1) | ***602.8042*** | ***1203.5938*** |  |  |  |  |  |  |  |
|  | [1188](http://df3/mascot/cgi/peptide_view.pl?file=../data/20121119/F002569.dat&query=1188&hit=1&index=&px=1) | ***602.9060*** | ***1203.7975*** |  |  |  |  |  |  |  |
|  | [1189](http://df3/mascot/cgi/peptide_view.pl?file=../data/20121119/F002569.dat&query=1189&hit=1&index=&px=1) | ***602.9303*** | ***1203.8460*** |  |  |  |  |  |  |  |
|  | [1190](http://df3/mascot/cgi/peptide_view.pl?file=../data/20121119/F002569.dat&query=1190&hit=1&index=&px=1) | ***602.9743*** | ***1203.9341*** |  |  |  |  |  |  |  |
|  | [1191](http://df3/mascot/cgi/peptide_view.pl?file=../data/20121119/F002569.dat&query=1191&hit=1&index=&px=1) | ***603.0320*** | ***1204.0494*** |  |  |  |  |  |  |  |
|  | [1192](http://df3/mascot/cgi/peptide_view.pl?file=../data/20121119/F002569.dat&query=1192&hit=1&index=&px=1) | ***603.2357*** | ***1204.4569*** |  |  |  |  |  |  |  |
|  | [1193](http://df3/mascot/cgi/peptide_view.pl?file=../data/20121119/F002569.dat&query=1193&hit=1&index=&px=1) | ***603.8135*** | ***1205.6124*** |  |  |  |  |  |  |  |
|  | [1194](http://df3/mascot/cgi/peptide_view.pl?file=../data/20121119/F002569.dat&query=1194&hit=1&index=&px=1) | ***604.4545*** | ***1206.8944*** |  |  |  |  |  |  |  |
|  | [1195](http://df3/mascot/cgi/peptide_view.pl?file=../data/20121119/F002569.dat&query=1195&hit=1&index=&px=1) | ***605.3928*** | ***1208.7711*** |  |  |  |  |  |  |  |
|  | [1196](http://df3/mascot/cgi/peptide_view.pl?file=../data/20121119/F002569.dat&query=1196&hit=1&index=&px=1) | ***605.4771*** | ***1208.9396*** |  |  |  |  |  |  |  |
|  | [1197](http://df3/mascot/cgi/peptide_view.pl?file=../data/20121119/F002569.dat&query=1197&hit=1&index=&px=1) | ***606.3680*** | ***1210.7215*** |  |  |  |  |  |  |  |
|  | [1198](http://df3/mascot/cgi/peptide_view.pl?file=../data/20121119/F002569.dat&query=1198&hit=1&index=&px=1) | ***606.4863*** | ***1210.9580*** |  |  |  |  |  |  |  |
|  | [1199](http://df3/mascot/cgi/peptide_view.pl?file=../data/20121119/F002569.dat&query=1199&hit=1&index=&px=1) | ***606.6373*** | ***1211.2600*** |  |  |  |  |  |  |  |
|  | [1200](http://df3/mascot/cgi/peptide_view.pl?file=../data/20121119/F002569.dat&query=1200&hit=1&index=&px=1) | ***606.6413*** | ***1211.2680*** |  |  |  |  |  |  |  |
|  | [1201](http://df3/mascot/cgi/peptide_view.pl?file=../data/20121119/F002569.dat&query=1201&hit=1&index=&px=1) | ***607.0277*** | ***1212.0409*** |  |  |  |  |  |  |  |
|  | [1202](http://df3/mascot/cgi/peptide_view.pl?file=../data/20121119/F002569.dat&query=1202&hit=1&index=&px=1) | ***607.1417*** | ***1212.2689*** |  |  |  |  |  |  |  |
|  | [1203](http://df3/mascot/cgi/peptide_view.pl?file=../data/20121119/F002569.dat&query=1203&hit=1&index=&px=1) | ***607.2570*** | ***1212.4995*** |  |  |  |  |  |  |  |
|  | [1204](http://df3/mascot/cgi/peptide_view.pl?file=../data/20121119/F002569.dat&query=1204&hit=1&index=&px=1) | ***608.1345*** | ***1214.2545*** |  |  |  |  |  |  |  |
|  | [1205](http://df3/mascot/cgi/peptide_view.pl?file=../data/20121119/F002569.dat&query=1205&hit=1&index=&px=1) | ***608.3431*** | ***1214.6717*** |  |  |  |  |  |  |  |
|  | [1209](http://df3/mascot/cgi/peptide_view.pl?file=../data/20121119/F002569.dat&query=1209&hit=1&index=&px=1) | ***609.1265*** | ***1216.2384*** |  |  |  |  |  |  |  |
|  | [1210](http://df3/mascot/cgi/peptide_view.pl?file=../data/20121119/F002569.dat&query=1210&hit=1&index=&px=1) | ***609.8315*** | ***1217.6485*** |  |  |  |  |  |  |  |
|  | [1211](http://df3/mascot/cgi/peptide_view.pl?file=../data/20121119/F002569.dat&query=1211&hit=1&index=&px=1) | ***610.2071*** | ***1218.3996*** |  |  |  |  |  |  |  |
|  | [1217](http://df3/mascot/cgi/peptide_view.pl?file=../data/20121119/F002569.dat&query=1217&hit=1&index=&px=1) | ***610.7529*** | ***1219.4913*** |  |  |  |  |  |  |  |
|  | [1230](http://df3/mascot/cgi/peptide_view.pl?file=../data/20121119/F002569.dat&query=1230&hit=1&index=&px=1) | ***611.0562*** | ***1220.0978*** |  |  |  |  |  |  |  |
|  | [1236](http://df3/mascot/cgi/peptide_view.pl?file=../data/20121119/F002569.dat&query=1236&hit=1&index=&px=1) | ***613.7405*** | ***1225.4664*** |  |  |  |  |  |  |  |
|  | [1237](http://df3/mascot/cgi/peptide_view.pl?file=../data/20121119/F002569.dat&query=1237&hit=1&index=&px=1) | ***614.4402*** | ***1226.8659*** |  |  |  |  |  |  |  |
|  | [1238](http://df3/mascot/cgi/peptide_view.pl?file=../data/20121119/F002569.dat&query=1238&hit=1&index=&px=1) | ***614.9903*** | ***1227.9660*** |  |  |  |  |  |  |  |
|  | [1239](http://df3/mascot/cgi/peptide_view.pl?file=../data/20121119/F002569.dat&query=1239&hit=1&index=&px=1) | ***615.2687*** | ***1228.5229*** |  |  |  |  |  |  |  |
|  | [1240](http://df3/mascot/cgi/peptide_view.pl?file=../data/20121119/F002569.dat&query=1240&hit=1&index=&px=1) | ***615.3517*** | ***1228.6889*** |  |  |  |  |  |  |  |
|  | [1241](http://df3/mascot/cgi/peptide_view.pl?file=../data/20121119/F002569.dat&query=1241&hit=1&index=&px=1) | ***615.3878*** | ***1228.7611*** |  |  |  |  |  |  |  |
|  | [1242](http://df3/mascot/cgi/peptide_view.pl?file=../data/20121119/F002569.dat&query=1242&hit=1&index=&px=1) | ***615.4733*** | ***1228.9320*** |  |  |  |  |  |  |  |
|  | [1243](http://df3/mascot/cgi/peptide_view.pl?file=../data/20121119/F002569.dat&query=1243&hit=1&index=&px=1) | ***615.4772*** | ***1228.9398*** |  |  |  |  |  |  |  |
|  | [1244](http://df3/mascot/cgi/peptide_view.pl?file=../data/20121119/F002569.dat&query=1244&hit=1&index=&px=1) | ***615.5295*** | ***1229.0444*** |  |  |  |  |  |  |  |
|  | [1245](http://df3/mascot/cgi/peptide_view.pl?file=../data/20121119/F002569.dat&query=1245&hit=1&index=&px=1) | ***615.7539*** | ***1229.4933*** |  |  |  |  |  |  |  |
|  | [1246](http://df3/mascot/cgi/peptide_view.pl?file=../data/20121119/F002569.dat&query=1246&hit=1&index=&px=1) | ***616.4836*** | ***1230.9526*** |  |  |  |  |  |  |  |
|  | [1247](http://df3/mascot/cgi/peptide_view.pl?file=../data/20121119/F002569.dat&query=1247&hit=1&index=&px=1) | ***616.4895*** | ***1230.9645*** |  |  |  |  |  |  |  |
|  | [1248](http://df3/mascot/cgi/peptide_view.pl?file=../data/20121119/F002569.dat&query=1248&hit=1&index=&px=1) | ***616.6080*** | ***1231.2015*** |  |  |  |  |  |  |  |
|  | [1249](http://df3/mascot/cgi/peptide_view.pl?file=../data/20121119/F002569.dat&query=1249&hit=1&index=&px=1) | ***616.6127*** | ***1231.2109*** |  |  |  |  |  |  |  |
|  | [1250](http://df3/mascot/cgi/peptide_view.pl?file=../data/20121119/F002569.dat&query=1250&hit=1&index=&px=1) | ***616.6475*** | ***1231.2804*** |  |  |  |  |  |  |  |
|  | [1251](http://df3/mascot/cgi/peptide_view.pl?file=../data/20121119/F002569.dat&query=1251&hit=1&index=&px=1) | ***617.1615*** | ***1232.3084*** |  |  |  |  |  |  |  |
|  | [1252](http://df3/mascot/cgi/peptide_view.pl?file=../data/20121119/F002569.dat&query=1252&hit=1&index=&px=1) | ***617.5548*** | ***1233.0951*** |  |  |  |  |  |  |  |
|  | [1253](http://df3/mascot/cgi/peptide_view.pl?file=../data/20121119/F002569.dat&query=1253&hit=1&index=&px=1) | ***617.7817*** | ***1233.5488*** |  |  |  |  |  |  |  |
|  | [1254](http://df3/mascot/cgi/peptide_view.pl?file=../data/20121119/F002569.dat&query=1254&hit=1&index=&px=1) | ***618.0720*** | ***1234.1295*** |  |  |  |  |  |  |  |
|  | [1255](http://df3/mascot/cgi/peptide_view.pl?file=../data/20121119/F002569.dat&query=1255&hit=1&index=&px=1) | ***618.2257*** | ***1234.4369*** |  |  |  |  |  |  |  |
|  | [1256](http://df3/mascot/cgi/peptide_view.pl?file=../data/20121119/F002569.dat&query=1256&hit=1&index=&px=1) | ***618.3468*** | ***1234.6791*** |  |  |  |  |  |  |  |
|  | [1257](http://df3/mascot/cgi/peptide_view.pl?file=../data/20121119/F002569.dat&query=1257&hit=1&index=&px=1) | ***618.4515*** | ***1234.8885*** |  |  |  |  |  |  |  |
|  | [1258](http://df3/mascot/cgi/peptide_view.pl?file=../data/20121119/F002569.dat&query=1258&hit=1&index=&px=1) | ***618.4930*** | ***1234.9715*** |  |  |  |  |  |  |  |
|  | [1259](http://df3/mascot/cgi/peptide_view.pl?file=../data/20121119/F002569.dat&query=1259&hit=1&index=&px=1) | ***618.7195*** | ***1235.4244*** |  |  |  |  |  |  |  |
|  | [1260](http://df3/mascot/cgi/peptide_view.pl?file=../data/20121119/F002569.dat&query=1260&hit=1&index=&px=1) | ***618.9827*** | ***1235.9508*** |  |  |  |  |  |  |  |
|  | [1261](http://df3/mascot/cgi/peptide_view.pl?file=../data/20121119/F002569.dat&query=1261&hit=1&index=&px=1) | ***619.3478*** | ***1236.6810*** |  |  |  |  |  |  |  |
|  | [1262](http://df3/mascot/cgi/peptide_view.pl?file=../data/20121119/F002569.dat&query=1262&hit=1&index=&px=1) | ***619.3508*** | ***1236.6871*** |  |  |  |  |  |  |  |
|  | [1263](http://df3/mascot/cgi/peptide_view.pl?file=../data/20121119/F002569.dat&query=1263&hit=1&index=&px=1) | ***619.3937*** | ***1236.7728*** |  |  |  |  |  |  |  |
|  | [1264](http://df3/mascot/cgi/peptide_view.pl?file=../data/20121119/F002569.dat&query=1264&hit=1&index=&px=1) | ***619.6936*** | ***1237.3727*** |  |  |  |  |  |  |  |
|  | [1265](http://df3/mascot/cgi/peptide_view.pl?file=../data/20121119/F002569.dat&query=1265&hit=1&index=&px=1) | ***619.7662*** | ***1237.5179*** |  |  |  |  |  |  |  |
|  | [1266](http://df3/mascot/cgi/peptide_view.pl?file=../data/20121119/F002569.dat&query=1266&hit=1&index=&px=1) | ***620.0096*** | ***1238.0047*** |  |  |  |  |  |  |  |
|  | [1267](http://df3/mascot/cgi/peptide_view.pl?file=../data/20121119/F002569.dat&query=1267&hit=1&index=&px=1) | ***620.0386*** | ***1238.0626*** |  |  |  |  |  |  |  |
|  | [1268](http://df3/mascot/cgi/peptide_view.pl?file=../data/20121119/F002569.dat&query=1268&hit=1&index=&px=1) | ***620.2325*** | ***1238.4505*** |  |  |  |  |  |  |  |
|  | [1269](http://df3/mascot/cgi/peptide_view.pl?file=../data/20121119/F002569.dat&query=1269&hit=1&index=&px=1) | ***620.2384*** | ***1238.4623*** |  |  |  |  |  |  |  |
|  | [1270](http://df3/mascot/cgi/peptide_view.pl?file=../data/20121119/F002569.dat&query=1270&hit=1&index=&px=1) | ***620.2504*** | ***1238.4863*** |  |  |  |  |  |  |  |
|  | [1271](http://df3/mascot/cgi/peptide_view.pl?file=../data/20121119/F002569.dat&query=1271&hit=1&index=&px=1) | ***620.3158*** | ***1238.6170*** |  |  |  |  |  |  |  |
|  | [1272](http://df3/mascot/cgi/peptide_view.pl?file=../data/20121119/F002569.dat&query=1272&hit=1&index=&px=1) | ***620.3485*** | ***1238.6825*** |  |  |  |  |  |  |  |
|  | [1273](http://df3/mascot/cgi/peptide_view.pl?file=../data/20121119/F002569.dat&query=1273&hit=1&index=&px=1) | ***620.6044*** | ***1239.1942*** |  |  |  |  |  |  |  |
|  | [1274](http://df3/mascot/cgi/peptide_view.pl?file=../data/20121119/F002569.dat&query=1274&hit=1&index=&px=1) | ***621.0464*** | ***1240.0782*** |  |  |  |  |  |  |  |
|  | [1275](http://df3/mascot/cgi/peptide_view.pl?file=../data/20121119/F002569.dat&query=1275&hit=1&index=&px=1) | ***621.1345*** | ***1240.2545*** |  |  |  |  |  |  |  |
|  | [1276](http://df3/mascot/cgi/peptide_view.pl?file=../data/20121119/F002569.dat&query=1276&hit=1&index=&px=1) | ***621.2519*** | ***1240.4892*** |  |  |  |  |  |  |  |
|  | [1277](http://df3/mascot/cgi/peptide_view.pl?file=../data/20121119/F002569.dat&query=1277&hit=1&index=&px=1) | ***621.2783*** | ***1240.5421*** |  |  |  |  |  |  |  |
|  | [1278](http://df3/mascot/cgi/peptide_view.pl?file=../data/20121119/F002569.dat&query=1278&hit=1&index=&px=1) | ***621.3202*** | ***1240.6258*** |  |  |  |  |  |  |  |
|  | [1279](http://df3/mascot/cgi/peptide_view.pl?file=../data/20121119/F002569.dat&query=1279&hit=1&index=&px=1) | ***621.3895*** | ***1240.7644*** |  |  |  |  |  |  |  |
|  | [1280](http://df3/mascot/cgi/peptide_view.pl?file=../data/20121119/F002569.dat&query=1280&hit=1&index=&px=1) | ***621.3997*** | ***1240.7848*** |  |  |  |  |  |  |  |
|  | [1281](http://df3/mascot/cgi/peptide_view.pl?file=../data/20121119/F002569.dat&query=1281&hit=1&index=&px=1) | ***621.4428*** | ***1240.8711*** |  |  |  |  |  |  |  |
|  | [1282](http://df3/mascot/cgi/peptide_view.pl?file=../data/20121119/F002569.dat&query=1282&hit=1&index=&px=1) | ***621.4799*** | ***1240.9453*** |  |  |  |  |  |  |  |
|  | [1283](http://df3/mascot/cgi/peptide_view.pl?file=../data/20121119/F002569.dat&query=1283&hit=1&index=&px=1) | ***621.4907*** | ***1240.9669*** |  |  |  |  |  |  |  |
|  | [1284](http://df3/mascot/cgi/peptide_view.pl?file=../data/20121119/F002569.dat&query=1284&hit=1&index=&px=1) | ***621.5952*** | ***1241.1759*** |  |  |  |  |  |  |  |
|  | [1285](http://df3/mascot/cgi/peptide_view.pl?file=../data/20121119/F002569.dat&query=1285&hit=1&index=&px=1) | ***621.5958*** | ***1241.1771*** |  |  |  |  |  |  |  |
|  | [1287](http://df3/mascot/cgi/peptide_view.pl?file=../data/20121119/F002569.dat&query=1287&hit=1&index=&px=1) | ***623.3114*** | ***1244.6083*** |  |  |  |  |  |  |  |
|  | [1288](http://df3/mascot/cgi/peptide_view.pl?file=../data/20121119/F002569.dat&query=1288&hit=1&index=&px=1) | ***623.4292*** | ***1244.8438*** |  |  |  |  |  |  |  |
|  | [1289](http://df3/mascot/cgi/peptide_view.pl?file=../data/20121119/F002569.dat&query=1289&hit=1&index=&px=1) | ***623.9734*** | ***1245.9322*** |  |  |  |  |  |  |  |
|  | [1291](http://df3/mascot/cgi/peptide_view.pl?file=../data/20121119/F002569.dat&query=1291&hit=1&index=&px=1) | ***624.2042*** | ***1246.3939*** |  |  |  |  |  |  |  |
|  | [1292](http://df3/mascot/cgi/peptide_view.pl?file=../data/20121119/F002569.dat&query=1292&hit=1&index=&px=1) | ***624.2543*** | ***1246.4940*** |  |  |  |  |  |  |  |
|  | [1293](http://df3/mascot/cgi/peptide_view.pl?file=../data/20121119/F002569.dat&query=1293&hit=1&index=&px=1) | ***624.3180*** | ***1246.6214*** |  |  |  |  |  |  |  |
|  | [1294](http://df3/mascot/cgi/peptide_view.pl?file=../data/20121119/F002569.dat&query=1294&hit=1&index=&px=1) | ***624.4268*** | ***1246.8390*** |  |  |  |  |  |  |  |
|  | [1295](http://df3/mascot/cgi/peptide_view.pl?file=../data/20121119/F002569.dat&query=1295&hit=1&index=&px=1) | ***624.5254*** | ***1247.0362*** |  |  |  |  |  |  |  |
|  | [1296](http://df3/mascot/cgi/peptide_view.pl?file=../data/20121119/F002569.dat&query=1296&hit=1&index=&px=1) | ***624.7147*** | ***1247.4148*** |  |  |  |  |  |  |  |
|  | [1297](http://df3/mascot/cgi/peptide_view.pl?file=../data/20121119/F002569.dat&query=1297&hit=1&index=&px=1) | ***625.4152*** | ***1248.8158*** |  |  |  |  |  |  |  |
|  | [1298](http://df3/mascot/cgi/peptide_view.pl?file=../data/20121119/F002569.dat&query=1298&hit=1&index=&px=1) | ***625.5041*** | ***1248.9937*** |  |  |  |  |  |  |  |
|  | [1299](http://df3/mascot/cgi/peptide_view.pl?file=../data/20121119/F002569.dat&query=1299&hit=1&index=&px=1) | ***625.8806*** | ***1249.7467*** |  |  |  |  |  |  |  |
|  | [1300](http://df3/mascot/cgi/peptide_view.pl?file=../data/20121119/F002569.dat&query=1300&hit=1&index=&px=1) | ***626.0323*** | ***1250.0501*** |  |  |  |  |  |  |  |
|  | [1301](http://df3/mascot/cgi/peptide_view.pl?file=../data/20121119/F002569.dat&query=1301&hit=1&index=&px=1) | ***626.0879*** | ***1250.1612*** |  |  |  |  |  |  |  |
|  | [1302](http://df3/mascot/cgi/peptide_view.pl?file=../data/20121119/F002569.dat&query=1302&hit=1&index=&px=1) | ***626.1633*** | ***1250.3120*** |  |  |  |  |  |  |  |
|  | [1303](http://df3/mascot/cgi/peptide_view.pl?file=../data/20121119/F002569.dat&query=1303&hit=1&index=&px=1) | ***626.3843*** | ***1250.7541*** |  |  |  |  |  |  |  |
|  | [1304](http://df3/mascot/cgi/peptide_view.pl?file=../data/20121119/F002569.dat&query=1304&hit=1&index=&px=1) | ***626.5604*** | ***1251.1063*** |  |  |  |  |  |  |  |
|  | [1305](http://df3/mascot/cgi/peptide_view.pl?file=../data/20121119/F002569.dat&query=1305&hit=1&index=&px=1) | ***627.0140*** | ***1252.0135*** |  |  |  |  |  |  |  |
|  | [1306](http://df3/mascot/cgi/peptide_view.pl?file=../data/20121119/F002569.dat&query=1306&hit=1&index=&px=1) | ***627.2565*** | ***1252.4985*** |  |  |  |  |  |  |  |
|  | [1307](http://df3/mascot/cgi/peptide_view.pl?file=../data/20121119/F002569.dat&query=1307&hit=1&index=&px=1) | ***627.4178*** | ***1252.8210*** |  |  |  |  |  |  |  |
|  | [1308](http://df3/mascot/cgi/peptide_view.pl?file=../data/20121119/F002569.dat&query=1308&hit=1&index=&px=1) | ***627.6000*** | ***1253.1854*** |  |  |  |  |  |  |  |
|  | [1309](http://df3/mascot/cgi/peptide_view.pl?file=../data/20121119/F002569.dat&query=1309&hit=1&index=&px=1) | ***627.7234*** | ***1253.4323*** |  |  |  |  |  |  |  |
|  | [1310](http://df3/mascot/cgi/peptide_view.pl?file=../data/20121119/F002569.dat&query=1310&hit=1&index=&px=1) | ***628.6042*** | ***1255.1938*** |  |  |  |  |  |  |  |
|  | [1311](http://df3/mascot/cgi/peptide_view.pl?file=../data/20121119/F002569.dat&query=1311&hit=1&index=&px=1) | ***628.6251*** | ***1255.2357*** |  |  |  |  |  |  |  |
|  | [1312](http://df3/mascot/cgi/peptide_view.pl?file=../data/20121119/F002569.dat&query=1312&hit=1&index=&px=1) | ***628.6761*** | ***1255.3377*** |  |  |  |  |  |  |  |
|  | [1313](http://df3/mascot/cgi/peptide_view.pl?file=../data/20121119/F002569.dat&query=1313&hit=1&index=&px=1) | ***628.7538*** | ***1255.4930*** |  |  |  |  |  |  |  |
|  | [1314](http://df3/mascot/cgi/peptide_view.pl?file=../data/20121119/F002569.dat&query=1314&hit=1&index=&px=1) | ***629.1897*** | ***1256.3648*** |  |  |  |  |  |  |  |
|  | [1315](http://df3/mascot/cgi/peptide_view.pl?file=../data/20121119/F002569.dat&query=1315&hit=1&index=&px=1) | ***629.8364*** | ***1257.6583*** |  |  |  |  |  |  |  |
|  | [1316](http://df3/mascot/cgi/peptide_view.pl?file=../data/20121119/F002569.dat&query=1316&hit=1&index=&px=1) | ***630.3057*** | ***1258.5968*** |  |  |  |  |  |  |  |
|  | [1317](http://df3/mascot/cgi/peptide_view.pl?file=../data/20121119/F002569.dat&query=1317&hit=1&index=&px=1) | ***630.3396*** | ***1258.6646*** |  |  |  |  |  |  |  |
|  | [1318](http://df3/mascot/cgi/peptide_view.pl?file=../data/20121119/F002569.dat&query=1318&hit=1&index=&px=1) | ***630.3785*** | ***1258.7425*** |  |  |  |  |  |  |  |
|  | [1319](http://df3/mascot/cgi/peptide_view.pl?file=../data/20121119/F002569.dat&query=1319&hit=1&index=&px=1) | ***630.6880*** | ***1259.3614*** |  |  |  |  |  |  |  |
|  | [1320](http://df3/mascot/cgi/peptide_view.pl?file=../data/20121119/F002569.dat&query=1320&hit=1&index=&px=1) | ***630.7758*** | ***1259.5370*** |  |  |  |  |  |  |  |
|  | [1321](http://df3/mascot/cgi/peptide_view.pl?file=../data/20121119/F002569.dat&query=1321&hit=1&index=&px=1) | ***630.8439*** | ***1259.6732*** |  |  |  |  |  |  |  |
|  | [1323](http://df3/mascot/cgi/peptide_view.pl?file=../data/20121119/F002569.dat&query=1323&hit=1&index=&px=1) | ***630.9077*** | ***1259.8009*** |  |  |  |  |  |  |  |
|  | [1324](http://df3/mascot/cgi/peptide_view.pl?file=../data/20121119/F002569.dat&query=1324&hit=1&index=&px=1) | ***630.9894*** | ***1259.9642*** |  |  |  |  |  |  |  |
|  | [1325](http://df3/mascot/cgi/peptide_view.pl?file=../data/20121119/F002569.dat&query=1325&hit=1&index=&px=1) | ***631.1161*** | ***1260.2177*** |  |  |  |  |  |  |  |
|  | [1327](http://df3/mascot/cgi/peptide_view.pl?file=../data/20121119/F002569.dat&query=1327&hit=1&index=&px=1) | ***631.4517*** | ***1260.8888*** |  |  |  |  |  |  |  |
|  | [1329](http://df3/mascot/cgi/peptide_view.pl?file=../data/20121119/F002569.dat&query=1329&hit=1&index=&px=1) | ***631.8258*** | ***1261.6371*** |  |  |  |  |  |  |  |
|  | [1331](http://df3/mascot/cgi/peptide_view.pl?file=../data/20121119/F002569.dat&query=1331&hit=1&index=&px=1) | ***632.6100*** | ***1263.2054*** |  |  |  |  |  |  |  |
|  | [1332](http://df3/mascot/cgi/peptide_view.pl?file=../data/20121119/F002569.dat&query=1332&hit=1&index=&px=1) | ***634.3324*** | ***1266.6502*** |  |  |  |  |  |  |  |
|  | [1333](http://df3/mascot/cgi/peptide_view.pl?file=../data/20121119/F002569.dat&query=1333&hit=1&index=&px=1) | ***634.5433*** | ***1267.0721*** |  |  |  |  |  |  |  |
|  | [1334](http://df3/mascot/cgi/peptide_view.pl?file=../data/20121119/F002569.dat&query=1334&hit=1&index=&px=1) | ***634.5628*** | ***1267.1111*** |  |  |  |  |  |  |  |
|  | [1335](http://df3/mascot/cgi/peptide_view.pl?file=../data/20121119/F002569.dat&query=1335&hit=1&index=&px=1) | ***635.3426*** | ***1268.6706*** |  |  |  |  |  |  |  |
|  | [1336](http://df3/mascot/cgi/peptide_view.pl?file=../data/20121119/F002569.dat&query=1336&hit=1&index=&px=1) | ***636.5201*** | ***1271.0257*** |  |  |  |  |  |  |  |
|  | [1337](http://df3/mascot/cgi/peptide_view.pl?file=../data/20121119/F002569.dat&query=1337&hit=1&index=&px=1) | ***637.0638*** | ***1272.1131*** |  |  |  |  |  |  |  |
|  | [1338](http://df3/mascot/cgi/peptide_view.pl?file=../data/20121119/F002569.dat&query=1338&hit=1&index=&px=1) | ***637.5325*** | ***1273.0504*** |  |  |  |  |  |  |  |
|  | [1339](http://df3/mascot/cgi/peptide_view.pl?file=../data/20121119/F002569.dat&query=1339&hit=1&index=&px=1) | ***637.7787*** | ***1273.5429*** |  |  |  |  |  |  |  |
|  | [1340](http://df3/mascot/cgi/peptide_view.pl?file=../data/20121119/F002569.dat&query=1340&hit=1&index=&px=1) | ***638.1064*** | ***1274.1983*** |  |  |  |  |  |  |  |
|  | [1341](http://df3/mascot/cgi/peptide_view.pl?file=../data/20121119/F002569.dat&query=1341&hit=1&index=&px=1) | ***638.9125*** | ***1275.8104*** |  |  |  |  |  |  |  |
|  | [1342](http://df3/mascot/cgi/peptide_view.pl?file=../data/20121119/F002569.dat&query=1342&hit=1&index=&px=1) | ***639.6379*** | ***1277.2613*** |  |  |  |  |  |  |  |
|  | [1343](http://df3/mascot/cgi/peptide_view.pl?file=../data/20121119/F002569.dat&query=1343&hit=1&index=&px=1) | ***641.0237*** | ***1280.0328*** |  |  |  |  |  |  |  |
|  | [1344](http://df3/mascot/cgi/peptide_view.pl?file=../data/20121119/F002569.dat&query=1344&hit=1&index=&px=1) | ***641.4575*** | ***1280.9005*** |  |  |  |  |  |  |  |
|  | [1345](http://df3/mascot/cgi/peptide_view.pl?file=../data/20121119/F002569.dat&query=1345&hit=1&index=&px=1) | ***642.0874*** | ***1282.1603*** |  |  |  |  |  |  |  |
|  | [1354](http://df3/mascot/cgi/peptide_view.pl?file=../data/20121119/F002569.dat&query=1354&hit=1&index=&px=1) | ***643.9180*** | ***1285.8215*** |  |  |  |  |  |  |  |
|  | [1355](http://df3/mascot/cgi/peptide_view.pl?file=../data/20121119/F002569.dat&query=1355&hit=1&index=&px=1) | ***644.0645*** | ***1286.1144*** |  |  |  |  |  |  |  |
|  | [1356](http://df3/mascot/cgi/peptide_view.pl?file=../data/20121119/F002569.dat&query=1356&hit=1&index=&px=1) | ***644.4034*** | ***1286.7922*** |  |  |  |  |  |  |  |
|  | [1357](http://df3/mascot/cgi/peptide_view.pl?file=../data/20121119/F002569.dat&query=1357&hit=1&index=&px=1) | ***644.5906*** | ***1287.1666*** |  |  |  |  |  |  |  |
|  | [1358](http://df3/mascot/cgi/peptide_view.pl?file=../data/20121119/F002569.dat&query=1358&hit=1&index=&px=1) | ***644.9697*** | ***1287.9249*** |  |  |  |  |  |  |  |
|  | [1359](http://df3/mascot/cgi/peptide_view.pl?file=../data/20121119/F002569.dat&query=1359&hit=1&index=&px=1) | ***645.0438*** | ***1288.0731*** |  |  |  |  |  |  |  |
|  | [1360](http://df3/mascot/cgi/peptide_view.pl?file=../data/20121119/F002569.dat&query=1360&hit=1&index=&px=1) | ***645.3674*** | ***1288.7203*** |  |  |  |  |  |  |  |
|  | [1361](http://df3/mascot/cgi/peptide_view.pl?file=../data/20121119/F002569.dat&query=1361&hit=1&index=&px=1) | ***645.8777*** | ***1289.7409*** |  |  |  |  |  |  |  |
|  | [1362](http://df3/mascot/cgi/peptide_view.pl?file=../data/20121119/F002569.dat&query=1362&hit=1&index=&px=1) | ***646.0255*** | ***1290.0365*** |  |  |  |  |  |  |  |
|  | [1363](http://df3/mascot/cgi/peptide_view.pl?file=../data/20121119/F002569.dat&query=1363&hit=1&index=&px=1) | ***646.2368*** | ***1290.4591*** |  |  |  |  |  |  |  |
|  | [1364](http://df3/mascot/cgi/peptide_view.pl?file=../data/20121119/F002569.dat&query=1364&hit=1&index=&px=1) | ***646.3112*** | ***1290.6078*** |  |  |  |  |  |  |  |
|  | [1365](http://df3/mascot/cgi/peptide_view.pl?file=../data/20121119/F002569.dat&query=1365&hit=1&index=&px=1) | ***646.3986*** | ***1290.7826*** |  |  |  |  |  |  |  |
|  | [1366](http://df3/mascot/cgi/peptide_view.pl?file=../data/20121119/F002569.dat&query=1366&hit=1&index=&px=1) | ***647.1614*** | ***1292.3082*** |  |  |  |  |  |  |  |
|  | [1367](http://df3/mascot/cgi/peptide_view.pl?file=../data/20121119/F002569.dat&query=1367&hit=1&index=&px=1) | ***647.3293*** | ***1292.6441*** |  |  |  |  |  |  |  |
|  | [1368](http://df3/mascot/cgi/peptide_view.pl?file=../data/20121119/F002569.dat&query=1368&hit=1&index=&px=1) | ***647.3799*** | ***1292.7452*** |  |  |  |  |  |  |  |
|  | [1369](http://df3/mascot/cgi/peptide_view.pl?file=../data/20121119/F002569.dat&query=1369&hit=1&index=&px=1) | ***647.4084*** | ***1292.8023*** |  |  |  |  |  |  |  |
|  | [1370](http://df3/mascot/cgi/peptide_view.pl?file=../data/20121119/F002569.dat&query=1370&hit=1&index=&px=1) | ***647.4449*** | ***1292.8753*** |  |  |  |  |  |  |  |
|  | [1371](http://df3/mascot/cgi/peptide_view.pl?file=../data/20121119/F002569.dat&query=1371&hit=1&index=&px=1) | ***649.4239*** | ***1296.8333*** |  |  |  |  |  |  |  |
|  | [1372](http://df3/mascot/cgi/peptide_view.pl?file=../data/20121119/F002569.dat&query=1372&hit=1&index=&px=1) | ***649.6431*** | ***1297.2716*** |  |  |  |  |  |  |  |
|  | [1373](http://df3/mascot/cgi/peptide_view.pl?file=../data/20121119/F002569.dat&query=1373&hit=1&index=&px=1) | ***650.0078*** | ***1298.0011*** |  |  |  |  |  |  |  |
|  | [1374](http://df3/mascot/cgi/peptide_view.pl?file=../data/20121119/F002569.dat&query=1374&hit=1&index=&px=1) | ***650.5507*** | ***1299.0868*** |  |  |  |  |  |  |  |
|  | [1375](http://df3/mascot/cgi/peptide_view.pl?file=../data/20121119/F002569.dat&query=1375&hit=1&index=&px=1) | ***650.5634*** | ***1299.1122*** |  |  |  |  |  |  |  |
|  | [1376](http://df3/mascot/cgi/peptide_view.pl?file=../data/20121119/F002569.dat&query=1376&hit=1&index=&px=1) | ***650.6512*** | ***1299.2878*** |  |  |  |  |  |  |  |
|  | [1377](http://df3/mascot/cgi/peptide_view.pl?file=../data/20121119/F002569.dat&query=1377&hit=1&index=&px=1) | ***650.6571*** | ***1299.2997*** |  |  |  |  |  |  |  |
|  | [1378](http://df3/mascot/cgi/peptide_view.pl?file=../data/20121119/F002569.dat&query=1378&hit=1&index=&px=1) | ***650.9210*** | ***1299.8275*** |  |  |  |  |  |  |  |
|  | [1379](http://df3/mascot/cgi/peptide_view.pl?file=../data/20121119/F002569.dat&query=1379&hit=1&index=&px=1) | ***650.9683*** | ***1299.9220*** |  |  |  |  |  |  |  |
|  | [1380](http://df3/mascot/cgi/peptide_view.pl?file=../data/20121119/F002569.dat&query=1380&hit=1&index=&px=1) | ***651.8969*** | ***1301.7792*** |  |  |  |  |  |  |  |
|  | [1381](http://df3/mascot/cgi/peptide_view.pl?file=../data/20121119/F002569.dat&query=1381&hit=1&index=&px=1) | ***651.9734*** | ***1301.9322*** |  |  |  |  |  |  |  |
|  | [1382](http://df3/mascot/cgi/peptide_view.pl?file=../data/20121119/F002569.dat&query=1382&hit=1&index=&px=1) | ***652.0055*** | ***1301.9964*** |  |  |  |  |  |  |  |
|  | [1383](http://df3/mascot/cgi/peptide_view.pl?file=../data/20121119/F002569.dat&query=1383&hit=1&index=&px=1) | ***652.2388*** | ***1302.4631*** |  |  |  |  |  |  |  |
|  | [1384](http://df3/mascot/cgi/peptide_view.pl?file=../data/20121119/F002569.dat&query=1384&hit=1&index=&px=1) | ***652.4949*** | ***1302.9752*** |  |  |  |  |  |  |  |
|  | [1385](http://df3/mascot/cgi/peptide_view.pl?file=../data/20121119/F002569.dat&query=1385&hit=1&index=&px=1) | ***652.5160*** | ***1303.0174*** |  |  |  |  |  |  |  |
|  | [1386](http://df3/mascot/cgi/peptide_view.pl?file=../data/20121119/F002569.dat&query=1386&hit=1&index=&px=1) | ***652.5818*** | ***1303.1490*** |  |  |  |  |  |  |  |
|  | [1387](http://df3/mascot/cgi/peptide_view.pl?file=../data/20121119/F002569.dat&query=1387&hit=1&index=&px=1) | ***652.5890*** | ***1303.1634*** |  |  |  |  |  |  |  |
|  | [1388](http://df3/mascot/cgi/peptide_view.pl?file=../data/20121119/F002569.dat&query=1388&hit=1&index=&px=1) | ***652.6836*** | ***1303.3526*** |  |  |  |  |  |  |  |
|  | [1389](http://df3/mascot/cgi/peptide_view.pl?file=../data/20121119/F002569.dat&query=1389&hit=1&index=&px=1) | ***653.1864*** | ***1304.3583*** |  |  |  |  |  |  |  |
|  | [1390](http://df3/mascot/cgi/peptide_view.pl?file=../data/20121119/F002569.dat&query=1390&hit=1&index=&px=1) | ***653.5974*** | ***1305.1803*** |  |  |  |  |  |  |  |
|  | [1391](http://df3/mascot/cgi/peptide_view.pl?file=../data/20121119/F002569.dat&query=1391&hit=1&index=&px=1) | ***653.8435*** | ***1305.6725*** |  |  |  |  |  |  |  |
|  | [1392](http://df3/mascot/cgi/peptide_view.pl?file=../data/20121119/F002569.dat&query=1392&hit=1&index=&px=1) | ***654.1121*** | ***1306.2096*** |  |  |  |  |  |  |  |
|  | [1393](http://df3/mascot/cgi/peptide_view.pl?file=../data/20121119/F002569.dat&query=1393&hit=1&index=&px=1) | ***655.7546*** | ***1309.4947*** |  |  |  |  |  |  |  |
|  | [1394](http://df3/mascot/cgi/peptide_view.pl?file=../data/20121119/F002569.dat&query=1394&hit=1&index=&px=1) | ***655.7739*** | ***1309.5333*** |  |  |  |  |  |  |  |
|  | [1395](http://df3/mascot/cgi/peptide_view.pl?file=../data/20121119/F002569.dat&query=1395&hit=1&index=&px=1) | ***655.8348*** | ***1309.6551*** |  |  |  |  |  |  |  |
|  | [1396](http://df3/mascot/cgi/peptide_view.pl?file=../data/20121119/F002569.dat&query=1396&hit=1&index=&px=1) | ***655.8820*** | ***1309.7494*** |  |  |  |  |  |  |  |
|  | [1397](http://df3/mascot/cgi/peptide_view.pl?file=../data/20121119/F002569.dat&query=1397&hit=1&index=&px=1) | ***656.1759*** | ***1310.3373*** |  |  |  |  |  |  |  |
|  | [1398](http://df3/mascot/cgi/peptide_view.pl?file=../data/20121119/F002569.dat&query=1398&hit=1&index=&px=1) | ***656.9237*** | ***1311.8329*** |  |  |  |  |  |  |  |
|  | [1399](http://df3/mascot/cgi/peptide_view.pl?file=../data/20121119/F002569.dat&query=1399&hit=1&index=&px=1) | ***657.3591*** | ***1312.7037*** |  |  |  |  |  |  |  |
|  | [1400](http://df3/mascot/cgi/peptide_view.pl?file=../data/20121119/F002569.dat&query=1400&hit=1&index=&px=1) | ***657.6563*** | ***1313.2979*** |  |  |  |  |  |  |  |
|  | [1401](http://df3/mascot/cgi/peptide_view.pl?file=../data/20121119/F002569.dat&query=1401&hit=1&index=&px=1) | ***657.9375*** | ***1313.8604*** |  |  |  |  |  |  |  |
|  | [1402](http://df3/mascot/cgi/peptide_view.pl?file=../data/20121119/F002569.dat&query=1402&hit=1&index=&px=1) | ***658.0175*** | ***1314.0204*** |  |  |  |  |  |  |  |
|  | [1403](http://df3/mascot/cgi/peptide_view.pl?file=../data/20121119/F002569.dat&query=1403&hit=1&index=&px=1) | ***658.4286*** | ***1314.8426*** |  |  |  |  |  |  |  |
|  | [1404](http://df3/mascot/cgi/peptide_view.pl?file=../data/20121119/F002569.dat&query=1404&hit=1&index=&px=1) | ***658.6085*** | ***1315.2025*** |  |  |  |  |  |  |  |
|  | [1405](http://df3/mascot/cgi/peptide_view.pl?file=../data/20121119/F002569.dat&query=1405&hit=1&index=&px=1) | ***658.6638*** | ***1315.3130*** |  |  |  |  |  |  |  |
|  | [1406](http://df3/mascot/cgi/peptide_view.pl?file=../data/20121119/F002569.dat&query=1406&hit=1&index=&px=1) | ***659.0433*** | ***1316.0721*** |  |  |  |  |  |  |  |
|  | [1407](http://df3/mascot/cgi/peptide_view.pl?file=../data/20121119/F002569.dat&query=1407&hit=1&index=&px=1) | ***659.0859*** | ***1316.1572*** |  |  |  |  |  |  |  |
|  | [1408](http://df3/mascot/cgi/peptide_view.pl?file=../data/20121119/F002569.dat&query=1408&hit=1&index=&px=1) | ***659.1501*** | ***1316.2857*** |  |  |  |  |  |  |  |
|  | [1409](http://df3/mascot/cgi/peptide_view.pl?file=../data/20121119/F002569.dat&query=1409&hit=1&index=&px=1) | ***659.3818*** | ***1316.7491*** |  |  |  |  |  |  |  |
|  | [1410](http://df3/mascot/cgi/peptide_view.pl?file=../data/20121119/F002569.dat&query=1410&hit=1&index=&px=1) | ***659.5978*** | ***1317.1811*** |  |  |  |  |  |  |  |
|  | [1411](http://df3/mascot/cgi/peptide_view.pl?file=../data/20121119/F002569.dat&query=1411&hit=1&index=&px=1) | ***659.9708*** | ***1317.9271*** |  |  |  |  |  |  |  |
|  | [1412](http://df3/mascot/cgi/peptide_view.pl?file=../data/20121119/F002569.dat&query=1412&hit=1&index=&px=1) | ***660.0637*** | ***1318.1129*** |  |  |  |  |  |  |  |
|  | [1413](http://df3/mascot/cgi/peptide_view.pl?file=../data/20121119/F002569.dat&query=1413&hit=1&index=&px=1) | ***660.6971*** | ***1319.3796*** |  |  |  |  |  |  |  |
|  | [1414](http://df3/mascot/cgi/peptide_view.pl?file=../data/20121119/F002569.dat&query=1414&hit=1&index=&px=1) | ***660.7711*** | ***1319.5276*** |  |  |  |  |  |  |  |
|  | [1415](http://df3/mascot/cgi/peptide_view.pl?file=../data/20121119/F002569.dat&query=1415&hit=1&index=&px=1) | ***661.5585*** | ***1321.1024*** |  |  |  |  |  |  |  |
|  | [1416](http://df3/mascot/cgi/peptide_view.pl?file=../data/20121119/F002569.dat&query=1416&hit=1&index=&px=1) | ***662.4319*** | ***1322.8492*** |  |  |  |  |  |  |  |
|  | [1417](http://df3/mascot/cgi/peptide_view.pl?file=../data/20121119/F002569.dat&query=1417&hit=1&index=&px=1) | ***662.5858*** | ***1323.1571*** |  |  |  |  |  |  |  |
|  | [1418](http://df3/mascot/cgi/peptide_view.pl?file=../data/20121119/F002569.dat&query=1418&hit=1&index=&px=1) | ***662.9237*** | ***1323.8329*** |  |  |  |  |  |  |  |
|  | [1419](http://df3/mascot/cgi/peptide_view.pl?file=../data/20121119/F002569.dat&query=1419&hit=1&index=&px=1) | ***663.0226*** | ***1324.0306*** |  |  |  |  |  |  |  |
|  | [1420](http://df3/mascot/cgi/peptide_view.pl?file=../data/20121119/F002569.dat&query=1420&hit=1&index=&px=1) | ***663.2063*** | ***1324.3980*** |  |  |  |  |  |  |  |
|  | [1421](http://df3/mascot/cgi/peptide_view.pl?file=../data/20121119/F002569.dat&query=1421&hit=1&index=&px=1) | ***663.5659*** | ***1325.1173*** |  |  |  |  |  |  |  |
|  | [1422](http://df3/mascot/cgi/peptide_view.pl?file=../data/20121119/F002569.dat&query=1422&hit=1&index=&px=1) | ***663.5684*** | ***1325.1222*** |  |  |  |  |  |  |  |
|  | [1423](http://df3/mascot/cgi/peptide_view.pl?file=../data/20121119/F002569.dat&query=1423&hit=1&index=&px=1) | ***663.6123*** | ***1325.2101*** |  |  |  |  |  |  |  |
|  | [1424](http://df3/mascot/cgi/peptide_view.pl?file=../data/20121119/F002569.dat&query=1424&hit=1&index=&px=1) | ***663.6151*** | ***1325.2157*** |  |  |  |  |  |  |  |
|  | [1425](http://df3/mascot/cgi/peptide_view.pl?file=../data/20121119/F002569.dat&query=1425&hit=1&index=&px=1) | ***663.7233*** | ***1325.4320*** |  |  |  |  |  |  |  |
|  | [1426](http://df3/mascot/cgi/peptide_view.pl?file=../data/20121119/F002569.dat&query=1426&hit=1&index=&px=1) | ***664.0654*** | ***1326.1163*** |  |  |  |  |  |  |  |
|  | [1427](http://df3/mascot/cgi/peptide_view.pl?file=../data/20121119/F002569.dat&query=1427&hit=1&index=&px=1) | ***664.0903*** | ***1326.1661*** |  |  |  |  |  |  |  |
|  | [1428](http://df3/mascot/cgi/peptide_view.pl?file=../data/20121119/F002569.dat&query=1428&hit=1&index=&px=1) | ***664.1877*** | ***1326.3609*** |  |  |  |  |  |  |  |
|  | [1429](http://df3/mascot/cgi/peptide_view.pl?file=../data/20121119/F002569.dat&query=1429&hit=1&index=&px=1) | ***664.3504*** | ***1326.6863*** |  |  |  |  |  |  |  |
|  | [1430](http://df3/mascot/cgi/peptide_view.pl?file=../data/20121119/F002569.dat&query=1430&hit=1&index=&px=1) | ***664.4224*** | ***1326.8302*** |  |  |  |  |  |  |  |
|  | [1431](http://df3/mascot/cgi/peptide_view.pl?file=../data/20121119/F002569.dat&query=1431&hit=1&index=&px=1) | ***664.5946*** | ***1327.1747*** |  |  |  |  |  |  |  |
|  | [1432](http://df3/mascot/cgi/peptide_view.pl?file=../data/20121119/F002569.dat&query=1432&hit=1&index=&px=1) | ***664.7253*** | ***1327.4361*** |  |  |  |  |  |  |  |
|  | [1433](http://df3/mascot/cgi/peptide_view.pl?file=../data/20121119/F002569.dat&query=1433&hit=1&index=&px=1) | ***665.0961*** | ***1328.1776*** |  |  |  |  |  |  |  |
|  | [1434](http://df3/mascot/cgi/peptide_view.pl?file=../data/20121119/F002569.dat&query=1434&hit=1&index=&px=1) | ***665.3424*** | ***1328.6703*** |  |  |  |  |  |  |  |
|  | [1435](http://df3/mascot/cgi/peptide_view.pl?file=../data/20121119/F002569.dat&query=1435&hit=1&index=&px=1) | ***665.4103*** | ***1328.8061*** |  |  |  |  |  |  |  |
|  | [1436](http://df3/mascot/cgi/peptide_view.pl?file=../data/20121119/F002569.dat&query=1436&hit=1&index=&px=1) | ***666.2499*** | ***1330.4852*** |  |  |  |  |  |  |  |
|  | [1437](http://df3/mascot/cgi/peptide_view.pl?file=../data/20121119/F002569.dat&query=1437&hit=1&index=&px=1) | ***666.5624*** | ***1331.1102*** |  |  |  |  |  |  |  |
|  | [1438](http://df3/mascot/cgi/peptide_view.pl?file=../data/20121119/F002569.dat&query=1438&hit=1&index=&px=1) | ***666.6411*** | ***1331.2677*** |  |  |  |  |  |  |  |
|  | [1439](http://df3/mascot/cgi/peptide_view.pl?file=../data/20121119/F002569.dat&query=1439&hit=1&index=&px=1) | ***667.1649*** | ***1332.3153*** |  |  |  |  |  |  |  |
|  | [1440](http://df3/mascot/cgi/peptide_view.pl?file=../data/20121119/F002569.dat&query=1440&hit=1&index=&px=1) | ***667.3530*** | ***1332.6915*** |  |  |  |  |  |  |  |
|  | [1441](http://df3/mascot/cgi/peptide_view.pl?file=../data/20121119/F002569.dat&query=1441&hit=1&index=&px=1) | ***667.4620*** | ***1332.9095*** |  |  |  |  |  |  |  |
|  | [1442](http://df3/mascot/cgi/peptide_view.pl?file=../data/20121119/F002569.dat&query=1442&hit=1&index=&px=1) | ***667.5071*** | ***1332.9996*** |  |  |  |  |  |  |  |
|  | [1443](http://df3/mascot/cgi/peptide_view.pl?file=../data/20121119/F002569.dat&query=1443&hit=1&index=&px=1) | ***667.6748*** | ***1333.3351*** |  |  |  |  |  |  |  |
|  | [1445](http://df3/mascot/cgi/peptide_view.pl?file=../data/20121119/F002569.dat&query=1445&hit=1&index=&px=1) | ***667.7047*** | ***1333.3949*** |  |  |  |  |  |  |  |
|  | [1446](http://df3/mascot/cgi/peptide_view.pl?file=../data/20121119/F002569.dat&query=1446&hit=1&index=&px=1) | ***667.7516*** | ***1333.4886*** |  |  |  |  |  |  |  |
|  | [1447](http://df3/mascot/cgi/peptide_view.pl?file=../data/20121119/F002569.dat&query=1447&hit=1&index=&px=1) | ***668.5363*** | ***1335.0580*** |  |  |  |  |  |  |  |
|  | [1448](http://df3/mascot/cgi/peptide_view.pl?file=../data/20121119/F002569.dat&query=1448&hit=1&index=&px=1) | ***668.7797*** | ***1335.5448*** |  |  |  |  |  |  |  |
|  | [1449](http://df3/mascot/cgi/peptide_view.pl?file=../data/20121119/F002569.dat&query=1449&hit=1&index=&px=1) | ***668.9896*** | ***1335.9647*** |  |  |  |  |  |  |  |
|  | [1450](http://df3/mascot/cgi/peptide_view.pl?file=../data/20121119/F002569.dat&query=1450&hit=1&index=&px=1) | ***669.2239*** | ***1336.4333*** |  |  |  |  |  |  |  |
|  | [1451](http://df3/mascot/cgi/peptide_view.pl?file=../data/20121119/F002569.dat&query=1451&hit=1&index=&px=1) | ***669.5247*** | ***1337.0348*** |  |  |  |  |  |  |  |
|  | [1452](http://df3/mascot/cgi/peptide_view.pl?file=../data/20121119/F002569.dat&query=1452&hit=1&index=&px=1) | ***670.1035*** | ***1338.1925*** |  |  |  |  |  |  |  |
|  | [1453](http://df3/mascot/cgi/peptide_view.pl?file=../data/20121119/F002569.dat&query=1453&hit=1&index=&px=1) | ***670.1179*** | ***1338.2213*** |  |  |  |  |  |  |  |
|  | [1454](http://df3/mascot/cgi/peptide_view.pl?file=../data/20121119/F002569.dat&query=1454&hit=1&index=&px=1) | ***670.6044*** | ***1339.1942*** |  |  |  |  |  |  |  |
|  | [1455](http://df3/mascot/cgi/peptide_view.pl?file=../data/20121119/F002569.dat&query=1455&hit=1&index=&px=1) | ***670.7074*** | ***1339.4002*** |  |  |  |  |  |  |  |
|  | [1456](http://df3/mascot/cgi/peptide_view.pl?file=../data/20121119/F002569.dat&query=1456&hit=1&index=&px=1) | ***670.8162*** | ***1339.6179*** |  |  |  |  |  |  |  |
|  | [1457](http://df3/mascot/cgi/peptide_view.pl?file=../data/20121119/F002569.dat&query=1457&hit=1&index=&px=1) | ***670.9215*** | ***1339.8285*** |  |  |  |  |  |  |  |
|  | [1458](http://df3/mascot/cgi/peptide_view.pl?file=../data/20121119/F002569.dat&query=1458&hit=1&index=&px=1) | ***670.9369*** | ***1339.8592*** |  |  |  |  |  |  |  |
|  | [1459](http://df3/mascot/cgi/peptide_view.pl?file=../data/20121119/F002569.dat&query=1459&hit=1&index=&px=1) | ***670.9375*** | ***1339.8604*** |  |  |  |  |  |  |  |
|  | [1460](http://df3/mascot/cgi/peptide_view.pl?file=../data/20121119/F002569.dat&query=1460&hit=1&index=&px=1) | ***671.1666*** | ***1340.3187*** |  |  |  |  |  |  |  |
|  | [1461](http://df3/mascot/cgi/peptide_view.pl?file=../data/20121119/F002569.dat&query=1461&hit=1&index=&px=1) | ***671.2286*** | ***1340.4426*** |  |  |  |  |  |  |  |
|  | [1462](http://df3/mascot/cgi/peptide_view.pl?file=../data/20121119/F002569.dat&query=1462&hit=1&index=&px=1) | ***671.3207*** | ***1340.6268*** |  |  |  |  |  |  |  |
|  | [1463](http://df3/mascot/cgi/peptide_view.pl?file=../data/20121119/F002569.dat&query=1463&hit=1&index=&px=1) | ***671.4114*** | ***1340.8082*** |  |  |  |  |  |  |  |
|  | [1464](http://df3/mascot/cgi/peptide_view.pl?file=../data/20121119/F002569.dat&query=1464&hit=1&index=&px=1) | ***671.4480*** | ***1340.8814*** |  |  |  |  |  |  |  |
|  | [1465](http://df3/mascot/cgi/peptide_view.pl?file=../data/20121119/F002569.dat&query=1465&hit=1&index=&px=1) | ***671.5076*** | ***1341.0006*** |  |  |  |  |  |  |  |
|  | [1466](http://df3/mascot/cgi/peptide_view.pl?file=../data/20121119/F002569.dat&query=1466&hit=1&index=&px=1) | ***671.6730*** | ***1341.3315*** |  |  |  |  |  |  |  |
|  | [1467](http://df3/mascot/cgi/peptide_view.pl?file=../data/20121119/F002569.dat&query=1467&hit=1&index=&px=1) | ***672.1401*** | ***1342.2657*** |  |  |  |  |  |  |  |
|  | [1476](http://df3/mascot/cgi/peptide_view.pl?file=../data/20121119/F002569.dat&query=1476&hit=1&index=&px=1) | ***673.7903*** | ***1345.5660*** |  |  |  |  |  |  |  |
|  | [1477](http://df3/mascot/cgi/peptide_view.pl?file=../data/20121119/F002569.dat&query=1477&hit=1&index=&px=1) | ***673.8719*** | ***1345.7293*** |  |  |  |  |  |  |  |
|  | [1478](http://df3/mascot/cgi/peptide_view.pl?file=../data/20121119/F002569.dat&query=1478&hit=1&index=&px=1) | ***674.1010*** | ***1346.1874*** |  |  |  |  |  |  |  |
|  | [1479](http://df3/mascot/cgi/peptide_view.pl?file=../data/20121119/F002569.dat&query=1479&hit=1&index=&px=1) | ***674.4100*** | ***1346.8055*** |  |  |  |  |  |  |  |
|  | [1480](http://df3/mascot/cgi/peptide_view.pl?file=../data/20121119/F002569.dat&query=1480&hit=1&index=&px=1) | ***674.5482*** | ***1347.0819*** |  |  |  |  |  |  |  |
|  | [1481](http://df3/mascot/cgi/peptide_view.pl?file=../data/20121119/F002569.dat&query=1481&hit=1&index=&px=1) | ***675.0946*** | ***1348.1747*** |  |  |  |  |  |  |  |
|  | [1482](http://df3/mascot/cgi/peptide_view.pl?file=../data/20121119/F002569.dat&query=1482&hit=1&index=&px=1) | ***675.1624*** | ***1348.3103*** |  |  |  |  |  |  |  |
|  | [1483](http://df3/mascot/cgi/peptide_view.pl?file=../data/20121119/F002569.dat&query=1483&hit=1&index=&px=1) | ***675.2700*** | ***1348.5255*** |  |  |  |  |  |  |  |
|  | [1484](http://df3/mascot/cgi/peptide_view.pl?file=../data/20121119/F002569.dat&query=1484&hit=1&index=&px=1) | ***675.7488*** | ***1349.4830*** |  |  |  |  |  |  |  |
|  | [1485](http://df3/mascot/cgi/peptide_view.pl?file=../data/20121119/F002569.dat&query=1485&hit=1&index=&px=1) | ***676.4689*** | ***1350.9232*** |  |  |  |  |  |  |  |
|  | [1486](http://df3/mascot/cgi/peptide_view.pl?file=../data/20121119/F002569.dat&query=1486&hit=1&index=&px=1) | ***676.5424*** | ***1351.0702*** |  |  |  |  |  |  |  |
|  | [1487](http://df3/mascot/cgi/peptide_view.pl?file=../data/20121119/F002569.dat&query=1487&hit=1&index=&px=1) | ***677.1676*** | ***1352.3207*** |  |  |  |  |  |  |  |
|  | [1488](http://df3/mascot/cgi/peptide_view.pl?file=../data/20121119/F002569.dat&query=1488&hit=1&index=&px=1) | ***677.6390*** | ***1353.2634*** |  |  |  |  |  |  |  |
|  | [1489](http://df3/mascot/cgi/peptide_view.pl?file=../data/20121119/F002569.dat&query=1489&hit=1&index=&px=1) | ***678.2064*** | ***1354.3982*** |  |  |  |  |  |  |  |
|  | [1490](http://df3/mascot/cgi/peptide_view.pl?file=../data/20121119/F002569.dat&query=1490&hit=1&index=&px=1) | ***678.9978*** | ***1355.9811*** |  |  |  |  |  |  |  |
|  | [1491](http://df3/mascot/cgi/peptide_view.pl?file=../data/20121119/F002569.dat&query=1491&hit=1&index=&px=1) | ***679.0211*** | ***1356.0277*** |  |  |  |  |  |  |  |
|  | [1492](http://df3/mascot/cgi/peptide_view.pl?file=../data/20121119/F002569.dat&query=1492&hit=1&index=&px=1) | ***679.1274*** | ***1356.2403*** |  |  |  |  |  |  |  |
|  | [1493](http://df3/mascot/cgi/peptide_view.pl?file=../data/20121119/F002569.dat&query=1493&hit=1&index=&px=1) | ***679.6144*** | ***1357.2142*** |  |  |  |  |  |  |  |
|  | [1494](http://df3/mascot/cgi/peptide_view.pl?file=../data/20121119/F002569.dat&query=1494&hit=1&index=&px=1) | ***680.0736*** | ***1358.1327*** |  |  |  |  |  |  |  |
|  | [1495](http://df3/mascot/cgi/peptide_view.pl?file=../data/20121119/F002569.dat&query=1495&hit=1&index=&px=1) | ***680.3644*** | ***1358.7142*** |  |  |  |  |  |  |  |
|  | [1496](http://df3/mascot/cgi/peptide_view.pl?file=../data/20121119/F002569.dat&query=1496&hit=1&index=&px=1) | ***681.0476*** | ***1360.0807*** |  |  |  |  |  |  |  |
|  | [1497](http://df3/mascot/cgi/peptide_view.pl?file=../data/20121119/F002569.dat&query=1497&hit=1&index=&px=1) | ***681.0911*** | ***1360.1676*** |  |  |  |  |  |  |  |
|  | [1498](http://df3/mascot/cgi/peptide_view.pl?file=../data/20121119/F002569.dat&query=1498&hit=1&index=&px=1) | ***681.1219*** | ***1360.2293*** |  |  |  |  |  |  |  |
|  | [1503](http://df3/mascot/cgi/peptide_view.pl?file=../data/20121119/F002569.dat&query=1503&hit=1&index=&px=1) | ***683.0154*** | ***1364.0162*** |  |  |  |  |  |  |  |
|  | [1504](http://df3/mascot/cgi/peptide_view.pl?file=../data/20121119/F002569.dat&query=1504&hit=1&index=&px=1) | ***683.0920*** | ***1364.1694*** |  |  |  |  |  |  |  |
|  | [1507](http://df3/mascot/cgi/peptide_view.pl?file=../data/20121119/F002569.dat&query=1507&hit=1&index=&px=1) | ***683.7772*** | ***1365.5398*** |  |  |  |  |  |  |  |
|  | [1508](http://df3/mascot/cgi/peptide_view.pl?file=../data/20121119/F002569.dat&query=1508&hit=1&index=&px=1) | ***683.9177*** | ***1365.8209*** |  |  |  |  |  |  |  |
|  | [1509](http://df3/mascot/cgi/peptide_view.pl?file=../data/20121119/F002569.dat&query=1509&hit=1&index=&px=1) | ***683.9308*** | ***1365.8470*** |  |  |  |  |  |  |  |
|  | [1510](http://df3/mascot/cgi/peptide_view.pl?file=../data/20121119/F002569.dat&query=1510&hit=1&index=&px=1) | ***684.0300*** | ***1366.0455*** |  |  |  |  |  |  |  |
|  | [1511](http://df3/mascot/cgi/peptide_view.pl?file=../data/20121119/F002569.dat&query=1511&hit=1&index=&px=1) | ***684.0414*** | ***1366.0682*** |  |  |  |  |  |  |  |
|  | [1512](http://df3/mascot/cgi/peptide_view.pl?file=../data/20121119/F002569.dat&query=1512&hit=1&index=&px=1) | ***684.2867*** | ***1366.5589*** |  |  |  |  |  |  |  |
|  | [1513](http://df3/mascot/cgi/peptide_view.pl?file=../data/20121119/F002569.dat&query=1513&hit=1&index=&px=1) | ***684.6134*** | ***1367.2123*** |  |  |  |  |  |  |  |
|  | [1514](http://df3/mascot/cgi/peptide_view.pl?file=../data/20121119/F002569.dat&query=1514&hit=1&index=&px=1) | ***685.1254*** | ***1368.2362*** |  |  |  |  |  |  |  |
|  | [1515](http://df3/mascot/cgi/peptide_view.pl?file=../data/20121119/F002569.dat&query=1515&hit=1&index=&px=1) | ***685.1369*** | ***1368.2593*** |  |  |  |  |  |  |  |
|  | [1517](http://df3/mascot/cgi/peptide_view.pl?file=../data/20121119/F002569.dat&query=1517&hit=1&index=&px=1) | ***685.3595*** | ***1368.7044*** |  |  |  |  |  |  |  |
|  | [1523](http://df3/mascot/cgi/peptide_view.pl?file=../data/20121119/F002569.dat&query=1523&hit=1&index=&px=1) | ***687.1948*** | ***1372.3751*** |  |  |  |  |  |  |  |
|  | [1525](http://df3/mascot/cgi/peptide_view.pl?file=../data/20121119/F002569.dat&query=1525&hit=1&index=&px=1) | ***687.6907*** | ***1373.3669*** |  |  |  |  |  |  |  |
|  | [1526](http://df3/mascot/cgi/peptide_view.pl?file=../data/20121119/F002569.dat&query=1526&hit=1&index=&px=1) | ***688.0129*** | ***1374.0113*** |  |  |  |  |  |  |  |
|  | [1527](http://df3/mascot/cgi/peptide_view.pl?file=../data/20121119/F002569.dat&query=1527&hit=1&index=&px=1) | ***688.0942*** | ***1374.1739*** |  |  |  |  |  |  |  |
|  | [1528](http://df3/mascot/cgi/peptide_view.pl?file=../data/20121119/F002569.dat&query=1528&hit=1&index=&px=1) | ***689.5905*** | ***1377.1665*** |  |  |  |  |  |  |  |
|  | [1529](http://df3/mascot/cgi/peptide_view.pl?file=../data/20121119/F002569.dat&query=1529&hit=1&index=&px=1) | ***689.7777*** | ***1377.5409*** |  |  |  |  |  |  |  |
|  | [1530](http://df3/mascot/cgi/peptide_view.pl?file=../data/20121119/F002569.dat&query=1530&hit=1&index=&px=1) | ***690.5961*** | ***1379.1776*** |  |  |  |  |  |  |  |
|  | [1531](http://df3/mascot/cgi/peptide_view.pl?file=../data/20121119/F002569.dat&query=1531&hit=1&index=&px=1) | ***691.0543*** | ***1380.0941*** |  |  |  |  |  |  |  |
|  | [1532](http://df3/mascot/cgi/peptide_view.pl?file=../data/20121119/F002569.dat&query=1532&hit=1&index=&px=1) | ***691.5438*** | ***1381.0731*** |  |  |  |  |  |  |  |
[truncated: 162,150 more chars]
