# Supplementary material for: Src Tyrosine Kinase Activation by 4-Hydroxynonenal Upregulates p38, ERK/AP-1 Signaling and COX-2 Expression in YPEN-1 Cells
Source: PLoS One. 2015 Oct 14;10(10):e0129244. doi: 10.1371/journal.pone.0129244 (PMC4605600; doi:10.1371/journal.pone.0129244)
Supplement: S3 Data — (PPTX) [file pone.0129244.s003.pptx]

## Slide 1
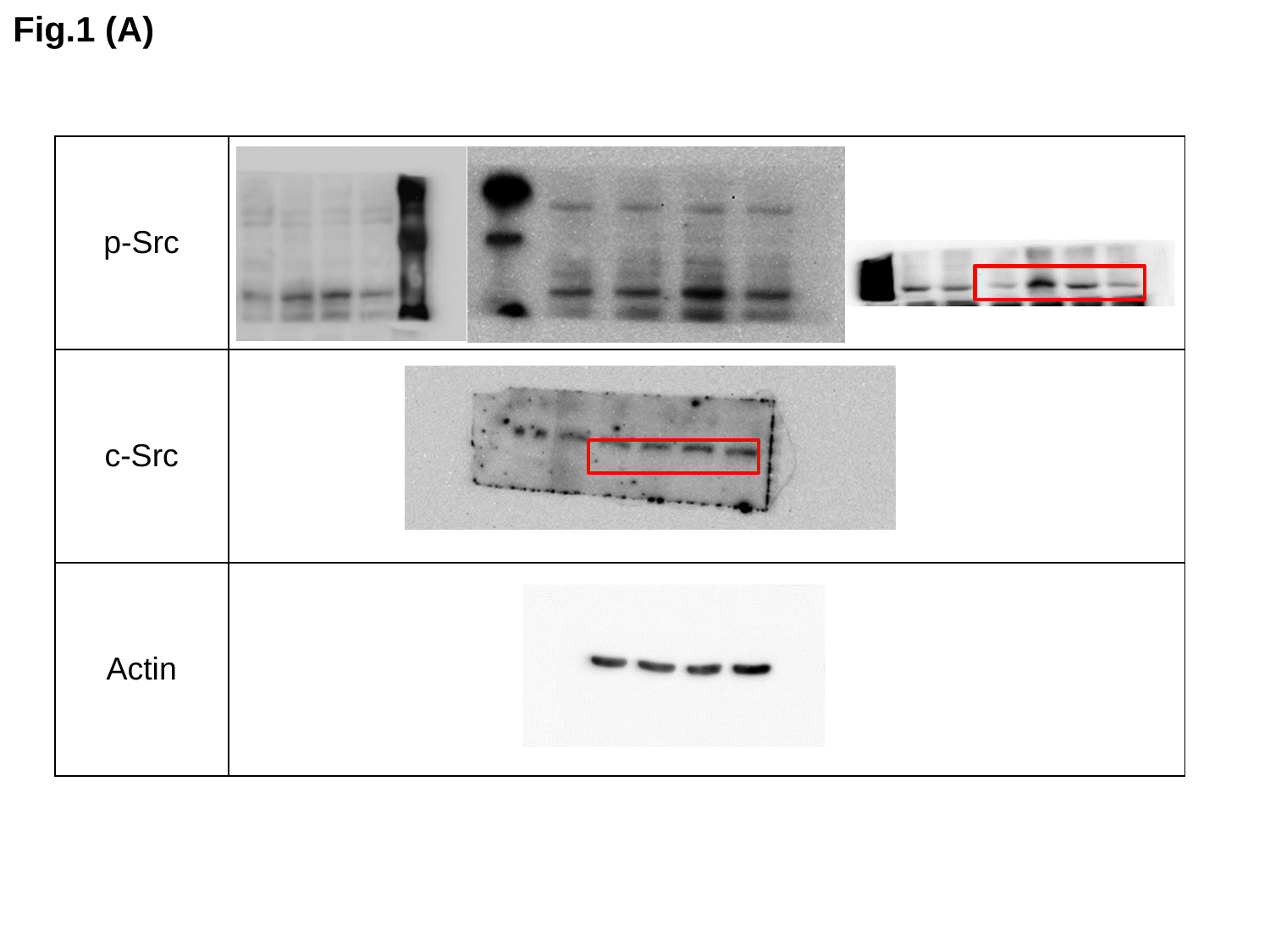

Fig.1 (A)
| p-Src | |
| --- | --- |
| c-Src | |
| Actin | |

## Slide 2
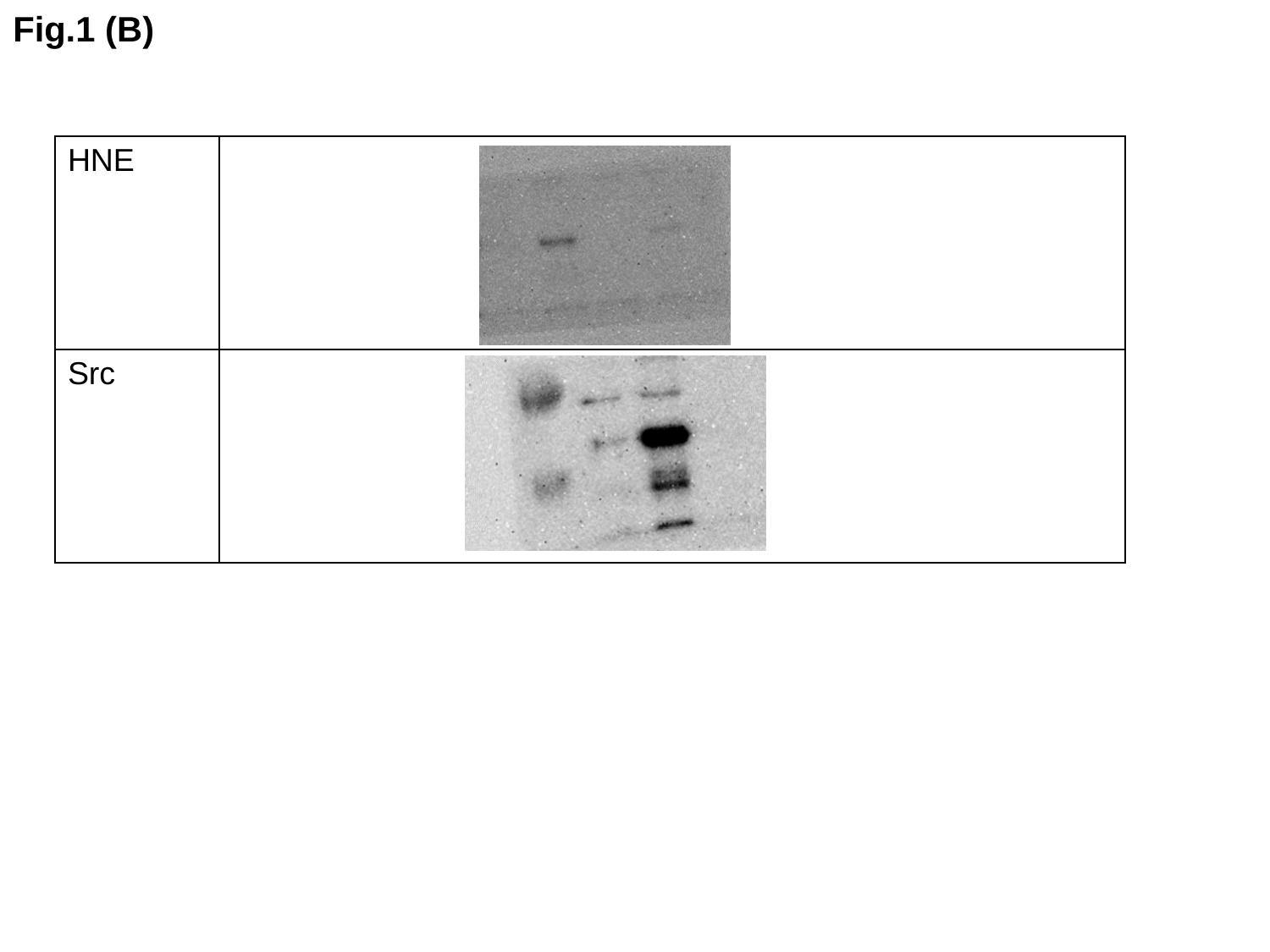

Fig.1 (B)
| HNE | |
| --- | --- |
| Src | |

## Slide 3
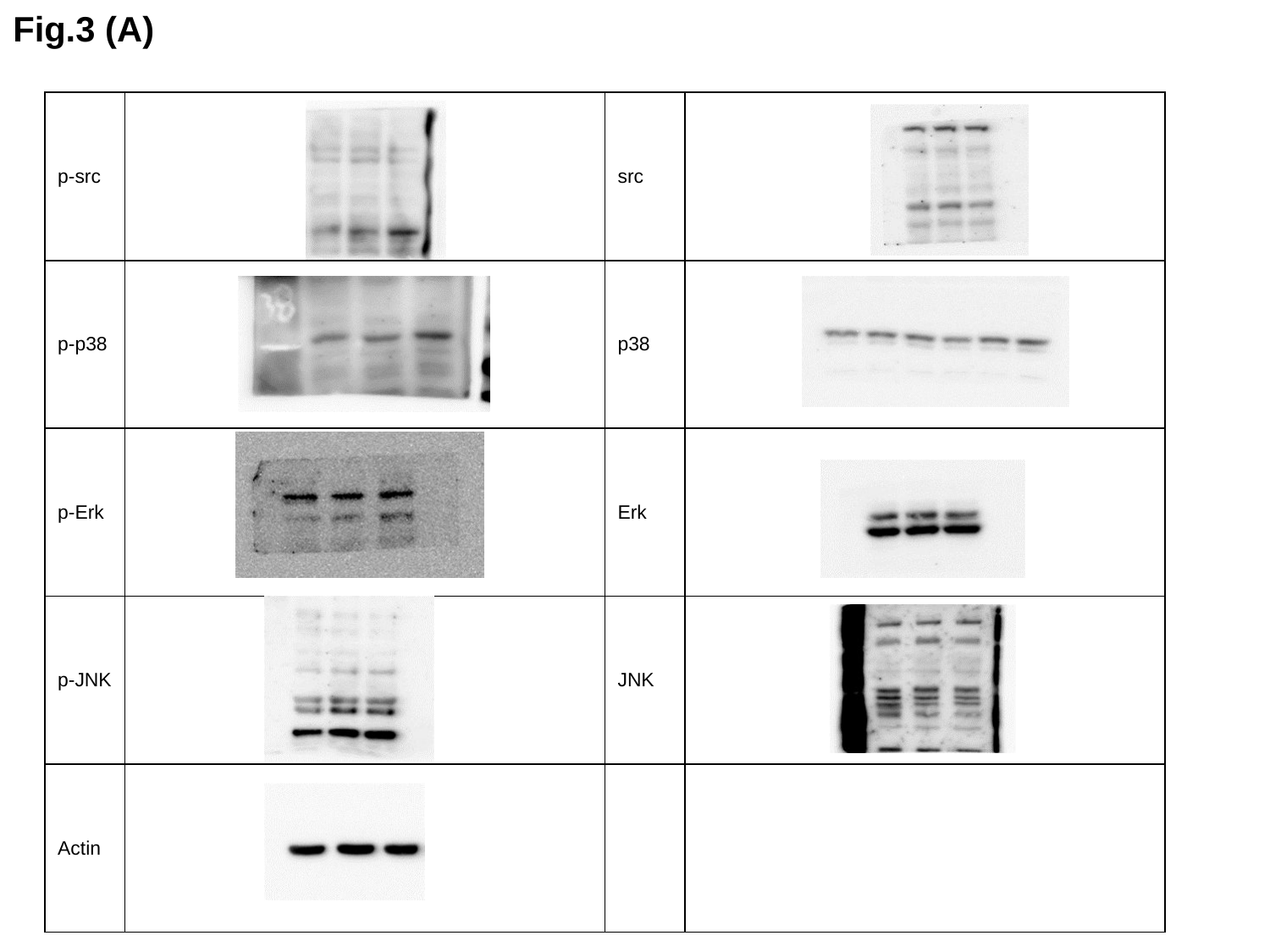

Fig.3 (A)
| p-src | | src | |
| --- | --- | --- | --- |
| p-p38 | | p38 | |
| p-Erk | | Erk | |
| p-JNK | | JNK | |
| Actin | | | |

## Slide 4
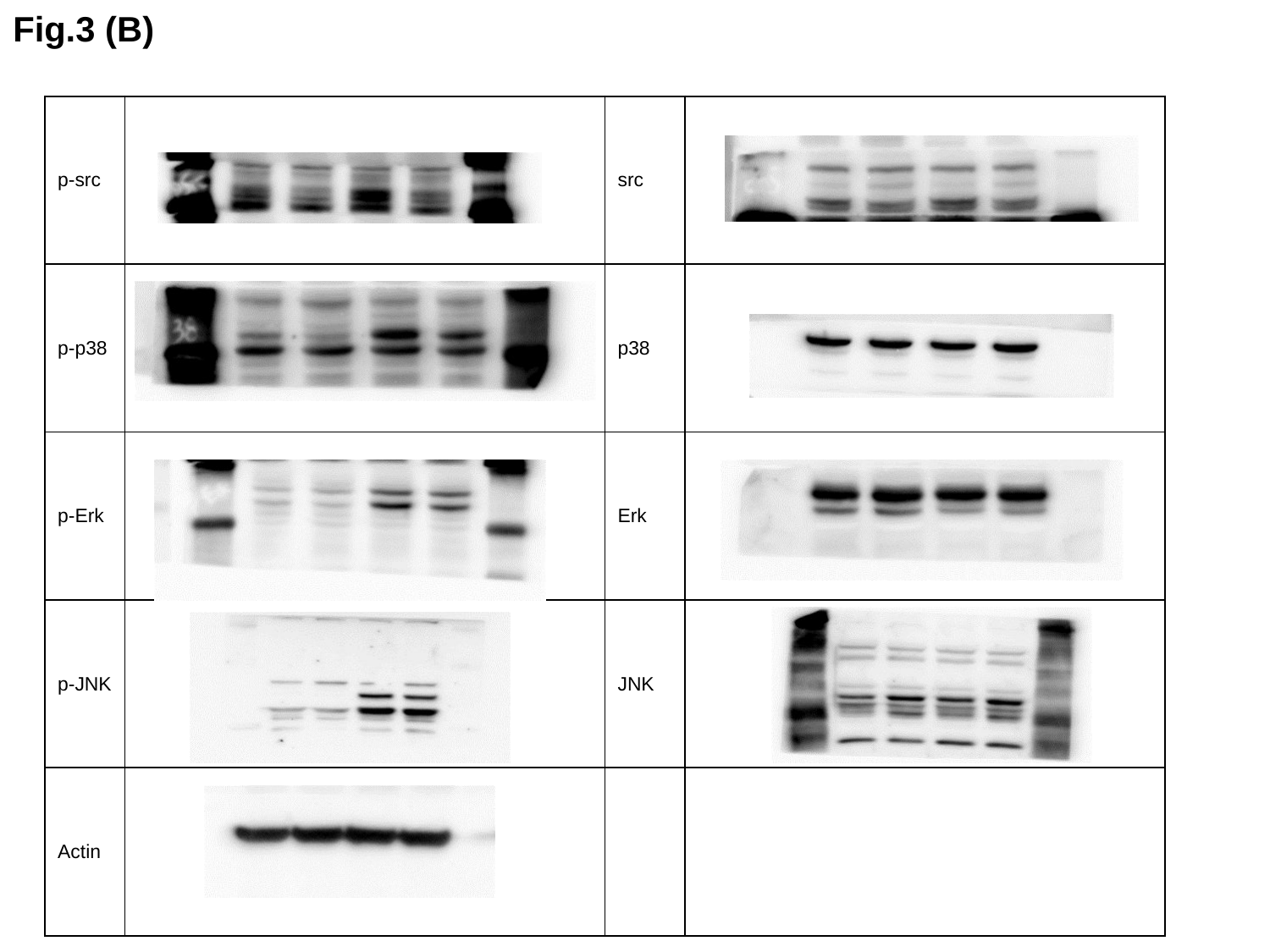

Fig.3 (B)
| p-src | | src | |
| --- | --- | --- | --- |
| p-p38 | | p38 | |
| p-Erk | | Erk | |
| p-JNK | | JNK | |
| Actin | | | |

## Slide 5
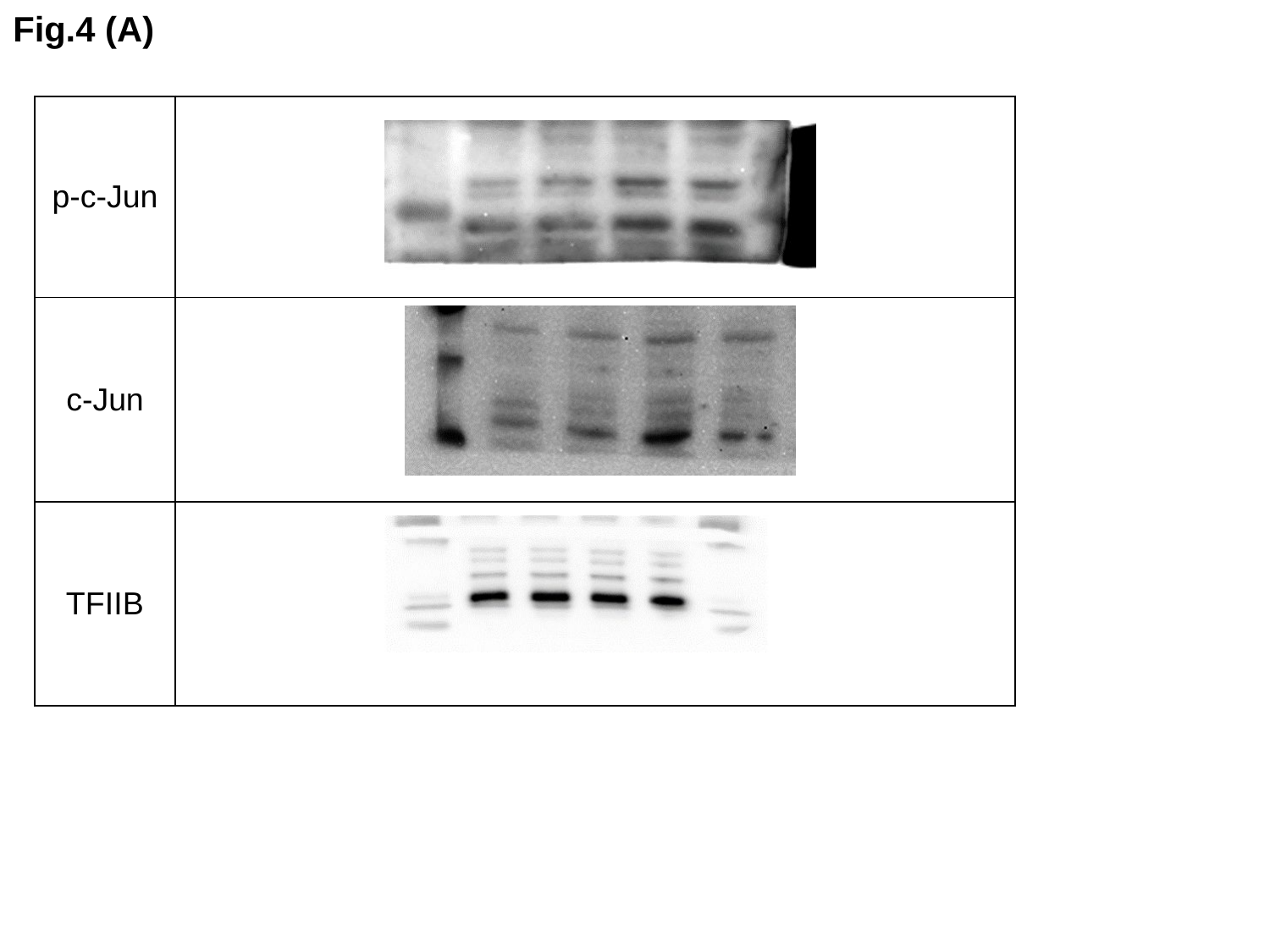

Fig.4 (A)
| p-c-Jun | |
| --- | --- |
| c-Jun | |
| TFIIB | |

## Slide 6
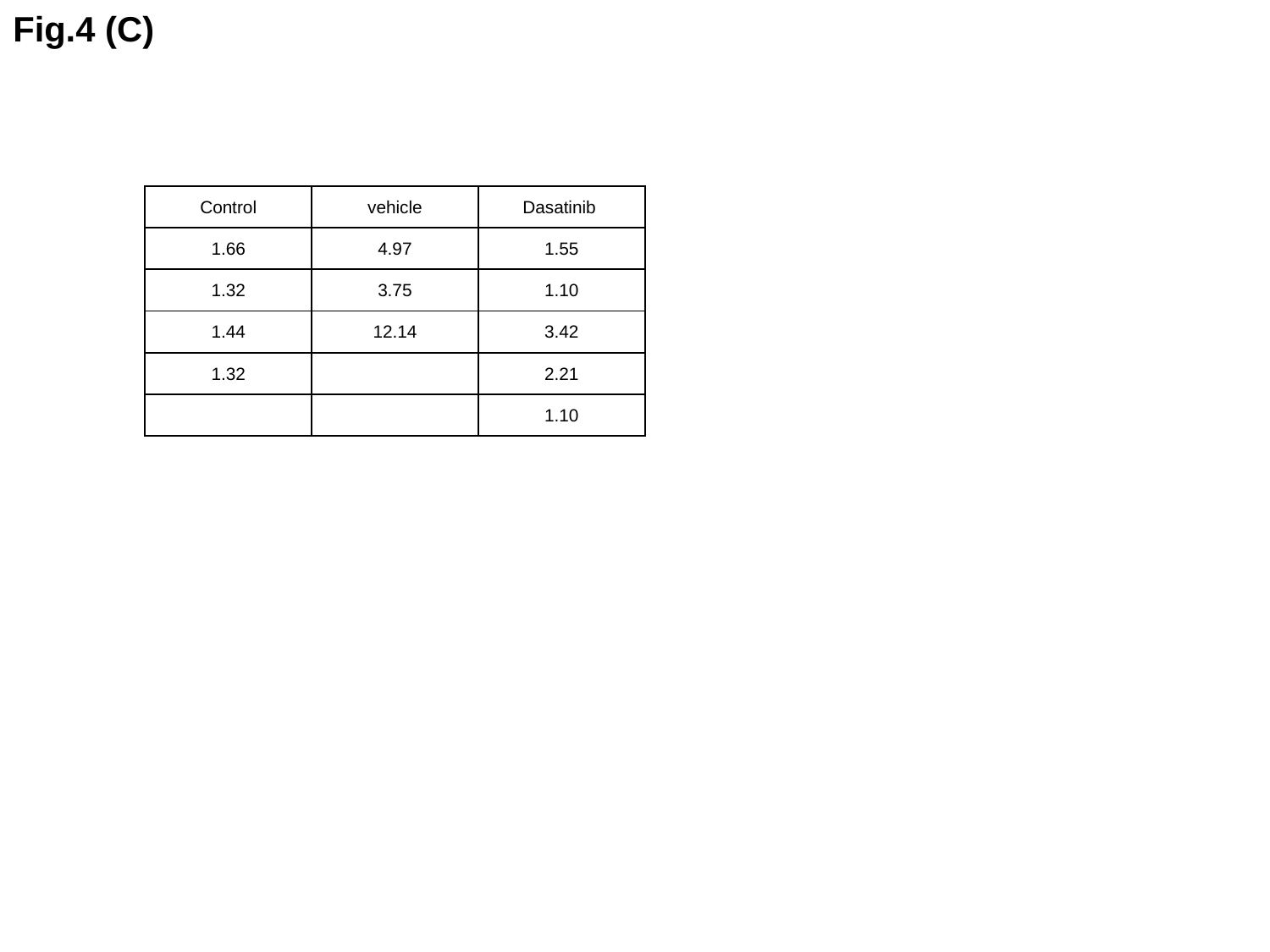

Fig.4 (C)
| Control | vehicle | Dasatinib |
| --- | --- | --- |
| 1.66 | 4.97 | 1.55 |
| 1.32 | 3.75 | 1.10 |
| 1.44 | 12.14 | 3.42 |
| 1.32 | | 2.21 |
| | | 1.10 |

## Slide 7
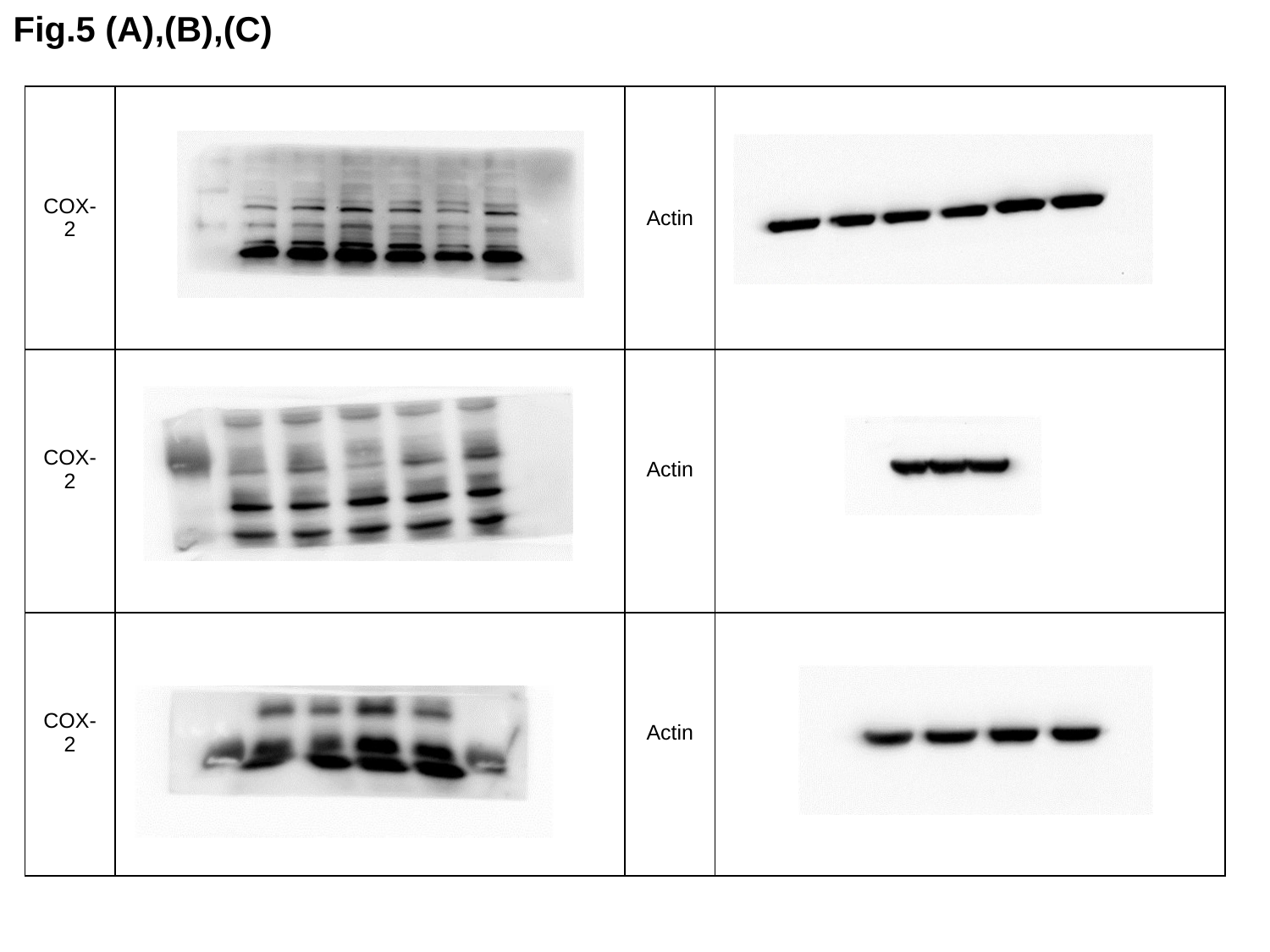

Fig.5 (A),(B),(C)
| COX-2 | | Actin | |
| --- | --- | --- | --- |
| COX-2 | | Actin | |
| COX-2 | | Actin | |
